# Supplementary material for: Shuffled ATG8 interacting motifs form an ancestral bridge between UFMylation and autophagy
Source: EMBO J. 2023 Feb 10;42(10):e112053. doi: 10.15252/embj.2022112053 (PMC10183829; doi:10.15252/embj.2022112053)
Supplement: Supplementary file 1 — Appendix [file EMBJ-42-e112053-s002.pdf]

Appendix table of contents

Appendix Fig. S1

Appendix Fig. S2

Appendix Fig. S3

Appendix Fig. S4

Appendix Fig. S5

Appendix Fig. S6

Appendix Fig. S7

Appendix Fig. S8

Appendix Fig. S9

Appendix Fig. S10

Appendix Fig. S11

Supplementary Data S1

Supplementary Data S2

Fig. S1

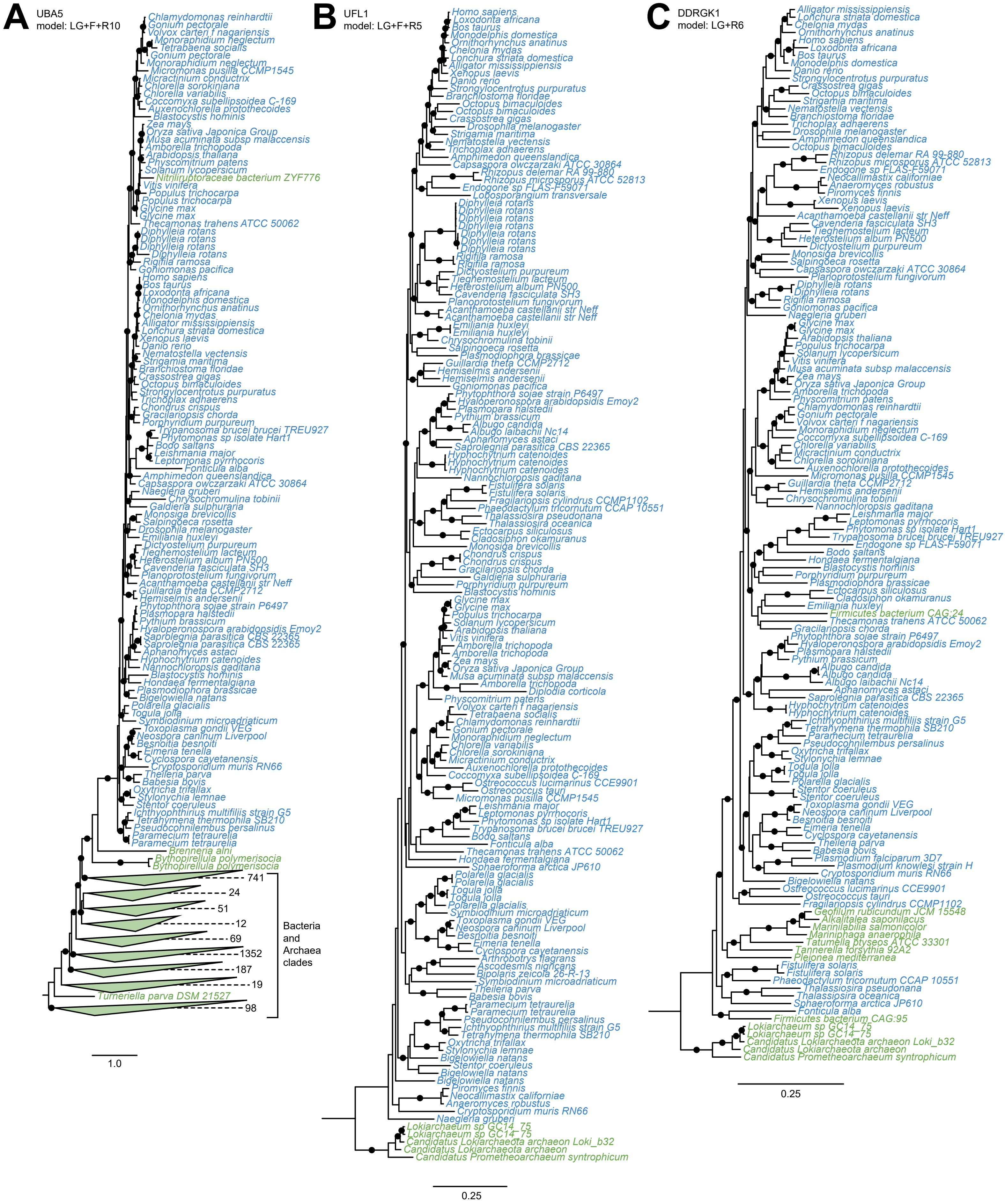

A
UBA5
model: LG+F+R10
Chlamydomonas reinhardtii
Gonium pectorale
Volvox carteri f nagariensis
Monoraphidium neglectum
Tetrabaena socialis
Gonium pectorale
Monoraphidium neglectum
Micromonas pusilla CCMP1545
Micractinium conductrix
Chlorella sorokiniana
Chlorella variabilis
Coccomyxa subellipsoidea C-169
Auxenochlorella protothecoides
Blastocystis hominis
Zea mays
Oryza sativa Japonica Group
Musa acuminata subsp malaccensis
Amborella trichopoda
Arabidopsis thaliana
Physcomitrium patens
Solanum lycopersicum
Nitriliruptoraceae bacterium ZYF776
Vitis vinifera
Populus trichocarpa
Populus trichocarpa
Glycine max
Glycine max
Thecamonas trahens ATCC 50062
Diphylleia rotans
Diphylleia rotans
Diphylleia rotans
Diphylleia rotans
Rigifila ramosa
Goniomonas pacifica
Homo sapiens
Bos taurus
Loxodonta africana
Monodelphis domestica
Ornithorhynchus anatinus
Chelonia mydas
Alligator mississippiensis
Lonchura striata domestica
Xenopus laevis
Danio rerio
Nematostella vectensis
Strigamia maritima
Branchiostoma floridae
Crassostrea gigas
Octopus bimaculoides
Strongylocentrotus purpuratus
Trichoplax adhaerens
Chondrus crispus
Gracilariopsis chorda
Porphyridium purpureum
Trypanosoma brucei brucei TREU927
Phytomonas sp isolate Hart1
Bodo saltans
Leishmania major
Leptomonas pyrrhocoris
Fonticula alba
Amphimedon queenslandica
Capsaspora owczarzaki ATCC 30864
Naegleria gruberi
Chrysochromulina tobinii
Galdieria sulphuraria
Monosiga brevicollis
Salpingoeca rosetta
Drosophila melanogaster
Emiliania huxleyi
Dictyostelium purpureum
Tieghemostelium lacteum
Heterostelium album PN500
Cavenderia fasciculata SH3
Planoprotostelium fungivorum
Acanthamoeba castellanii str Neff
Guillardia theta CCMP2712
Hemiselmis andersenii
Phytophthora sojae strain P6497
Plasmopara halstedii
Pythium brassicum
Hyaloperonospora arabidopsidis Emoy2
Saprolegnia parasitica CBS 22365
Saprolegnia parasitica CBS 22365
Aphanomyces astaci
Hyphochytrium catenoides
Nannochloropsis gaditana
Blastocystis hominis
Hondaea fermentalgiana
Plasmodiophora brassicae
Bigelowiella natans
Polarella glacialis
Togula jolla
Symbiodinium microadriaticum
Toxoplasma gondii VEG
Neospora caninum Liverpool
Besnoitia besnoiti
Eimeria tenella
Cyclospora cayetanensis
Cryptosporidium muris RN66
Theileria parva
Babesia bovis
Oxytricha trifallax
Stylonychia lemnae
Stentor coeruleus
Ichthyophthirius multifiliis strain G5
Tetrahymena thermophila SB210
Pseudocohnilembus persalinus
Paramecium tetraurelia
Paramecium tetraurelia
Brenneria alni
Bythopirellula polymerisocia
Bythopirellula polymerisocia
741
24
51
12
69
1352
187
19
Turneriella parva DSM 21527
98
Bacteria and Archaea clades
1.0
B
UFL1
model: LG+F+R5
Homo sapiens
Loxodonta africana
Bos taurus
Monodelphis domestica
Ornithorhynchus anatinus
Chelonia mydas
Lonchura striata domestica
Alligator mississippiensis
Xenopus laevis
Danio rerio
Strongylocentrotus purpuratus
Branchiostoma floridae
Octopus bimaculoides
Octopus bimaculoides
Crassostrea gigas
Drosophila melanogaster
Strigamia maritima
Nematostella vectensis
Trichoplax adhaerens
Amphimedon queenslandica
Capsaspora owczarzaki ATCC 30864
Rhizopus delemar RA 99-880
Rhizopus microsporus ATCC 52813
Endogone sp FLAS-F59071
Lobosporangium transversale
Diphylleia rotans
Diphylleia rotans
Diphylleia rotans
Diphylleia rotans
Diphylleia rotans
Diphylleia rotans
Diphylleia rotans
Rigifila ramosa
Rigifila ramosa
Dictyostelium purpureum
Tieghemostelium lacteum
Heterostelium album PN500
Cavenderia fasciculata SH3
Planoprotostelium fungivorum
Acanthamoeba castellanii str Neff
Acanthamoeba castellanii str Neff
Emiliania huxleyi
Emiliania huxleyi
Chrysochromulina tobinii
Salpingoeca rosetta
Plasmodiophora brassicae
Guillardia theta CCMP2712
Hemiselmis andersenii
Hemiselmis andersenii
Goniomonas pacifica
Phytophthora sojae strain P6497
Hyaloperonospora arabidopsidis Emoy2
Plasmopara halstedii
Pythium brassicum
Albugo candida
Albugo laibachii Nc14
Aphanomyces astaci
Saprolegnia parasitica CBS 22365
Hyphochytrium catenoides
Hyphochytrium catenoides
Hyphochytrium catenoides
Nannochloropsis gaditana
Fistulifera solaris
Fistulifera solaris
Fragilariopsis cylindrus CCMP1102
Phaeodactylum tricornutum CCAP 10551
Thalassiosira pseudonana
Thalassiosira oceanica
Ectocarpus siliculosus
Cladosiphon okamuranus
Monosiga brevicollis
Chondrus crispus
Chondrus crispus
Gracilariopsis chorda
Galdieria sulphuraria
Porphyridium purpureum
Blastocystis hominis
Glycine max
Glycine max
Populus trichocarpa
Solanum lycopersicum
Arabidopsis thaliana
Vitis vinifera
Amborella trichopoda
Amborella trichopoda
Zea mays
Oryza sativa Japonica Group
Musa acuminata subsp malaccensis
Amborella trichopoda
Diplodia corticola
Physcomitrium patens
Volvox carteri f nagariensis
Tetrabaena socialis
Chlamydomonas reinhardtii
Gonium pectorale
Monoraphidium neglectum
Chlorella variabilis
Chlorella sorokiniana
Micractinium conductrix
Auxenochlorella protothecoides
Coccomyxa subellipsoidea C-169
Ostreococcus lucimarinus CCE9901
Ostreococcus tauri
Micromonas pusilla CCMP1545
Leishmania major
Leptomonas pyrrhocoris
Phytomonas sp isolate Hart1
Trypanosoma brucei brucei TREU927
Bodo saltans
Fonticula alba
Thecamonas trahens ATCC 50062
Hondaea fermentalgiana
Sphaeroforma arctica JP610
Polarella glacialis
Polarella glacialis
Togula jolla
Togula jolla
Polarella glacialis
Symbiodinium microadriaticum
Toxoplasma gondii VEG
Neospora caninum Liverpool
Besnoitia besnoiti
Eimeria tenella
Cyclospora cayetanensis
Arthrobotrys flagrans
Ascodesmis nigricans
Bipolaris zeicola 26-R-13
Symbiodinium microadriaticum
Theileria parva
Babesia bovis
Paramecium tetraurelia
Paramecium tetraurelia
Pseudocohnilembus persalinus
Ichthyophthirius multifiliis strain G5
Tetrahymena thermophila SB210
Oxytricha trifallax
Stylonychia lemnae
Bigelowiella natans
Stentor coeruleus
Bigelowiella natans
Bigelowiella natans
Piromyces finnis
Neocallimastix californiae
Anaeromyces robustus
Cryptosporidium muris RN66
Naegleria gruberi
Lokiarchaeum sp GC14_75
Lokiarchaeum sp GC14_75
Candidatus Lokiarchaeota archaeon Loki_b32
Candidatus Lokiarchaeota archaeon
Candidatus Prometheoarchaeum syntrophicum
0.25
C
DDRGK1
model: LG+R6
Alligator mississippiensis
Lonchura striata domestica
Chelonia mydas
Ornithorhynchus anatinus
Homo sapiens
Loxodonta africana
Bos taurus
Monodelphis domestica
Danio rerio
Strongylocentrotus purpuratus
Crassostrea gigas
Octopus bimaculoides
Strigamia maritima
Nematostella vectensis
Branchiostoma floridae
Trichoplax adhaerens
Drosophila melanogaster
Amphimedon queenslandica
Octopus bimaculoides
Rhizopus delemar RA 99-880
Rhizopus microsporus ATCC 52813
Endogone sp FLAS-F59071
Neocallimastix californiae
Anaeromyces robustus
Piromyces finnis
Xenopus laevis
Xenopus laevis
Acanthamoeba castellanii str Neff
Cavenderia fasciculata SH3
Tieghemostelium lacteum
Heterostelium album PN500
Dictyostelium purpureum
Monosiga brevicollis
Salpingoeca rosetta
Capsaspora owczarzaki ATCC 30864
Planoprotostelium fungivorum
Diphylleia rotans
Diphylleia rotans
Rigifila ramosa
Goniomonas pacifica
Naegleria gruberi
Glycine max
Glycine max
Arabidopsis thaliana
Populus trichocarpa
Solanum lycopersicum
Vitis vinifera
Musa acuminata subsp malaccensis
Zea mays
Oryza sativa Japonica Group
Amborella trichopoda
Physcomitrium patens
Chlamydomonas reinhardtii
Gonium pectorale
Volvox carteri f nagariensis
Monoraphidium neglectum
Coccomyxa subellipsoidea C-169
Chlorella variabilis
Micractinium conductrix
Chlorella sorokiniana
Auxenochlorella protothecoides
Micromonas pusilla CCMP1545
Guillardia theta CCMP2712
Hemiselmis andersenii
Chrysochromulina tobinii
Nannochloropsis gaditana
Leishmania major
Leptomonas pyrrhocoris
Phytomonas sp isolate Hart1
Trypanosoma brucei brucei TREU927
Endogone sp FLAS-F59071
Bodo saltans
Hondaea fermentalgiana
Blastocystis hominis
Porphyridium purpureum
Plasmodiophora brassicae
Ectocarpus siliculosus
Cladosiphon okamuranus
Emiliania huxleyi
Firmicutes bacterium CAG:24
Thecamonas trahens ATCC 50062
Gracilariopsis chorda
Phytophthora sojae strain P6497
Hyaloperonospora arabidopsidis Emoy2
Plasmopara halstedii
Pythium brassicum
Albugo candida
Albugo candida
Albugo laibachii Nc14
Aphanomyces astaci
Saprolegnia parasitica CBS 22365
Hyphochytrium catenoides
Hyphochytrium catenoides
Ichthyophthirius multifiliis strain G5
Tetrahymena thermophila SB210
Paramecium tetraurelia
Pseudocohnilembus persalinus
Oxytricha trifallax
Stylonychia lemnae
Togula jolla
Togula jolla
Polarella glacialis
Stentor coeruleus
Stentor coeruleus
Toxoplasma gondii VEG
Neospora caninum Liverpool
Besnoitia besnoiti
Eimeria tenella
Cyclospora cayetanensis
Theileria parva
Babesia bovis
Plasmodium falciparum 3D7
Plasmodium knowlesi strain H
Cryptosporidium muris RN66
Bigelowiella natans
Ostreococcus lucimarinus CCE9901
Ostreococcus tauri
Fragilariopsis cylindrus CCMP1102
Geofilum rubicundum JCM 15548
Alkalitalea saponilacus
Marinilabilia salmonicolor
Mariniphaga anaerophila
Tatumella ptyseos ATCC 33301
Tannerella forsythia 92A2
Plejonea mediterranea
Fistulifera solaris
Fistulifera solaris
Phaeodactylum tricornutum CCAP 10551
Thalassiosira pseudonana
Thalassiosira oceanica
Sphaeroforma arctica JP610
Fonticula alba
Firmicutes bacterium CAG:95
Lokiarchaeum sp GC14_75
Lokiarchaeum sp GC14_75
Candidatus Lokiarchaeota archaeon Loki_b32
Candidatus Lokiarchaeota archaeon
Candidatus Prometheoarchaeum syntrophicum
0.25

Appendix Figure S1. The prokaryotic origins of UBA5, UFL1, and DDRGK1.

Maximum likelihood phylogenies generated from multiple sequence alignments containing eukaryotic and prokaryotic homologs of UBA5 (**A**), UFL1 (**B**), and DDRGK1 (**C**). Eukaryotes and prokaryotes are denoted in blue and green, respectively. Black circles represent SH-aLRT (Shimodaira Hasegawa approximate likelihood ratio tests) ≥ 0.85. Phylogenetic models used for generating each phylogeny and the number of sequences within collapsed nodes are noted. Scale bars represent the number of substitutions per 100 amino acids.

Fig. S2

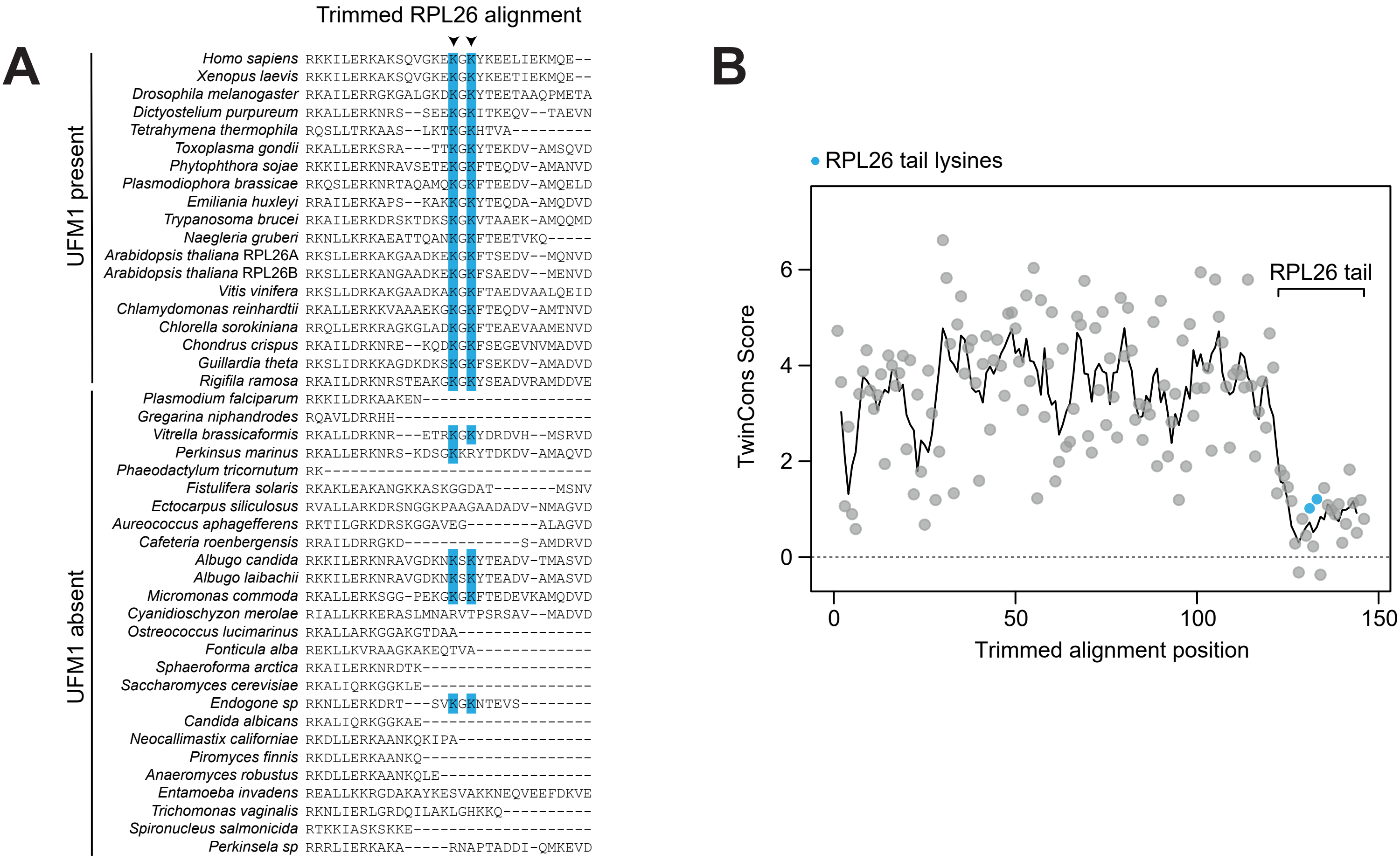

A
Trimmed RPL26 alignment
UFM1 present
Homo sapiens RKKILERKAKSQVGKEKGKYKEELIEKMQE--
Xenopus laevis RKKILERKAKSQVGKEKGKYKEETIEKMQE--
Drosophila melanogaster RKAILERRGKGALGKDKGKYTEETAAQPMETA
Dictyostelium purpureum RKALLERKNRS--SEEKGKITKEQV--TAEVN
Tetrahymena thermophila RQSLLTRKAAS--LKTKGKHTVA---------
Toxoplasma gondii RKALLERKSRA---TTKGKYTEKDV-AMSQVD
Phytophthora sojae RKKILERKNRAVSETEKGKFTEQDV-AMANVD
Plasmodiophora brassicae RKQSLERKNRTAQAMQKGKFTEEDV-AMQELD
Emiliania huxleyi RRAILERKAPS--KAKKGKYTEQDA-AMQDVD
Trypanosoma brucei RKAILERKDRSKTDKSKGKVTAAEK-AMQQMD
Naegleria gruberi RKNLLKRKAEATTQANKGKFTEETVKQ-----
Arabidopsis thaliana RPL26A RKSLLERKAKGAADKEKGKFTSEDV--MQNVD
Arabidopsis thaliana RPL26B RKSLLERKANGAADKEKGKFSAEDV--MENVD
Vitis vinifera RKSLLDRKAKGAADKAKGKFTAEDVAALQEID
Chlamydomonas reinhardtii RKALLERKKVAAAEKGKGKFTEQDV-AMTNVD
Chlorella sorokiniana RRQLLERKRAGKGLADKGKFTEAEVAAMENVD
Chondrus crispus RKAILDRKNRE--KQDKGKFSEGEVNVMADVD
Guillardia theta RKSLIDRKKAGDKDKSKGKFSEKDV-AMADVD
Rigifila ramosa RKAILDRKNRSTEAKGKGKYSEADVRAMDDVE
UFM1 absent
Plasmodium falciparum RKKILDRKAAKEN-------------------
Gregarina niphandrodes RQAVLDRRHH----------------------
Vitrella brassicaformis RKALLDRKNR---ETRKGKYDRDVH--MSRVD
Perkinsus marinus RKALLERKNRS-KDSGKKRYTDKDV-AMAQVD
Phaeodactylum tricornutum RK------------------------------
Fistulifera solaris RKAKLEAKANGKKASKGGDAT-------MSNV
Ectocarpus siliculosus RVALLARKDRSNGGKPAAGAADADV-NMAGVD
Aureococcus aphagefferens RKTILGRKDRSKGGAVEG--------ALAGVD
Cafeteria roenbergensis RRAILDRRGKD------------S-AMDRVD
Albugo candida RKKILERKNRAVGDKNKSKYTEADV-TMASVD
Albugo laibachii RKKILERKNRAVGDKNKSKYTEADV-AMASVD
Micromonas commoda RKALLERKSGG-PEKGKGKFTEDEVKAMQDVD
Cyanidioschyzon merolae RIALLKRKERASLMNARVTPSRSAV--MADVD
Ostreococcus lucimarinus RKALLARKGGAKGTDAA---------------
Fonticula alba REKLLKVRAAGKAKEQTVA-------------
Sphaeroforma arctica RKAILERKNRDTK-------------------
Saccharomyces cerevisiae RKALIQRKGGKLE-------------------
Endogone sp RKNLLERKDRT---SVKGKNTEVS--------
Candida albicans RKALIQRKGGKAE-------------------
Neocallimastix californiae RKDLLERKAANKQKIPA---------------
Piromyces finnis RKDLLERKAANKQ-------------------
Anaeromyces robustus RKDLLERKAANKQLE-----------------
Entamoeba invadens REALLKKRGDAKAYKESVAKKNEQVEEFDKVE
Trichomonas vaginalis RKNLIERLGRDQILAKLGHKKQ----------
Spironucleus salmonicida RTKKIASKSKKE--------------------
Perkinsela sp RRRLIERKAKA-----RNAPTADDI-QMKEVD
B
RPL26 tail lysines
RPL26 tail
TwinCons Score
0
2
4
6
0
50
100
150
Trimmed alignment position

Appendix Figure S2. Conservation analysis of RPL26 shows that the UFMylated tail region is divergent.

(A) **Multiple sequence alignment of RPL26 showing the conservation of the C-terminal tail in species with and without UFM1.** Lysine residues that are ufmylated have been highlighted. (**B) TwinCons analysis comparing the sequence conservation of RPL26.** The tail region is highly polymorphic.

Fig. S3

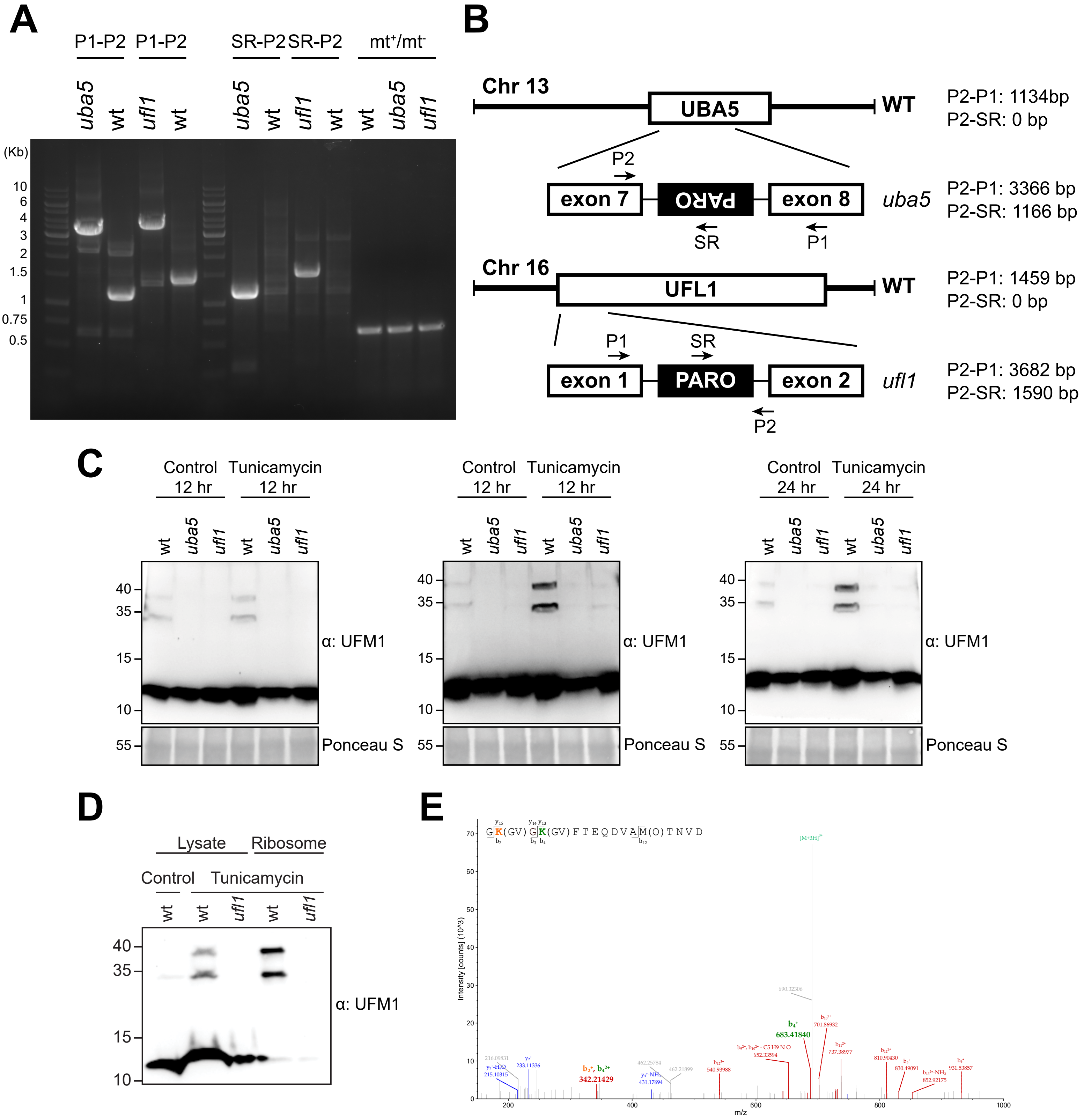
A
P1-P2
P1-P2
SR-P2
SR-P2
mt+/mt-
uba5
wt
ufl1
wt
uba5
wt
ufl1
wt
wt
uba5
ufl1
(Kb)
10
6
4
3
2
1.5
1
0.75
0.5
B
Chr 13
UBA5
WT
P2-P1: 1134bp
P2-SR: 0 bp
P2
exon 7
PARO
exon 8
SR
P1
uba5
P2-P1: 3366 bp
P2-SR: 1166 bp
Chr 16
UFL1
WT
P2-P1: 1459 bp
P2-SR: 0 bp
P1
SR
exon 1
PARO
exon 2
P2
ufl1
P2-P1: 3682 bp
P2-SR: 1590 bp
C
Control 12 hr
Tunicamycin 12 hr
Control 12 hr
Tunicamycin 12 hr
Control 24 hr
Tunicamycin 24 hr
wt
uba5
ufl1
40
35
15
10
55
α: UFM1
Ponceau S
D
Lysate
Ribosome
Control
Tunicamycin
wt
wt
ufl1
wt
ufl1
40
35
15
10
α: UFM1
E
GK(GV)GK(GV)FTEQDVAM(O)TNVD
Intensity [counts] (10^3)
m/z
[M+3H]3+
b4+
683.41840
b2+, b42+
342.21429
0
200
400
600
800
1000

Appendix Figure S3. Characterization of the *Chlamydomonas reinhardtii* UFMylation pathway mutants.

(A) Genotyping of *C. reinhardtii uba5* and *ufl1* mutants. *Left Panel*, mating type (mt +/-) and insertion site PCR products from purified genomic DNA samples prepared from wt, *uba5* and *ufl1* genotypes. PCR products were run on a 1% (w/v) agarose gel. DNA size markers are reported in Kb. **(B) Schematic diagram indicating the insertion site of the mutagenic cassette (PARO) in *ufl1* and *uba5* mutants.** Primers are indicated with arrows and expected PCR products from wild type and mutants are reported next to each respective diagram. **(C) RPL26 mono- and di-UFMylation is lost in *uba5* and *ufl1* mutants**. Cells were either left untreated or treated for 24 hours with 200 ng/mL tunicamycin. Protein extracts were analyzed by immunoblotting with anti-UFM1 antibodies. Total proteins were analyzed by Ponceau S staining. Quantification is shown in Figure 1C. **(D) *C. reinhardtii* (Cr) ribosomes are specifically mono- and di-UFMylated.** Liquid TAP cultures were either left untreated (control) or treated for 24 hours with 200 ng/mL tunicamycin. Protein extracts and purified ribosomes were analyzed by immunoblotting with anti-UFM1 antibodies. (E) ***C. reinhardtii* (Cr) RPL26 is di-UFMylated.** nanoLC-MS/MS spectra of K-GV-containing peptides associated with RPL26. Spectrum, derived from RPL26-$(UFM1)_2$, is of a RPL26 peptide containing K-GV remnants on K131 and K133, and oxidation of M141.

Fig. S4

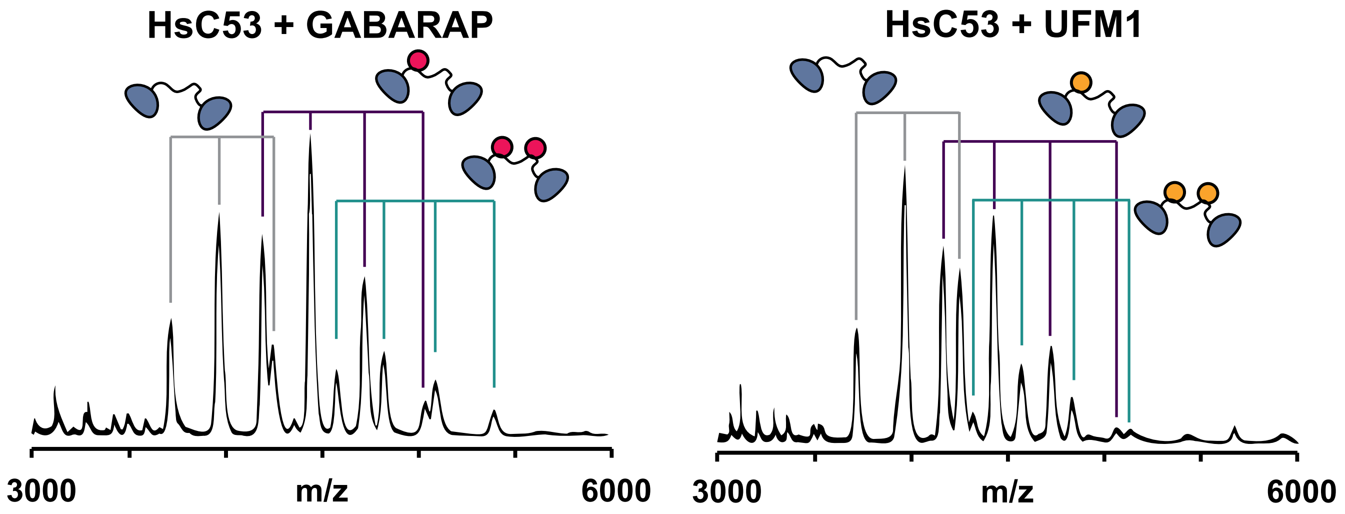

Appendix Figure S4. Native Mass-Spectrometry (nMS) spectra of HsC53 with GABARAP or HsUFM1 show very similar binding profiles.

Left Panel, GABARAP (4 µM) and HsC53 (2 µM). *Right Panel*, HsUFM1 (4 µM) and HsC53 (2 µM). Binding of HsC53 to GABARAP and HsUFM1 is observed in 1:1 (violet) and 1:2 ratios (teal)**.**

Fig. S5

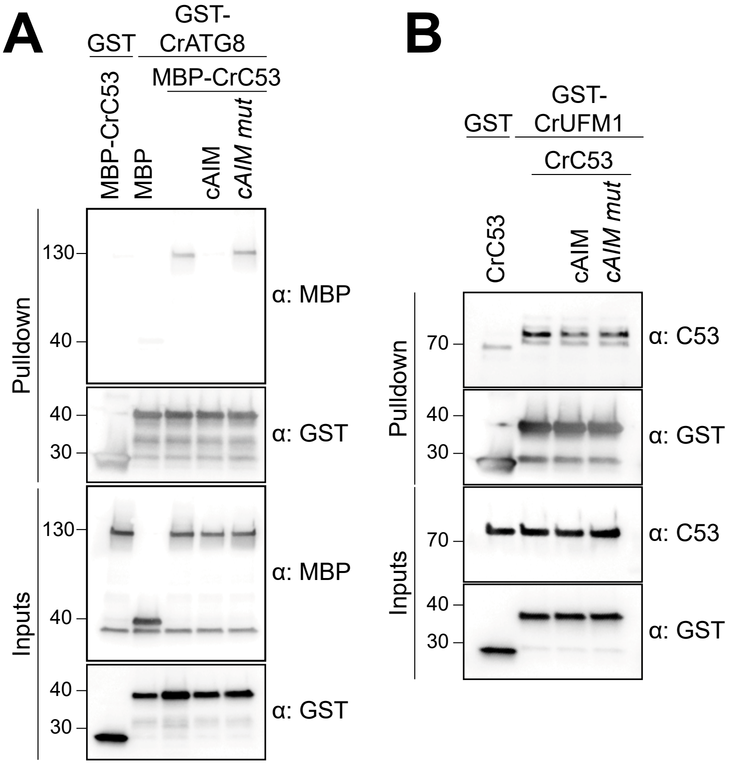
A
GST
GST-
CrATG8
MBP-CrC53
MBP-CrC53
MBP
cAIM
cAIM mut
Pulldown
Inputs
130
40
30
α: MBP
α: GST
B
GST
GST-
CrUFM1
CrC53
CrC53
cAIM
cAIM mut
70
α: C53

Appendix Figure S5. The canonical ATG8 Interacting Motif (cAIM) peptide cannot outcompete C53-UFM1 interaction for *C. reinhardtii* (Cr) protein orthologs.

(A) CrC53 binds CrATG8A in a cAIM-dependent manner. (B) CrC53 binds UFM1 in a cAIM-independent manner. Bacterial lysates containing recombinant protein or purified recombinant proteins were mixed and pulled down with glutathione magnetic agarose beads. Input and bound proteins were visualized by immunoblotting with anti-GST, anti-MBP or anti-AtC53 antibodies. cAIM wild type or mutant peptides were used to a final concentration of 200 μM.

Fig. S6

A

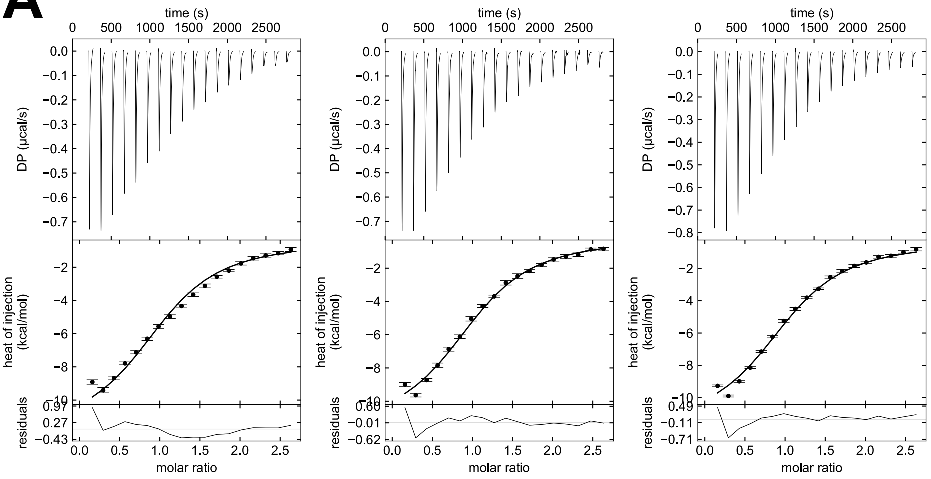

	Best-fit value	68.3% Confidence interval
K_D	7.51	5.30, 10.97
ΔH	-11.98	-14.31, -10.40

B

1 2

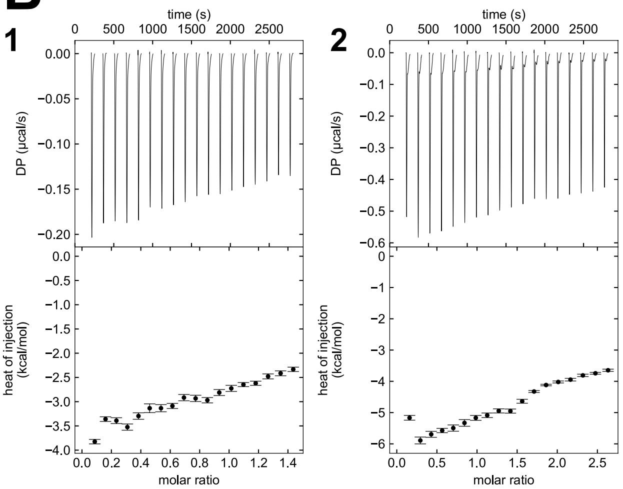

Appendix Figure S6. Titrations of HsUFM1 with UBA5 LIR peptide and HsC53^{wt}.

(A) Titrations of HsUFM1 with UBA5 LIR. The concentrations of reactants are 40 μM for UFM1 (in cell) and 550 μM UBA5 LIR (in syringe). **(B) Titrations of HsUFM1 with HsC53^{wt}** The concentrations of reactants are 40 μM for UFM1 (in cell) and 300 μM (**1**) or 550 μM HsC53^{wt} (in syringe) (**2**). Global analysis was performed using a hetero-association model A + B. The top panels show the SVD reconstructed thermograms, the middle panel shows the isotherms, and the bottom panel shows the residuals. Extracted global parameters and their 68.3% confidence interval are reported in the respective tables. Thermograms were reconstructed with NITPIC, global analysis was done in SEDPHAT, and data visualization was plotted in GUSSI. The dissociation constant (K_D) is reported μM units, while the enthalpy (ΔH) is reported in kcal/mol units.

Fig. S7

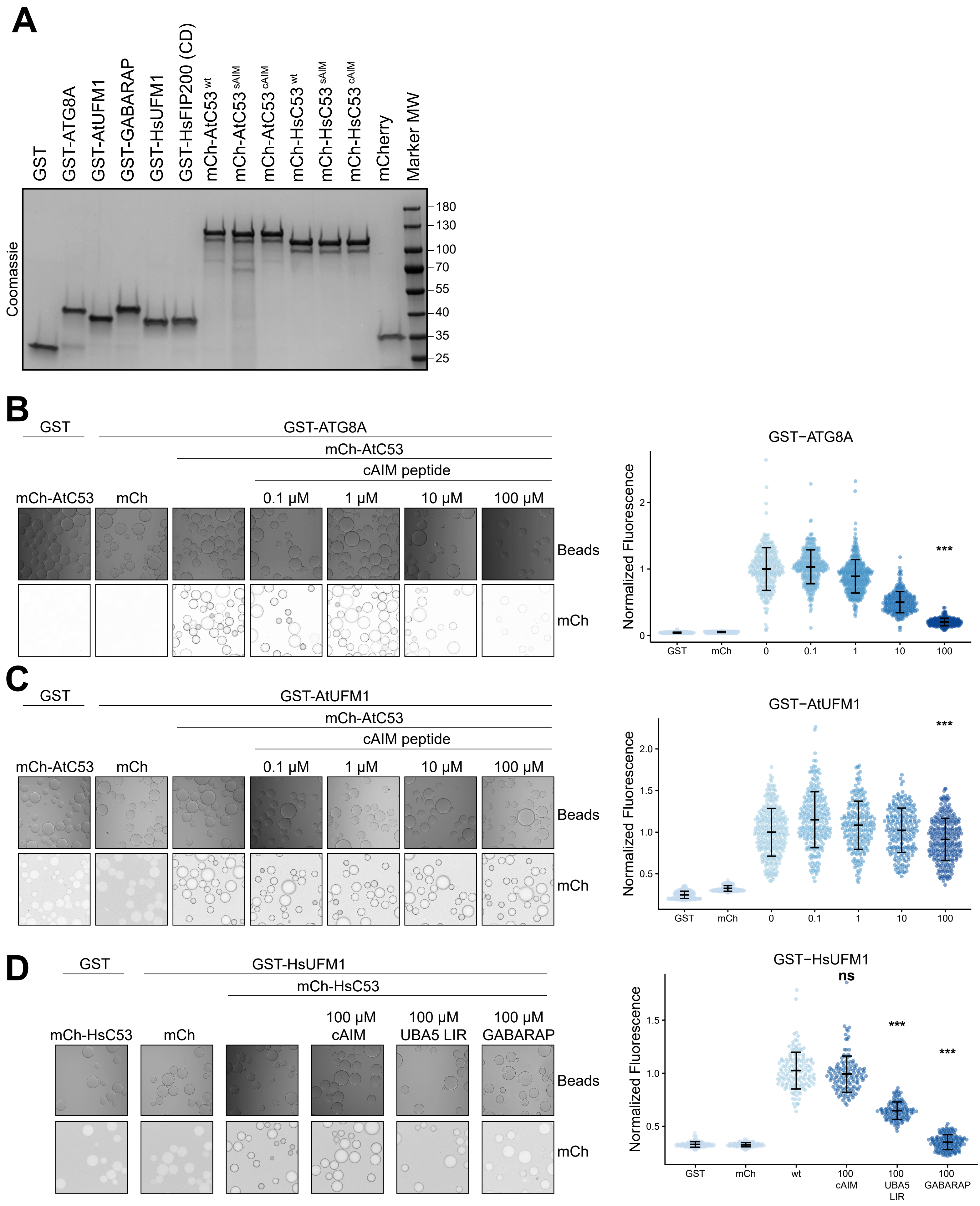

A
GST
GST-ATG8A
GST-AtUFM1
GST-GABARAP
GST-HsUFM1
GST-HsFIP200 (CD)
mCh-AtC53 wt
mCh-AtC53 sAIM
mCh-AtC53 cAIM
mCh-HsC53 wt
mCh-HsC53 sAIM
mCh-HsC53 cAIM
mCherry
Marker MW
Coomassie
180
130
100
70
55
40
35
25
B
GST
GST-ATG8A
mCh-AtC53
cAIM peptide
mCh-AtC53
mCh
0.1 µM
1 µM
10 µM
100 µM
Beads
mCh
GST−ATG8A
Normalized Fluorescence
***
GST
mCh
0
0.1
1
10
100
C
GST
GST-AtUFM1
mCh-AtC53
cAIM peptide
mCh-AtC53
mCh
0.1 µM
1 µM
10 µM
100 µM
Beads
mCh
GST−AtUFM1
Normalized Fluorescence
***
D
GST
GST-HsUFM1
mCh-HsC53
mCh-HsC53
mCh
100 µM cAIM
100 µM UBA5 LIR
100 µM GABARAP
Beads
mCh
GST−HsUFM1
ns
***
***
Normalized Fluorescence
GST
mCh
wt
100 cAIM
100 UBA5 LIR
100 GABARAP

Appendix Figure S7. The canonical ATG8 Interacting Motif (cAIM) cannot outcompete C53-UFM1 interaction.

(A) Purified proteins used for the protein-protein interaction microscopy binding assays. Recombinant proteins were analyzed for purity by SDS-PAGE followed by Coomassie staining. Marker molecular weights (MW) are indicated in kDa. mCh: mCherry. **(B, C) Microscopy-based protein–protein interaction assays showing unlike ATG8A-C53 interaction, UFM1-C53 interaction is insensitive to cAIM peptide competition**. Glutathione-sepharose beads were prepared by incubating them with GST-ATG8A (C) or GST-AtUFM1 (D). The pre-assembled beads were then washed and mixed with 1 µM of AtC53 containing increasing concentrations of cAIM peptide (0-100 µM). The beads were then imaged using a confocal microscope. *Left Panel,* representative confocal images (inverted grayscale) for each condition are shown. *Right panel*, normalized fluorescence is shown for each condition with the mean (± SD) of 2 independent replicates containing 2 technical replicates. Unpaired two-samples Wilcoxon test with continuity correction was performed to analyze the differences between wild type without cAIM peptide and wild type with 100 µM cAIM peptide. ***, p-value < 0.001. Total number of beads, mean, median, standard deviation and p-values are reported in Supplementary data S1. **(D) Microscopy-based protein–protein interaction assays showing UBA5 LIR peptide and GABARAP can compete for C53 interaction with UFM1**. Glutathione-sepharose beads were prepared by incubating them with GST-HsUFM1. The pre-assembled beads were then washed and mixed with 1 µM of HsC53 with either 100 µM cAIM peptide, 100 µM UBA5 LIR peptide or 100 µM GABARAP. The beads were then imaged using a confocal microscope. *Left Panel,* representative confocal images (inverted grayscale) for each condition are shown. *Right panel*, normalized fluorescence is shown for each condition with the mean (± SD). Unpaired two-samples Wilcoxon test with continuity correction was performed to analyze the differences between wild type and wild type mixed with either cAIM peptide, UBA5 LIR peptide or GABARAP. ns, p-value > 0.05, ***, p-value < 0.001. Total number of beads, mean, median, standard deviation and p-values are reported in Supplementary data S1.

Fig. S8

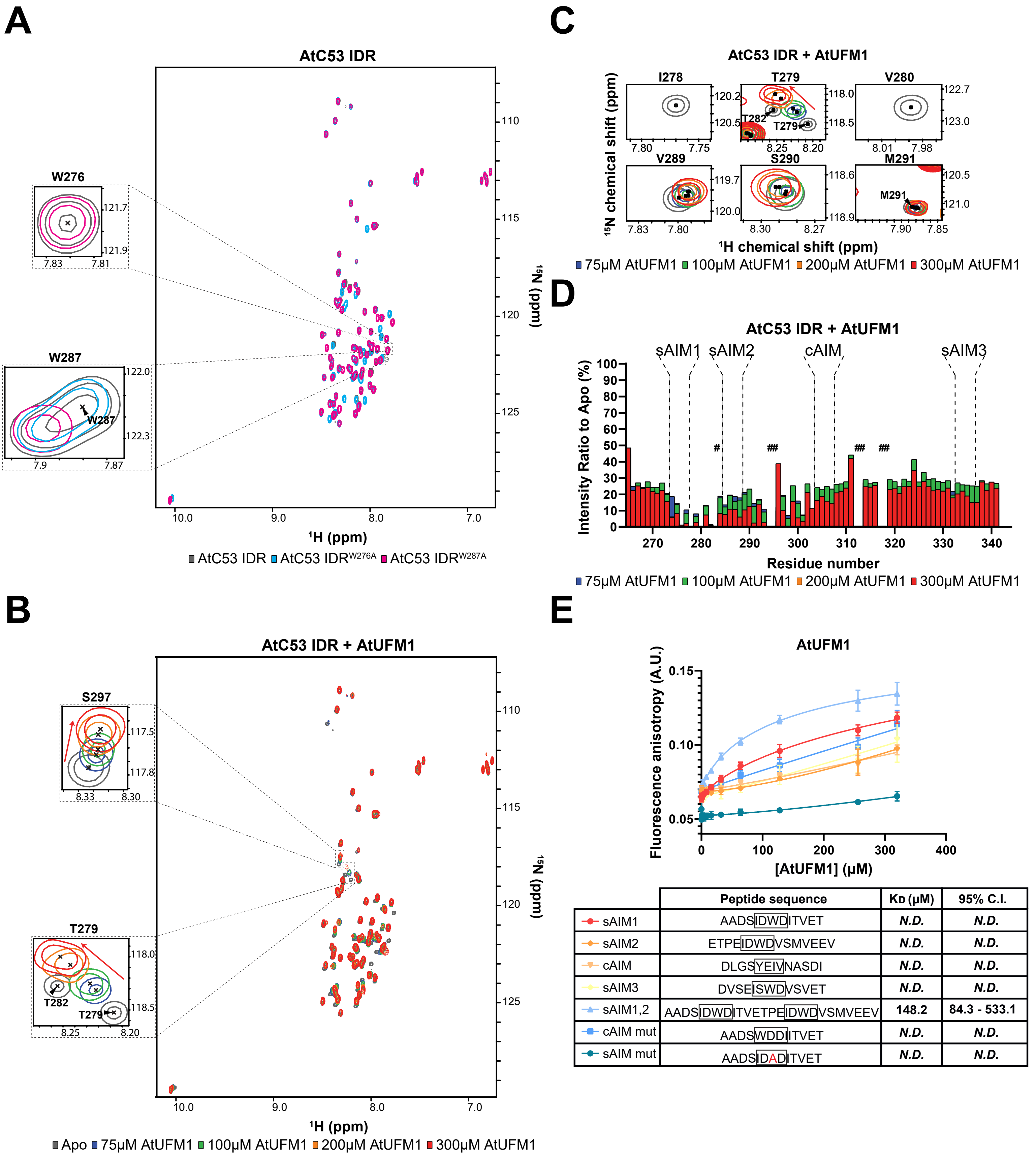

	Peptide sequence	KD (µM)	95% C.I.
sAIM1	AADSIDWDITVET	*N.D.*	*N.D.*
sAIM2	ETPEIDWDVSMVEEV	*N.D.*	*N.D.*
cAIM	DLGSYEIVNASDI	*N.D.*	*N.D.*
sAIM3	DVSEISWDVSVET	*N.D.*	*N.D.*
sAIM1,2	AADSIDWDITVETPEIDWDVSMVEEV	148.2	84.3 - 533.1
cAIM mut	AADSWDDIITVET	*N.D.*	*N.D.*
sAIM mut	AADSIDADITVET	*N.D.*	*N.D.*

Appendix Figure S8. Structural characterization of AtC53 IDR binding to AtUFM1 using NMR spectroscopy.

(A) Validation of AtC53 IDR backbone resonance assignments. Overlaid ^{1}H-^{15}N HSQC spectra of 100 µM isotope-labelled AtC53 IDR (grey), AtC53 IDR^{W276A} (cyan) and AtC53 IDR^{W287A} (magenta). Insets of resonances corresponding to residues W276 and W287 are shown. **(B) Addition of AtUFM1 changes the magnetic resonance of specific residues in AtC53.** Overlaid ^{1}H-^{15}N HSQC spectra of isotope-labelled AtC53 IDR (100 µM) in their free (gray) or bound state to 75 µM (blue), 100 µM (green), 200 µM (orange) and 300 µM (red) unlabelled AtUFM1. Examples of individual peaks that shift upon binding are shown as insets. Chemical shifts are indicated with arrows. **(C) Residues downstream AtC53 sAIM1 but not sAIM2 contribute to AtUFM1 binding.** Insets of overlaid ^{1}H-^{15}N HSQC spectra of isotope-labelled AtC53 IDR (100 µM) showing chemical shift perturbations of individual peaks from backbone amides of AIM residues in their free (gray) or bound state to 75 µM (blue), 100 µM (green), 200 µM (orange) and 300 µM (red) unlabelled AtUFM1. Chemical shifts are indicated with arrows. **(D) Signal intensity changes in AtC53 IDR upon binding of AtUFM1 are concentration dependent.** Intensity ratio broadening of AtC53 IDR (100 µM) in the presence of 75 µM (blue), 100 µM (green), 200 µM (orange) and 300 µM (red) AtUFM1. Bars corresponding to residues in AIMs are highlighted. Unassigned AtC53 IDR residues are indicated by hashtags. **(E) A peptide spanning C53 sAIM1 and sAIM2 binds UFM1.** Fluorescence anisotropy (FA) assay using AtC53 IDR-derived peptides as a probe for AtUFM1 binding. Error bars represent the FA values expressed in arbitrary units (A.U.) as mean ± s.d. of triplicate experiments using the same buffer and protein conditions. Peptide sequences are listed, sAIMs are highlighted. N.D. not determined.

Fig. S9

A

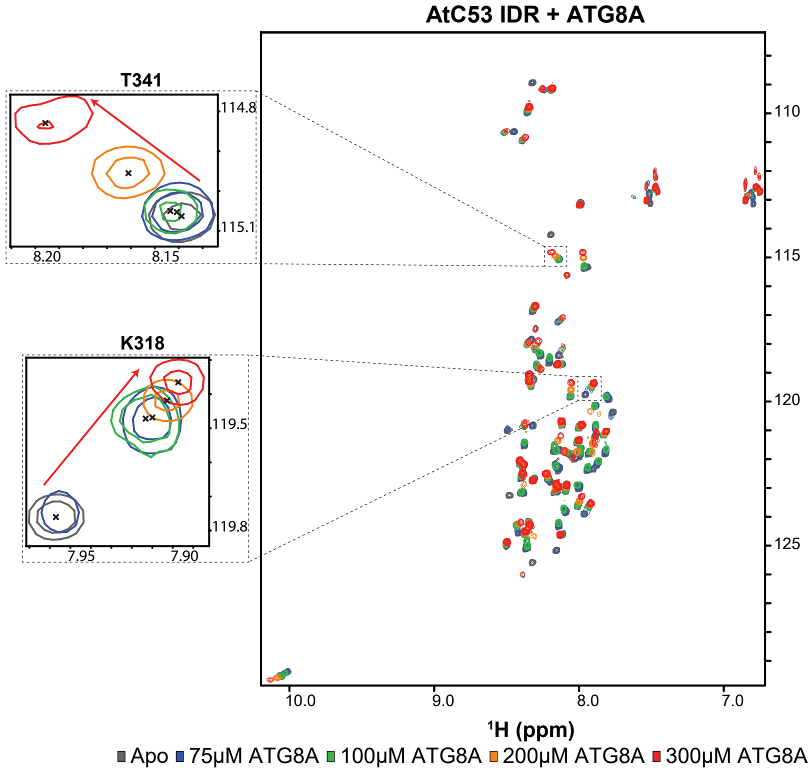

B

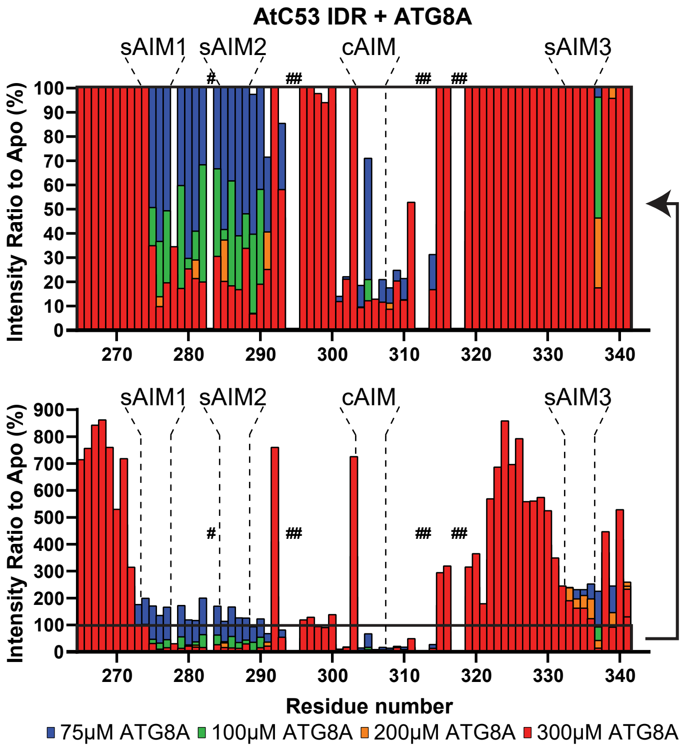

C

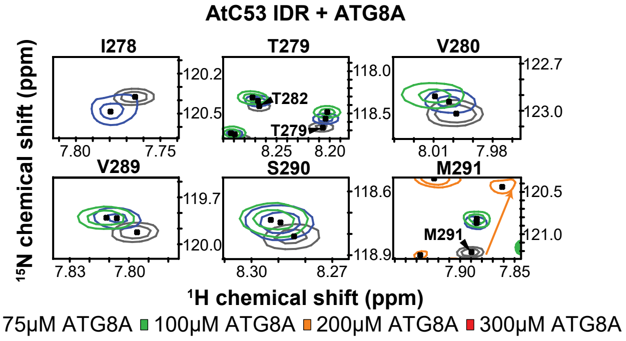

D

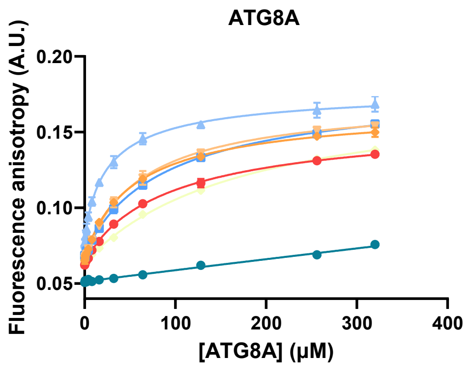

	Peptide sequence	KD (μM)	95% C.I.
sAIM1	AADSIDWDITVET	97.59	77.91 - 133.0
sAIM2	ETPEIDWDVSMVEEV	56.82	50.14 - 66.01
cAIM	DLGSYEIVNASDI	62.47	53.80 - 75.25
sAIM3	DVSEISWDVSVET	176.5	137.6 - 249.7
sAIM1,2	AADSIDWDITVETPEIDWDVSMVEEV	26.54	21.81 - 33.87
cAIM mut	AADSWDDITVET	94.27	77.47 - 122.2
sAIM mut	AADSIDADITVET	*N.D.*	*N.D.*

Appendix Figure S9. Structural characterization of AtC53 IDR binding to ATG8A using NMR spectroscopy.

(A) Addition of ATG8A affects a greater number of residues in the AtC53 IDR spectra. Overlaid ^{1}H-^{15}N HSQC spectra of isotope-labelled AtC53 IDR (100 µM) in their free (gray) or bound state to 75 µM (blue), 100 µM (green), 200 µM (orange) and 300 µM (red) unlabelled ATG8A. Insets of individual peaks that shifted upon binding are shown. Chemical shifts are indicated with arrows. **(B) Signal intensity changes in AtC53 IDR upon binding of ATG8A are concentration dependent.** Intensity ratio broadening of AtC53 IDR (100 µM) in the presence of 75 µM (blue), 100 µM (green), 200 µM (orange) and 300 µM (red) ATG8A. Top panel represents an inset of lower panel. Unassigned AtC53 IDR residues are indicated by hashtags. Bars corresponding to residues in AIMs are highlighted. Top panel represents an inset of lower panel. **(C) Residues downstream AtC53 sAIM1 and sAIM2 contribute to ATG8A binding.** Insets of overlaid ^{1}H-^{15}N HSQC spectra of isotope-labelled AtC53 IDR (100 µM) showing chemical shift perturbations of individual peaks from backbone amides of AIM residues in their free (gray) or bound state to 75 µM (blue), 100 µM (green), 200 µM (orange) and 300 µM (red) unlabelled ATG8A. Chemical shifts are indicated with arrows. **(D) A peptide spanning C53 sAIM1 and sAIM2 binds ATG8A.** Fluorescence anisotropy (FA) assay using AtC53 IDR-derived peptides as a probe for ATG8A binding. Error bars represent the FA values expressed in arbitrary units (A.U.) as mean ± s.d. of triplicate experiments using the same buffer and protein conditions. Peptide sequences are listed, sAIMs are highlighted. N.D. not determined.

Fig. S10

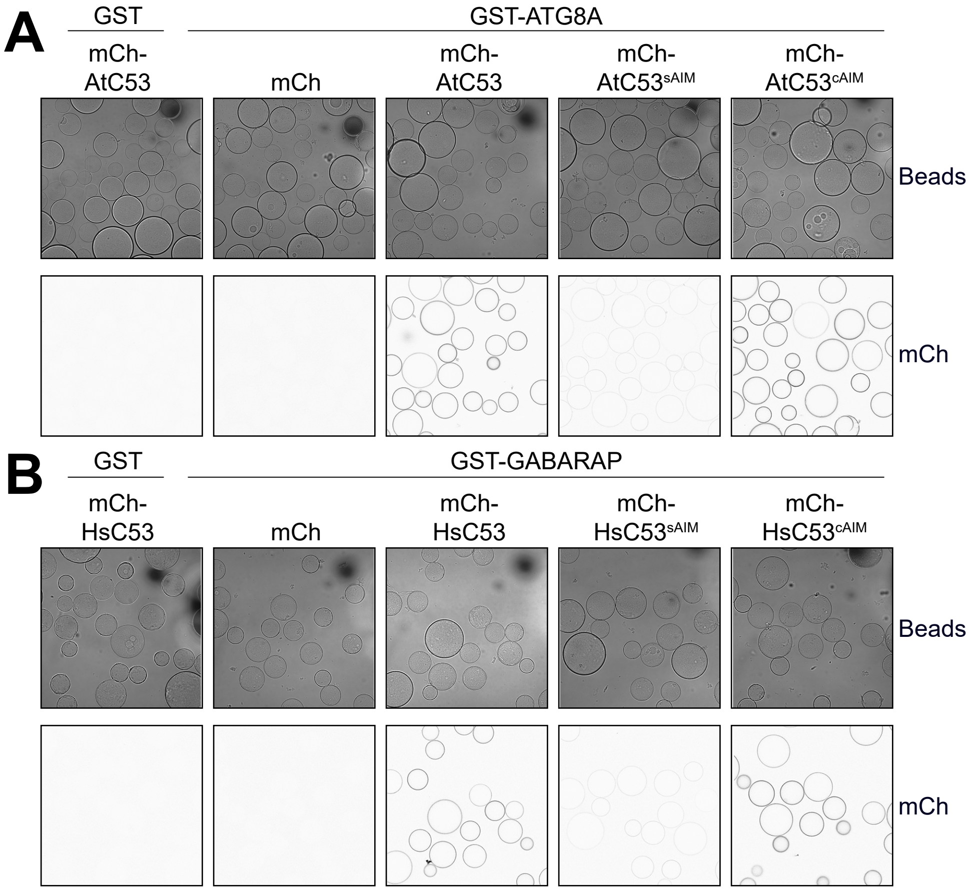

A
GST
GST-ATG8A
mCh-AtC53
mCh
mCh-AtC53
mCh-AtC53sAIM
mCh-AtC53cAIM
Beads
mCh
B
GST
GST-GABARAP
mCh-HsC53
mCh
mCh-HsC53
mCh-HsC53sAIM
mCh-HsC53cAIM
Beads
mCh

Appendix Figure S10. Microscopy-based protein–protein interaction assays showing $C53^{cAIM}$ has increased affinity towards ATG8 or GABARAP.

(A, B) Representative confocal images (inverted grayscale) for each condition from Figure 5 D, E are shown.

Fig. S11

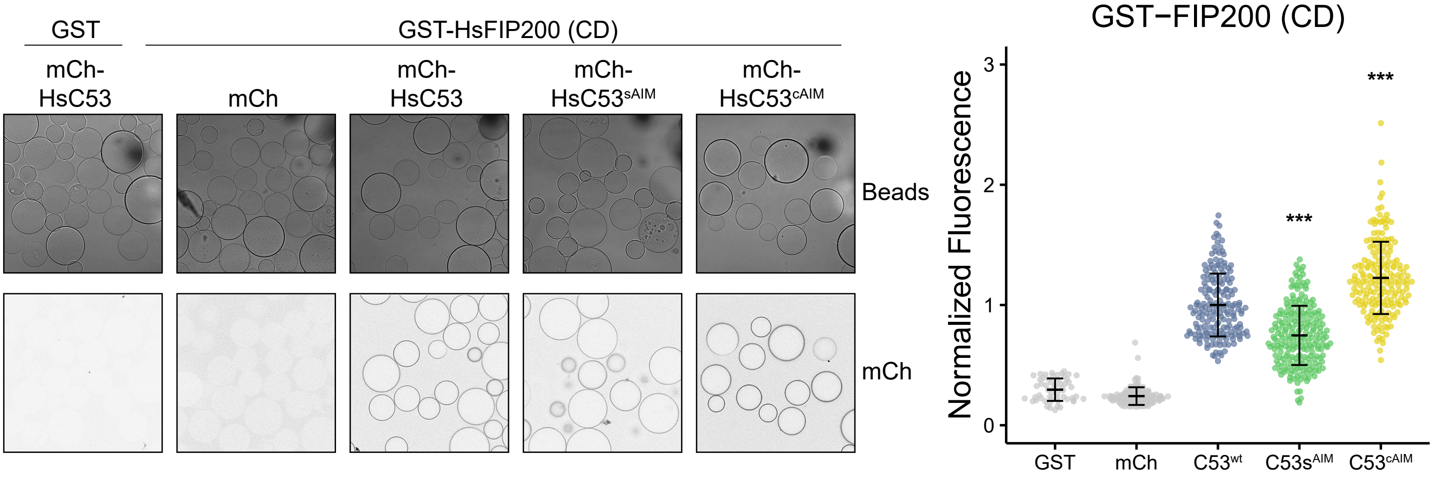
GST
GST-HsFIP200 (CD)
mCh-HsC53
mCh
mCh-HsC53
mCh-HsC53sAIM
mCh-HsC53cAIM
Beads
mCh
GST−FIP200 (CD)
Normalized Fluorescence
0
1
2
3
***
***
GST
mCh
C53wt
C53sAIM
C53cAIM

Appendix Fig. S11. C53-HsFIP200 Claw domain (CD) interaction is also mediated by the sAIM sequences and strengthened by sAIM to cAIM conversion.

Glutathione-sepharose beads were prepared by incubating them with GST-FIP200 CD. The pre-assembled beads were then washed and mixed with 1 μM of HsC53, 1 μM of $HsC53^{sAIM}$ or 1 μM of $HsC53^{cAIM}$ mutants. The beads were then imaged using a confocal microscope. *Left Panel,* representative confocal images (inverted grayscale) for each condition are shown. *Right panel*, normalized fluorescence is shown for each condition with the mean (± SD) of 2 independent replicates containing 2 technical replicates. Unpaired two-samples Wilcoxon test with continuity correction was performed to analyze the differences between wild type and mutants. ***, p-value < 0.001. Total number of beads, mean, median, standard deviation and p-values are reported in Supplementary data S1.

Fig. S12

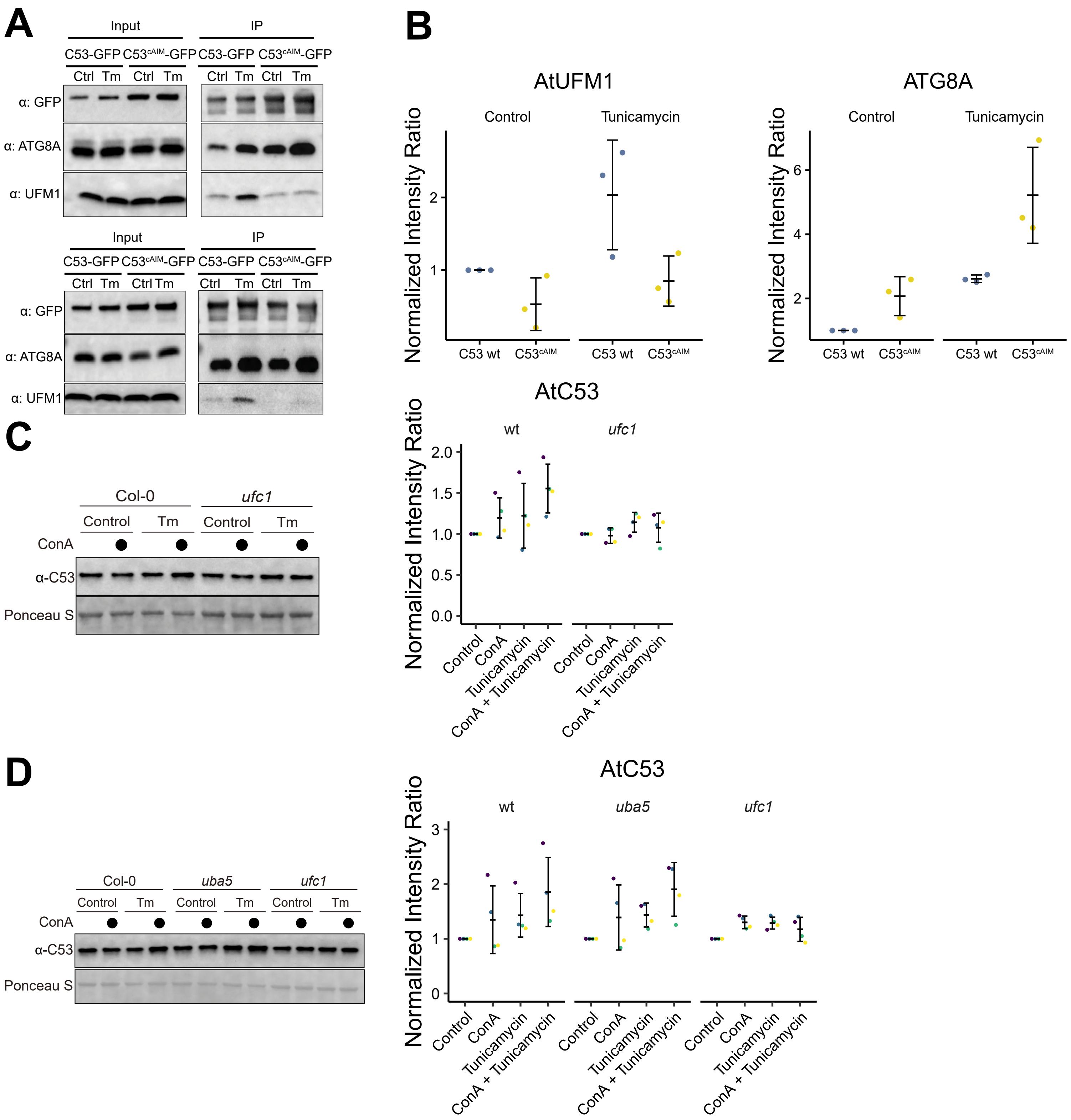
A
Input
IP
C53-GFP C53cAIM-GFP C53-GFP C53cAIM-GFP
Ctrl Tm Ctrl Tm Ctrl Tm Ctrl Tm
α: GFP
α: ATG8A
α: UFM1
B
AtUFM1
ATG8A
Control
Tunicamycin
Normalized Intensity Ratio
C53 wt
C53cAIM
AtC53
wt
ufc1
Control
ConA
Tunicamycin
ConA + Tunicamycin
C
Col-0
ufc1
Control
Tm
ConA
α-C53
Ponceau S
D
Col-0
uba5
ufc1
Control
Tm
ConA
α-C53
Ponceau S
AtC53
wt
uba5
ufc1
Normalized Intensity Ratio
Control
ConA
Tunicamycin
ConA + Tunicamycin

Appendix Figure S12. *In vivo* pull downs showing sAIM to cAIM conversion strengthens C53-ATG8 association while weakens C53-UFM1 association.

(A) Biological replicates of representative experiment shown in Figure 5F. 6-day old *Arabidopsis* seedlings expressing AtC53-GFP, AtC53^{cAIM}-GFP in *c53* mutant background were incubated in liquid 1/2 MS medium with 1% sucrose supplemented with DMSO as control (Ctrl) or 10 µg/ml tunicamycin (Tm) for 16 hours and used for co-immunoprecipitation. Lysates were incubated with GFP-Trap Magnetic Agarose, input and bound proteins were detected by immunoblotting using the respective antibodies as indicated. **(B) Quantification of blots in (Fig. 5F, Fig. S12A).** UFM1 and ATG8 protein levels that associate with AtC53-GFP or AtC53^{cAIM}-GFP are shown. Bars represent the mean (± SD) of 3 biological replicates (BR). **(C, D) Autophagic flux analysis of endogenous C53 protein using AtC53 antibody.** Western blot analysis of Col-0, *uba5* and *ufc1* Arabidopsis seedlings incubated in either control, or 10 µg/ml tunicamycin for 16 hrs. In addition, each treatment was supplemented with or without 1 µm concanamycin A (conA) as indicated to visualize vacuolar degradation. *Right panel*, quantification of the relative intensities of the protein bands were normalized for the total protein level of the lysate, relative protein intensity in the non-Tm and non-ConA treated control is set as 1 for each genotype. Average C53 levels and SD for n = 4 are shown.

Supplementary Data S1. Total number of beads, mean, median, standard deviation and p-values of the microscopy-based protein-protein interaction assays are reported.

Supplementary Data S2. Fiji macro and agarose bead model for automatic quantification.
