# Supplementary material for: Shuffled ATG8 interacting motifs form an ancestral bridge between UFMylation and autophagy
Source: EMBO J. 2023 Feb 10;42(10):e112053. doi: 10.15252/embj.2022112053 (PMC10183829; doi:10.15252/embj.2022112053)
Supplement: Supplementary file 2 — Expanded View Figures PDF [file EMBJ-42-e112053-s005.pdf]

Expanded View Figures

Figure EV1. Expanded version of the tree depicted in Fig 1A, displaying the presence and absence of UFMylation proteins across the eukaryotic taxa.

A–I The tree has been divided into eukaryotic supergroups including the Opisthokonta (A), Amoebozoa (B), Haptophyta and SAR (C), Archaeplastida (D), Discoba (E), Metamonada (F), CRuMs (G) and Apusozoa (H). (I) Pairwise binary Phi correlation of UFMylation proteins distributions.

 © 2023 The Authors

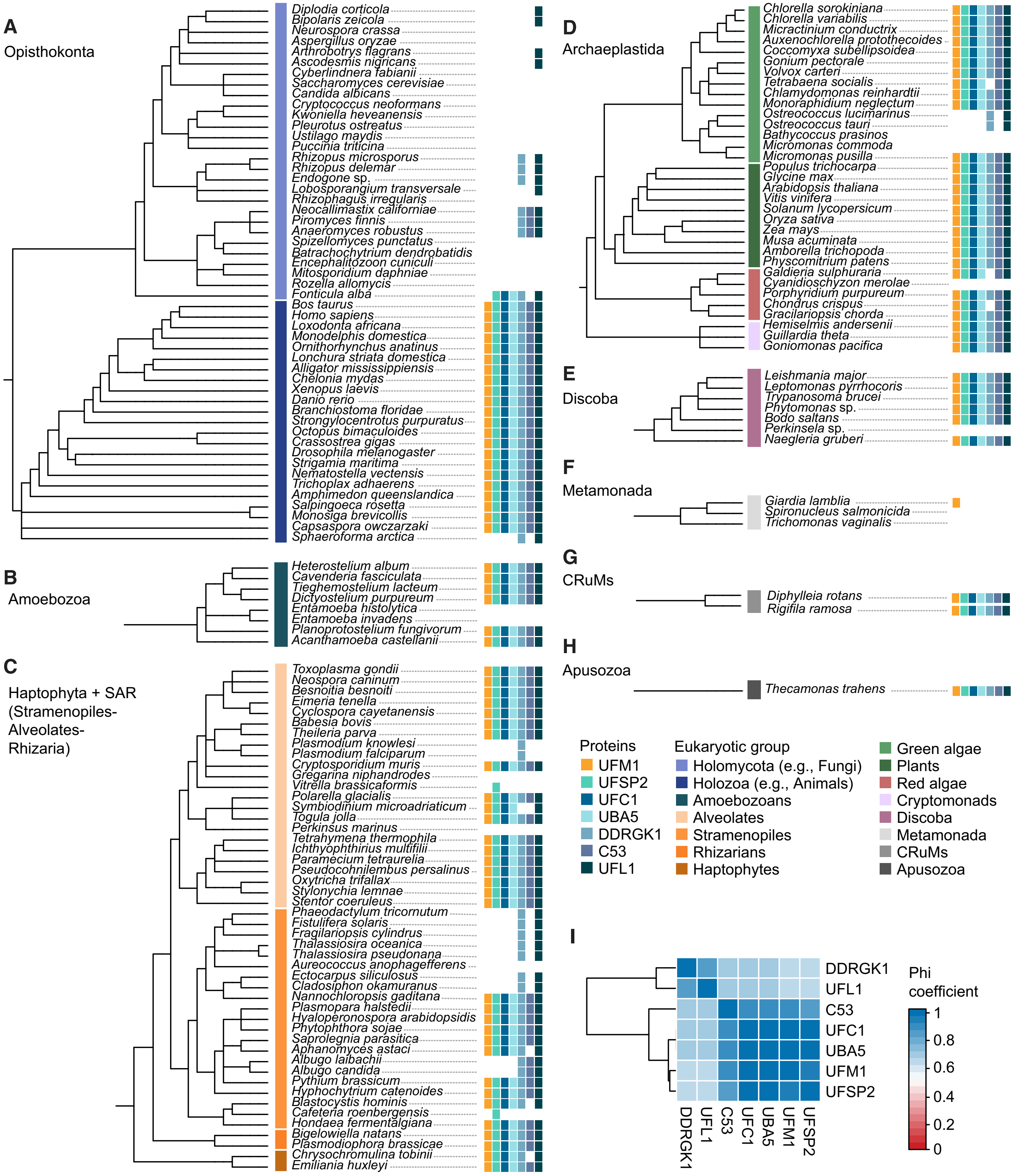

A
Opisthokonta
Diplodia corticola
Bipolaris zeicola
Neurospora crassa
Aspergillus oryzae
Arthrobotrys flagrans
Ascodesmis nigricans
Cyberlindnera fabianii
Saccharomyces cerevisiae
Candida albicans
Cryptococcus neoformans
Kwoniella heveanensis
Pleurotus ostreatus
Ustilago maydis
Puccinia triticina
Rhizopus microsporus
Rhizopus delemar
Endogone sp.
Lobosporangium transversale
Rhizophagus irregularis
Neocallimastix californiae
Piromyces finnis
Anaeromyces robustus
Spizellomyces punctatus
Batrachochytrium dendrobatidis
Encephalitozoon cuniculi
Mitosporidium daphniae
Rozella allomycis
Fonticula alba
Bos taurus
Homo sapiens
Loxodonta africana
Monodelphis domestica
Ornithorhynchus anatinus
Lonchura striata domestica
Alligator mississippiensis
Chelonia mydas
Xenopus laevis
Danio rerio
Branchiostoma floridae
Strongylocentrotus purpuratus
Octopus bimaculoides
Crassostrea gigas
Drosophila melanogaster
Strigamia maritima
Nematostella vectensis
Trichoplax adhaerens
Amphimedon queenslandica
Salpingoeca rosetta
Monosiga brevicollis
Capsaspora owczarzaki
Sphaeroforma arctica
B
Amoebozoa
Heterostelium album
Cavenderia fasciculata
Tieghemostelium lacteum
Dictyostelium purpureum
Entamoeba histolytica
Entamoeba invadens
Planoprotostelium fungivorum
Acanthamoeba castellanii
C
Haptophyta + SAR (Stramenopiles-Alveolates-Rhizaria)
Toxoplasma gondii
Neospora caninum
Besnoitia besnoiti
Eimeria tenella
Cyclospora cayetanensis
Babesia bovis
Theileria parva
Plasmodium knowlesi
Plasmodium falciparum
Cryptosporidium muris
Gregarina niphandrodes
Vitrella brassicaformis
Polarella glacialis
Symbiodinium microadriaticum
Togula jolla
Perkinsus marinus
Tetrahymena thermophila
Ichthyophthirius multifilii
Paramecium tetraurelia
Pseudocohnilembus persalinus
Oxytricha trifallax
Stylonychia lemnae
Stentor coeruleus
Phaeodactylum tricornutum
Fistulifera solaris
Fragilariopsis cylindrus
Thalassiosira oceanica
Thalassiosira pseudonana
Aureococcus anophagefferens
Ectocarpus siliculosus
Cladosiphon okamuranus
Nannochloropsis gaditana
Plasmopara halstedii
Hyaloperonospora arabidopsidis
Phytophthora sojae
Saprolegnia parasitica
Aphanomyces astaci
Albugo laibachii
Albugo candida
Pythium brassicum
Hyphochytrium catenoides
Blastocystis hominis
Cafeteria roenbergensis
Hondaea fermentalgiana
Bigelowiella natans
Plasmodiophora brassicae
Chrysochromulina tobinii
Emiliania huxleyi
D
Archaeplastida
Chlorella sorokiniana
Chlorella variabilis
Micractinium conductrix
Auxenochlorella protothecoides
Coccomyxa subellipsoidea
Gonium pectorale
Volvox carteri
Tetrabaena socialis
Chlamydomonas reinhardtii
Monoraphidium neglectum
Ostreococcus lucimarinus
Ostreococcus tauri
Bathycoccus prasinos
Micromonas commoda
Micromonas pusilla
Populus trichocarpa
Glycine max
Arabidopsis thaliana
Vitis vinifera
Solanum lycopersicum
Oryza sativa
Zea mays
Musa acuminata
Amborella trichopoda
Physcomitrium patens
Galdieria sulphuraria
Cyanidioschyzon merolae
Porphyridium purpureum
Chondrus crispus
Gracilariopsis chorda
Hemiselmis andersenii
Guillardia theta
Goniomonas pacifica
E
Discoba
Leishmania major
Leptomonas pyrrhocoris
Trypanosoma brucei
Phytomonas sp.
Bodo saltans
Perkinsela sp.
Naegleria gruberi
F
Metamonada
Giardia lamblia
Spironucleus salmonicida
Trichomonas vaginalis
G
CRuMs
Diphylleia rotans
Rigifila ramosa
H
Apusozoa
Thecamonas trahens
Proteins
UFM1
UFSP2
UFC1
UBA5
DDRGK1
C53
UFL1
Eukaryotic group
Holomycota (e.g., Fungi)
Holozoa (e.g., Animals)
Amoebozoans
Alveolates
Stramenopiles
Rhizarians
Haptophytes
Green algae
Plants
Red algae
Cryptomonads
Discoba
Metamonada
CRuMs
Apusozoa
I
DDRGK1
UFL1
C53
UFC1
UBA5
UFM1
UFSP2
Phi coefficient
1
0.8
0.6
0.4
0.2
0

Figure EV1.

Figure EV2. C53 orthologous from two TSAR representatives as well as representatives from Haptista, Cryptista, Diplomonada, Amoebozoa, and CRuMs interact with human and Arabidopsis ATG8 and UFM1.

Bacterial lysates containing recombinant protein were mixed and pulled down with glutathione magnetic agarose beads. Input and bound proteins were visualized by immunoblotting with anti-GST and anti-MBP antibodies. *Tetrahymena thermophila* (Tt), *Dictyostelium purpureum* (Dp), *Emiliania huxleyi* (Eh), *Guillardia theta* (Gt), *Trypanosoma brucei* (Tb), *Rigifila ramosa* (Rr) and *Phytophthora sojae* (Ps).

 © 2023 The Authors

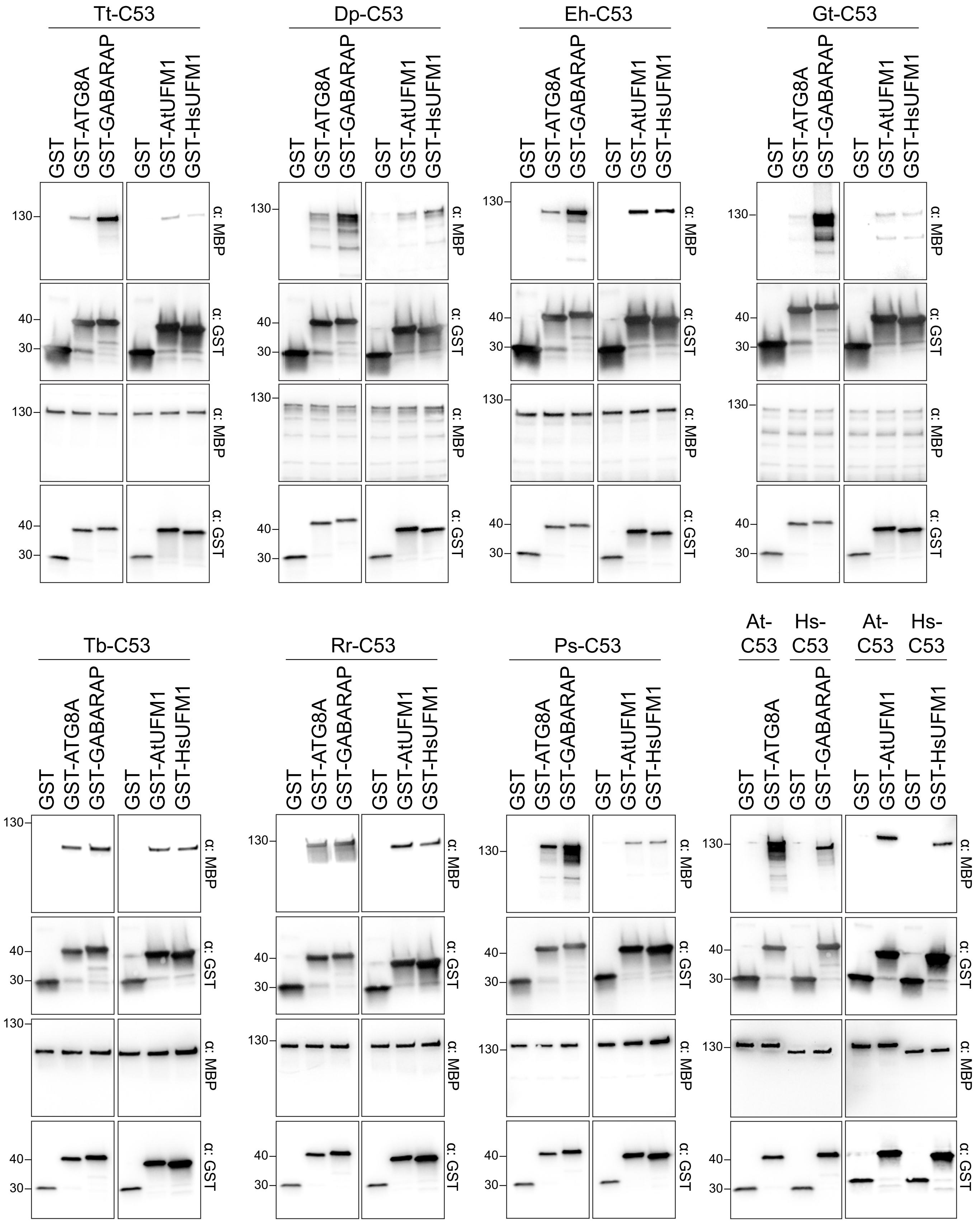
Tt-C53
Dp-C53
Eh-C53
Gt-C53
GST
GST-ATG8A
GST-GABARAP
GST
GST-AtUFM1
GST-HsUFM1
α: MBP
α: GST
α: MBP
α: GST
130
40
30
Tb-C53
Rr-C53
Ps-C53
At-C53
Hs-C53
At-C53
Hs-C53

Figure EV2.

Figure EV3. Structural characterization of UFM1 binding to C53 IDR using NMR spectroscopy.

A A small number of residues are affected by the addition of HsC53 IDR as shown in the HsUFM1 spectra. Overlaid ^{1}H-^{15}N HSQC spectra of isotope-labeled HsUFM1 (100 μM) in their free (gray) or bound state to 75 μM (blue), 100 μM (green), 200 μM (orange) and 300 μM (red) unlabeled HsC53 IDR. Insets of individual peaks that shift upon binding are shown.

B HsC53 IDR binding to HsUFM1 causes general signal intensity drop in HsUFM1 spectra. Intensity ratio broadening of HsUFM1 (100 μM) in the presence of 75 μM (blue), 100 μM (green), 200 μM (orange) and 300 μM (red) HsC53 IDR.

C Chemical shift perturbations (CSPs) in the HsUFM1 spectrum (gray) upon addition of 75 μM (blue), 100 μM (green), 200 μM (orange) and 300 μM (red) HsC53 IDR. The dashed line represents SD.

D Three-dimensional mapping of residues showing CSP in HsUFM1 NMR spectra upon HsC53 IDR binding. CSPs were mapped on the UFM1 structure (PDB: 1WXS) presented schematically on the left plot and as a surface representation in two projections on the right plot. Residues that are not affected or are slightly ($CSP < 0.025$), intermediately ($0.025 < CSP < 0.04$), or strongly ($CSP > 0.04$) affected by the binding are colored in tan, orange and red, respectively.

E AtC53 IDR binding to AtUFM1 is similar to that of AtUBA5 and involves sAIM1. Overlaid ^{1}H-^{15}N HSQC spectra of isotope-labeled AtUFM1 (100 μM) in their free (gray) or bound state to 100 μM unlabeled AtC53 IDR (red), 100 μM unlabeled AtC53 IDR^{W276A} (yellow) or 200 μM AtUBA5 LIR/UFIM (green). Insets of chemical shift perturbations of individual peaks are shown.

 © 2023 The Authors

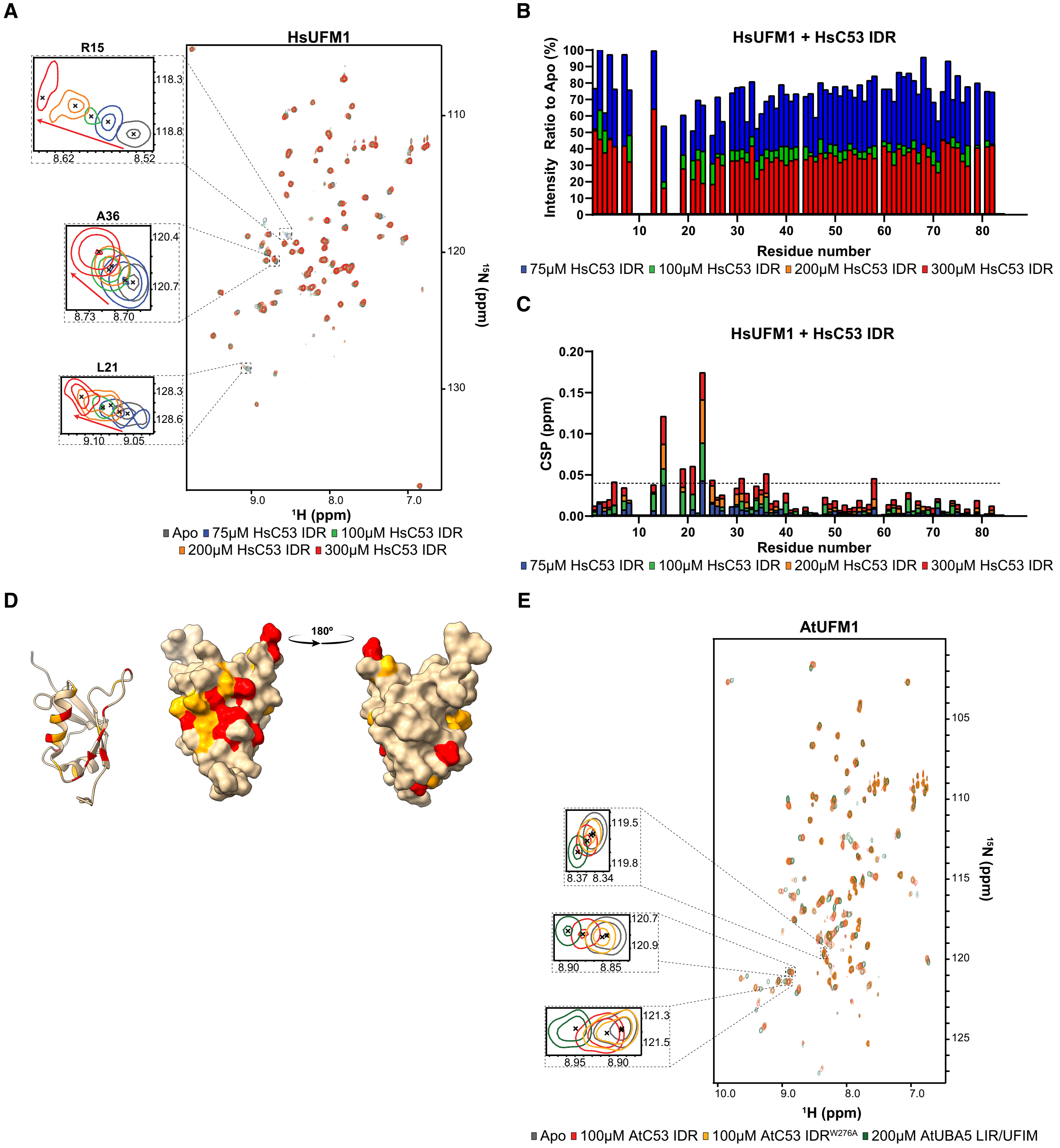
A
HsUFM1
R15
A36
L21
¹H (ppm)
¹⁵N (ppm)
Apo 75µM HsC53 IDR 100µM HsC53 IDR 200µM HsC53 IDR 300µM HsC53 IDR
B
HsUFM1 + HsC53 IDR
Intensity Ratio to Apo (%)
Residue number
75µM HsC53 IDR 100µM HsC53 IDR 200µM HsC53 IDR 300µM HsC53 IDR
C
HsUFM1 + HsC53 IDR
CSP (ppm)
Residue number
75µM HsC53 IDR 100µM HsC53 IDR 200µM HsC53 IDR 300µM HsC53 IDR
D
180°
E
AtUFM1
¹H (ppm)
¹⁵N (ppm)
Apo 100µM AtC53 IDR 100µM AtC53 IDR W276A 200µM AtUBA5 LIR/UFIM

Figure EV3.

Figure EV4. Structural characterization of ATG8 binding to C53 IDR using NMR spectroscopy.

A Addition of HsC53 IDR affects numerous residues in the GABARAP spectra. Overlaid ^1H-^{15}N HSQC spectra of isotope-labeled GABARAP (100 μM) in their free (gray) or bound state to 50 μM (blue), 100 μM (green) or 200 μM (orange) unlabeled HsC53 IDR. Insets of individual peaks that shifted upon binding are shown.

B HsC53 IDR binding to GABARAP causes a general signal intensity drop in GABARAP spectra. Intensity ratio broadening of GABARAP (100 μM) in the presence of 50 μM (blue), 100 μM (green) or 200 μM (orange) unlabeled HsC53 IDR. HN resonances for residues that could not be assigned in the bound state are shown as red asterisks.

C NMR chemical shift perturbations (CSP) of GABARAP in the presence of 50 μM (blue), 100 μM (green) or 200 μM (orange) HsC53 IDR. HN resonances for residues that could not be assigned in the bound state are shown as red asterisks. The dashed line represents S.D.

D Three-dimensional mapping of residues showing CSP in GABARAP NMR spectra upon HsC53 IDR binding. CSPs were mapped on the GABARAP structure (PDB: 6HB9) presented schematically on the left plot and as a surface representation in two projections on the right plot. Residues that are not affected or are slightly (CSP < 0.1), intermediately (0.1 < CSP < 0.2), or strongly (CSP > 0.2) affected by the binding are colored in tan, orange and red, respectively. The inset highlights the position of the HP0, HP1 and HP2 hydrophobic pockets in GABARAP.

E AtC53 IDR binding to ATG8 is similar to that of AtUBA5. Overlaid ^1H-^{15}N HSQC spectra of isotope-labeled ATG8A (100 μM) in their free (gray) or bound state to 100 μM unlabeled AtC53 IDR (red), 100 μM unlabeled AtC53 IDR^{W276A} (yellow) or 200 μM AtUBA5 LIR/UFIM (green). Insets of chemical shift perturbations of individual peaks are shown.

 © 2023 The Authors

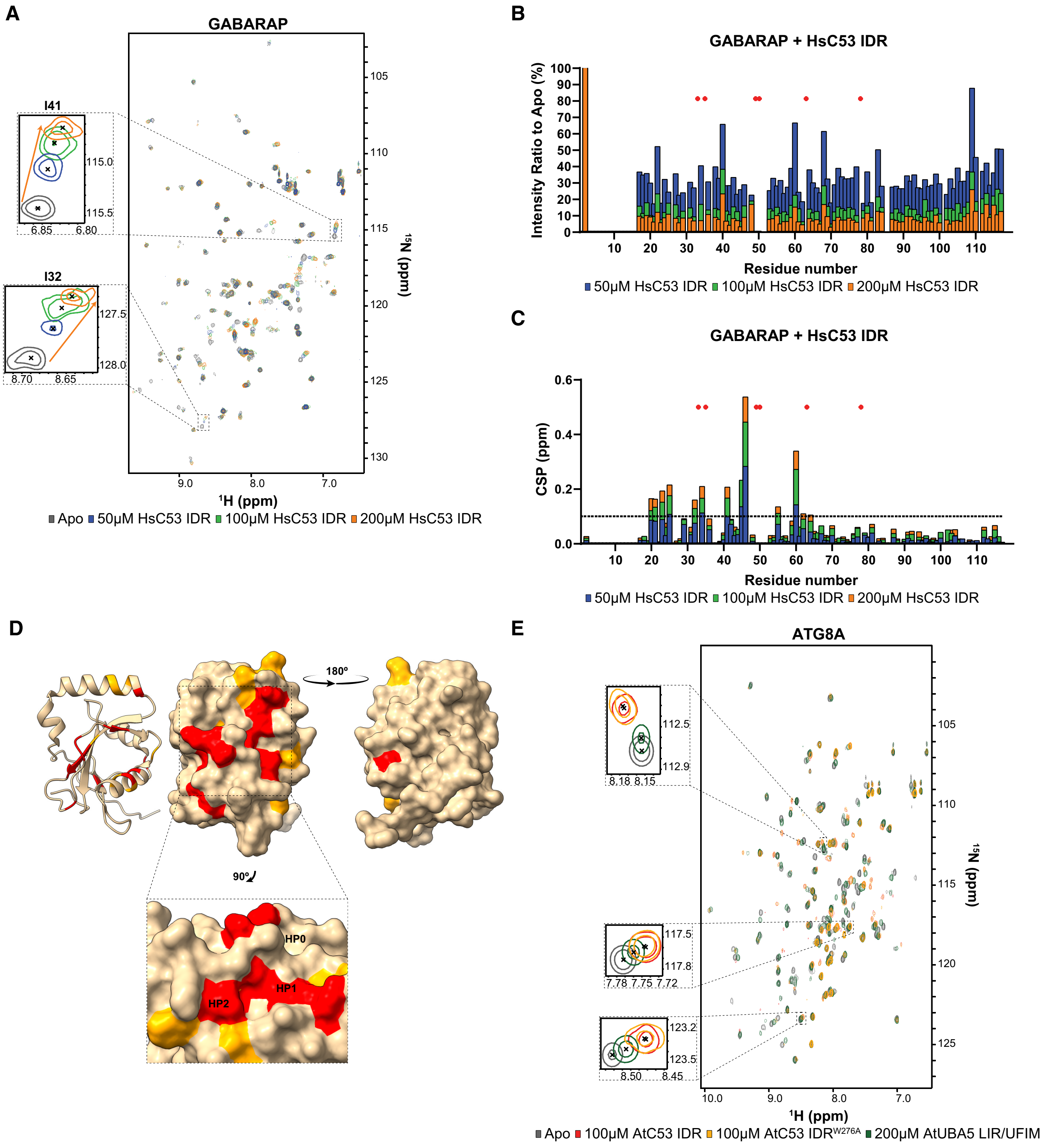
A
GABARAP
I41
I32
^{1}H (ppm)
^{15}N (ppm)
Apo 50μM HsC53 IDR 100μM HsC53 IDR 200μM HsC53 IDR
B
GABARAP + HsC53 IDR
Intensity Ratio to Apo (%)
Residue number
50μM HsC53 IDR 100μM HsC53 IDR 200μM HsC53 IDR
C
GABARAP + HsC53 IDR
CSP (ppm)
Residue number
50μM HsC53 IDR 100μM HsC53 IDR 200μM HsC53 IDR
D
180°
90°
HP0
HP1
HP2
E
ATG8A
^{1}H (ppm)
^{15}N (ppm)
Apo 100μM AtC53 IDR 100μM AtC53 IDR^{W276A} 200μM AtUBA5 LIR/UFIM

Figure EV4.

Figure EV5. HsC53^{cAIM} binds GABARAP with higher affinity when compared to HsC53^{wt}.

A Titrations of GABARAP with HsC53^{wt}. The concentrations of reactants are 40 μM (1, 2, 3, 4) or 70 μM (5) for GABARAP (in cell) and 250 μM (1, 2, 3, 4) or 310 μM HsC53^{wt} (in syringe) (5).

B Titrations of GABARAP with HsC53^{cAIM}. The concentrations of reactants are 40 μM (1, 2, 3, 4) or 70 μM (5) for GABARAP (in cell) and 250 μM (1, 2, 3, 4) or 360 μM HsC53^{cAIM} (in syringe) (5).

C Titrations of GABARAP with HsC53^{sAIM}. The concentrations of reactants are 40 μM for GABARAP (in cell) and 250 μM HsC53^{sAIM}. Global analysis was performed using a hetero-association model A + B. The top panels show the SVD reconstructed thermograms, the middle panel shows the isotherms, and the bottom panel shows the residuals. Extracted global parameters and their 68.3% confidence interval are reported in the respective tables. Thermograms were reconstructed with NITPIC, global analysis was done in SEDPHAT, and data visualization was plotted in GUSSI. The dissociation constant (K_D) is reported in μM units, while the enthalpy (ΔH) is reported in kcal/mol units.

© 2023 The Authors

A

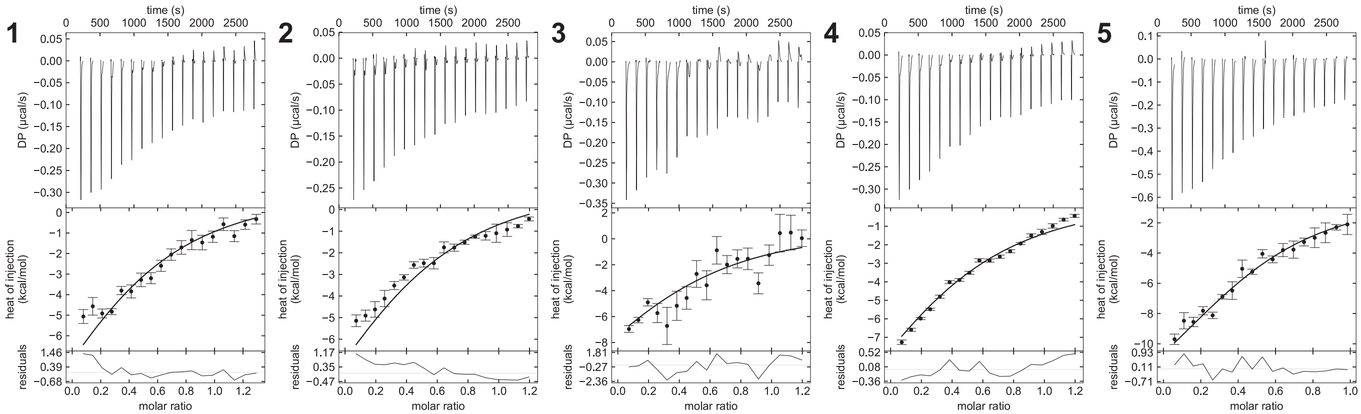

	Best-fit value	68.3% Confidence interval
K_D	20.86	8.35, 53.77
ΔH	-16.47	-37.07, -9.98

B

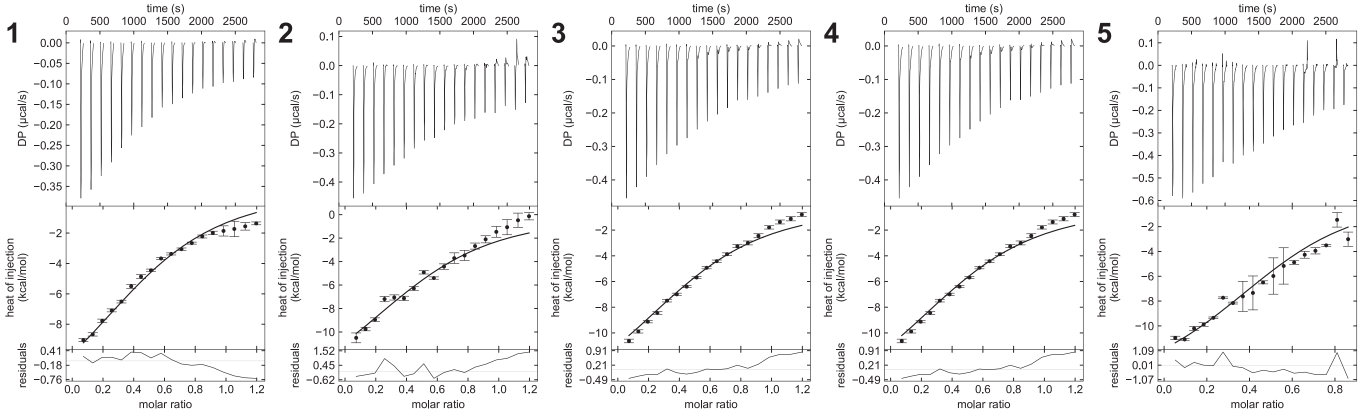

	Best-fit value	68.3% Confidence interval
K_D	11.24	5.91, 24.90
ΔH	-16.96	-25.18, -12.30

C

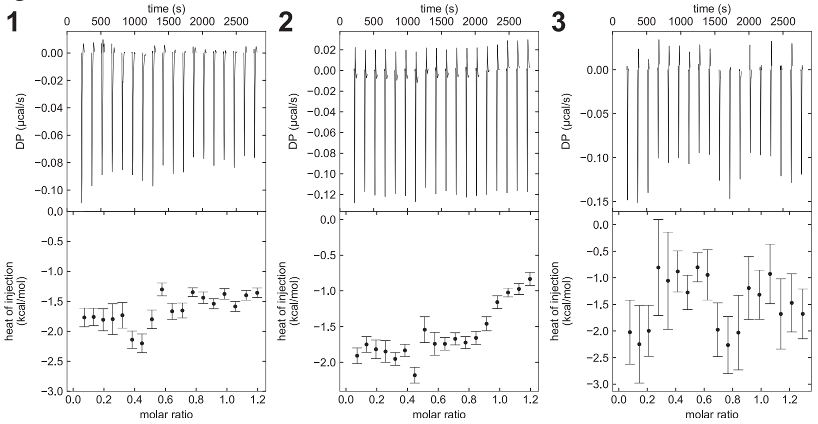

Figure EV5.
