# Supplementary material for: Shuffled ATG8 interacting motifs form an ancestral bridge between UFMylation and autophagy
Source: EMBO J. 2023 Feb 10;42(10):e112053. doi: 10.15252/embj.2022112053 (PMC10183829; doi:10.15252/embj.2022112053)
Supplement: Supplementary file 4 — PDF+ [file EMBJ-42-e112053-s004.pdf]

Article

TRANSPARENT PROCESS

OPEN ACCESS

THE EMBO JOURNAL

Shuffled ATG8 interacting motifs form an ancestral bridge between UFMylation and autophagy

Lorenzo Picchianti[1,2,†], Víctor Sánchez de Medina Hernández[1,2,†], Ni Zhan[1,†], Nicholas AT Irwin[3,4], Roan Groh[1,2], Madlen Stephani[1,2], Harald Hornegger[5], Rebecca Beveridge[6], Justyna Sawa-Makarska[5], Thomas Lendl[7], Nenad Grujic[1], Christin Naumann[8], Sascha Martens[5], Thomas A Richards[3], Tim Clausen[7], Silvia Ramundo[1], G Elif Karagöz[5,*] & Yasin Dagdas[1,**]

Abstract

UFMylation involves the covalent modification of substrate proteins with UFM1 (Ubiquitin-fold modifier 1) and is important for maintaining ER homeostasis. Stalled translation triggers the UFMylation of ER-bound ribosomes and activates C53-mediated autophagy to clear toxic polypeptides. C53 contains noncanonical shuffled ATG8-interacting motifs (sAIMs) that are essential for ATG8 interaction and autophagy initiation. However, the mechanistic basis of sAIM-mediated ATG8 interaction remains unknown. Here, we show that C53 and sAIMs are conserved across eukaryotes but secondarily lost in fungi and various algal lineages. Biochemical assays showed that the unicellular alga *Chlamydomonas reinhardtii* has a functional UFMylation pathway, refuting the assumption that UFMylation is linked to multicellularity. Comparative structural analyses revealed that both UFM1 and ATG8 bind sAIMs in C53, but in a distinct way. Conversion of sAIMs into canonical AIMs impaired binding of C53 to UFM1, while strengthening ATG8 binding. Increased ATG8 binding led to the autoactivation of the C53 pathway and sensitization of Arabidopsis thaliana to ER stress. Altogether, our findings reveal an ancestral role of sAIMs in UFMylation-dependent fine-tuning of C53-mediated autophagy activation.

Keywords ER-phagy; phylogenomics; ribosome stalling; selective autophagy; UFMylation

Subject Categories Autophagy & Cell Death; Plant Biology

DOI 10.15252/embj.2022112053 | Received 6 July 2022 | Revised 12 January 2023 | Accepted 18 January 2023 | Published online 10 February 2023

The EMBO Journal (2023) 42: e112053

Introduction

Perturbations in cellular homeostasis, termed "cellular stress," trigger protein aggregation and impair organelle function, reducing organismal fitness and lifespan. Quality control pathways closely monitor the health of cellular components to alleviate cellular stress (Pohl & Dikic, 2019). Cells first try to refold aberrant proteins and repair organelles to restore cellular homeostasis (Karagöz *et al*, 2019; Kirkin & Rogov, 2019; Sun & Brodsky, 2019). If these attempts fail, dysfunctional proteins and organelles are rapidly degraded (Dikic, 2017). Defects in cellular quality control have been linked to several diseases, including cognitive decline, aging, cancer, and metabolic disorders in humans, and reduced stress tolerance and fitness in plants (Marshall & Vierstra, 2018; Pohl & Dikic, 2019; Stephani & Dagdas, 2020; Klionsky *et al*, 2021). Although studies in the last decade have revealed a comprehensive suite of interconnected pathways that mediate protein and organelle degradation, the regulatory mechanisms that keep them switched off under normal conditions remain largely unknown.

Selective autophagy is a major quality control pathway that degrades excess or harmful cellular components, including protein aggregates or damaged organelles with high precision (Mizushima, 2018). Modular selective autophagy receptors (SARs) bring those cargo to the core autophagy machinery, resulting in their selective degradation (Dikic & Elazar, 2018; Marshall & Vierstra, 2018). SARs recruit the autophagy machinery through their interaction with ATG8, a ubiquitin-like protein conjugated to the phagophore, and ATG11/FIP200, a scaffold protein of the autophagy initiation complex ATG1/ULK1 (Kirkin, 2020). Recent structure–function studies have shown that SARs interact with ATG8 via various amino acid sequence motifs (Kirkin & Rogov, 2019). The **c**anonical **A**TG8-

1 Gregor Mendel Institute (GMI), Austrian Academy of Sciences, Vienna BioCenter (VBC), Vienna, Austria
2 Vienna BioCenter PhD Program, Doctoral School of the University of Vienna and Medical University of Vienna, Vienna, Austria
3 Department of Zoology, University of Oxford, Oxford, UK
4 Merton College, University of Oxford, Oxford, UK
5 Max Perutz Labs, Medical University of Vienna, Vienna BioCenter (VBC), Vienna, Austria
6 Department of Pure and Applied Chemistry, University of Strathclyde, Glasgow, UK
7 Research Institute of Molecular Pathology (IMP), Vienna BioCenter (VBC), Vienna, Austria
8 Department of Molecular Signal Processing, Leibniz Institute of Plant Biochemistry, Halle (Saale), Germany
*Corresponding author. Tel: +43 1 4277 61635; E-mail: guelsuen.karagoez@meduniwien.ac.at
**Corresponding author. Tel: +43 1 79044 9850; E-mail: yasin.dagdas@gmi.oeaw.ac.at
†These authors contributed equally to this work

© 2023 The Authors. Published under the terms of the CC BY 4.0 license.

Interacting **M**otif, (**cAIM**), also known as an **L**C3-**I**nteracting **R**egion (**LIR**), is a well-characterized short linear motif that interacts with ATG8 by forming a parallel β-sheet with β-sheet 2 in ATG8 (Birgisdottir *et al*, 2013). The cAIM is represented by the WXXL consensus sequence, where W is an aromatic residue (W/F/Y), L is an aliphatic hydrophobic residue (L/I/V), and X can be any residue (Johansen & Lamark, 2020). Recently, we showed that the ER-phagy receptor C53 (CDK5RAP3 in humans) interacts with plant and mammalian ATG8 isoforms via a noncanonical AIM sequence, with the consensus sequence IDWG/D, which we named the shuffled AIM (sAIM; Stephani *et al*, 2020). The structural basis of the sAIM-ATG8 interaction and its importance in C53-mediated autophagy and endoplasmic reticulum homeostasis are unclear.

Our work and a recent genome-wide CRISPR screen revealed that selective ER autophagy (ER-phagy) is regulated by UFMylation (Liang *et al*, 2020; Stephani *et al*, 2020). Further analysis of a UFMylated ER membrane protein NADH-cytochrome b5 reductase 3 (CYB5R3), also showed that C53 and the macroautophagy pathway are essential for the degradation of CYB5R3 via ER-phagy (Ishimura *et al*, 2022). UFMylation is similar to ubiquitination, where UFM1 is conjugated to substrate proteins via an enzymatic cascade (Daniel & Liebau, 2014; Banerjee *et al*, 2020). First, UFM1 is cleaved to its mature form by the protease UFSP2 and then activated by UBA5, an E1-activating enzyme. UBA5 transfers UFM1 to UFC1, the E2-conjugating enzyme, through a trans-binding mechanism (Oweis *et al*, 2016; Kumar *et al*, 2021). Finally, UFM1 is transferred to the substrate by UFL1, which, in complex with the ER membrane protein DDRGK1, forms an E3 ligase complex to covalently modify lysine residues on substrates (Peter *et al*, 2022; Zhou *et al*, 2022). To date, one of the best characterized UFMylation substrate is the 60S ribosomal subunit RPL26 (Walczak *et al*, 2019). RPL26 UFMylation is triggered by the stalling of ER-bound ribosomes and contributes to the degradation of incomplete polypeptides trapped on the ribosomes (Liang *et al*, 2020; Wang *et al*, 2020). We have shown that C53-mediated autophagy is also activated by ribosome stalling and contributes to ER-homeostasis (Stephani *et al*, 2020). However, how UFMylation regulates C53-mediated autophagy has yet to be determined.

Here, we combined evolutionary analyses with cellular and structural biology experiments to investigate the regulation of C53-mediated autophagy by UFMylation. We reconstructed the evolutionary history of the UFMylation pathway and C53 and found that it is ubiquitous across eukaryotes. Based on our phylogenetic analyses, we reconstituted the UFMylation machinery of the unicellular green algae, *Chlamydomonas reinhardtii*, and showed that it is functional and essential for the ER stress tolerance, demonstrating the importance of UFMylation beyond plants and animals. Biochemical and structural studies supported by evolutionary conservation analyses revealed that sAIMs within the intrinsically disordered region (IDR) of C53 form versatile binding sites that allow C53 to interact with both ubiquitin-like proteins (UBLs), UFM1 and ATG8. However, ATG8 and UFM1 bind these motifs with different specificities and affinities. While ATG8 bound strongest to a cAIM and displayed equal preference for the first and the second sAIM in the C53 IDR, UFM1 interacted preferentially with the first sAIM. Conversion of C53 sAIMs into canonical AIMs shifted C53 binding preference toward ATG8 and led to premature activation of autophagy, sensitizing *Arabidopsis thaliana* to ER stress. Altogether, our findings reveal an ancient UFM1-dependent regulatory mechanism that prevents premature activation of C53-mediated autophagy.

Results

The UFMylation pathway and C53 co-evolve across eukaryotes and are functional in the unicellular alga, *Chlamydomonas reinhardtii*

To explore the link between the UFMylation pathway and C53, we used a sensitive phylogenomic approach to search for the existence of proteins involved in C53-mediated autophagy in 153 eukaryotes from across the eukaryotic tree of life. Similar to previous studies, we identified the presence of UFMylation proteins in all major eukaryotic lineages, reaffirming that not only UFMylation pathway, but also C53, were likely present in the last eukaryotic common ancestor (Figs 1A and EV1A–H). However, although UFM1, C53, UFC1, and UFSP2 were exclusively observed in eukaryotes, UBA5 was identified in diverse Archaea and Bacteria and was likely derived from a bacterial homolog (Appendix Fig S1A). By contrast, UFL1 and DDRGK1 both appear to originate in Asgard archaea, the

Figure 1. UFMylation has a dynamic evolutionary history and functions to maintain ER homeostasis in the unicellular green alga, *Chlamydomonas reinhardtii*.

A A eukaryotic phylogeny displaying the presence or absence of UFMylation proteins across diverse species. Protein presence is displayed at the tip of each branch and major eukaryotic taxonomic groups are denoted with a colored ribbon. Losses of UFM1 have been highlighted. A schematic diagram depicting the UFMylation cascade and C53-receptor complex has been included for reference. See Fig EV1 for an expanded phylogeny, including species names.

B *Chlamydomonas reinhardtii* (Cr) UBA5 and UFC1 are active E1 and E2 enzymes. SDS–PAGE analysis showing transfer of UFM1 to CrUBA5 and CrUFC1. The gels are run in nonreducing conditions except where otherwise specified. The presented gel is representative of two independent experiments. ATP, Adenosine triphosphate; BME, β-mercaptoethanol.

C RPL26 mono- and di-UFMylation is lost in *Chlamydomonas reinhardtii* (Cr) *uba5* and *ufl1* mutants. Liquid TAP cultures were either left untreated (control) or treated for 24 h with 200 ng/ml tunicamycin. Protein extracts were analyzed by immunoblotting with anti-UFM1 antibodies. Total proteins were analyzed by Ponceau S staining. 12 h and 24 h treatment replicates are shown in Appendix Fig S3C. *Right Panel*, Quantification of UFMylated RPL26. RPL26 mono-UFMylated; RPL26-$(UFM1)_2$: RPL26 di-UFMylated.

D *Chlamydomonas reinhardtii* (Cr) UFMylation pathway mutants are sensitive to ER stress triggered by tunicamycin. Liquid TAP cultures of wild type (wt), *uba5*, *ufl1*, and *ire1* mutants were either left untreated (control) or treated for 3 days with 200 ng/ml of tunicamycin. *Left panel*, representative images of control and treated liquid cultures taken 3 days after incubation. *Middle Panel*, optical density (OD) 600 (OD_{600}) quantification of each genetic background under control conditions. Bars represent the mean (± SD) of 5 biological replicates. Two-tailed unpaired *t*-tests were performed to analyze the differences between wild type and mutants. *Right Panel*, normalized OD_{600} quantification of each genetic background under tunicamycin treatment conditions. Bars represent the mean (± SD) of 5 biological replicates. Two-tailed unpaired *t*-tests were performed to analyze the differences between wild type and mutants. ns P-value > 0.05; ***P-value < 0.001. BR, Biological Replicate.

© 2023 The Authors

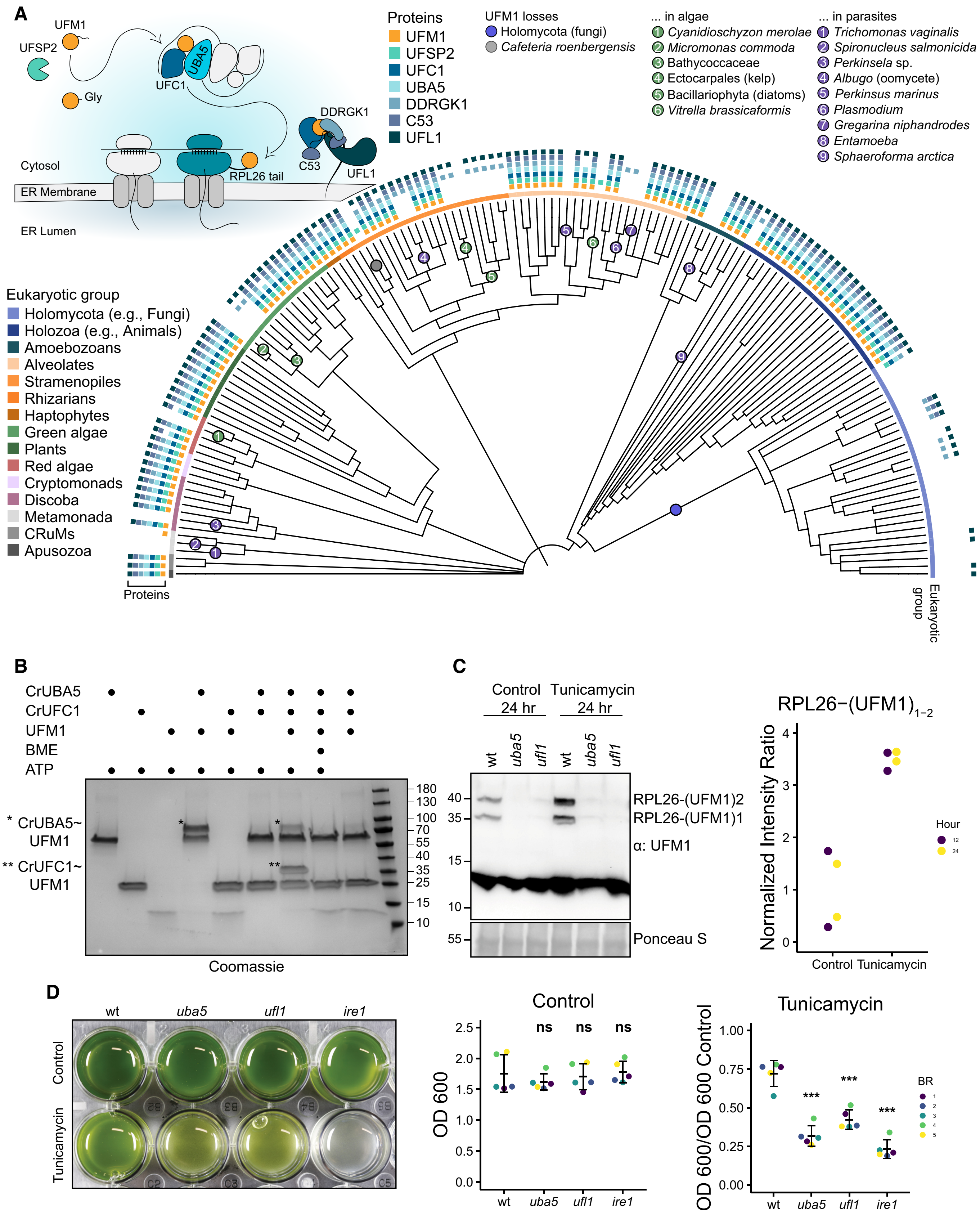
A
UFM1
UFSP2
Gly
UBA5
UFC1
DDRGK1
C53
UFL1
RPL26 tail
Cytosol
ER Membrane
ER Lumen
Proteins
UFM1
UFSP2
UFC1
UBA5
DDRGK1
C53
UFL1
UFM1 losses
Holomycota (fungi)
Cafeteria roenbergensis
... in algae
1 Cyanidioschyzon merolae
2 Micromonas commoda
3 Bathycoccaceae
4 Ectocarpales (kelp)
5 Bacillariophyta (diatoms)
6 Vitrella brassicaformis
... in parasites
1 Trichomonas vaginalis
2 Spironucleus salmonicida
3 Perkinsela sp.
4 Albugo (oomycete)
5 Perkinsus marinus
6 Plasmodium
7 Gregarina niphandrodes
8 Entamoeba
9 Sphaeroforma arctica
Eukaryotic group
Holomycota (e.g., Fungi)
Holozoa (e.g., Animals)
Amoebozoans
Alveolates
Stramenopiles
Rhizarians
Haptophytes
Green algae
Plants
Red algae
Cryptomonads
Discoba
Metamonada
CRuMs
Apusozoa
Proteins
Eukaryotic group
B
CrUBA5
CrUFC1
UFM1
BME
ATP
* CrUBA5~ UFM1
** CrUFC1~ UFM1
180
130
100
70
55
40
35
25
15
10
Coomassie
C
Control 24 hr
Tunicamycin 24 hr
wt
uba5
ufl1
wt
uba5
ufl1
40
35
15
10
55
RPL26-(UFM1)2
RPL26-(UFM1)1
α: UFM1
Ponceau S
RPL26−(UFM1)1−2
Normalized Intensity Ratio
Hour
12
24
Control
Tunicamycin
D
wt
uba5
ufl1
ire1
Control
Tunicamycin
Control
OD 600
ns
ns
ns
wt
uba5
ufl1
ire1
Tunicamycin
OD 600/OD 600 Control
***
***
***
BR
1
2
3
4
5

Figure 1.

closest prokaryotic relatives of eukaryotes (Zaremba-Niedzwiedzka *et al*, 2017), suggesting that they existed prior to the evolution of UFM1 and may have a UFM1-independent function (Appendix Fig S1B and C). This indicates that the UFMylation pathway has an ancient mosaic origin comprising proteins from both bacteria and Asgard archaea.

Despite its ancestral origins, multiple eukaryotic groups have lost some or all of the UFMylation proteins. Although the apparent absence of a gene can result from dataset incompleteness (e.g., incomplete genome assembly and annotation), recurrent absences across multiple closely related genomes imply gene loss. In this regard, we noted a loss of UFMylation in fungi as well as some alveolate parasites (*Plasmodium*, *Gregarina*, and *Perkinsus*) and algae (*Vitrella brassicaformis*), in accordance with previous observations (Fig 1A; Grau-Bove *et al*, 2015; Tsaban *et al*, 2021). However, we identified additional losses in numerous other parasitic and algal lineages. Gene loss in parasites is a recurrent phenomenon resulting from parasitic genome streamlining (Wolf & Koonin, 2013), but the absence of UFM1 in genera such as not only *Plasmodium*, but also *Entamoeba*, *Trichomonas*, *Spironucleus*, *Perkinsela*, and some oomycetes, could indicate that the pathway is expendable in parasitic organisms and suggests these species could be sensitive to drug-induced ER stress (Fig 1A). UFM1 has also been lost repeatedly in algal lineages such as diatoms, chlorophytes, and a red alga, implying that life history or other shared cellular characters may also dictate pathway retention. Similar to parasites and algae, fungi have also lost UFM1 and C53, although certain fungi retain UFMylation pathway components, indicating that either repeated losses have occurred or that genes were lost and subsequently reacquired through horizontal gene transfer (Fig 1A). As in these fungal taxa, some eukaryotes retain UFMylation pathway proteins despite the loss of UFM1. In particular, UFL1 and DDRGK1 are frequently retained and in a few cases, C53 (e.g., the oomycete genus *Albugo* and the chytrid class Neocallimastigomycetes; Fig 1A). This suggests that these proteins, especially the UFL1-DDRGK1 complex, may have additional cellular functions independent of UFMylation. These losses also revealed the strong correlation between C53 and the UFMylation pathway highlighting the co-evolution between these proteins (Fig EV1I). Altogether, these data demonstrate that C53 and the UFMylation pathway are present throughout eukaryotes but their retention may depend on the life history of individual species. Likewise, the co-evolution between C53 and UFM1 suggests a highly conserved functional link between C53 and UFM1.

Although UFM1 has been detected in unicellular species computationally, previous reports have suggested that the relationship between ER maintenance and UFMylation is specific to multicellular organisms (Grau-Bove *et al*, 2015; Walczak *et al*, 2019; Tsaban *et al*, 2021). Therefore, to interpret our evolutionary results, we first sought to assess the functionality of the UFMylation pathway in a unicellular species. To this end, we investigated UFMylation in *Chlamydomonas reinhardtii* (Cr), a single-celled green alga. First, we purified CrUBA5, CrUFC1, and CrUFM1 and tested their ability to conjugate UFM1 *in vitro*. E2-charging of CrUFM1 worked similar to the human UFMylation cascade (Oweis *et al*, 2016). UFM1 was transferred to UFC1 through the formation of a thioester bond, which could be reduced by β-mercaptoethanol, in a UBA5-dependent manner (Fig 1B). This indicates that the UFM1 conjugation mechanism is conserved in *C. reinhardtii*, prompting us to test substrate UFMylation *in vivo* using the classic UFMylation target, RPL26. We first examined the sequence conservation of the RPL26 tail across eukaryotes, which has been shown to be UFMylated at two C-terminal lysine residues (Walczak *et al*, 2019). Protein sequence alignment and Twincons analysis revealed that the UFMylated lysine residues in RPL26 are conserved in species with UFM1, including *C. reinhardtii*, but are more divergent in species lacking UFM1 (Appendix Fig S2A and B). Moreover, immunoblot analysis using a UFM1 antibody revealed two bands corresponding to mono- and di-ufmylated RPL26 (Fig 1C). RPL26 UFMylation was dependent on the UFMylation machinery, as both bands were absent in *uba5* and *ufl1* mutants (Fig 1C; Appendix Fig S3A–C). Finally, we performed mass spectrometry analysis to confirm the UFMylation of CrRPL26 (Appendix Fig S3D and E and Dataset EV1). Consistent with previous studies (Stephani *et al*, 2020; Wang *et al*, 2020), RPL26 UFMylation was also induced upon ER stress triggered by tunicamycin, a glycosylation inhibitor that leads to the accumulation of unfolded proteins in the ER (Fig 1C). Finally, we performed ER stress tolerance assays to test the physiological importance of UFMylation in *C. reinhardtii*. *uba5* and *ufl1* mutants were more sensitive to ER stress compared with the wild-type cells, confirming UFMylation is essential for ER stress tolerance in *C. reinhardtii* (Fig 1D). Altogether, these findings suggest that the contribution of UFMylation to ER homeostasis is not limited to plants and animals.

C53 interacts with UFM1 via the shuffled ATG8-interacting motifs (sAIMs)

The widespread role of UFMylation in regulating ER homeostasis and the strong co-association between C53 and UFM1 led us to investigate the relationship between these proteins using an evolutionary approach (Fig 1A). First, we performed ConSurf analysis to estimate the conservation of each residue (Ashkenazy *et al*, 2016). C53 has two α-helical domains at the N- and C- termini, connected with an intrinsically disordered region. In contrast to the alpha helical domains, which were highly conserved, the IDR was more divergent. However, within the IDR, there were four highly conserved regions that corresponded to the sAIMs, indicating that C53 sAIMs are conserved across diverse eukaryotes (Fig 2A). To explore a possible connection between UFM1 and the sAIMs, we examined the conservation of individual sAIMs between species with and without UFM1 using TwinCons (Fig 2B; Penev *et al*, 2021). Although IDR residues are generally not conserved between and within groups (TwinCons ≈ 0), the sAIMs show a strong dichotomy between species with and without UFM1, demonstrating a link between sAIM conservation and the presence of UFM1 (TwinCons score < 0). In agreement with this, a C53 multiple sequence alignment revealed that the IDRs in species lacking UFM1 are consistently shorter relative to UFM1-encoding species and lack sAIMs (Fig 2C). sAIMs were present in other species with the exception of *Toxoplasma gondii* and other apicomplexan parasites (Fig 2C). This indicates that, similar to *Plasmodium* and *Gregarina*, which have lost UFM1, other apicomplexans may have a reduced dependency on C53-mediated autophagy while still retaining UFM1 for other functions.

 © 2023 The Authors

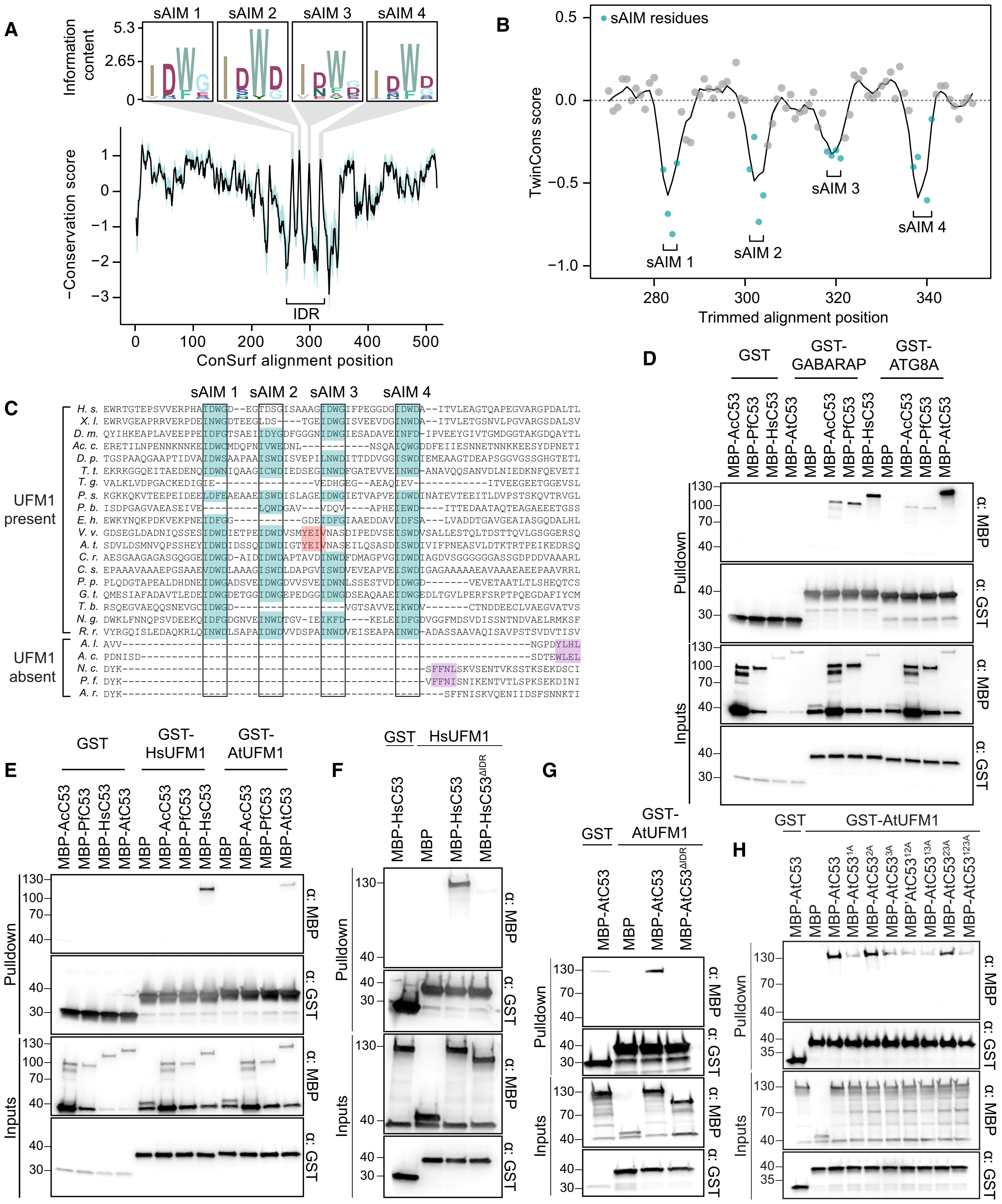
A
sAIM 1
sAIM 2
sAIM 3
sAIM 4
Information content
–Conservation score
IDR
ConSurf alignment position
B
sAIM residues
TwinCons score
Trimmed alignment position
C
UFM1 present
UFM1 absent
D
E
F
G
H
Pulldown
Inputs
GST
GST-GABARAP
GST-ATG8A
GST-HsUFM1
GST-AtUFM1
HsUFM1
α: MBP
α: GST

Figure 2.

Figure 2. sAIM sequences within C53 intrinsically disordered region (IDR) are highly conserved and essential for UFM1 interaction.

A ConSurf conservation analysis of C53 from diverse eukaryotes. Conserved regions within the IDR (intrinsically disordered region) have been highlighted and supplemented with sequence logos.

B TwinCons analysis comparing the conservation and divergence of C53 among species with and without UFM1. The four regions corresponding to the sAIMs have been highlighted. Negative values reflect divergent signature regions between the two species groups.

C A trimmed multiple sequence alignment depicting the conservation of the sAIMs. The four sAIMs and cAIMs in plants and UFM1-lacking species have been highlighted in teal and light red, respectively. Putative cAIMs are highlighted in purple. Abbreviations: *H. s., Homo sapiens*; *X. l., Xenopus laevis*; *D. m., Drosophila melanogaster*; *Ac. c., Acanthamoeba castellanii*; *D. p., Dictyostelium purpurea*; *T. t., Tetrahymena thermophila*; *T. g., Toxoplasma gondii*; *P. s. Phytopthora sojae*; *P. b., Plasmodiophora brassicae*; *E. h., Emiliania huxleyi*; *V. v., Vitis vinifera*; *A. t., Arabidopsis thaliana*; *C. r., Chlamydomonas reinhardtii*; *C. s., Chlorella sorokiniana*; *P. p, Porphyridium purpureum*; *G. t., Guillardia theta*; *T. b., Trypanosoma brucei*, *N. g., Naegleria gruberi*; *R. r., Rigifila ramose*; *A. l., Albugo laibachii*; *A. c., Albugo candida*; *N. c., Neocallimastix californiae*; *P. f., Piromyces finnis*; *A. r., Anaeromyces robustus*.

D, E AcC53 and PfC53 do not have sAIM sequences and cannot interact with UFM1. Ac, *Albugo candida*, Pf, *Piromyces finnis*.

F, G C53 IDR is essential for UFM1 interaction. HsC53 (B) and AtC53 (C) IDRs are necessary to mediate the interaction with AtUFM1 and HsUFM1, respectively. MBP-AtC53$^{\Delta IDR}$: MBP-AtC53$^{(1–239,\ (KGSGSTSGSG)2,\ 373–549)}$; MBP-HsC53$^{\Delta IDR}$: HsC53$^{(1–262,\ (KGSGSTSGSG),\ 317–506)}$.

H AtC53^{sAIM} cannot interact with AtUFM1. Individual or combinatorial mutations in sAIM1 (1A: W276A), sAIM2 (2A: W287A), and sAIM3 (3A: W335A) suggest sAIM1 is crucial for UFM1 interaction. (B, C, E, F, G) Bacterial lysates containing recombinant protein were mixed and pulled down with glutathione magnetic agarose beads. Input and bound proteins were visualized by immunoblotting with anti-GST and anti-MBP antibodies.

The correlation between UFM1 and the C53 sAIMs suggested a functional interaction. To test this, we synthesized C53 homologs from two species that lack UFM1, the oomycete *Albugo candida* (Ac) and the chytrid *Piromyces finnis* (Pf), and we tested whether they interact with UFM1 or ATG8 using *in vitro* pulldown assays. Both AcC53 and PfC53 were able to interact with Arabidopsis ATG8A and human ATG8 isoform GABARAP (Fig 2D), but they did not interact with either of the UFM1 orthologs tested (Fig 2E). Their ability to bind ATG8 may be due to the presence of putative cAIMs within the truncated IDRs of both AcC53 and PfC53 (Fig 2C and D). By contrast, C53 homologs from other species that represent major eukaryotic lineages, including *Tetrahymena thermophila* (Tt), *Dictyostelium purpureum* (Dp), *Emiliania huxleyi* (Eh), *Guillardia theta* (Gt), *Trypanosoma brucei* (Tb), *Rigifila ramosa* (Rr), and *Phytophthora sojae* (Ps) interacted with both UFM1 and ATG8 (Fig EV2). In conclusion, the sAIMs are likely retained for binding both UFM1 and ATG8.

Given the ability of C53 sAIMs to bind both UFM1 and ATG8, we sought to characterize the human UFM1-C53 complex using native mass spectrometry (nMS). We found that C53 binds to human UFM1 in a 1:1 or 1:2 stoichiometry, similar to the C53-GABARAP interaction (Appendix Fig S4). To map the UFM1-interacting region in C53, we performed *in vitro* pulldowns with *Homo sapiens* (Hs) and *Arabidopsis thaliana* (At) C53 truncations. As in the C53-ATG8 interaction, C53 IDR was necessary for the interaction between C53 and UFM1 (Fig 2F and G). Further individual and combinatorial mutagenesis of the tryptophan residues in sAIMs showed that the UFM1-C53 interaction is mediated by sAIMs located in the IDR (Fig 2H).

We next asked whether ATG8 and UFM1 bind the sAIMs in a similar manner. First, we performed nMS analysis to test the interaction of HsUFM1 with a canonical AIM (cAIM) peptide (Stephani *et al*, 2020). Unlike the UBA5-LIR peptide (GPLHDDNEWNISVVDD), which has been shown to interact with UFM1 (Habisov *et al*, 2016; Padala *et al*, 2017; Huber *et al*, 2020), the cAIM peptide did not appreciably interact with UFM1 (Fig 3A). Consistently, the cAIM peptide outcompeted the GABARAP-C53 interaction but not the HsUFM1-C53 interaction (Fig 3B). *C. reinhardtii* proteins behaved similarly; CrC53 interacted with ATG8 in a cAIM-dependent manner and CrUFM1 in a cAIM-independent manner (Appendix Fig S5A and B).

To quantitatively analyze the interaction between C53 and UFM1, we first used Isothermal titration calorimetry (ITC). Unfortunately, we did not detect appreciable binding between UFM1 and C53, likely due to the transient nature of the interaction (Appendix Fig S6A and B). We next performed microscopy-based on-bead binding assays to compare the mode of binding of UFM1-C53 to that of UFM1-UBA5 LIR interaction. The advantage of this technique is the ability to visualize protein–protein interactions with fast dissociation constants at equilibrium. Therefore, it is more suited to detect relatively weak, transient interactions (Turco *et al*, 2019). We purified GST-tagged Arabidopsis and human

Figure 3. Canonical ATG8-interacting motif (cAIM) cannot outcompete C53-UFM1 interaction.

A Complex formation between cAIM peptide and UFM1. Native mass spectrometry (nMS) spectra of (1) HsUFM1 (5 μM), (2) HsUFM1 (5 μM) and UBA5 LIR peptide (25 μM) and (3) HsUFM1 (5 μM) and cAIM peptide (25 μM). UFM1 forms a 1:1 complex with the UBA5 LIR peptide. Only a negligible amount of 1:1 complex is formed between the cAIM peptide and UFM1, indicating a lower affinity interaction.

B The cAIM peptide cannot outcompete HsUFM1-HsC53 interaction. Bacterial lysates containing recombinant protein were mixed and pulled down with glutathione magnetic agarose beads. Input and bound proteins were visualized by immunoblotting with anti-GST and anti-MBP antibodies. cAIM peptide was used to a final concentration of 200 μM. HsC53^{sAIM}: HsC53$^{W269A,\ W294A,\ W312A}$.

C, D Microscopy-based protein–protein interaction assays showing unlike GABARAP-C53 interaction, UFM1-C53 interaction is insensitive to cAIM peptide competition. Glutathione-sepharose beads were prepared by incubating them with GST-GABARAP (C) or GST-HsUFM1 (D). The preassembled beads were then washed and mixed with 1 μM of HsC53 containing increasing concentrations of cAIM peptide (0–100 μM). The beads were then imaged using a confocal microscope. *Left Panel*, representative confocal images (inverted grayscale) for each condition are shown. *Right panel*, normalized fluorescence is shown for each condition with the mean (± SD) of 4 replicates. Unpaired two-samples Wilcoxon test with continuity correction was performed to analyze the differences between wild type and wild type with 100 μM AIM peptide. ns, not significant, P-value > 0.05, ***P-value < 0.001. Total number of beads, mean, median, standard deviation and P-values are reported in Appendix Data S1.

 © 2023 The Authors

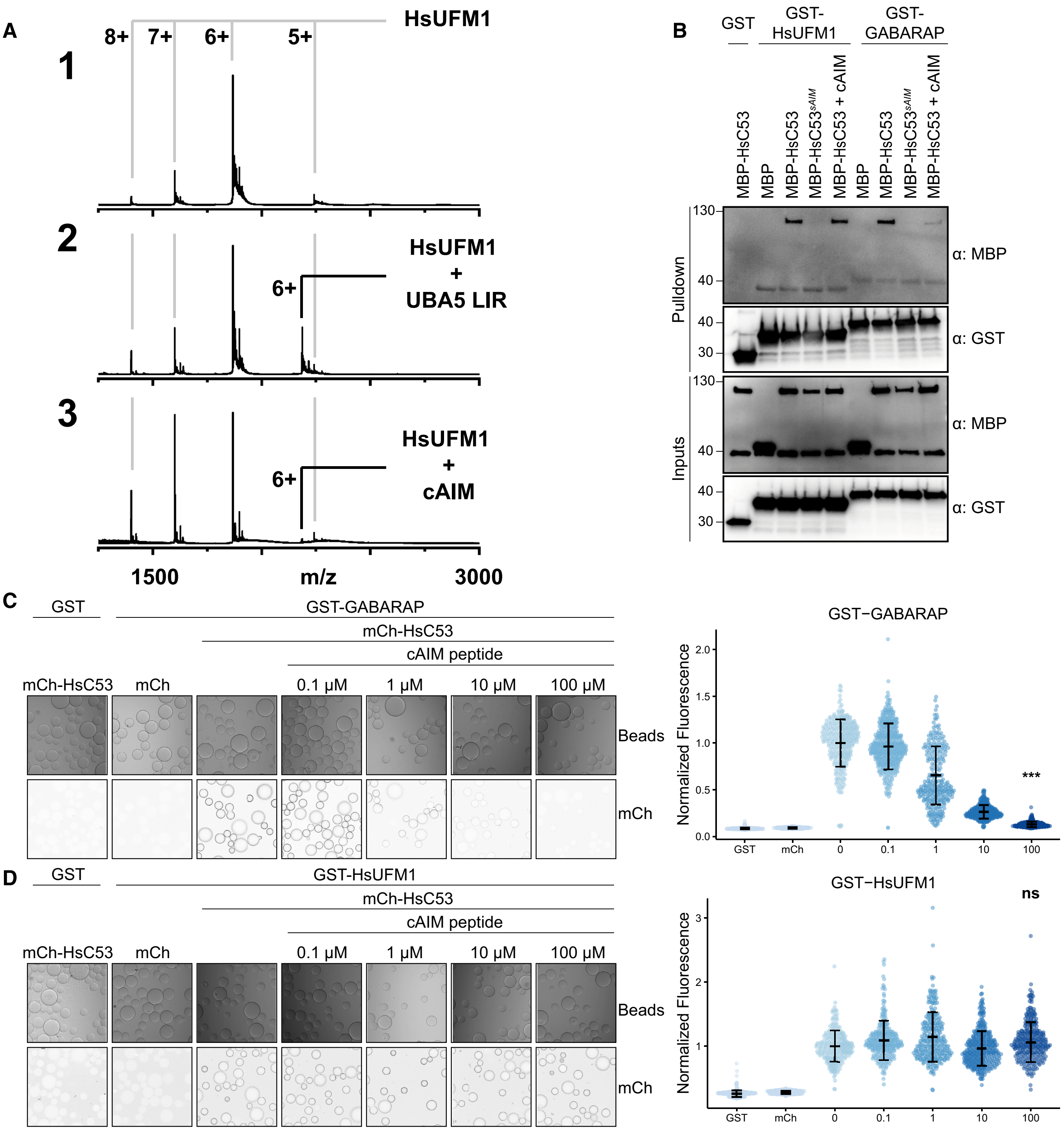

Figure 3.

ATG8 and UFM1 proteins and coupled them to the glutathione-coated beads (Sepharose 4B, Cytiva). We then tested whether mCherry-tagged Arabidopsis and human C53 proteins could bind to the ATG8 or UFM1 coupled beads (Appendix Fig S7A). Both Arabidopsis and human C53 interacted with wild-type ATG8 and UFM1. HsC53-GABARAP and AtC53-ATG8A interaction was outcompeted with increased concentrations of the cAIM peptide (Fig 3C; Appendix Fig S7B). By contrast, the cAIM peptide could not outcompete the HsC53-HsUFM1 or AtC53-AtUFM1 interaction (Fig 3D; Appendix Fig S7C). On the contrary, the UBA5-LIR peptide and GABARAP were able to disrupt C53-UFM1 interaction (Appendix Fig S7D). Altogether, these results suggested that ATG8 and UFM1 bind the sAIMs within C53 IDR, albeit in a different manner.

© 2023 The Authors

Comparative structural analysis revealed the differences between C53 IDR-UFM1 and C53 IDR-ATG8 interaction

To elucidate the difference between UFM1 and ATG8 binding to C53 IDR, we performed comparative nuclear magnetic resonance (NMR) spectroscopy analysis. We first obtained backbone resonance assignments of AtC53 IDR using three-dimensional sequential assignment strategies. We could assign 89% of the residues in AtC53 IDR. Since the sAIMs in AtC53 IDR are highly similar in sequence, we validated our assignments using sAIM1 (AtC53 IDR^{W276A}) and sAIM2 (AtC53 IDR^{W287A}) mutants (Fig 4A; Appendix Fig S8A). The 2D heteronuclear single quantum correlation (HSQC) spectrum of ^{15}N-labeled AtC53 IDR displayed narrow dispersion of the backbone amide residues, validating its intrinsically disordered nature.

The NMR signals are sensitive to their chemical environment; binding of an interaction partner or conformational changes induced by protein–protein interaction shifts the NMR spectra. Moreover, NMR signal intensity drops mainly due to an increase in molecular weight upon complex formation and the chemical exchange that happens at the interaction surface (Cox *et al*, 1993; Sette *et al*, 1999; Zuiderweg, 2002; Wüthrich, 2003). Using the assignment of the backbone residues, we next mapped UFM1 and ATG8 interaction sites in AtC53 IDR by acquiring 2D HSQC spectra of ^{15}N-labeled AtC53 IDR in the presence and absence of unlabeled AtUFM1 or ATG8A. Upon AtUFM1 binding, the signals of AtC53 IDR displayed both reduction in their intensity (Fig 4B) and chemical shift perturbations (CSP; Fig 4C). CSP analysis showed that upon AtUFM1 binding, the signals corresponding to Asp275, (sAIM1), Asp286 and Asp288 (sAIM2) and Thr279, Glu281, and Glu285 that are located between sAIM1 and sAIM2 shifted in a concentration-dependent manner (Fig 4B–D; Appendix Fig S8B). Instead, the signals corresponding to Ile274 and Trp276 found in sAIM1, Ile278, and Val280 found in the region between sAIM1 and sAIM2 and Trp287 located in sAIM2 exhibited line broadening and reduced intensity upon AtUFM1 binding (Fig 4C and D; Appendix Fig S8C). Importantly, the signals derived from sAIM3 only slightly shifted (Fig 4C and D). These results are in line with the pulldown assays performed with the Trp to Ala mutants of the sAIMs (Fig 2H), confirming that sAIM1 and sAIM2 regions are the major interaction sites for AtUFM1. Moreover, NMR analyses also revealed that the hydrophobic residues between the two sAIMs contributed to the binding. The sAIM1 region showed a significant decrease in signal intensity already at the lowest UFM1 concentration, confirming sAIM1 is the highest affinity binding site for UFM1, followed by sAIM2 region (Fig 4B–D; Appendix Fig S8D). As sAIM1 and sAIM2 share the same sequence motif (IDWD), these results indicated that the hydrophobic residues following sAIM1 contribute to C53 IDR-UFM1 interaction and result in the preferential binding of UFM1 to sAIM1. To confirm these data, we performed Fluorescence anisotropy experiments with AtC53 IDR-derived peptides harboring sAIM or cAIM. We found that UFM1 bound to a peptide that covers both sAIM1 and sAIM2 (sAIM1,2) with an affinity of 148.2 μM (Appendix Fig S8E). This affinity is lower than the affinity predicted from the CSP data as the NMR signals corresponding to sAIM1 already disappeared upon the addition of 75 μM UFM1. UFM1 bound to the peptides derived from individual sAIMs with lower affinity and the binding did not reach saturation even at 320 μM UFM1 concentration (Appendix Fig S8E), underlining that additional contacts provided by the sAIM2 increases its affinity for UFM1 and the interaction might be further stabilized in the context of the complete IDR region of AtC53.

We next characterized the binding of ATG8A to AtC53 IDR. Upon ATG8A binding, a large number of signals in the AtC53 IDR spectrum disappeared or shifted (Fig 4E and F; Appendix Fig S9A). The signals of the cAIM and its neighboring residues covering Leu301 to Glu314 disappeared or shifted at the lowest ATG8A concentration (75 μM), followed by sAIM1 and sAIM2 regions as we titrated AtC53 IDR with increased concentrations of ATG8A (Fig 4E–G; Appendix Fig S9B and C). Importantly, the signals corresponding to Ile274 and Trp276 in sAIM1, which disappeared upon 75 μM UFM1 titration, only disappeared upon 200 μM ATG8A addition, suggesting that while the most preferred binding site for UFM1 is sAIM1, it is cAIM for ATG8A. Similar to UFM1, CSP analysis showed that the signals in sAIM3 region only shifted at the highest titrated ATG8A concentration (300 μM) and did not show significant signal intensity reduction, suggesting sAIM3 is a low-affinity binding site for both ATG8A and UFM1 (Fig 4F and G; Appendix Fig S9B). These results were confirmed by the Fluorescence anisotropy

Figure 4. Comparative nuclear magnetic resonance (NMR) spectroscopy analyses show C53 IDR-UFM1 interaction is different than C53 IDR-ATG8 interaction. ▶

A AtC53 IDR harbors highly conserved canonical and shuffled ATG8 interaction motifs. Schematic representation of AtC53 domains with the primary sequence of C53 IDR. The AIM sequences and amino acid Consurf conservation are indicated with rectangular boxes and a color code, respectively.

B Binding of AtUFM1 to AtC53 IDR leads to a general drop in signal intensity. NMR signal intensity of AtC53 IDR (100 μM) in the presence of 300 μM AtUFM1 normalized to signal intensity of AtC53 IDR alone. Bars corresponding to residues in the AIMs are highlighted.

C UFM1-IDR binding involves sAIM1 and sAIM2. NMR chemical shift perturbations (CSP) of AtC53 IDR (100 μM) in the presence of 75 μM (blue), 100 μM (green), 200 μM (orange) and 300 μM (red) AtUFM1.

D The backbone amide signals of AtC53 IDR shift upon AtUFM1 addition in a concentration-dependent manner. Insets of overlaid ^{1}H-^{15}N HSQC spectra of isotope-labeled AtC53 IDR (100 μM) showing chemical shift perturbations of individual peaks from backbone amides of AIM residues in their free (gray) or bound state to unlabeled AtUFM1. Chemical shifts are indicated with arrows.

E Binding of ATG8A to AtC53 IDR leads to a localized signal intensity drop in sAIM1-2 and cAIM regions. NMR signal intensity of AtC53 IDR (100 μM) in the presence of 300 μM ATG8A is normalized to signal intensity of AtC53 IDR alone. Bars corresponding to residues in AIMs are highlighted. The intensity levels are capped at 100%. See Appendix Fig S9B for the full plot.

F ATG8A-IDR binding involves sAIM1-2 and the cAIM regions. NMR CSP of AtC53 IDR (100 μM) in the presence of 75 μM (blue), 100 μM (green), 200 μM (orange) and 300 μM (red) ATG8A.

G AtC53 IDR spectra signals in the binding sites shift and broadened upon ATG8 addition. Insets of overlaid ^{1}H-^{15}N HSQC spectra of isotope-labeled AtC53 IDR (100 μM) showing chemical shift perturbations of individual peaks from backbone amides of AIM residues in their free (gray) or bound state to unlabeled ATG8A. Unassigned AtC53 IDR residues are indicated by hashtags and HN resonances for residues that could not be assigned in the bound state are shown as gray bars (showing intensity signals of neighbor signals). Chemical shifts are indicated with arrows. Titrations with different concentrations of the ligands are colored similarly to (C and F).

 © 2023 The Authors

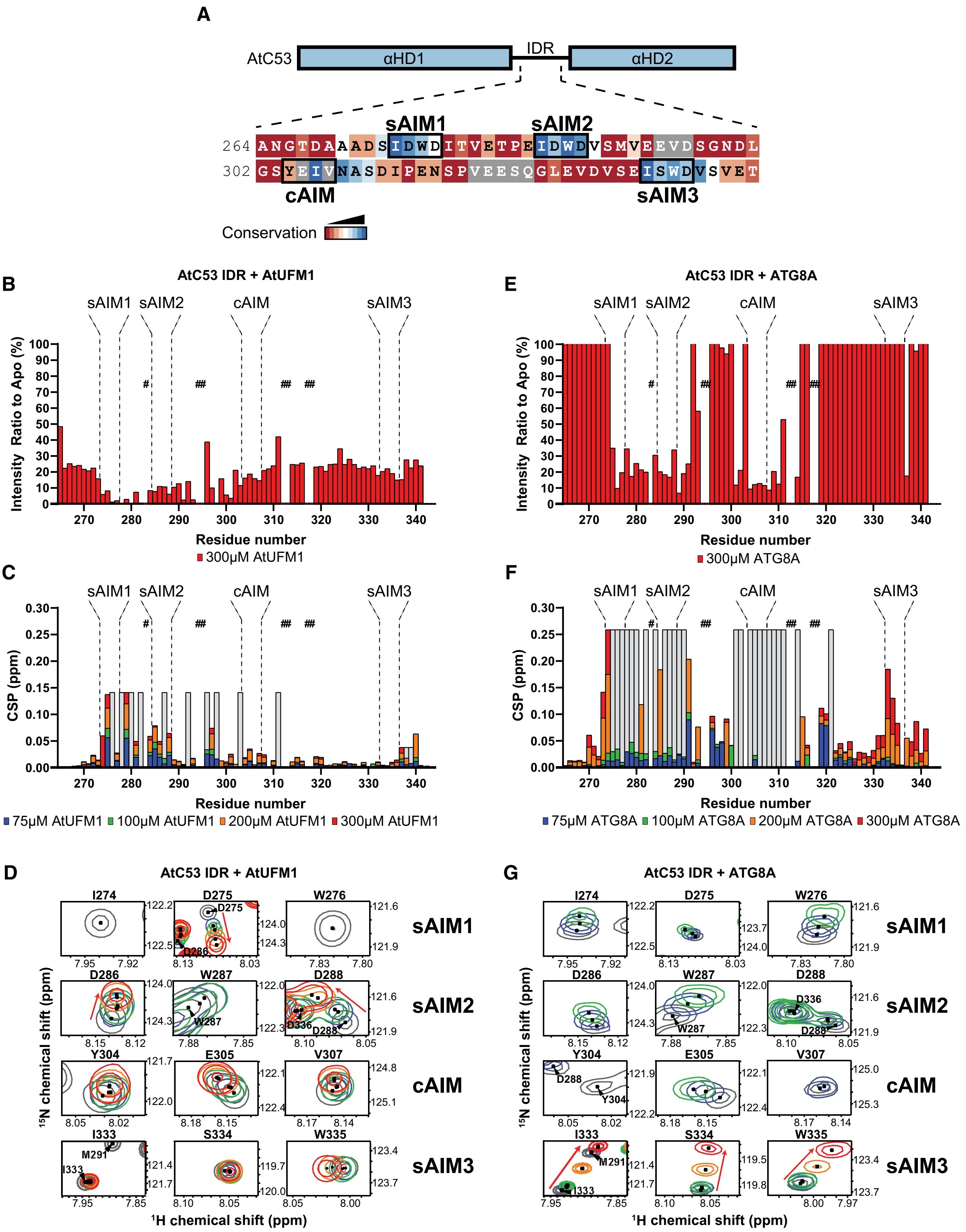
A
AtC53
αHD1
IDR
αHD2
sAIM1
sAIM2
264 ANGTDAAADSIDWDITVETPEIDWDVSMVEEVDSGNDL
302 GSYEIVNASDIPENSPVEESQGLEVDVSEISWDVSVET
cAIM
sAIM3
Conservation
B
AtC53 IDR + AtUFM1
Intensity Ratio to Apo (%)
Residue number
300μM AtUFM1
C
CSP (ppm)
Residue number
75μM AtUFM1 100μM AtUFM1 200μM AtUFM1 300μM AtUFM1
D
AtC53 IDR + AtUFM1
E
AtC53 IDR + ATG8A
300μM ATG8A
F
75μM ATG8A 100μM ATG8A 200μM ATG8A 300μM ATG8A
G
AtC53 IDR + ATG8A
15N chemical shift (ppm)
1H chemical shift (ppm)

Figure 4.

assays, showing that peptides derived from sAIM1 and sAIM2 and the cAIM bound to ATG8A (Appendix Fig S9D). As for UFM1 binding, the sAIM1,2 peptide displayed highest affinity for ATG8A (26.5 μM) than individual sAIMs, indicating that additional contacts from sAIM1,2 peptide extend beyond the canonical binding pocket of ATG8A and contribute to the binding. Notably, even though the affinity of individual sAIMs was low compared with the IDR, the interactions were specific, as the sAIM mutant peptide where the tryptophan was mutated to alanine did not show any change in Fluorescence anisotropy upon titration with both UFM1 and ATG8 (Appendix Fig S9C and D). Strikingly, residues covering amino acids that precede sAIM1 (265–272) and between cAIM and sAIM3 (315–332) experienced at least a threefold increase in their signal intensity upon ATG8A titration (Fig 4E; Appendix Fig S9B). However, they displayed minor chemical shift perturbations (Fig 4F), suggesting these residues do not directly bind ATG8A, but their dynamics change upon ATG8A binding. Altogether, these data suggest that certain regions in AtC53 IDR might be found in a conformational ensemble that is modulated upon binding of ATG8 but not UFM1. Also, in contrast to UFM1 binding, ATG8A binding modulates the conformational equilibrium of C53 IDR. In sum, although both UFM1 and ATG8 bind the sAIMs, their binding modes are different.

To dissect the interaction of sAIMs with UFM1 and ATG8, we used HsC53 IDR, since it lacks the cAIM and only contains sAIMs (Fig 2C). The backbone amide residues of HsUFM1 and GABARAP have been assigned previously (Kouno *et al*, 2002; Sasakawa *et al*, 2006). We successfully transferred 89% of the available backbone amide assignments of HsUFM1 and 85% of GABARAP to our 2D HSQC spectra. To characterize the interaction of C53 IDR with both UFM1 and GABARAP, we acquired 2D HSQC spectra of ^{15}N-labeled UFM1 and ^{15}N-labeled ATG8A/GABARAP in the presence and absence of unlabeled C53 IDR. The CSP analysis showed that the signals of Ser5, Arg15, Lys19, Leu21, Val23, Glu25, Ala31, Ala36, and Asn58 of HsUFM1 shifted upon HsC53 IDR binding (Fig EV3A–C). Additional residues such as Lys7, Asp13, Thr27, Thr30, Val32, Lys34, Phe35, Phe40, and Asn65 also experienced lower, yet important CSPs indicating a minor contribution of these residues for C53 IDR interaction (Fig EV3C). When we mapped CSPs onto the three-dimensional structure of HsUFM1, we observed a well-defined interaction site on the UFM1 surface covering the α-helix 1 (31–36) with contributions from residues in β-strand 1 (Ser5) and β-strand 2 (Lys19, Leu21, Val23, and Glu25; Fig EV3D). The C53 IDR binding site converges to a region that is involved in the interaction with the UBA5 LIR/UFIM (Habisov *et al*, 2016), suggesting C53 sAIM interacts with UFM1 in a similar manner to UBA5 LIR/UFIM. To test whether C53 IDR and UBA5 bind UFM1 similarly in plants, we acquired 2D HSQC spectra of ^{15}N-labeled AtUFM1 in the presence and absence of unlabeled AtC53 IDR or AtUBA5 LIR/UFIM peptide. Most of the signals that shifted upon AtC53 IDR binding followed the same trend when AtUFM1 is titrated with AtUBA5 LIR/UFIM, consistent with a conserved binding mode (Fig EV3E). Furthermore, mutation of the tryptophan residue in sAIM1 (AtC53 IDR^{W276A}) reduced chemical shift perturbations in AtUFM1 spectrum, supporting its dominant role in AtUFM1 binding (Figs 4C and D, and EV3E).

We next analyzed the HsC53 IDR-GABARAP interaction. The CSP analysis indicated that GABARAP residues Tyr25, Val33, Glu34, Lys35, Ile41, Asp45, Lys46, Tyr49, Leu50, Phe60, Leu63, Phe78, and Ala89 formed intermolecular contacts with C53 IDR (Fig EV4A–C). Additional residues such as Lys20, Ile21, Lys23, Ile32, Leu55, Phe62, and Ile64 displayed lower CSPs indicating a minor contribution of these residues in the interaction (Fig EV4C). Mapping of CSPs onto the three-dimensional structure of GABARAP highlighted the well-defined LIR docking site (LDS) on the GABARAP surface (Fig EV4D), composed of α-helix 2 (20–25), β-strand 2 (49–52), and α-helix 3 (56–68) residues. Canonical LIR/AIM binding involves the formation of an intermolecular β-sheet with β-strand 2 on ATG8-family proteins and the accommodation of the aromatic and aliphatic residues on two hydrophobic pockets (HP): HP1, which comprises residues in α-helix 2 and β-strand 2, and HP2, formed between the β-strand 2 and α-helix 3, commonly referred to as W and L-site, respectively (Noda *et al*, 2010). C53 IDR binding to GABARAP also induces CSPs for residues in β-strand 1 (28–35), closed to α-helix 1 (Fig EV4D). This region has been reported to undergo conformational changes that lead to the formation of a new hydrophobic pocket (HP0) in GABARAP surface upon HsUBA5 LIR/UFIM binding (Huber *et al*, 2020). This suggests, like UFM1, C53 sAIM-ATG8 binding mechanism is similar to UBA5 LIR/UFIM. We confirmed that these binding features are also conserved in plants by acquiring the 2D HSQC spectra of ^{15}N-labeled ATG8A in the presence and absence of unlabeled AtC53 IDR or AtUBA5 LIR/UFIM peptide. As for UFM1, most of the signals that shifted followed the same trend upon titration with either C53 IDR or UBA5 LIR/UFIM, demonstrating both motifs bind to a similar site on ATG8 (Fig EV4E). However, unlike its interaction with UFM1, mutating the aromatic residue in sAIM1 (AtC53 IDR^{W276A}) did not reduce CSPs in ATG8A spectrum (Fig EV4E), since binding can proceed via sAIM2 and cAIM residues. Altogether, these results indicate sAIMs bind UFM1 and ATG8 in a similar manner to UBA5 LIR/UFIM.

C53 sAIMs are crucial for C53-mediated autophagy and ER stress tolerance

Our evolutionary and structural analyses suggest that the sAIMs evolved and were selected for their ability to interact with both UFM1 and ATG8. What would happen if we converted sAIMs to cAIMs? We hypothesized that converting sAIMs into cAIMs would reduce the affinity of C53 toward UFM1 and lead to autoactivation of C53-mediated autophagy, even in the absence of ER stress (Fig 5A). To test this hypothesis, we generated an AtC53^{cAIM} mutant by reordering the residues of each sAIM from IDWD to WDDI. We first assessed the interaction of AtC53^{cAIM} with ATG8A by *in vitro* pulldowns. AtC53^{cAIM} bound ATG8A stronger than the wild-type C53 protein. Like the wild-type C53 protein, AtC53^{cAIM} interacted via the LIR docking site (LDS), as observed by competition with cAIM peptide and loss of interaction in the ATG8^{LDS} mutant (Fig 5B). On the contrary, AtC53^{cAIM} almost completely lost its ability to bind UFM1, consistent with the dependence of UFM1-binding on the sAIMs (Fig 5C).

To further corroborate our *in vitro* pulldown assays, we performed quantitative on-bead binding assays. GST-ATG8 and GST-GABARAP recruited C53^{cAIM} mutant 22% (mean average) and 35% (mean average) more efficiently than the respective C53 wild-type proteins (Fig 5D and E; Appendix Fig S10A and B). C53^{sAIM} mutant (with inactivated sAIMs) was instead recruited 74% (mean average) and 78% (mean average) less to GST-ATG8 and GST-

 © 2023 The Authors

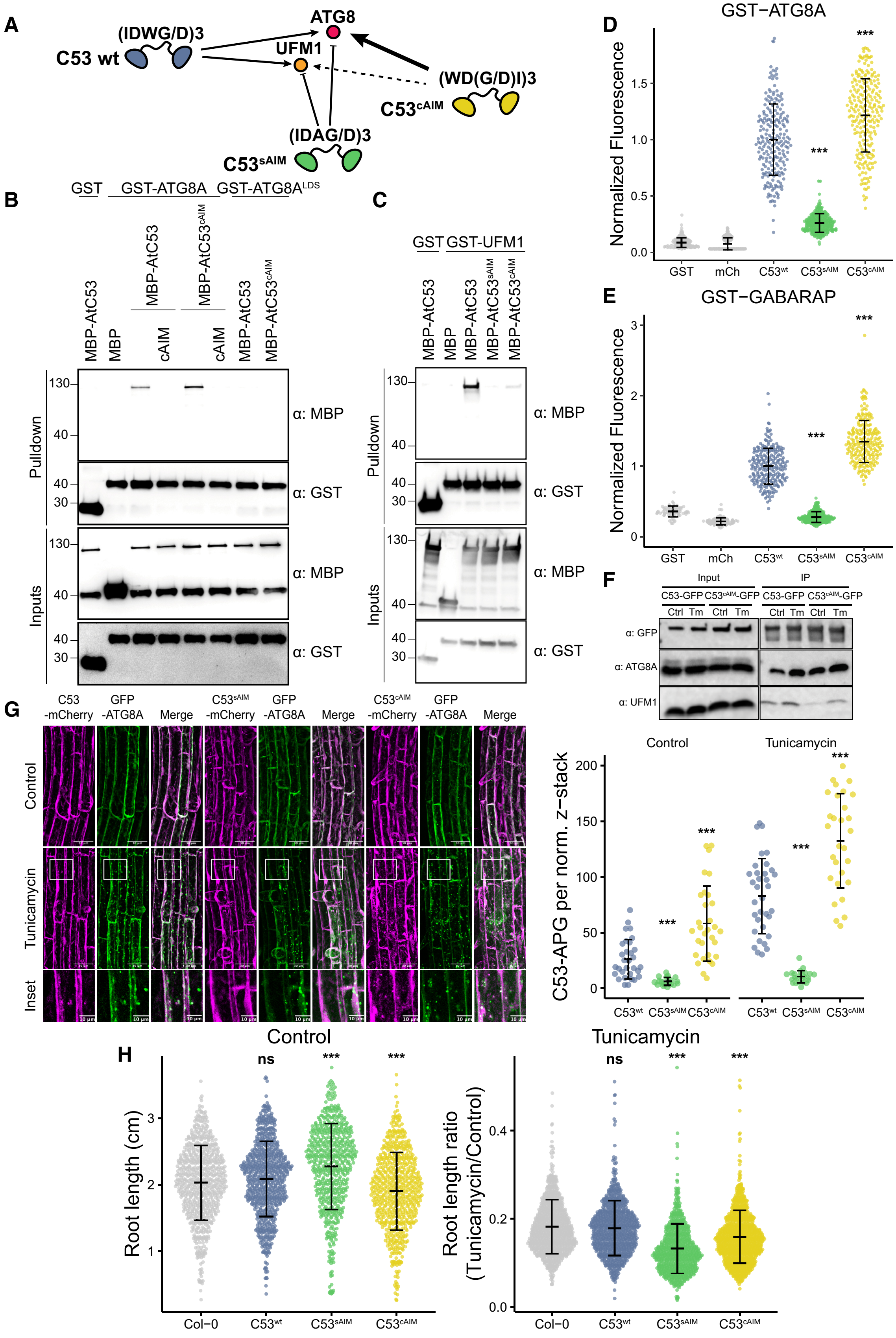
A
ATG8
(IDWG/D)3
C53 wt
UFM1
(WD(G/D)I)3
C53cAIM
(IDAG/D)3
C53sAIM
B
GST
GST-ATG8A
GST-ATG8ALDS
MBP-AtC53
MBP
MBP-AtC53
cAIM
MBP-AtC53cAIM
cAIM
MBP-AtC53
MBP-AtC53cAIM
Pulldown
Inputs
α: MBP
α: GST
C
GST
GST-UFM1
MBP-AtC53
MBP
MBP-AtC53
MBP-AtC53sAIM
MBP-AtC53cAIM
D
GST−ATG8A
Normalized Fluorescence
GST
mCh
C53wt
C53sAIM
C53cAIM
E
GST−GABARAP
F
Input
IP
C53-GFP
C53cAIM-GFP
Ctrl
Tm
α: GFP
α: ATG8A
α: UFM1
G
C53 -mCherry
GFP -ATG8A
Merge
C53sAIM -mCherry
C53cAIM -mCherry
Control
Tunicamycin
Inset
C53-APG per norm. z−stack
H
Root length (cm)
Root length ratio (Tunicamycin/Control)
Col−0
ns
***

Figure 5.

Figure 5. C53 sAIM sequences are essential for ER stress tolerance.

A Diagram summarizing our hypothesis that conversion of sAIMs to cAIMs would prevent C53-UFM1 interaction and strengthen C53-ATG8 interaction.

B, C Conversion of sAIM into cAIM leads to reduced UFM1 binding and stronger ATG8 interaction. Bacterial lysates containing recombinant proteins were mixed and pulled down with glutathione magnetic agarose beads. Input and bound proteins were visualized by immunoblotting with anti-GST and anti-MBP antibodies. AtC53^{sAIM}: AtC53 $^{(W276A,\ W287A,\ W335A)}$; AtC53^{cAIM}: AtC53$^{(IDWD274WDDI,\ IDWD285WDDI,\ IDWD333WDDI)}$; ATG8^{LDS}: ATG8^{YL50AA}.

D, E Microscopy-based protein–protein interaction assays showing C53^{cAIM} has increased affinity toward ATG8 or GABARAP. Glutathione-sepharose beads were prepared by incubating them with GST-ATG8A (D) or GST-GABARAP (E). The preassembled beads were then washed and mixed with (D) 1 μM of HsC53, 1 μM of HsC53^{sAIM} or 1 μM of HsC53^{cAIM} mutants or (E) 1 μM of AtC53, 1 μM of AtC53^{sAIM} or 1 μM of AtC53^{cAIM} mutants. HsC53^{sAIM}: HsC53$^{(W269A,\ W294A,\ W312A)}$; HsC53^{cAIM}: HsC53$^{(IDWG267WDGI,\ IDWG292WDGI,\ IDWG310WDGI)}$. The beads were then imaged using a confocal microscope. Representative confocal images for each condition are shown in Appendix Fig S10A and B. Normalized fluorescence is shown for each condition with the mean (± SD) of 3 replicate. Unpaired two-samples Wilcoxon test with continuity correction was performed to analyze the differences between wild type and mutants. ***P-value < 0.001. Total number of beads, mean, median, standard deviation and P-values are reported in Appendix Data S1.

F *In vivo* pull downs showing sAIM to cAIM conversion strengthens C53-ATG8 association and weakens C53-UFM1 association. 6-day old *Arabidopsis* seedlings expressing AtC53-GFP, AtC53^{cAIM}-GFP in *c53* mutant background were incubated in liquid 1/2 MS medium with 1% sucrose supplemented with DMSO as control (Ctrl) or 10 μg/ml tunicamycin (Tm) for 16 h and used for co-immunoprecipitation. Lysates were incubated with GFP-Trap Magnetic Agarose, input and bound proteins were detected by immunoblotting using the respective antibodies as indicated.

G AtC53^{cAIM} forms more GFP-ATG8A colocalizing puncta upon ER stress. *Upper Panel*, representative confocal images of transgenic *Arabidopsis* seedlings co-expressing C53-mCherry (magenta), C53^{sAIM}-mCherry and C53^{cAIM}-mCherry with GFP-ATG8a in *c53* mutant background under normal condition and after tunicamycin stress. 6-day old seedlings were incubated in liquid 1/2 MS medium with 1% sucrose supplemented with DMSO as control or tunicamycin (10 μg/ml) for 6 h before imaging. Scale bars, 30 μm. Inset scale bars, 10 μm. *Right Panel*, Quantification of the C53-autophagosomes (C53-APG) per normalized Z-stacks. Bars represent the mean (± SD) of at least 20 roots from 3 biological replicates for each genotype and treatment. Unpaired two-samples Wilcoxon test with continuity correction was performed to analyze the differences between wild type and mutants. ***P-value < 0.001.

H AtC53^{cAIM} mutant is sensitive to ER stress. Root length quantification of 7-day old *Arabidopsis* seedlings grown vertically on sucrose-free 1/2 MS agar plates supplemented with DMSO control (*Left Panel*, absolute root length in centimeters [cm]) or 100 ng/ml tunicamycin (*Right Panel*, ratio between the root length of tunicamycin-treated seedlings and the average of respective control condition). T4 transgenic lines expressing C53-GFP, C53^{sAIM}-GFP and C53^{cAIM}-GFP in *c53* mutant background were used. Statistical results of more than 500 seedlings from 3 biological repeats per each genotype for control and tunicamycin-treated condition are shown. Bars represent the mean (± SD) of 3 biological replicates. Unpaired two-samples Wilcoxon test with continuity correction was performed to analyze the differences between wild type and mutants. ^{ns}P-value > 0.05, ***P-value < 0.001.

GABARAP, respectively (Fig 5D and E; Appendix Fig S10A and B). These findings were also confirmed by ITC. While the C53^{sAIM} mutant showed no binding to GABARAP, the C53^{cAIM} showed increased affinity toward GABARAP with a K_D value of 11.24 μM (1.8-fold increase compared with the C53 wild type; Fig EV5A–C). In addition to ATG8, C53 also interacts with the scaffold protein FIP200/ATG11 (Turco *et al*, 2020; Zientara-Rytter & Subramani, 2020). We therefore tested the binding affinities of C53 and C53^{cAIM} to FIP200. Similar to our observations with ATG8, FIP200 interaction was lost in C53^{sAIM} mutant and HsC53^{cAIM} displayed a stronger interaction (Appendix Fig S11). These results demonstrate that converting sAIM to cAIM increases the affinity of C53 toward ATG8 and decreases its affinity to UFM1.

We next explored the physiological consequences of sAIM to cAIM conversion. We complemented an *Arabidopsis thaliana c53* mutant with either C53-GFP, C53^{sAIM}-GFP, or C53^{cAIM}-GFP fusions. Consistent with our *in vitro* data, *in vivo* pulldown assays showed that C53^{cAIM}-GFP had a stronger interaction with ATG8 than C53-GFP. On the contrary, the association between C53^{cAIM}-GFP and UFM1 was weaker than between C53-GFP and UFM1 (Fig 5F; Appendix Fig S12A and B).

Under normal conditions, Arabidopsis C53 predominantly has a diffuse cytoplasmic localization pattern. Upon ER stress, it is recruited to the ATG8-labeled autophagosomes to be degraded in the vacuole (Stephani *et al*, 2020). Consistent with our previous findings, C53 autophagic flux is impaired in UFMylation E2 enzyme mutant *ufc1* (Appendix Fig S12C and D; Stephani *et al*, 2020). This is consistent with a recent study, where UFC1 was shown to form a complex with C53-UFL1-DDRGK1 (Peter *et al*, 2022). Furthermore, in agreement with our *in vivo* pulldown results, C53^{cAIM}-mCherry formed puncta even under normal conditions, suggesting it associates with ATG8 and is recruited to the autophagosomes even in the absence of stress (Fig 5G). Altogether, these findings suggest sAIM to cAIM conversion leads to the premature activation of C53-mediated autophagy.

Finally, we measured ER stress tolerance of C53^{cAIM}-expressing Arabidopsis plants. Tunicamycin leads to the shortening of the roots in *Arabidopsis thaliana* (Brandizzi, 2021). Compared with wild-type complemented plants, C53^{cAIM}-expressing Arabidopsis lines formed shorter roots even under control conditions (Fig 5H). This suggests premature activation of C53 is detrimental for plant growth, likely due to the degradation of C53 without the bound cargo. The root length was further reduced in tunicamycin-containing plates, indicating the inability to degrade C53 cargo that arise upon ER stress is detrimental for plants. Taken together, our results illustrate that C53's ability to bind UFM1 and ATG8, which is encoded in sAIM regions, is crucial for its function and ER stress tolerance.

Discussion

Despite the discovery of UFMylation almost two decades ago, its structural basis, the full spectrum of UFMylated substrates, and its physiological role are still not fully resolved (Gerakis *et al*, 2019; Banerjee *et al*, 2020). Studies in metazoans and our recent work have shown that UFMylation is involved in a wide range of homeostatic pathways, including ER stress tolerance, immunity, autophagy, lipid droplet biogenesis, and the DNA damage responses (Qin *et al*, 2019; preprint: Eck *et al*, 2020; Kulsuptrakul *et al*, 2020; Liang *et al*, 2020; Liu *et al*, 2020; Stephani *et al*, 2020; Wang *et al*, 2020; Balce *et al*, 2021; Lee *et al*, 2021; Ishimura *et al*, 2022). In ER homeostasis, UFMylation is activated by stalling of ER-bound ribosomes and brings about the degradation of incomplete polypeptides, which can be toxic for the cell (Stephani *et al*, 2020; Wang *et al*, 2020).

© 2023 The Authors

Limited phylogenetic analysis, comparing yeast to plants and metazoans, suggested that the pathway had evolved in multicellular eukaryotes to relieve the protein synthesis burden resulting from extracellular matrix biogenesis (Walczak *et al*, 2019). However, our phylogenomic analysis, in agreement with previous studies, clearly shows that UFMylation did not evolve in multicellular eukaryotes, but was secondarily lost in fungi and other lineages (Grau-Bove *et al*, 2015; Tsaban *et al*, 2021; Fig 1). Indeed, many single-celled organisms including *Chlamydomonas* harbor a full complement of UFMylation components in their genome, whereas certain multicellular lineages, such as kelp (Phaeophyceae), have lost the majority of the pathway. Further, we provide biochemical and physiological evidence that UFMylation is functional in *Chlamydomonas*, unequivocally refuting the idea that it has evolved only in multicellular organisms (Fig 1). Our phylogenetic analysis also revealed that in addition to the Fungi, several algal groups, and pathogens such as *Plasmodium*, *Entamoeba*, and *Trichomonas* have also lost UFMylation, raising the question of how these species resolve stalled ER-bound ribosomes and why the pathway was expendable in these lineages. Comparative studies addressing these questions could provide potential translational avenues for developing genetic or chemical means to prevent infections.

Another conclusion of our phylogenetic studies is the tight connection between the presence of sAIMs located in the C53 IDR and UFM1. Species that lack UFM1 also lost the sAIMs in C53 (Fig 2). Using biochemical and structural approaches, we found that sAIMs form versatile docking sites that can interact with both UFM1 and ATG8. UFM1 interaction is mostly mediated by sAIM1 and sAIM2, whereas the ATG8 interaction is driven by the cAIM, sAIM1, and sAIM2 (Figs 2 and 4). It is surprising that the sAIM3, which is highly similar to sAIM1/2, does not show significant binding to UFM1. A plausible explanation is that the aspartic acid at the second positions in sAIM1 and 2 (I**D**WD) plays an important role for the interaction and having a serine instead of an aspartic acid in sAIM3 (I**S**WD) weakens the binding. Consistently, the NMR analyses showed that the signals of the residues neighboring sAIMs showed significant chemical shifts, suggesting that they also contribute to the interaction with both UFM1 and ATG8.

The NMR experiments revealed that UFM1 and ATG8 binding induces distinct conformational changes in the C53 IDR (Fig 4; Appendix Figs S8D and S9B). UFM1 binding reduces the overall signal intensity with further reduction at the direct binding sites corresponding to sAIM1 and sAIM2. On the contrary, ATG8 binding leads to a local signal intensity drop at the sAIM1-2 and cAIM but increases the signal intensity of residues that do not interact with ATG8. These data suggest that upon ATG8 binding C53 IDR becomes more dynamic, potentially allowing it to bind the autophagic cargo. These structural rearrangements could also affect the E3 ligase activity of the UFL1 enzyme complex. Indeed, a recent study has shown that C53 negatively regulates UFMylation activity, when bound to the UFL1-DDRGK1 complex (Peter *et al*, 2022). Altogether, these results indicate that evolution of suboptimal ATG8-interacting motifs enabled C53 to interact with another regulatory protein, UFM1, creating an autoinhibition mechanism that regulates ER-phagy. This illustrates how complex regulatory circuits could evolve by shuffling existing short linear motifs.

Interestingly, another noncanonical motif on UBA5, the E1 enzyme of the UFMylation cascade, can also bind both UFM1 and ATG8 through similar binding pockets (Figs EV3 and EV4). Removing or mutating UBA5 LIR affects the kinetics of UFMylation and the GABARAP-dependent recruitment of UBA5 to ER upon stress (Huber *et al*, 2020). Our findings go a step further and show that noncanonical motifs on C53 are essential for organismal fitness, as converting sAIMs to canonical AIMs leads to reduced ER stress tolerance in *Arabidopsis thaliana* (Fig 5H). Consistently, a recent study in mice also showed that C53 sAIMs are crucial for ER homeostasis and mutations in the UFMylation system leads to brain disorders (Ishimura *et al*, 2022). Further *in vitro* reconstitution studies that involve the UFMylation machinery, C53 receptor complex, and stalled membrane-bound ribosomes are necessary to understand the dynamic changes that lead to C53 activation. Altogether, these experiments could explain how UFMylation and autophagy intersect at the ER.

In summary, our data converge on the model that UFM1 and ATG8 compete for C53 binding via the shuffled ATG8-interacting motifs (Stephani *et al*, 2020). Under normal conditions, C53 is bound to UFM1, keeping it inactive. Upon stress, UFM1 is displaced by ATG8, leading to structural rearrangements that activate C53-mediated autophagy. These results provide a mechanism where the cell keeps selective autophagy pathways inactive under normal conditions to prevent the spurious degradation of healthy cellular components and saves the energy that is required to form autophagosomes.

Materials and Methods

Reagents and Tools table

Reagent/Resource	Reference or Source	Identifier or Catalog Number
**Experimental Models**		
wt (Arabidopsis thaliana)		Col-0
wt (Chlamydomonas reinhardtii)	Li *et al* (2016)	CC-4533
uba5 (Chlamydomonas reinhardtii)	Li *et al* (2019)	Cre13.g582350
ufl1 (Chlamydomonas reinhardtii)	Li *et al* (2019)	Cre16.g686650
ire1 (Chlamydomonas reinhardtii)	Li *et al* (2019)	Cre08.g371052

© 2023 The Authors

Reagents and Tools table (continued)

Reagent/Resource	Reference or Source	Identifier or Catalog Number
c53 (Arabidopsis thaliana)	Stephani *et al* (2020)	At5g06830
pUbi::C53-mCherry × GFP-ATG8A/*c53 (Arabidopsis thaliana)*	This study	N/A
pUbi::C53$^{\text{sAIM(W276A, W287A, Y304A, W335A)}}$-mCherry × GFP-ATG8A/*c53 (Arabidopsis thaliana)*	This study	N/A
pUbi::C53$^{\text{cAIM(IDWD274WDDI, IDWD285WDDI, IDWD333WDDI)}}$-mCherry × GFP-ATG8A/*c53 (Arabidopsis thaliana)*	This study	N/A
Arabidopsis thaliana: pUbi::C53-GFP × *c53 (Arabidopsis thaliana)*	Stephani *et al* (2020)	N/A
pUbi::C53$^{\text{sAIM(W276A, W287A, Y304A, W335A)}}$-GFP × *c53 (Arabidopsis thaliana)*	Stephani *et al* (2020)	N/A
pUbi::C53$^{\text{cAIM(IDWD274WDDI, IDWD285WDDI, IDWD333WDDI)}}$-GFP × *c53 (Arabidopsis thaliana)*	This study	N/A
**Recombinant DNA**		
E. coli: Destination (expression) vector	Stephani *et al* (2020)	N/A
E. coli: GST-ATG8A	Stephani *et al* (2020)	N/A
E. coli: GST-ATG8A$^{\text{LDS(YL50AA)}}$	Stephani *et al* (2020)	N/A
E. coli: GST-GABARAP	Stephani *et al* (2020)	N/A
E. coli: GST-CrATG8	This study	N/A
E. coli: GST-CrUFM1	This study	N/A
E. coli: HIS6-CrC53	This study	N/A
E. coli: MBP-CrC53	This study	N/A
E. coli: GST-AtUFM1	Stephani *et al* (2020)	N/A
E. coli: GST-HsUFM1	This study	N/A
E. coli: MBP-AtC53	Stephani *et al* (2020)	N/A
E. coli: MBP-AtC53$^{\text{IDR(239-372)}}$	Stephani *et al* (2020)	N/A
E. coli: MBP-AtC53$^{\Delta\text{IDR(1-239,(KGSGSTSGSG)2,373-549)}}$	Stephani *et al* (2020)	N/A
E. coli: MBP-HsC53	Stephani *et al* (2020)	N/A
E. coli: MBP-HsC53$^{\text{IDR(263-316)}}$	Stephani *et al* (2020)	N/A
E. coli: MBP-HsC53$^{\Delta\text{IDR(1-262, (KGSGSTSGSG),317-506)}}$	Stephani *et al* (2020)	N/A
E. coli: MBP-AtC53$^{\text{1A (W276A)}}$	Stephani *et al* (2020)	N/A
E. coli: MBP-AtC53$^{\text{2A (W287A)}}$	Stephani *et al* (2020)	N/A
E. coli: MBP-AtC53$^{\text{3A (W335A)}}$	Stephani *et al* (2020)	N/A
E. coli: MBP-AtC53$^{\text{12A (W276A, W287A)}}$	Stephani *et al* (2020)	N/A
E. coli: MBP-AtC53$^{\text{13A (W276A, W335A)}}$	Stephani *et al* (2020)	N/A
E. coli: MBP-AtC53 $^{\text{23A (W287A, W335A)}}$	Stephani *et al* (2020)	N/A
E. coli: MBP-AtC53$^{\text{123A(W276A, W287A, W335A)}}$	Stephani *et al* (2020)	N/A
E. coli: MBP-AtC53$^{\text{sAIM(Y304A, W276A, W287A, W335A)}}$	Stephani *et al* (2020)	N/A
E. coli: MBP-HsC53$^{\text{sAIM(W269A, W294A, W312A)}}$	Stephani *et al* (2020)	N/A
E. coli: MBP	Stephani *et al* (2020)	N/A
E. coli: HIS6-GABARAP	Stephani *et al* (2020)	N/A
E. coli: HIS6-AtC53	Stephani *et al* (2020)	N/A
E. coli: mCh-AtC53 $^{\text{sAIM (Y304A, W276A, W287A, W335A)}}$	This study	N/A
E. coli: mCh-HsC53 $^{\text{sAIM(W269A, W294A, W312A)}}$	This study	N/A
E. coli: mCh-AtC53	This study	N/A
E. coli: mCh-HsC53	This study	N/A
E. coli: GST	Stephani *et al* (2020)	N/A
E. coli: mCherry	This study	N/A
E. coli: MBP- *E. coli*: AtC53$^{\text{cAIM(IDWD274WDDI, IDWD285WDDI, IDWD333WDDI)}}$	This study	N/A

 © 2023 The Authors

Reagents and Tools table (continued)

Reagent/Resource	Reference or Source	Identifier or Catalog Number
E. coli: MBP-HsC53$^{cAIM(IDWG267WDGI,\ IDWG292WDGI,\ IDWG310WDGI)}$	This study	N/A
E. coli: mCh-AtC53$^{cAIM(IDWD274WDDI,\ IDWD285WDDI,\ IDWD333WDDI)}$	This study	N/A
E. coli: mCh-HsC53$^{cAIM(IDWG267WDGI,\ IDWG292WDGI,\ IDWG310WDGI)}$	This study	N/A
E. coli: HIS6-HsC53	Stephani *et al* (2020)	N/A
E. coli: HIS6-HsUFM1	Stephani *et al* (2020)	N/A
E. coli: HIS6-AtUFM1	Stephani *et al* (2020)	N/A
E. coli: MBP-PfC53	This study	N/A
E. coli: MBP-AcC53	This study	N/A
E. coli: HIS6-MBP-3C-AtC53 IDR $^{(264\text{-}341)}$	This study	N/A
E. coli: HIS6-MBP-3C-AtC53 IDR$^{1A\ (W276A)\ (264\text{-}341)}$	This study	N/A
E. coli: HIS6-MBP-3C-AtC53 IDR$^{2A\ (W287A)\ (264\text{-}341)}$	This study	N/A
E. coli: HIS6-3C-GABARAP	This study	N/A
E. coli: HIS6-3C-ATG8A	This study	N/A
E. coli: HIS6-3C-AtUFM1	This study	N/A
E. coli: HIS6-3C-HsUFM1	This study	N/A
E. coli: HIS6-MBP-3C-HsC53 IDR $^{(263\text{-}316)}$	This study	N/A
pUbi::C53$^{sAIM(W276A,\ W287A,\ Y304A,\ W335A)}$-mCherry	This study	N/A
pUbi::C53$^{cAIM(IDWD274WDDI,\ IDWD285WDDI,\ IDWD333WDDI)}$-mCherry	This study	N/A
pUbi::C53$^{cAIM(IDWD274WDDI,\ IDWD285WDDI,\ IDWD333WDDI)}$-GFP	This study	N/A
pUbi::C53-mCherry	Stephani *et al* (2020)	N/A
pUbi::C53-GFP	Stephani *et al* (2020)	N/A
pUbi::C53$^{sAIM(W276A,\ W287A,\ Y304A,\ W335A)}$-GFP	Stephani *et al* (2020)	N/A
**Antibodies**		
Goat anti-rabbit IgG HRP-Conjugate (1:10K)	Biorad	1706515
Goat anti-mouse IgG-HRP Conjugate (1:10K)	Biorad	1706516
Rabbit anti-mCherry (1:5K)	Abcam	ab167453
Goat anti-GST HRP Conjugate (1:1K)	GE Healthcare	RPN1236
Rabbit anti-GFP (1:3K)	Invitrogen	A11122
Mouse anti-GFP (1:3K)	Roche	11814460001
Mouse anti-MBP (1:3K)	Sigma Aldrich	M1321-200UL
Rabbit anti-ATG8A (1:1K)	Agrisera	AS14 2811
Rabbit anti-C53 (1:5K)	Stephani *et al* (2020)	N/A
Rabbit anti-AtUFM1 (1:3K)	This study	N/A
Rabbit anti-HsUFM1 (1:3K)	Abcam	Ab109305
**Oligonucleotides and other sequence-based reagents**		
Chlamydomonas reinhardtii primer: E3_P1	This study	AGAGCTCCTGCATACCCTGA
Chlamydomonas reinhardtii primer: E3_E1_SR	This study	CCGAGGAGAAACTGGCCTT
Chlamydomonas reinhardtii primer: E3_E1_oMJ	This study	CAGGCCATGTGAGAGTTTGC
Chlamydomonas reinhardtii primer: E3_P2	This study	CTCCTCAATGAGTGTGGCAA
Chlamydomonas reinhardtii primer: E1_P2	This study	CACACGGACATGACTGGAAC
Chlamydomonas reinhardtii primer: E1_P1	This study	AGAGTTACGGCCGCAGATT
**Chemicals, enzymes and other reagents**		
E. coli: DH5α	In-house facility	N/A
E. coli: Rosetta2 (DE3) pLysS	In-house facility	N/A
A. tumefaciens: GV3101 (pSoup)	In-house facility	N/A

© 2023 The Authors

Reagents and Tools table (continued)

Reagent/Resource	Reference or Source	Identifier or Catalog Number
cAIM *wt* peptide	Synthetized *in house*	EPLDFDWEIVLEEEM
cAIM mutant peptide	Synthetized *in house*	EPLDFDAEIALEEEM
AtUBA5 LIR peptide	Synthetized *in house*	GPLHDDNEWNISVVDD
HsUBA5 LIR peptide	Synthetized *in house*	EIIHEDNEWGIELVSE
Tunicamycin	SCBT	sc-3506
DTT	Sigma Aldrich	43815
Concanamycin-A (conA)	Santa Cruz	sc-202111A
Gamborg B5 vitamin mixture 1000X	Duchefa	G0415.0250
Gamborg B5 medium (microsalt mixture)	Duchefa	M0302.0025
Gamborg B5 medium (including vitamins)	Duchefa	G0210.0010
Gamborg B5 medium (basal salt mixture)	Duchefa	G0209.0050
Murashige & Skoog vitamin mixture 1000X	Duchefa	M0409.0250
Murashige & Skoog micro salt mixture	Duchefa	M0301.0050
Murashige & Skoog macro salt mixture	Duchefa	M0305.0050
Murshige & Skoog Basal salt mixture with MES	Duchefa	M0254.0050
Murashige & Skoog without nitrogen	Caisson labs	MSP07
MES monohydrate	Applichem	A1074
Puromycin	Sigma Aldrich	P8833
L-Glutamine	Sigma Aldrich	G7513
M9 Minimal media	In-house facility	N/A
Ammonium-^{15}N chloride	Sigma Aldrich	39466-62-1
D-Glucose (U-13C6, 99%)	Cambridge Isotope Laboratories, Inc.	110187-42-3
Thamine hydrochloride	Sigma Aldrich	T1270
Biotin	Sigma Aldrich	B4639
Choline chloride	Alfa Aesar	A15828
Folic acid	Acros Organics	21663
Niacinamide	Sigma Aldrich	N3376
D-Pantothenic acid hemicalcium salt	Sigma Aldrich	P2250
Pyridoxal hydrochloride	Alfa Aesar	A17855
(-)-Riboflavin	Sigma Aldrich	R4500
Ethylenedinitrilotetraacetic acid disodium salt dihydrate	Merck	108454
Iron (III) chloride hexahydrate Fe (III)$Cl_3 \cdot 6H_2O$	Merck	103943
Zinc chloride $ZnCl_2$	Merck	108816
Copper (II) chloride dihydrate Cu (II)$Cl_2 \cdot 2H_2O$	Sigma Aldrich	221783
Cobalt (II) chloride hexahydrate Co (II)$Cl_2 \cdot 6H_2O$	Sigma Aldrich	S2644
Boric acid	Sigma Aldrich	B6768
Manganese (II) chloride tetrahydrate Mn (II)$Cl_2 \cdot 4H_2O$	Sigma Aldrich	M3634
GFP-Trap	Chromotek	Gta-20
Glutathion Sepharose 4 B	Cytiva	17-5132-01
Pierce™ Glutathione Magnetic Agarose Beads	Thermo Scientific™	78601
HisTrap FF 5 ml	Cytiva	17525501
HisTrap FF 1 ml	Cytiva	17531901
Resource Q 6 ml	Cytiva	17117901
Resource S 6 ml	Cytiva	17118001

© 2023 The Authors

Reagents and Tools table (continued)

Reagent/Resource	Reference or Source	Identifier or Catalog Number
HiPrep 26/10 Desalting	Cytiva	17508701
HiLoad 16/600 Superdex 75 pg	Cytiva	28989333
HiLoad 16/600 Superdex 200 pg	Cytiva	28989335
GFP-Trap Magnetic Agarose	Chromotek	Gtma-20
Protein A Agarose	Sigma	P2545
**Software**		
CLC main work bench 7	Qiagen	N/A
Zen Software	Carl Zeiss	N/A
Image J (Fiji)	NIH	N/A
Image Lab	BioRad	N/A
iBright analysis software	Invitrogen	N/A
Adobe Illustrator 2022	Adobe Inc.	N/A
RStudio 2021.09.2+382 "Ghost Orchid" Release; R version 4.1.2	RStudio; The R Foundation for Statistical Computing	N/A
TopSpin3.2	Bruker	N/A
CcpNmr3.0	Continuum Analytics, Inc.	N/A

Methods and Protocols

Phylogenomic analysis

To reconstruct the evolutionary history of the UFMylation pathway, we searched for UFMylation proteins (including RPL26) in 153 eukaryotic datasets comprising 149 genomes and four transcriptomes from two dinoflagellates, *Togula jolla* and *Polarella glacialis*, and two CRuM (Collodictyonids, Rigifilids, Mantamonadids) taxa, including *Diphylleia rotans* and *Rigifila ramosa* (Appendix Data S1). CRuM transcriptomes were assembled from raw sequencing reads using Trinity v2.12.0, and proteins were predicted using TransDecoder v 5.5.0 (minimum protein size of 25 amino acids; Haas *et al*, 2013). Initially, *Homo sapiens* proteins were used as queries to search predicted proteomes using Diamond BLASTp v2.0.9 (E-value < 10^{-5}, ultra-sensitive mode; Buchfink *et al*, 2014). Multiple sequence alignments were then inferred using MAFFT v7.490 (—auto) and trimmed using trimAl v1.4 with a gap threshold of 30%, before preliminary phylogenies were generated using IQ-Tree v2.1.2 (LG4X model, fast mode; Capella-Gutiérrez *et al*, 2009; Katoh & Standley, 2013; Minh *et al*, 2020). The resulting phylogenies were annotated using SWISS-PROT (version 2022_01) and Pfam (version 35.0) and then interpreted in FigTree v1.4.2. From the phylogeny, orthologs were identified, extracted, and used as queries for a second iteration of BLAST searching as described previously (Boeckmann *et al*, 2003; Rambaut, 2012; El-Gebali *et al*, 2019). In order to improve search sensitivity, the orthologs identified using BLAST were then used to generate profile hidden Markov models (HMMs). To do this, the proteins were realigned with the structurally informed aligner MAFFT-DASH with the L-INS-i algorithm and were then trimmed with a gap threshold of 10% (Rozewicki *et al*, 2019). HMMs were then generated from the alignments and used to research the proteomic datasets using HMMER v3.1b2 (E-value < 10^{-5}; Mistry *et al*, 2013). The identified homologs were once again aligned, trimmed, and assessed phylogenetically, facilitating the removal of paralogs. Lastly, to account for the possibility that proteins could be missing due to genomic misannotation, proteins identified from the predicted proteomes were used as queries for tBLASTn (E-value < 10^{-5}) searches against eukaryotic genomes and protein predictions were generated using Exonerate v2.2 (see https://github.com/nickatirwin/Phylogenomic-analysis; Slater & Birney, 2005). Newly predicted proteins were combined with the previously identified proteins and were once again phylogenetically screened for paralogs. The presence and absence of the resulting orthologs was plotted across a eukaryotic phylogeny using ITOL v6 with taxonomic information inferred from NCBI Taxonomy following adjustments made based on recent phylogenomic analyses (Federhen, 2012; Burki *et al*, 2020; Letunic & Bork, 2021). Pairwise binary correlations between UFMylation pathway proteins were calculated as phi coefficients in R v.4.2.0 using the "psych" package (Revelle & Revelle, 2015) and were clustered using the Ward D2 method with Euclidean distances through the "pheatmap" package (Kolde & Kolde, 2018). UFMylation HMMs were also used to search bacterial and archaeal reference proteomes downloaded from UniProt (release 2022_04). Phylogenies including prokaryotic homologs were generated as described above and visualized in ITOL v6.

To investigate the sequence conservation of C53 and RPL26, multiple sequence alignments were generated from the identified orthologs using MAFFT with the L-INS-i algorithm. The alignments were then trimmed using a gap threshold of 30%, and fragmented sequences with less than 50% data were filtered out. In the case of C53, alignment of the poorly conserved intrinsically disordered region (IDR) was improved through realignment of specific blocks using MUSCLE v3.8 implemented in AliView v1.26 (Edgar, 2004; Larsson, 2014). For C53, phylogenetic analyses were conducted using IQ-Tree and substitution models were selected using ModelFinder (LG + F + R6; Kalyaanamoorthy *et al*, 2017). The phylogeny and C53 alignment were then used in an analysis using ConSurf to examine sequence conservation (query sequence: Q9FG23, model: WAG). Likewise, both the C53 and RPL26 alignments were used to assess sequence conservation and divergence between species with

and without UFM1 using TwinCons (using the LG substitution model and Voronoi clustering; Penev *et al*, 2021). Lastly, alignment logos for the C53 shuffled AIMs were generated with Skylign using weighted counts (Wheeler *et al*, 2014).

Cloning procedures

Constructs for *Arabidopsis thaliana* and *Escherichia coli* transformation were generated using the GreenGate (GG) cloning method (Lampropoulos *et al*, 2013). Plasmids used are listed in Materials and Methods section. The coding sequence of genes of interest was either ordered from Twist Biosciences or Genewiz or amplified from Col-0 using the primers listed in the Materials and Methods section. The internal *Bsa*I sites were mutated by site-directed mutagenesis without affecting the amino acid sequence.

Chlamydomonas reinhardtii genomic DNA extraction

The following protocol was adapted from Perlaza *et al* (2019). A 6 ml aliquot of a liquid TAP culture in mid-log phase was spun down, and the media were decanted. The pellet was resuspended in 400 µl of water, and then 1 volume of DNA lysis buffer was added (200 mM Tris–HCl pH 8.0, 6% SDS, 2 mM EDTA). To digest proteins, 5 µl of 20 mg/ml proteinase K (Thermo Fisher) was added and allowed to incubate at room temperature (RT) for 15 min. 200 µl of 5 M NaCl was then added and mixed gently. Next, to selectively precipitate nucleic acids, 160 µl of 10% CTAB in 0.7 M NaCl was added and allowed to sit for 10 min at 65°C with gentle agitation. Two or more consecutive rounds of DNA extraction using ultrapure phenol:chloroform:isoamyl alcohol (25:24:1, v/v/v) were performed to achieve a clean interphase. Then, the upper aqueous phase was retained and mixed with 1 volume of 2-propanol. This was mixed gently for 15 min at RT. Then, it was spun down for 30 min at 21,000 *g* at 4°C. The supernatant was removed, and 1 volume of ice-cold 70% ethanol was added and mixed with the pellet. This mixture was spun down for 15 min at 21,000 *g*. The supernatant was removed, and the DNA precipitate was dried in a speed-vac for about 10–25 min and resuspended in 40 µl of nuclease-free water.

The purity of the genomic DNA preparation was assessed using a spectrophotometer, ensuring absorbance ratios at 260/280 nm and 260/230 nm to be ~ 1.8 and ~ 2.0, respectively, prior to using the genomic DNA preparation for most of the follow-up applications.

Genotyping of the Chlamydomonas reinhardtii mutants

The insertion of the mutagenic cassette (PARO) in the UBA5 and UFL1 loci was verified by PCR by using primers designed to anneal inside and outside of the PARO cassette, using KOD Extreme Hot Start DNA Polymerase (Sigma). The PCR products were run on 1% (w/v) agarose. The primer sequences and expected PCR products can be found in Materials and Methods.

Chlamydomonas reinhardtii in vivo UFMylation assays

Cell cultures were grown in liquid TAP medium in 100 ml Erlenmeyer flasks for about 2 days to an OD_{600} of 1.5–2. These cultures were then transferred to fresh liquid TAP medium, with or without 0.2 mg/l Tunicamycin, to a final OD_{600} of 0.1. After either 12 or 24 h of treatment, 5 ml of cell culture was spun down, flash-frozen in liquid nitrogen, and stored at −70°C.

The pellets were thawed and resuspended in 150 µl of SDS-lysis buffer (100 mM Tris–HCl pH 8.0, 600 mM NaCl, 4% SDS, 20 mM EDTA, freshly supplied with Roche Protease Inhibitors). Samples were vortexed for 10 min at RT and centrifuged at maximum speed for 15 min at 4°C to remove the cell debris. The supernatant, containing a total extract of denatured proteins, was transferred to a new eppendorf tube, and a 5 µl aliquot was saved for BCA quantification and diluted accordingly.

5× SDS loading buffer (250 mM Tris–HCl pH 6.8, 5% SDS, 0.025% bromophenol blue, 25% glycerol), freshly supplied with 5% of β-mercaptoethanol, was added to the extract and denatured at 90°C for 10 min. The samples were loaded on 4–20% SDS–PAGE gradient gel (BioRad), and electrophoresis was run at 100 V for 1.5 h.

Chlamydomonas reinhardtii ribosome purification

Cell cultures were grown in liquid TAP medium in 250 ml Erlenmeyer flasks for about 2 days to an OD600 of 1.5–2. These cultures were then grown with or without 0.2 mg/l Tunicamycin to a final OD_{600} of 0.1. After 24 h of treatment, 50 ml of cell culture was spun down and resuspended in lysis buffer (200 mM Tris pH 9.0, 200 mM KCl, 35 mM $MgCl_2$, 1% Triton X-100, 2% Polyoxyethanl (10)tridecyl ether, 0.5% Na-deoxycholate, 1 mM Dithiothreitol, 100 µg/ml Cycloheximide) freshly supplied with Roche Protease Inhibitors. The cell suspension was frozen in liquid nitrogen and stored at −70°C. Frozen cells were then cryo-milled for 1 min, 4 times at 20 s^{-1} using a Retsch Mixer Mill MM400. Cell powder was thawed, transferred to an Eppendorf Tube, and centrifuged 30 min, 4°C, 13,000 *g*. The supernatant was transferred in a fresh Eppendorf tube.

To purify the ribosomes from the lysate, a 30% sucrose cushion centrifugation was performed. 1 ml supernatant was loaded on top of 1 ml cushion (20 mM Tris pH 8.0, 150 mM NaCl, 5 mM $MgCl_2$, 1 mM Dithiothreitol, 30% Sucrose) in open-top thick wall polycarbonate tube (Beckman Coulter®, 349622). Samples were centrifuged 16 h, 4°C, 70,000 rpm (TLA 100.3 rotor, Beckman Coulter Optima™ MAX-XP Ultracentrifuge). Supernatant was discarded and pellet resuspended in 20 mM Tris pH 8.0, 150 mM NaCl, 5 mM $MgCl_2$, 1 mM Dithiothreitol.

Samples (total lysate and purified ribosomes) were subsequently analyzed by western blotting and mass spectrometry.

For mass spectrometry, samples were precipitated prior to mass spec sample preparation. Samples were filled up to 1 ml with H2O, and 10 µl of 10% sodium deoxycholate (NaDoc) was added and vortexed. Precipitation followed with 100 µl of 50% TCA and incubation for 10 min of ice. The precipitated was pelletized (20 min, 4°C, 20,000 *g*). The pellet was washed in 1 ml of 100% acetone and pelletized again. Supernatant was discarded and pellet dried. Samples were prepared and tryptic digested, using the iST 96x kit (PO 00027, PreOmics) according to the manufacturer's description. 1.5 µg of each generated peptide sample was analyzed by nanoLC-MS/MS on an Orbitrap Eclipse Tribrid mass spectrometer. The mass spectrometry proteomics data have been deposited to the ProteomeXchange Consortium through the PRIDE partner repository (Perez-Riverol *et al*, 2019).

Chlamydomonas reinhardtii in vivo co-immunoprecipitation and mass spectrometry analysis

A 4 mg of total protein lysate was incubated with homemade UFM1 antibody (2.5 h, 4°C, rotating) in Co-IP buffer (50 mM Tris–HCl, pH

© 2023 The Authors

7.5, 150 mM NaCl, 10 mM $MgCl_2$, 10% glycerol, 0.1% Nonidet P-40, Protease Inhibitor Cocktail tablet). Protein A Agarose beads were added and incubated for two more hours. Beads were washed six times with the Co-IP buffer by pelleting gently the agarose beads (1 min, 4°C, 850 rpm). Sample was eluted by adding 5× SDS Loading buffer and incubating at 95°C for 10 min.

Coomassie-stained gel bands corresponding to the molecular weight of double UFMylated RPL26 were in-gel digested with Arg-C (Arg-C sequencing grade, Promega) as described (Shevchenko, 2006) with the following modifications: Following washes and acetonitrile dehydration, gel bands were reduced with 6 mM DTT in 100 mM ammonium bicarbonate (ABC) at 56°C for 30 min. Excess buffer was discarded, and gel bands were alkylated with 100 µl of 27 mM Iodoacetamide in 100 mM ABC at room temperature for 30 min in the dark. Following washes and acetonitrile dehydration, bands were rehydrated in 30 µl Arg-C buffer (100 mM Tris–HCl pH 8.0, 2 mM $CaCl_2$, 2 mM DTT) containing 50 ng/µl Arg-C. After 5 min incubation at 5°C, they were overlaid by another 30 µl Arg-C buffer and incubated at 37°C overnight. Gel pieces were extracted twice by addition of 20 µl 5% formic acid and sonication for 10 min in a cooled ultrasonic bath.

A similar aliquot of each band extract was analyzed by nanoLC-MS/MS on an Orbitrap Exploris 480 mass spectrometer. The mass spectrometry proteomics data have been deposited to the ProteomeXchange Consortium through the PRIDE partner repository (Perez-Riverol *et al*, 2019).

nanoLC-MS/MS analysis

The nano-HPLC system (UltiMate 3000 RSLC nano system, Thermo Fisher Scientific) was coupled to an Orbitrap Exploris 480/Orbitrap Eclipse Tribrid mass spectrometer equipped with a FAIMS pro interface and a Nanospray Flex ion source (all parts Thermo Fisher Scientific). Peptides were loaded onto a trap column (PepMap Acclaim C18, 5 mm × 300 µm ID, 5 µm particles, 100 Å pore size, Thermo Fisher Scientific) at a flow rate of 25 µl/min using 0.1% TFA as mobile phase. After 10 min, the trap column was switched in line with the analytical column (PepMap Acclaim C18, 500 mm × 75 µm ID, 2 µm, 100 Å, Thermo Fisher Scientific) operated at 30°C. Peptides were eluted using a flow rate of 230 nl/min, starting with the mobile phases 98% A (0.1% formic acid in water) and 2% B (80% acetonitrile, 0.1% formic acid) and linearly increasing to 35% B over the next 60 min (Exploris) or 180 min (Eclipse), respectively.

The Exploris 480 mass spectrometer was operated in data-dependent mode, performing a full scan (m/z range 350–1,200, resolution 60,000, target value 1E6) at three different compensation voltages (CV -45, -60, and -75), followed each by MS/MS scans of the most abundant ions for a cycle time of 1 s per CV. MS/MS spectra were acquired using an HCD collision energy of 30%, isolation width of 1.0 m/z, resolution of 30.000, target value at 2E5, minimum intensity of 5E4, and maximum injection time of 100 ms. Precursor ions selected for fragmentation (including charge state 2–6) were excluded for 10 s. The monoisotopic precursor selection (MIPS) mode was set to peptide, and the exclude isotopes feature was enabled.

The Eclipse was operated in data-dependent mode, performing a full scan (m/z range 350–1,500, resolution 120,000, target value 1E6) at four different compensation voltages (CV-45, −55, −65, and −75), followed each by MS/MS scans of the most abundant ions for a cycle time of 0.75 s per CV. MS/MS spectra were acquired using an isolation width of 1.2 m/z, target value of 3E4 and intensity threshold of 5E4, maximum injection time 20 ms, HCD with a collision energy of 30%, using the Iontrap for detection in the rapid scan mode. Precursor ions selected for fragmentation (including charge state 2–6) were excluded for 60 s. The monoisotopic precursor selection (MIPS) mode was set to Peptide, and the exclude isotopes feature was enabled.

Mass spectrometry data processing

For peptide identification, the RAW files were loaded into Proteome Discoverer (version 2.5.0.400, Thermo Scientific). All hereby created MS/MS spectra were searched using MSAmanda v2.0.0.19924 (Dorfer *et al*, 2014).

Peptide and protein identification was performed in two steps. For the initial search, the RAW files were searched against an Uniprot database that was filtered for the organism Chlamydomonas_reinhardtii and combined with common contaminants and sequences of tagged proteins of interest (18,942 sequences; 14,090,486 residues), using the following search parameters: Iodoacetamide derivative on cysteine was set as a fixed modification and oxidation of methionine as variable modification. The peptide mass tolerance was set to ±10 ppm, the fragment mass tolerance was set to ±10 ppm for the Orbitrap MS2 scans, to ±400 mmu for Iontrap scans, and the maximal number of missed cleavages was set to 2, using tryptic enzymatic specificity without proline restriction. The result was filtered to 1% FDR on protein level using the Percolator algorithm integrated in Proteome Discoverer (Käll *et al*, 2007) .

A sub-database of proteins identified in this search was generated for further processing. For the second search, the RAW files were searched against the created sub-database using the same settings as above plus considering additional variable modifications: Phosphorylation on serine, threonine, and tyrosine; deamidation on asparagine and glutamine; UFM_VG (monoisotopic mass of 156.089878 Da) on lysine; UFM_DRVG (427.217932 Da) on lysine and glutamine to pyro-glutamate conversion at peptide N-terminal glutamine; and acetylation on protein N terminus.

The localization of the post-translational modification sites within the peptides was performed with the tool ptmRS, based on the tool phosphoRS (Taus *et al*, 2011).

Identifications were filtered again to 1% FDR on protein and PSM level. Additionally, an MSAmanda score cutoff of at least 150 and 70 was applied for high- and low-resolution spectra, respectively, and proteins were filtered to be identified by a minimum of 2 PSMs in at least 1 sample.

Peptides were subjected to label-free quantification using IMPapQuant (Doblmann *et al*, 2019). Proteins were quantified by summing unique and razor peptides and applying intensity-based absolute quantification (iBAQ; Schwanhäusser *et al*, 2011). Protein-abundances-normalization was done using sum normalization, based on the MaxLFQ algorithm (Cox *et al*, 2014).

***Chlamydomonas reinhardtii* survival assays**

Cell cultures were grown in liquid TAP medium in a 100 ml Erlenmeyer flask for about 2 days to an OD_{600} of 1.5–2. These cultures were then transferred to fresh liquid TAP medium, with or without 0.2 mg/l Tunicamycin, to a final OD_{600} of 0.1. After 24, 48, and

© 2023 The Authors

72 h of treatment, the optical density (OD) of the cultures was measured using a spectrophotometer at 600 nm.

Arabidopsis thaliana plant materials and growth conditions

The Columbia-0 (Col-0) accession of *Arabidopsis* was used in this study unless otherwise indicated. *Arabidopsis* mutants used in this study are listed in the Materials and Methods section. Generation of transgenic *Arabidopsis* plants was carried out by *Agrobacterium*-mediated transformation (Bechtold & Pelletier, 1998).

Seeds were imbibed at 4°C for 3 days in dark. For the co-immunoprecipitation experiment, seeds were sterilized and cultured in liquid 1/2 MS medium containing 1% sucrose with constant shaking under continuous LED light. For the root length measurements, seeds are sterilized and sown on sucrose-free 1/2 MS agar plates and grown at 22°C at 60% humidity under continuous white light at 12/12-h light/dark cycle.

Root length quantification

Seedlings were grown vertically for 7 days on sucrose-free 1/2 MS plates supplemented with indicated chemicals. Plates were photographed using a Canon EOS 80D camera. The root length was measured using ImageJ software (version: 2.1.0/1.53c) for further analysis (Schindelin *et al*, 2012).

In vivo co-immunoprecipitation

Arabidopsis seedlings were cultured in liquid 1/2 MS medium with 1% sucrose for 7–8 days. These seedlings were then treated for additional 16 h in 1/2 MS liquid medium with 1% sucrose supplemented with DMSO or tunicamycin, respectively. About 1–2 mg plant material was harvested and homogenized using liquid nitrogen and immediately dissolved in grinding buffer (50 mM Tris–HCl, pH 7.5, 150 mM NaCl, 10 mM $MgCl_2$, 10% glycerol, 0.1% Nonidet P-40, Protease Inhibitor Cocktail tablet) by vortex. Plant lysates were cleared by centrifugation at 16,000 *g* for 5 min at 4°C several times. After binding to Protein A Agarose, 3 mg total plant protein was incubated with 25 µl GFP-Trap Magnetic Agarose beads (ChromoTek) at 4°C for 2.5 h. Pellets were washed with grinding buffer for six times and boiled for 10 min at 95°C prior to immunoblotting with the respective antibodies.

Autophagy flux assay in Arabidopsis thaliana

A total of 15–25 seedlings were cultured in liquid 1/2 MS medium for 5 days under continuous light with shaking at 80 rpm. Seedlings were then treated in liquid 1/2 MS medium supplemented with indicated chemicals (10 µg/ml tunicamycin, 1 µm concanamycin A) for 16 h. DMSO was supplemented for the control samples. Seedlings were then frozen in liquid nitrogen after the chemical treatment and homogenized. For western blotting, SDS loading buffer was added and the samples were boiled at 95°C for 10 min. Lysates were cleared by centrifugation at 16,000 *g* for 10 min. Protein concentration was measured by Coomassie brilliant blue G-250 method. 5 µg of total lysate was loaded per lane.

Confocal microscopy

Arabidopsis roots were imaged using a Zeiss LSM780 confocal microscope with an Apochromat 20× objective lens at 2× magnification. Z-stack merged images with 2 µm thickness per Z-stack were used for analysis. At least 5 Z-stacks were used for puncta quantification and image presentation. Confocal images were processed with ImageJ software (Schindelin *et al*, 2012).

Quantification of confocal micrographs

ImageJ software (version: 2.1.0/1.53c; Schindelin *et al*, 2012) is used for autophagic puncta number quantification. ATG8A puncta colocalized C53 punctuates were manually mounted for each stack and added for all stacks for a single image. Autophagosome number per normalized Z-stack was calculated by total autophagosome number of a certain image divided by the relative root area.

Western blotting

Blotting on nitrocellulose membranes was performed using a semidry Turbo transfer blot system (BioRad). Membranes were blocked with 5% skimmed milk or BSA in TBS and 0.1% Tween 20 (TBS-T) for 1 h at room temperature or at 4°C overnight. This was followed by incubation with primary and subsequent secondary antibody conjugated to horseradish peroxidase. After five 5 min washes with TBS-T, the immune reaction was developed using either Pierce™ ECL Western Blotting Substrate (ThermoFisher) or SuperSignal™ West Pico PLUS Chemiluminescent Substrate (ThermoFisher) and detected with either ChemiDoc Touch Imaging System (BioRad) or iBright Imaging System (Invitrogen).

Western blot image quantification

Protein band intensities were quantified with ImageJ (Schindelin *et al*, 2012). Equal rectangles were drawn around the total protein gel lane and the band of interest. The area of the peak in the profile was taken as a measure of the band intensity. The protein band of interest was normalized for the total protein level of the protein lane used as a bait. Average relative intensities and a standard error of three independent experiments were calculated.

Protein expression and purification for biochemical assays

Recombinant proteins were produced using *E. coli* strain Rosetta2 (DE3) pLysS grown in 2× TY media at 37°C to an A_{600} of 0.4–0.6 followed by induction with 300 µM IPTG and overnight incubation at 18°C.

For *in vitro* UFMylation assays, *in vitro* pulldowns, and *in vitro* protein–protein microscopy binding assays, pelleted cells were resuspended in lysis buffer (100 mM HEPES pH 7.5, 300 mM NaCl) containing protease inhibitors (Complete™, Roche) and sonicated. The clarified lysate was first purified by affinity, by using HisTrap FF (GE HealthCare) columns. The proteins were eluted with lysis buffer containing 500 mM imidazole. The eluted fraction was buffer exchanged to 10 mM HEPES pH 7.5, 100 mM NaCl and loaded either on Cation Exchange (ResourceS, Cytiva) or Anion Exchange (ResourceQ, Cytiva) chromatography columns. The proteins were eluted from 5 to 55% of Ion exchange buffer B (10 mM HEPES pH 7.5, 1 M NaCl by NaCl) gradient in 20 CV. Finally, the proteins were separated by size-exclusion chromatography with HiLoad® 16/600 Superdex® 200 pg or HiLoad® 16/600 Superdex® 75 pg, which were previously equilibrated in 50 mM HEPES pH 7.5, 150 mM NaCl.

The proteins were concentrated using Vivaspin concentrators (3,000, 5,000, 10,000, or 30,000 MWCO). Protein concentration was calculated from the UV absorption at 280 nm by DS-11 FX+ Spectrophotometer (DeNovix).

© 2023 The Authors

Protein expression and purification for nuclear magnetic resonance (NMR) spectroscopy

All recombinant proteins were produced using *E. coli* strain Rosetta2 (DE3) pLysS. Transformed cells were grown in 2× TY media supplemented with 100 μg/ml spectinomycin at 37°C to log phase (OD_{600} 0.6–0.8), followed by induction with 300 μM isopropyl β-D-1-thiogalactopyranoside (IPTG) and incubation at 18°C overnight. Recombinant isotopically labeled proteins used for nuclear magnetic resonance (NMR) spectroscopy were grown in M9 minimal media as described previously (Marley *et al*, 2001) supplemented in the presence of 100 μg/ml spectinomycin at 37°C to log phase (OD_{600} 0.6–0.8), followed by induction with 600 μM isopropyl β-D-1-thiogalactopyranoside (IPTG) and incubation at 18°C overnight. Cells were harvested by centrifugation and resuspended in lysis buffer of 100 mM Sodium Phosphate (pH 7.0), 300 mM NaCl, 20 mM imidazole supplemented with Complete EDTA-free protease inhibitor (Roche) and benzonase. Cells were lysed by sonication, and lysate was clarified by centrifugation at 20,000 *g*. The clarified lysate was loaded on a HisTrapFF (GE Healthcare) column pre-equilibrated with the lysis buffer. Proteins were washed with lysis buffer for 10 CV and eluted with lysis buffer containing 500 mM Imidazole. The eluted fraction was buffer exchanged to 10 mM Sodium Phosphate (pH 7.0), 50 mM NaCl and loaded either on Cation Exchange (ResourceS, Cytiva) or Anion Exchange (ResourceQ, Cytiva) chromatography columns. The proteins were eluted by NaCl gradient (50% in 20 CV). Samples were further purified by size-exclusion chromatography with HiLoad 16/600 Superdex 200 pg or HiLoad 16/600 Superdex 75 pg (GE Healthcare) with 50 mM Sodium Phosphate (pH 7.0), 100 mM NaCl. The proteins were concentrated using VivaSpin concentrators (3,000, 5,000, 10,000, or 30,000 MWCO). Protein concentration was calculated from the UV absorption at 280 nm by DS-11 FX+ Spectrophotometer (DeNovix) or at 205 nm by Jasco V-750 UV–Visible Spectrophotometer.

In vitro UFMylation assays

CrUBA5, CrUFC1, and UFM1 were mixed to a final concentration of 5 μM, 5 μM, and 20 μM, respectively, in a buffer containing 25 mM HEPES pH 7.5, 150 mM NaCl and 10 mM $MgCl_2$. The enzymatic reaction was started by adding ATP to a final concentration of 5 μM. The enzymatic mixture was incubated for 1 h at 37°C and then stopped with the addition of nonreducing Laemmli Loading Buffer. Beta-MercaptoEthanol (BME) was added only where specified to reduce UBA5-UFM1 or UFC1-UFM1 thioester bond. The samples were loaded on 4–20% SDS–PAGE gradient gel (BioRad), and electrophoresis was run at 100 V for 1.5 h.

In vitro pulldowns

For pulldown experiments, 5 μl of glutathione magnetic agarose beads (Pierce Glutathione Magnetic Agarose Beads, Thermo Scientific) was equilibrated by washing them two times with wash buffer (100 mM Sodium Phosphate pH 7.2, 300 mM NaCl, 1 mM DTT, 0.01% (v/v) IGEPAL). Normalized *E. coli* clarified lysates or purified proteins were mixed, according to the experiment, added to the washed beads, and incubated on an end-over-end rotator for 1 h at 4°C. Beads were washed five times with 1 ml wash buffer. Bound proteins were eluted by adding 50 μl Laemmli buffer. Samples were analyzed by western blotting or Coomassie staining.

Microscopy-based on-bead protein–protein interaction assays

Glutathione-Sepharose 4B bead slurry (Cytiva, average diameter 90 μm) was washed and diluted 10 times in HEPES buffer (25 mM HEPES pH 7.5, 150 mM NaCl, 1 mM DTT). The beads were then incubated for 30 min at 4°C (16 rpm horizontal rotation) with GST-tagged bait proteins (2 μM of GST, GST-FIP200 CD, GST-ATG8A, GST-GABARAP, GST-AtUFM1, and GST-HsUFM1). The beads were washed five times in 10 times the bead volume of HEPES buffer. The buffer was removed, and the beads were resuspended 1:20 in HEPES buffer. 10 μl of diluted beads was mixed with 20 μl of mCherry-tagged binding partner at a concentration of 1.5 μM (0.5 μl bead slurry and 1 μM binding partner final concentrations) with or without competitor, as stated in the relative experiment. The mixture was transferred to a black, glass bottom, 384-well plate (Greiner Bio-One) and incubated for 30–60 min at RT.

Imaging was performed with either a Zeiss LSM700 confocal microscope with 20× magnification or with a Zeiss LSM800 confocal microscope with 10× magnification.

Quantification of microscopy-based protein–protein interaction assays

From images acquired from a Zeiss LSM700 confocal microscope, the quantification of fluorescence was performed in ImageJ (Schindelin *et al*, 2012) by drawing a line across each bead and taking the maximum gray value along the line. The maximum gray value for any given pixel represents the fluorescence intensity.

For images acquired from a Zeiss LSM800 confocal microscope, we used a custom Fiji Macro. Within this workflow, a pretrained model was created for the deep learning application "Stardist" (https://imagej.net/plugins/stardist; preprint: Schmidt *et al*, 2018). This model was based on a manually annotated training set, using the fluorescently labeled beads as a basis for creating the ground truth annotations and then performing the training on the brightfield channel. Out-of-focus beads were rejected in this step and therefore excluded from the training. After applying the deep learning-based segmentation, the regions were reduced to a ring around the edge of the beads. Beads on image borders were excluded from the analysis. In the end, the mean fluorescent intensities were exported out and used for quantification.

For each method, the fluorescence intensity was normalized against the mean of the control condition.

Fiji macro and agarose bead model for automatic quantification are available in Appendix Data S2.

Mass spectrometry measurements

Proteins were buffer exchanged into ammonium acetate using BioRad Micro Bio-Spin 6 Columns. Native mass spectrometry experiments were carried out on a Synapt G2Si instrument (Waters, Manchester, UK) with a nanoelectrospray ionization source (nESI). Mass calibration was performed by a separate infusion of NaI cluster ions. Solutions were ionized from a thin-walled borosilicate glass capillary (i.d. 0.78 mm, o.d. 1.0 mm, Sutter Instrument Co., Novato, CA, USA) pulled in-house to nESI tip with a Flaming/Brown micropipette puller (Sutter Instrument Co., Novato, CA, USA). A potential of 0.8 kV was applied to the solution via a thin platinum wire (diameter 0.125 mm, Goodfellow, Huntingdon, UK). The following instrument parameters were used: capillary voltage 0.8 kV, sample cone voltage 40 V, source offset 60 V, source

© 2023 The Authors

temperature 40°C, trap collision energy 4.0 V, and trap gas 3 ml/min. Data were processed using Masslynx V4.2 and OriginPro 2021.

Isothermal titration calorimetry (ITC)

All experiments were carried out at 25°C in 50 mM sodium phosphate buffer pH 7.0, 100 mM NaCl, using the PEAQ-ITC Automated (Malvern Panalytical Ltd). For protein–protein interactions, the calorimetric cell was filled with either GABARAP or HsUFM1 and titrated with either HsC53 wt, HsC53 sAIM, HsC53 cAIM, or HsUBA5 LIR. A single injection of 0.4 µl of HsC53 or AtC53 IDRs (not taken into account) was followed by 18 injections of 2 µl each. Injections were made at 150-s intervals with a duration of 4 s and a stirring speed of 750 rpm. The reference power was set to 10 µcal/s; the feedback mode was set to high. The raw titration data were integrated with NITPIC (Keller *et al*, 2012), globally fitted with SEDPHAT (Zhao *et al*, 2015), and plotted with GUSSI (Brautigam, 2015).

Fluorescence anisotropy

For fluorescence anisotropy measurements, all peptides were *in-house* synthetized and HPLC purified attached to 5(6)-Carboxytetramethylrhodamine (TAMRA) to its N-terminal. Binding affinities of ATG8A or AtUFM1 to TAMRA-labeled peptides were measured by the change in fluorescence anisotropy in an FS5 Spectrofluorometer (Edinburgh Instruments) with excitation at 550 nm (slit width 5 nm) and emission at 580 nm (slit width 5 nm). Increasing concentrations of ATG8A or AtUFM1 (250 nM-320 µM) were added to 50 nM fluorescently labeled peptide in a total reaction volume of 20 µl. Measurements were performed in 1.5 × 1.5 mm Ultra-Micro QS Cuvettes after incubation of peptide with ATG8A or AtUFM1 for 10 min at 4°C. Binding curves were fitted using Prism Software (GraphPad).

NMR spectroscopy

All NMR spectroscopy measurements were performed using Bruker AVIII 600 MHz or Avance 800 MHz spectrometers at 25°C. The data were processed using TopSpin 3.2 (Bruker) and NMRPipe (Delaglio *et al*, 1995) and analyzed using CcpNmr Analysis (Skinner *et al*, 2016).

Sequence-specific backbone assignments of AtC53 IDR were achieved using 2D ^{1}H-^{15}N HSQC, 3D HNCA, 3D CBCACONH, 3D HNCACB, 3D HNCO, and 3D HNCACO, including 70 residues of 75 nonproline residues (93%). NMR titrations were performed by adding unlabeled protein (75–300 µM) to 100 µM of ^{15}N single-labeled protein in 50 mM sodium phosphate (pH 7.0), 100 mM NaCl and 10% (v/v) D_2O and monitored by two-dimensional ^{1}H-^{15}N HSQC.

Statistical analysis

All statistical analysis was performed using R Statistical Software (version 4.1.2; R Foundation for Statistical Computing, Vienna, Austria; R Core Team, 2021). Statistical significance of differences between two experimental groups was assessed with a two-tailed unpaired two-samples *t*-test if the two groups were normally distributed (Shapiro–Wilk test) and their variances were equal (F-test). If the groups were normally distributed, the variances were not equal a two-samples Welch *t*-test was performed. If the groups were not normally distributed, an unpaired two-samples Wilcoxon test with continuity correction was performed. Differences between two data sets were considered significant at $P < 0.05$ (*); $P < 0.01$ (**); $P < 0.001$ (***). *P*-value > 0.05 (ns, not significant). Exact sample number, mean, median standard deviation, and *P*-values for each statistical test are provided in Appendix Data S1.

Data availability

The mass spectrometry proteomics data have been deposited to the ProteomeXchange Consortium via the PRIDE partner repository with the dataset identifier PXD038157: http://www.ebi.ac.uk/pride/archive/projects/PXD038157. All the raw data are available via https://doi.org/10.5281/zenodo.7313984.

Expanded View for this article is available online.

Acknowledgements

We thank Vienna Biocenter Core Facilities (VBCF) Protein Chemistry, Biooptics, Plant Sciences, Molecular Biology, and Protein Technologies. We acknowledge funding from Austrian Academy of Sciences, Austrian Science Fund (FWF, P32355, P34944), Austrian Science Fund (FWF-SFB F79), Vienna Science and Technology Fund (WWTF, LS17-047, LS21-009), European Research Council Grant (Project number: 101043370), Marie Curie Fellowship to NZ (Project number: 101028611). RB acknowledges the support of a UKRI Future Leaders Fellowship (Grant Reference MR/T020970/1) and a Chancellor's Fellowship from the University of Strathclyde. NATI was supported by a Junior Research Fellowship from Merton College, Oxford. The IMP is supported by Boehringer Ingelheim. We thank J. Matthew Watson and members of the Dagdas lab for the critical reading and editing of the manuscript. We are thankful to Max Perutz Labs NMR facility and Georg Kontaxis for the NMR measurements. We thank Mathias Madalinski and Zsuzsanna Muhari-Portik for peptide synthesis. We also thank Orla Dunne, Arthur Sedivy, and David Drechsel for help in protein purification and characterization experiments.

Author contributions

Lorenzo Picchianti: Conceptualization; data curation; formal analysis; validation; investigation; visualization; methodology; writing—original draft; writing—review and editing. **Víctor Sánchez de Medina Hernández:** Data curation; formal analysis; validation; investigation; visualization; methodology; writing—original draft; writing—review and editing. **Ni Zhan:** Resources; data curation; validation; investigation; visualization; methodology; writing—original draft; writing—review and editing. **Nicholas A Irwin:** Conceptualization; resources; data curation; software; formal analysis; supervision; validation; investigation; visualization; methodology; writing—original draft; writing—review and editing. **Roan Groh:** Formal analysis; investigation; methodology; writing—review and editing. **Madlen Stephani:** Conceptualization; resources; formal analysis; investigation; methodology. **Harald Hornegger:** Investigation; visualization; methodology. **Rebecca Beveridge:** Data curation; formal analysis; investigation; methodology; writing—original draft. **Justyna Sawa-Makarska:** Resources; supervision; investigation. **Thomas Lendl:** Resources; software; supervision. **Nenad Grujic:** Resources; investigation. **Christin Naumann:** Data curation; formal analysis; investigation; writing—review and editing. **Sascha Martens:** Supervision; funding acquisition. **Thomas A Richards:** Supervision; funding acquisition; writing—original draft. **Tim Clausen:** Supervision; funding acquisition; investigation; writing—original draft. **Silvia Ramundo:** Supervision; funding acquisition; investigation; writing—original draft. **G Elif Karagoz:** Conceptualization;

 © 2023 The Authors

data curation; formal analysis; supervision; funding acquisition; visualization; writing—original draft; project administration; writing—review and editing. **Yasin Dagdas:** Conceptualization; supervision; funding acquisition; visualization; writing—original draft; project administration; writing—review and editing.

Disclosure and competing interests statement

The authors declare that they have no conflit of interest.

References

Ashkenazy H, Abadi S, Martz E, Chay O, Mayrose I, Pupko T, Ben-Tal N (2016) ConSurf 2016: an improved methodology to estimate and visualize evolutionary conservation in macromolecules. *Nucleic Acids Res* 44: W344 – W350

Balce DR, Wang Y-T, McAllaster MR, Dunlap BF, Orvedahl A, Hykes BL, Droit L, Handley SA, Wilen CB, Doench JG *et al* (2021) UFMylation inhibits the proinflammatory capacity of interferon-γ–activated macrophages. *Proc Natl Acad Sci U S A* 118: e2011763118

Banerjee S, Kumar M, Wiener R (2020) Decrypting UFMylation: how proteins are modified with UFM1. *Biomolecules* 10: 1442

Bechtold N, Pelletier G (1998) In planta agrobacterium-mediated transformation of adult Arabidopsis thaliana plants by vacuum infiltration. *Methods Mol Biol* 82: 259 – 266

Birgisdottir ÅB, Lamark T, Johansen T (2013) The LIR motif - crucial for selective autophagy. *J Cell Sci* 126: 3237 – 3247

Boeckmann B, Bairoch A, Apweiler R, Blatter MC, Estreicher A, Gasteiger E, Martin MJ, Michoud K, O'Donovan C, Phan I *et al* (2003) The SWISS-PROT protein knowledgebase and its supplement TrEMBL in 2003. *Nucleic Acids Res* 31: 365 – 370

Brandizzi F (2021) Maintaining the structural and functional homeostasis of the plant endoplasmic reticulum. *Dev Cell* 56: 919 – 932

Brautigam CA (2015) Calculations and publication-quality illustrations for analytical ultracentrifugation data. *Methods Enzymol* 562: 109 – 133

Buchfink B, Xie C, Huson DH (2014) Fast and sensitive protein alignment using DIAMOND. *Nat Methods* 12: 59 – 60

Burki F, Roger AJ, Brown MW, Simpson AGB (2020) The new tree of eukaryotes. *Trends Ecol Evol* 35: 43 – 55

Capella-Gutiérrez S, Silla-Martínez JM, Gabaldón T (2009) trimAl: a tool for automated alignment trimming in large-scale phylogenetic analyses. *Bioinformatics* 25: 1972 – 1973

Cox M, Dekker N, Boelens R, Verrijzer CP, van der Vliet PC, Kaptein R (1993) NMR studies of the POU-specific DNA-binding domain of Oct-1: sequential proton and nitrogen-15 assignments and secondary structure. *Biochemistry* 32: 6032 – 6040

Cox J, Hein MY, Luber CA, Paron I, Nagaraj N, Mann M (2014) Accurate proteome-wide label-free quantification by delayed normalization and maximal peptide ratio extraction, termed MaxLFQ. *Mol Cell Proteomics* 13: 2513 – 2526

Daniel J, Liebau E (2014) The Ufm1 cascade. *Cell* 3: 627 – 638

Delaglio F, Grzesiek S, Vuister GW, Zhu G, Pfeifer J, Bax A (1995) NMRPipe: a multidimensional spectral processing system based on UNIX pipes. *J Biomol NMR* 6: 277 – 293

Dikic I (2017) Proteasomal and Autophagic degradation systems. *Annu Rev Biochem* 86: 193 – 224

Dikic I, Elazar Z (2018) Mechanism and medical implications of mammalian autophagy. *Nat Rev Mol Cell Biol* 19: 349 – 364

Doblmann J, Dusberger F, Imre R, Hudecz O, Stanek F, Mechtler K, Dürnberger G (2019) apQuant: accurate label-free quantification by quality filtering. *J Proteome Res* 18: 535 – 541

Dorfer V, Pichler P, Stranzl T, Stadlmann J, Taus T, Winkler S, Mechtler K (2014) MS Amanda, a universal identification algorithm optimized for high accuracy tandem mass spectra. *J Proteome Res* 13: 3679 – 3684

Eck F, Kaulich M, Behrends C (2020) ACSL3 is a novel GABARAPL2 interactor that links ufmylation and lipid droplet biogenesis. *bioRxiv* https://doi.org/10.1101/2020.01.01.892521 [PREPRINT]

Edgar RC (2004) MUSCLE: a multiple sequence alignment method with reduced time and space complexity. *BMC Bioinformatics* 5: 113

El-Gebali S, Mistry J, Bateman A, Eddy SR, Luciani A, Potter SC, Qureshi M, Richardson LJ, Salazar GA, Smart A *et al* (2019) The Pfam protein families database in 2019. *Nucleic Acids Res* 47: D427 – D432

Federhen S (2012) The NCBI taxonomy database. *Nucleic Acids Res* 40: 136 – 143

Gerakis Y, Quintero M, Li H, Hetz C (2019) The UFMylation system in proteostasis and beyond. *Trends Cell Biol* 29: 974 – 986

Grau-Bove X, Sebe-Pedros A, Ruiz-Trillo I (2015) The eukaryotic ancestor had a complex ubiquitin signaling system of archaeal origin. *Mol Biol Evol* 32: 726 – 739

Haas BJ, Papanicolaou A, Yassour M, Grabherr M, Blood PD, Bowden J, Couger MB, Eccles D, Li B, Lieber M *et al* (2013) De novo transcript sequence reconstruction from RNA-seq using the trinity platform for reference generation and analysis. *Nat Protoc* 8: 1494 – 1512

Habisov S, Huber J, Ichimura Y, Akutsu M, Rogova N, Loehr F, McEwan DG, Johansen T, Dikic I, Doetsch V *et al* (2016) Structural and functional analysis of a novel interaction motif within UFM1-activating enzyme 5 (UBA5) required for binding to ubiquitin-like proteins and Ufmylation. *J Biol Chem* 291: 9025 – 9041

Huber J, Obata M, Gruber J, Akutsu M, Löhr F, Rogova N, Güntert P, Dikic I, Kirkin V, Komatsu M *et al* (2020) An atypical LIR motif within UBA5 (ubiquitin like modifier activating enzyme 5) interacts with GABARAP proteins and mediates membrane localization of UBA5. *Autophagy* 16: 256 – 270

Ishimura R, El-Gowily AH, Noshiro D, Komatsu-Hirota S, Ono Y, Shindo M, Hatta T, Abe M, Uemura T, Lee-Okada H-C *et al* (2022) The UFM1 system regulates ER-phagy through the ufmylation of CYB5R3. *Nat Commun* 13: 7857

Johansen T, Lamark T (2020) Selective autophagy: ATG8 family proteins, LIR motifs and cargo receptors. *J Mol Biol* 432: 80 – 103

Käll L, Canterbury JD, Weston J, Noble WS, MacCoss MJ (2007) Semi-supervised learning for peptide identification from shotgun proteomics datasets. *Nat Methods* 4: 923 – 925

Kalyaanamoorthy S, Minh BQ, Wong TKF, Von Haeseler A, Jermiin LS (2017) ModelFinder: fast model selection for accurate phylogenetic estimates. *Nat Methods* 14: 587 – 589

Karagöz GE, Acosta-Alvear D, Walter P (2019) The unfolded protein response: detecting and responding to fluctuations in the protein-folding capacity of the endoplasmic reticulum. *Cold Spring Harb Perspect Biol* 11: a033886

Katoh K, Standley DM (2013) MAFFT multiple sequence alignment software version 7: improvements in performance and usability. *Mol Biol Evol* 30: 772 – 780

Keller S, Vargas C, Zhao H, Piszczek G, Brautigam CA, Schuck P (2012) High-precision isothermal titration calorimetry with automated peak-shape analysis. *Anal Chem* 84: 5066 – 5073

Kirkin V (2020) History of the selective autophagy research: how did it begin and where does it stand today? *J Mol Biol* 432: 3 – 27

© 2023 The Authors

Kirkin V, Rogov VV (2019) A diversity of selective autophagy receptors determines the specificity of the autophagy pathway. *Mol Cell* 76: 268 – 285

Klionsky DJ, Petroni G, Amaravadi RK, Baehrecke EH, Ballabio A, Boya P, Bravo-San Pedro JM, Cadwell K, Cecconi F, Choi AMK *et al* (2021) Autophagy in major human diseases. *EMBO J* 40: e108863

Kolde R, Kolde MR (2018) Package 'pheatmap'. R Package 1

Kouno T, Miura K, Kanematsu T, Shirakawa M, Hirata M, Kawano K (2002) Letter to the editor: 1H, 13C and 15N resonance assignments of GABARAP, GABAA receptor associated protein. *J Biomol NMR* 22: 97 – 98

Kulsuptrakul J, Wang R, Meyers NL, Ott M, Puschnik AS (2020) A genome-wide CRISPR screen identifies UFMylation and TRAMP-like complexes as host factors required for hepatitis A virus infection. *Cell Rep* 34: 108859

Kumar M, Padala P, Fahoum J, Hassouna F, Tsaban T, Zoltsman G, Banerjee S, Cohen-Kfir E, Dessau M, Rosenzweig R *et al* (2021) Structural basis for UFM1 transfer from UBA5 to UFC1. *Nat Commun* 12: 1 – 13

Lampropoulos A, Sutikovic Z, Wenzl C, Maegele I, Lohmann JU, Forner J (2013) GreenGate - a novel, versatile, and efficient cloning system for plant Transgenesis. *PLoS One* 8: e83043

Larsson A (2014) AliView: a fast and lightweight alignment viewer and editor for large datasets. *Bioinformatics* 30: 3276 – 3278

Lee L, Perez Oliva AB, Martinez-Balsalobre E, Churikov D, Peter J, Rahmouni D, Audoly G, Azzoni V, Audebert S, Camoin L *et al* (2021) UFMylation of MRE11 is essential for telomere length maintenance and hematopoietic stem cell survival. *Sci Adv* 7: eabc7371

Letunic I, Bork P (2021) Interactive tree of life (iTOL) v5: an online tool for phylogenetic tree display and annotation. *Nucleic Acids Res* 49: W293 – W296

Li X, Zhang R, Patena W, Gang SS, Blum SR, Ivanova N, Yue R, Robertson JM, Lefebvre PA, Fitz-Gibbon ST *et al* (2016) An indexed, mapped mutant library enables reverse genetics studies of biological processes in *Chlamydomonas reinhardtii*. *Plant Cell* 28: 367 – 387

Li X, Patena W, Fauser F, Jinkerson RE, Saroussi S, Meyer MT, Ivanova N, Robertson JM, Yue R, Zhang R *et al* (2019) A genome-wide algal mutant library and functional screen identifies genes required for eukaryotic photosynthesis. *Nat Genet* 51: 627 – 635

Liang JR, Lingeman E, Luong T, Ahmed S, Muhar M, Nguyen T, Olzmann JA, Corn JE (2020) A genome-wide ER-phagy screen highlights key roles of mitochondrial metabolism and ER-resident UFMylation. *Cell* 180: 1160 – 1177.e20

Liu J, Guan D, Dong M, Yang J, Wei H, Liang Q, Song L, Xu L, Bai J, Liu C *et al* (2020) UFMylation maintains tumour suppressor p53 stability by antagonizing its ubiquitination. *Nat Cell Biol* 22: 1056 – 1063

Marley J, Lu M, Bracken C (2001) A method for efficient isotopic labeling of recombinant proteins. *J Biomol NMR* 20: 71 – 75

Marshall RS, Vierstra RD (2018) Autophagy: the master of bulk and selective recycling. *Annu Rev Plant Biol* 69: 173 – 208

Minh BQ, Schmidt HA, Chernomor O, Schrempf D, Woodhams MD, Von Haeseler A, Lanfear R, Teeling E (2020) IQ-TREE 2: new models and efficient methods for phylogenetic inference in the genomic era. *Mol Biol Evol* 37: 1530 – 1534

Mistry J, Finn RD, Eddy SR, Bateman A, Punta M (2013) Challenges in homology search: HMMER3 and convergent evolution of coiled-coil regions. *Nucleic Acids Res* 41: e121

Mizushima N (2018) A brief history of autophagy from cell biology to physiology and disease. *Nat Cell Biol* 20: 521 – 527

Noda NN, Ohsumi Y, Inagaki F (2010) Atg8-family interacting motif crucial for selective autophagy. *FEBS Lett* 584: 1379 – 1385

Oweis W, Padala P, Hassouna F, Cohen-Kfir E, Gibbs DR, Todd EA, Berndsen CE, Wiener R (2016) Trans-binding mechanism of ubiquitin-like protein activation revealed by a UBA5-UFM1 complex. *Cell Rep* 16: 3113 – 3120

Padala P, Oweis W, Mashahreh B, Soudah N, Cohen-Kfir E, Todd EA, Berndsen CE, Wiener R (2017) Novel insights into the interaction of UBA5 with UFM1 via a UFM1-interacting sequence. *Sci Rep* 7: 508

Penev PI, Alvarez-Carreño C, Smith E, Petrov AS, Williams LD (2021) TwinCons: conservation score for uncovering deep sequence similarity and divergence. *PLoS Comput Biol* 17: e1009541

Perez-Riverol Y, Csordas A, Bai J, Bernal-Llinares M, Hewapathirana S, Kundu DJ, Inuganti A, Griss J, Mayer G, Eisenacher M *et al* (2019) The PRIDE database and related tools and resources in 2019: improving support for quantification data. *Nucleic Acids Res* 47: D442 – D450

Perlaza K, Toutkoushian H, Boone M, Lam M, Iwai M, Jonikas MC, Walter P, Ramundo S (2019) The mars1 kinase confers photoprotection through signaling in the chloroplast unfolded protein response. *Elife* 8: e49577

Peter JJ, Magnussen HM, DaRosa PA, Millrine D, Matthews SP, Lamoliatte F, Sundaramoorthy R, Kopito RR, Kulathu Y (2022) A non-canonical scaffold-type E3 ligase complex mediates protein UFMylation. *EMBO J* 41: e111015

Pohl C, Dikic I (2019) Cellular quality control by the ubiquitin-proteasome system and autophagy. *Science* 366: 818 – 822

Qin B, Yu J, Nowsheen S, Wang M, Tu X, Liu T, Li H, Wang L, Lou Z (2019) UFL1 promotes histone H4 ufmylation and ATM activation. *Nat Commun* 10: 1242

R Core Team (2021) *R: a language and environment for statistical computing*. Vienna, Austria: R Foundation for Statistical Computing

Rambaut A (2012) FigTree, a graphical viewer of phylogenetic trees. http://tree.bio.ed.ac.uk/software/figtree

Revelle W, Revelle MW (2015) Package 'psych'. The comprehensive R archive network 337: 338

Rozewicki J, Li S, Amada KM, Standley DM, Katoh K (2019) MAFFT-DASH: integrated protein sequence and structural alignment. *Nucleic Acids Res* 47: W5 – W10

Sasakawa H, Sakata E, Yamaguchi Y, Komatsu M, Tatsumi K, Kominami E, Tanaka K, Kato K (2006) Solution structure and dynamics of Ufm1, a ubiquitin-fold modifier 1. *Biochem Biophys Res Commun* 343: 21 – 26

Schindelin J, Arganda-Carreras I, Frise E, Kaynig V, Longair M, Pietzsch T, Preibisch S, Rueden C, Saalfeld S, Schmid B *et al* (2012) Fiji: an open-source platform for biological-image analysis. *Nat Methods* 9: 676 – 682

Schmidt U, Weigert M, Broaddus C, Myers G (2018) Cell detection with star-convex polygons. *arXiv* https://doi.org/10.48550/arXiv.1806.03535 [PREPRINT]

Schwanhäusser B, Busse D, Li N, Dittmar G, Schuchhardt J, Wolf J, Chen W, Selbach M (2011) Global quantification of mammalian gene expression control. *Nature* 473: 337 – 342

Sette M, Spurio R, van Tilborg P, Gualerzi CO, Boelens R (1999) Identification of the ribosome binding sites of translation initiation factor IF3 by multidimensional heteronuclear NMR spectroscopy. *RNA* 5: 82 – 92

Shevchenko A, Tomas H, Havlis J, Olsen JV, Mann M (2006) In-gel digestion for mass spectrometric characterization of proteins and proteomes. *Nat Protoc* 1: 2856 – 2560

Skinner SP, Fogh RH, Boucher W, Ragan TJ, Mureddu LG, Vuister GW (2016) CcpNmr AnalysisAssign: a flexible platform for integrated NMR analysis. *J Biomol NMR* 66: 111 – 124

Slater GSC, Birney E (2005) Automated generation of heuristics for biological sequence comparison. *BMC Bioinformatics* 6: 31

© 2023 The Authors

Stephani M, Dagdas Y (2020) Plant selective autophagy—still an uncharted territory with a lot of hidden gems. *J Mol Biol* 432: 63 – 79

Stephani M, Picchianti L, Gajic A, Beveridge R, Skarwan E, Sanchez de Medina Hernandez V, Mohseni A, Clavel M, Zeng Y, Naumann C *et al* (2020) A cross-kingdom conserved er-phagy receptor maintains endoplasmic reticulum homeostasis during stress. *Elife* 9: e58396

Sun Z, Brodsky JL (2019) Protein quality control in the secretory pathway. *J Cell Biol* 218: 3171 – 3187

Taus T, Köcher T, Pichler P, Paschke C, Schmidt A, Henrich C, Mechtler K (2011) Universal and confident phosphorylation site localization using phosphoRS. *J Proteome Res* 10: 5354 – 5362

Tsaban T, Stupp D, Sherill-Rofe D, Bloch I, Sharon E, Schueler-Furman O, Wiener R, Tabach Y (2021) CladeOScope: functional interactions through the prism of clade-wise co-evolution. *NAR Genom Bioinform* 3: lqab024

Turco E, Witt M, Abert C, Bock-Bierbaum T, Su M-Y, Trapannone R, Sztacho M, Danieli A, Shi X, Zaffagnini G *et al* (2019) FIP200 claw domain binding to p62 promotes autophagosome formation at ubiquitin condensates. *Mol Cell* 74: 330 – 346.e11

Turco E, Fracchiolla D, Martens S (2020) Recruitment and activation of the ULK1/Atg1 kinase complex in selective autophagy. *J Mol Biol* 432: 123 – 134

Walczak CP, Leto DE, Zhang L, Riepe C, Muller RY, DaRosa PA, Ingolia NT, Elias JE, Kopito RR (2019) Ribosomal protein RPL26 is the principal target of UFMylation. *Proc Natl Acad Sci U S A* 116: 1299 – 1308

Wang L, Xu Y, Rogers H, Saidi L, Noguchi CT, Li H, Yewdell JW, Guydosh NR, Ye Y (2020) UFMylation of RPL26 links translocation-associated quality control to endoplasmic reticulum protein homeostasis. *Cell Res* 30: 5 – 20

Wheeler TJ, Clements J, Finn RD (2014) Skylign: a tool for creating informative, interactive logos representing sequence alignments and profile hidden Markov models. *BMC Bioinformatics* 15: 7

Wolf YI, Koonin EV (2013) Genome reduction as the dominant mode of evolution. *Bioessays* 35: 829 – 837

Wüthrich K (2003) NMR studies of structure and function of biological macromolecules (Nobel lecture). *J Biomol NMR* 27: 13 – 39

Zaremba-Niedzwiedzka K, Caceres EF, Saw JH, Bäckström D, Juzokaite L, Vancaester E, Seitz KW, Anantharaman K, Starnawski P, Kjeldsen KU *et al* (2017) Asgard archaea illuminate the origin of eukaryotic cellular complexity. *Nature* 541: 353 – 358

Zhao H, Piszczek G, Schuck P (2015) SEDPHAT – a platform for global ITC analysis and global multi-method analysis of molecular interactions. *Methods* 76: 137 – 148

Zhou J, Liang Q, Dong M, Ma X, Jin Y, Guan D, Liu J, Wang M, Cong YS (2022) Optimized protocol to detect protein UFMylation in cells and *in vitro* via immunoblotting. *STAR Protoc* 3: 101074

Zientara-Rytter K, Subramani S (2020) Mechanistic insights into the role of Atg11 in selective autophagy. *J Mol Biol* 432: 104 – 122

Zuiderweg ERP (2002) Mapping protein-protein interactions in solution by NMR spectroscopy. *Biochemistry* 41: 1 – 7

License: This is an open access article under the terms of the Creative Commons Attribution License, which permits use, distribution and reproduction in any medium, provided the original work is properly cited.

© 2023 The Authors

Expanded View Figures

Figure EV1. Expanded version of the tree depicted in Fig 1A, displaying the presence and absence of UFMylation proteins across the eukaryotic taxa.

A–I The tree has been divided into eukaryotic supergroups including the Opisthokonta (A), Amoebozoa (B), Haptophyta and SAR (C), Archaeplastida (D), Discoba (E), Metamonada (F), CRuMs (G) and Apusozoa (H). (I) Pairwise binary Phi correlation of UFMylation proteins distributions.

© 2023 The Authors

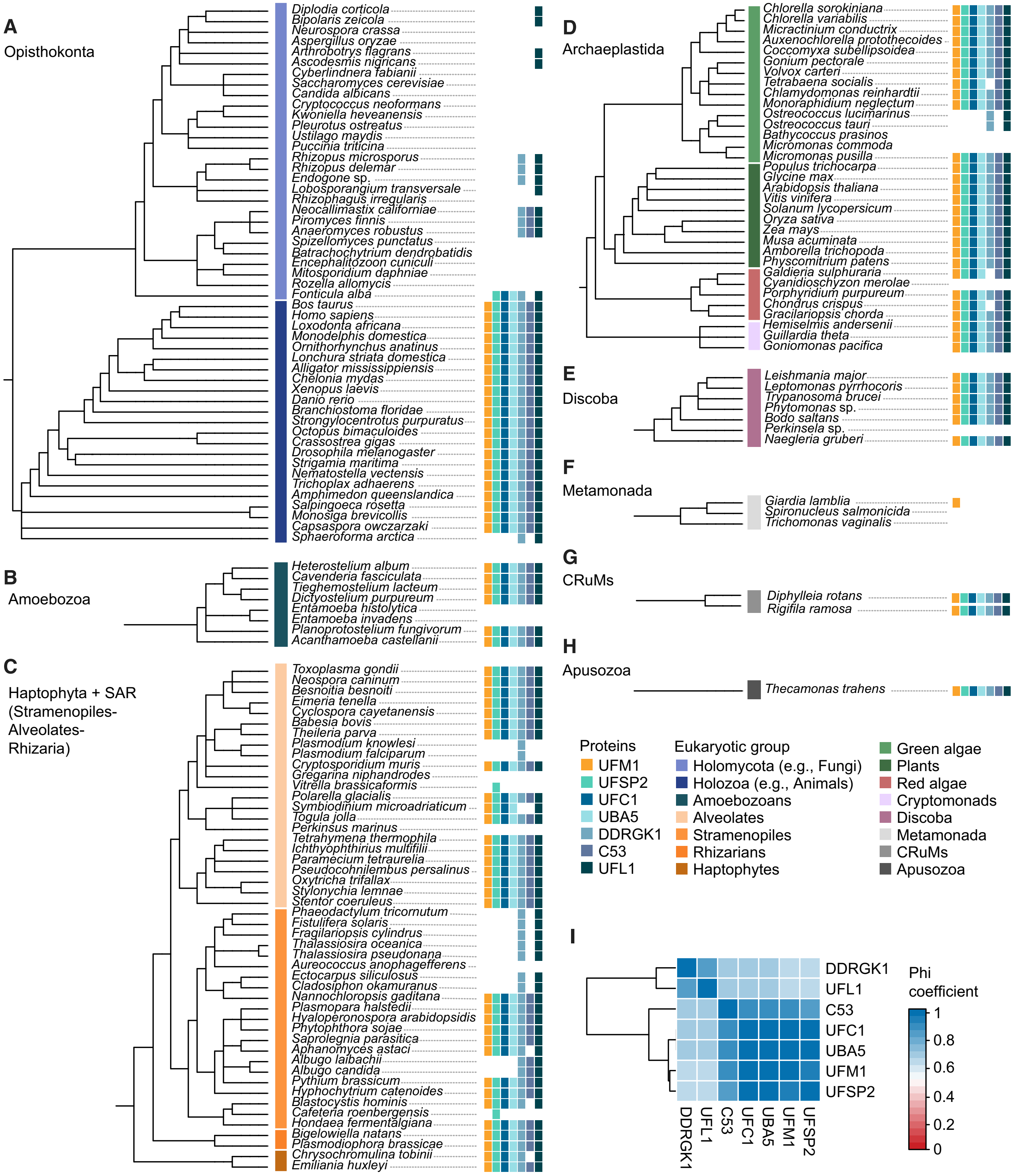
A
Opisthokonta
Diplodia corticola
Bipolaris zeicola
Neurospora crassa
Aspergillus oryzae
Arthrobotrys flagrans
Ascodesmis nigricans
Cyberlindnera fabianii
Saccharomyces cerevisiae
Candida albicans
Cryptococcus neoformans
Kwoniella heveanensis
Pleurotus ostreatus
Ustilago maydis
Puccinia triticina
Rhizopus microsporus
Rhizopus delemar
Endogone sp.
Lobosporangium transversale
Rhizophagus irregularis
Neocallimastix californiae
Piromyces finnis
Anaeromyces robustus
Spizellomyces punctatus
Batrachochytrium dendrobatidis
Encephalitozoon cuniculi
Mitosporidium daphniae
Rozella allomycis
Fonticula alba
Bos taurus
Homo sapiens
Loxodonta africana
Monodelphis domestica
Ornithorhynchus anatinus
Lonchura striata domestica
Alligator mississippiensis
Chelonia mydas
Xenopus laevis
Danio rerio
Branchiostoma floridae
Strongylocentrotus purpuratus
Octopus bimaculoides
Crassostrea gigas
Drosophila melanogaster
Strigamia maritima
Nematostella vectensis
Trichoplax adhaerens
Amphimedon queenslandica
Salpingoeca rosetta
Monosiga brevicollis
Capsaspora owczarzaki
Sphaeroforma arctica
B
Amoebozoa
Heterostelium album
Cavenderia fasciculata
Tieghemostelium lacteum
Dictyostelium purpureum
Entamoeba histolytica
Entamoeba invadens
Planoprotostelium fungivorum
Acanthamoeba castellanii
C
Haptophyta + SAR (Stramenopiles-Alveolates-Rhizaria)
Toxoplasma gondii
Neospora caninum
Besnoitia besnoiti
Eimeria tenella
Cyclospora cayetanensis
Babesia bovis
Theileria parva
Plasmodium knowlesi
Plasmodium falciparum
Cryptosporidium muris
Gregarina niphandrodes
Vitrella brassicaformis
Polarella glacialis
Symbiodinium microadriaticum
Togula jolla
Perkinsus marinus
Tetrahymena thermophila
Ichthyophthirius multifiliis
Paramecium tetraurelia
Pseudocohnilembus persalinus
Oxytricha trifallax
Stylonychia lemnae
Stentor coeruleus
Phaeodactylum tricornutum
Fistulifera solaris
Fragilariopsis cylindrus
Thalassiosira oceanica
Thalassiosira pseudonana
Aureococcus anophagefferens
Ectocarpus siliculosus
Cladosiphon okamuranus
Nannochloropsis gaditana
Plasmopara halstedii
Hyaloperonospora arabidopsidis
Phytophthora sojae
Saprolegnia parasitica
Aphanomyces astaci
Albugo laibachii
Albugo candida
Pythium brassicum
Hyphochytrium catenoides
Blastocystis hominis
Cafeteria roenbergensis
Hondaea fermentalgiana
Bigelowiella natans
Plasmodiophora brassicae
Chrysochromulina tobinii
Emiliania huxleyi
D
Archaeplastida
Chlorella sorokiniana
Chlorella variabilis
Micractinium conductrix
Auxenochlorella protothecoides
Coccomyxa subellipsoidea
Gonium pectorale
Volvox carteri
Tetrabaena socialis
Chlamydomonas reinhardtii
Monoraphidium neglectum
Ostreococcus lucimarinus
Ostreococcus tauri
Bathycoccus prasinos
Micromonas commoda
Micromonas pusilla
Populus trichocarpa
Glycine max
Arabidopsis thaliana
Vitis vinifera
Solanum lycopersicum
Oryza sativa
Zea mays
Musa acuminata
Amborella trichopoda
Physcomitrium patens
Galdieria sulphuraria
Cyanidioschyzon merolae
Porphyridium purpureum
Chondrus crispus
Gracilariopsis chorda
Hemiselmis andersenii
Guillardia theta
Goniomonas pacifica
E
Discoba
Leishmania major
Leptomonas pyrrhocoris
Trypanosoma brucei
Phytomonas sp.
Bodo saltans
Perkinsela sp.
Naegleria gruberi
F
Metamonada
Giardia lamblia
Spironucleus salmonicida
Trichomonas vaginalis
G
CRuMs
Diphylleia rotans
Rigifila ramosa
H
Apusozoa
Thecamonas trahens
Proteins
UFM1
UFSP2
UFC1
UBA5
DDRGK1
C53
UFL1
Eukaryotic group
Holomycota (e.g., Fungi)
Holozoa (e.g., Animals)
Amoebozoans
Alveolates
Stramenopiles
Rhizarians
Haptophytes
Green algae
Plants
Red algae
Cryptomonads
Discoba
Metamonada
CRuMs
Apusozoa
I
DDRGK1
UFL1
C53
UFC1
UBA5
UFM1
UFSP2
Phi coefficient
1
0.8
0.6
0.4
0.2
0

Figure EV1.

Figure EV2. C53 orthologous from two TSAR representatives as well as representatives from Haptista, Cryptista, Diplomonada, Amoebozoa, and CRuMs interact with human and Arabidopsis ATG8 and UFM1.

Bacterial lysates containing recombinant protein were mixed and pulled down with glutathione magnetic agarose beads. Input and bound proteins were visualized by immunoblotting with anti-GST and anti-MBP antibodies. *Tetrahymena thermophila* (Tt), *Dictyostelium purpureum* (Dp), *Emiliania huxleyi* (Eh), *Guillardia theta* (Gt), *Trypanosoma brucei* (Tb), *Rigifila ramosa* (Rr) and *Phytophthora sojae* (Ps).

© 2023 The Authors

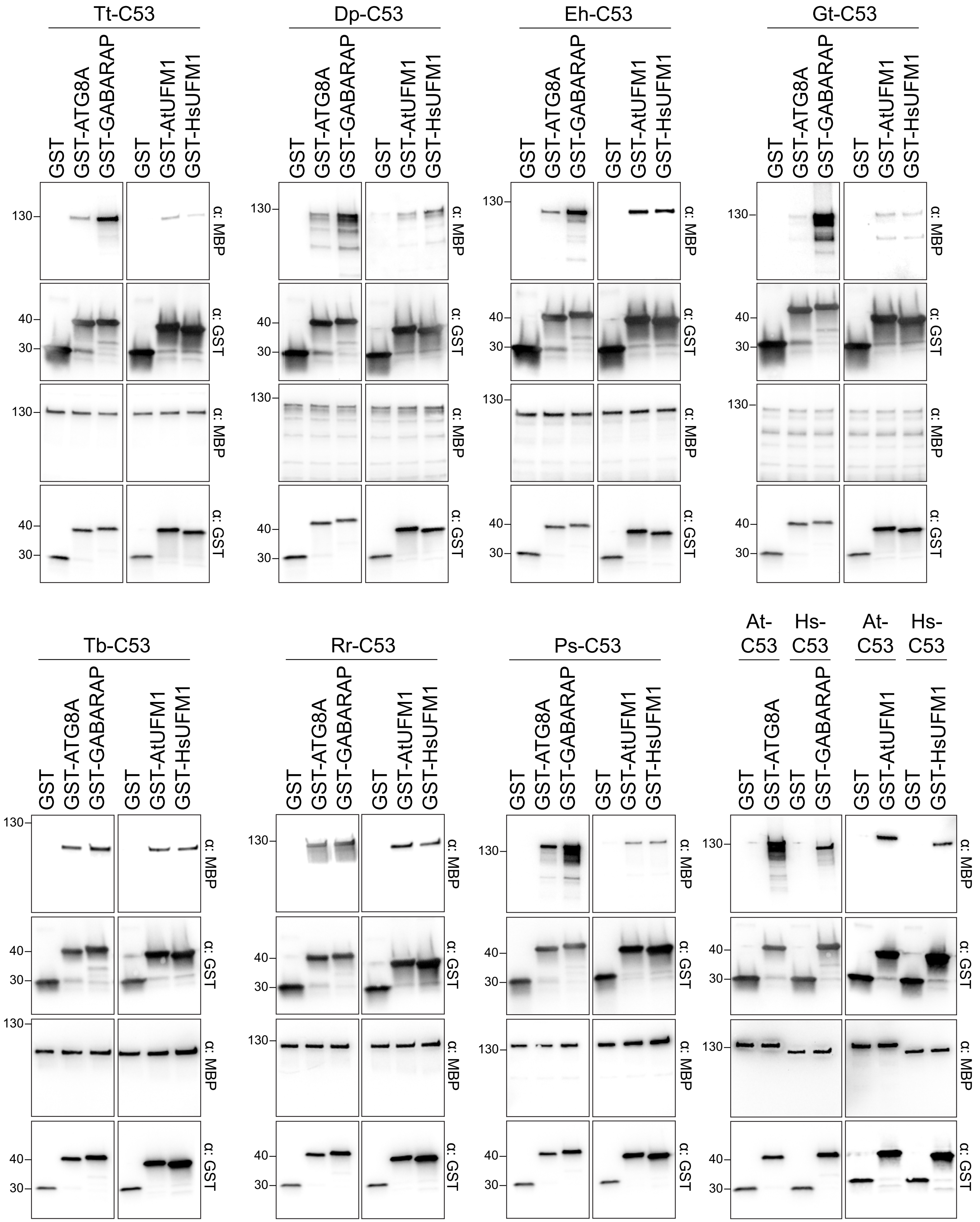
Tt-C53
GST
GST-ATG8A
GST-GABARAP
GST
GST-AtUFM1
GST-HsUFM1
130
40
30
α: MBP
α: GST
Dp-C53
Eh-C53
Gt-C53
Tb-C53
Rr-C53
Ps-C53
At-C53
Hs-C53

Figure EV2.

Figure EV3. Structural characterization of UFM1 binding to C53 IDR using NMR spectroscopy.

A A small number of residues are affected by the addition of HsC53 IDR as shown in the HsUFM1 spectra. Overlaid ^{1}H-^{15}N HSQC spectra of isotope-labeled HsUFM1 (100 μM) in their free (gray) or bound state to 75 μM (blue), 100 μM (green), 200 μM (orange) and 300 μM (red) unlabeled HsC53 IDR. Insets of individual peaks that shift upon binding are shown.

B HsC53 IDR binding to HsUFM1 causes general signal intensity drop in HsUFM1 spectra. Intensity ratio broadening of HsUFM1 (100 μM) in the presence of 75 μM (blue), 100 μM (green), 200 μM (orange) and 300 μM (red) HsC53 IDR.

C Chemical shift perturbations (CSPs) in the HsUFM1 spectrum (gray) upon addition of 75 μM (blue), 100 μM (green), 200 μM (orange) and 300 μM (red) HsC53 IDR. The dashed line represents SD.

D Three-dimensional mapping of residues showing CSP in HsUFM1 NMR spectra upon HsC53 IDR binding. CSPs were mapped on the UFM1 structure (PDB: 1WXS) presented schematically on the left plot and as a surface representation in two projections on the right plot. Residues that are not affected or are slightly ($CSP < 0.025$), intermediately ($0.025 < CSP < 0.04$), or strongly ($CSP > 0.04$) affected by the binding are colored in tan, orange and red, respectively.

E AtC53 IDR binding to AtUFM1 is similar to that of AtUBA5 and involves sAIM1. Overlaid ^{1}H-^{15}N HSQC spectra of isotope-labeled AtUFM1 (100 μM) in their free (gray) or bound state to 100 μM unlabeled AtC53 IDR (red), 100 μM unlabeled AtC53 IDR^{W276A} (yellow) or 200 μM AtUBA5 LIR/UFIM (green). Insets of chemical shift perturbations of individual peaks are shown.

 © 2023 The Authors

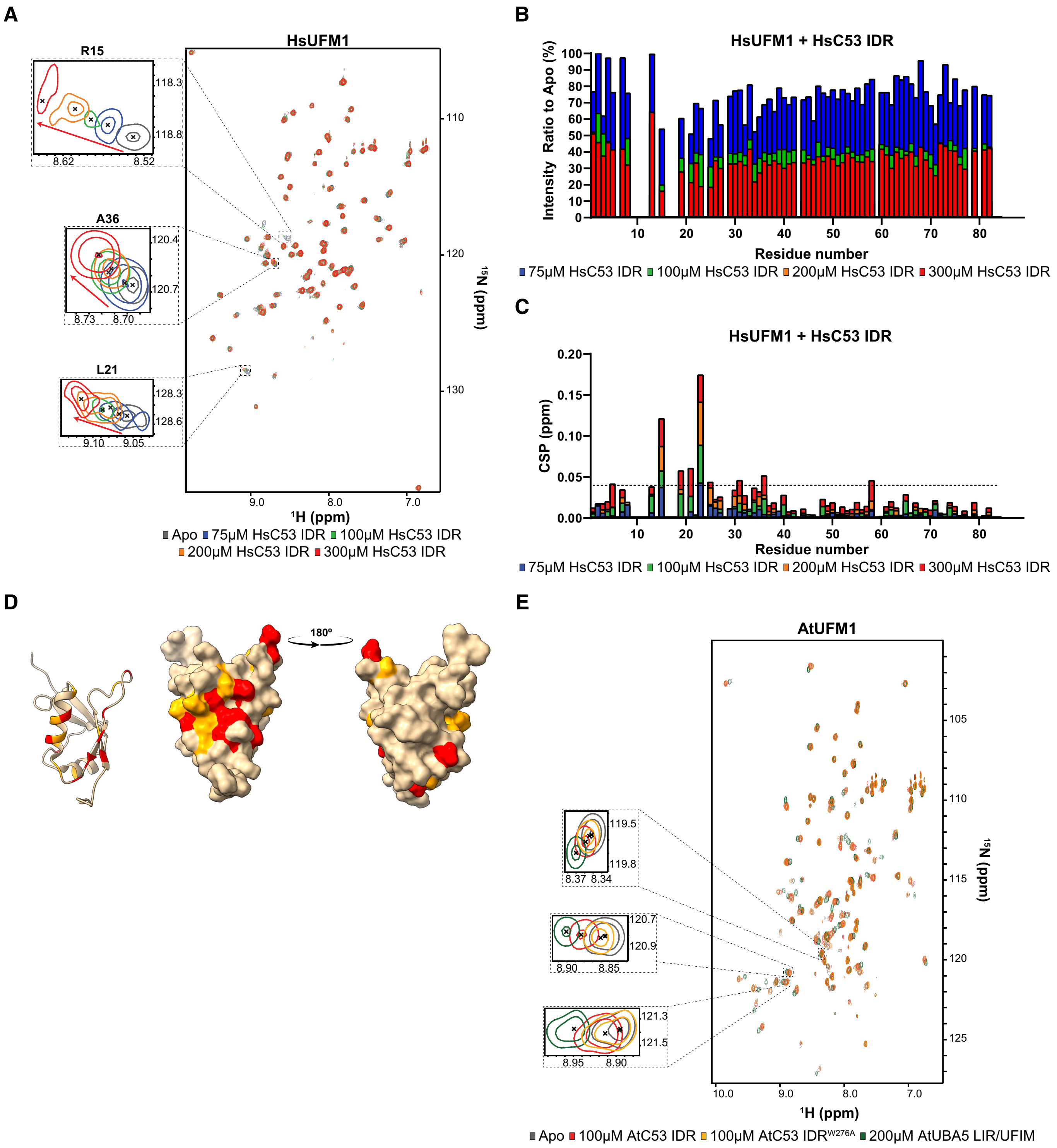

A
HsUFM1
R15
A36
L21
¹H (ppm)
¹⁵N (ppm)
Apo 75µM HsC53 IDR 100µM HsC53 IDR 200µM HsC53 IDR 300µM HsC53 IDR
B
HsUFM1 + HsC53 IDR
Intensity Ratio to Apo (%)
Residue number
75µM HsC53 IDR 100µM HsC53 IDR 200µM HsC53 IDR 300µM HsC53 IDR
C
HsUFM1 + HsC53 IDR
CSP (ppm)
Residue number
75µM HsC53 IDR 100µM HsC53 IDR 200µM HsC53 IDR 300µM HsC53 IDR
D
180°
E
AtUFM1
¹H (ppm)
¹⁵N (ppm)
Apo 100µM AtC53 IDR 100µM AtC53 IDR W276A 200µM AtUBA5 LIR/UFIM

Figure EV3.

Figure EV4. Structural characterization of ATG8 binding to C53 IDR using NMR spectroscopy.

A Addition of HsC53 IDR affects numerous residues in the GABARAP spectra. Overlaid ^{1}H-^{15}N HSQC spectra of isotope-labeled GABARAP (100 μM) in their free (gray) or bound state to 50 μM (blue), 100 μM (green) or 200 μM (orange) unlabeled HsC53 IDR. Insets of individual peaks that shifted upon binding are shown.

B HsC53 IDR binding to GABARAP causes a general signal intensity drop in GABARAP spectra. Intensity ratio broadening of GABARAP (100 μM) in the presence of 50 μM (blue), 100 μM (green) or 200 μM (orange) unlabeled HsC53 IDR. HN resonances for residues that could not be assigned in the bound state are shown as red asterisks.

C NMR chemical shift perturbations (CSP) of GABARAP in the presence of 50 μM (blue), 100 μM (green) or 200 μM (orange) HsC53 IDR. HN resonances for residues that could not be assigned in the bound state are shown as red asterisks. The dashed line represents S.D.

D Three-dimensional mapping of residues showing CSP in GABARAP NMR spectra upon HsC53 IDR binding. CSPs were mapped on the GABARAP structure (PDB: 6HB9) presented schematically on the left plot and as a surface representation in two projections on the right plot. Residues that are not affected or are slightly (CSP < 0.1), intermediately (0.1 < CSP < 0.2), or strongly (CSP > 0.2) affected by the binding are colored in tan, orange and red, respectively. The inset highlights the position of the HP0, HP1 and HP2 hydrophobic pockets in GABARAP.

E AtC53 IDR binding to ATG8 is similar to that of AtUBA5. Overlaid ^{1}H-^{15}N HSQC spectra of isotope-labeled ATG8A (100 μM) in their free (gray) or bound state to 100 μM unlabeled AtC53 IDR (red), 100 μM unlabeled AtC53 IDR^{W276A} (yellow) or 200 μM AtUBA5 LIR/UFIM (green). Insets of chemical shift perturbations of individual peaks are shown.

 © 2023 The Authors

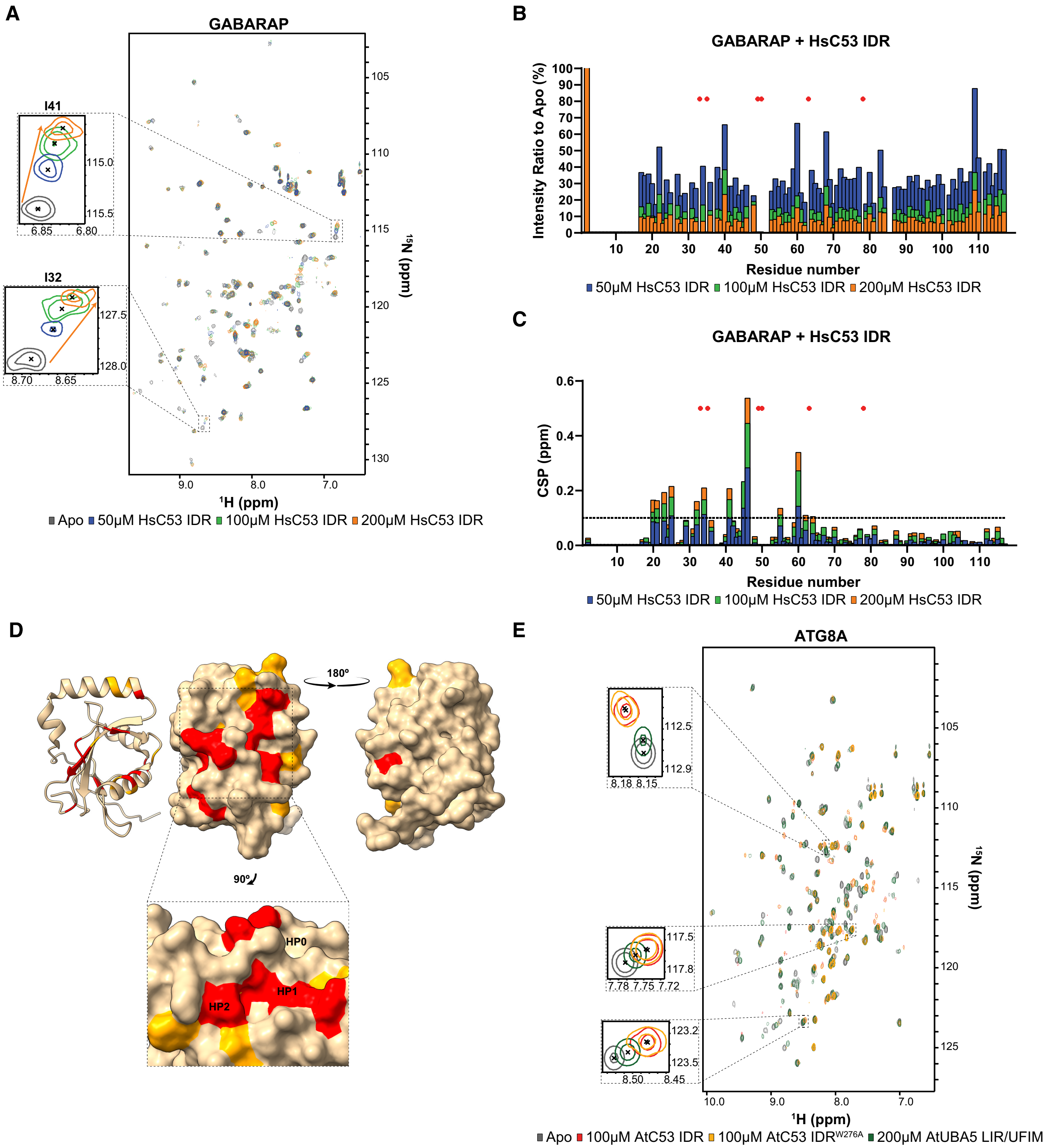
A
GABARAP
I41
I32
Apo 50μM HsC53 IDR 100μM HsC53 IDR 200μM HsC53 IDR
B
GABARAP + HsC53 IDR
Intensity Ratio to Apo (%)
Residue number
50μM HsC53 IDR 100μM HsC53 IDR 200μM HsC53 IDR
C
GABARAP + HsC53 IDR
CSP (ppm)
Residue number
50μM HsC53 IDR 100μM HsC53 IDR 200μM HsC53 IDR
D
180°
90°
HP0
HP1
HP2
E
ATG8A
Apo 100μM AtC53 IDR 100μM AtC53 IDR W276A 200μM AtUBA5 LIR/UFIM

Figure EV4.

Figure EV5. HsC53^{cAIM} binds GABARAP with higher affinity when compared to HsC53^{wt}.

A Titrations of GABARAP with HsC53^{wt}. The concentrations of reactants are 40 μM (1, 2, 3, 4) or 70 μM (5) for GABARAP (in cell) and 250 μM (1, 2, 3, 4) or 310 μM HsC53^{wt} (in syringe) (5).

B Titrations of GABARAP with HsC53^{cAIM}. The concentrations of reactants are 40 μM (1, 2, 3, 4) or 70 μM (5) for GABARAP (in cell) and 250 μM (1, 2, 3, 4) or 360 μM HsC53^{cAIM} (in syringe) (5).

C Titrations of GABARAP with HsC53^{sAIM}. The concentrations of reactants are 40 μM for GABARAP (in cell) and 250 μM HsC53^{sAIM}. Global analysis was performed using a hetero-association model A + B. The top panels show the SVD reconstructed thermograms, the middle panel shows the isotherms, and the bottom panel shows the residuals. Extracted global parameters and their 68.3% confidence interval are reported in the respective tables. Thermograms were reconstructed with NITPIC, global analysis was done in SEDPHAT, and data visualization was plotted in GUSSI. The dissociation constant (K_D) is reported in μM units, while the enthalpy (ΔH) is reported in kcal/mol units.

© 2023 The Authors

A

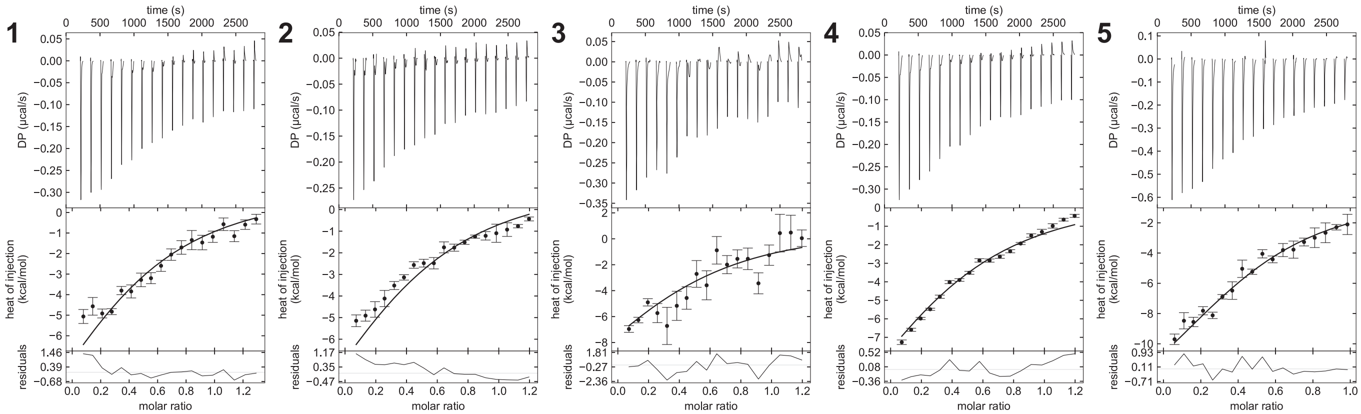

	Best-fit value	68.3% Confidence interval
K_D	20.86	8.35, 53.77
ΔH	-16.47	-37.07, -9.98

B

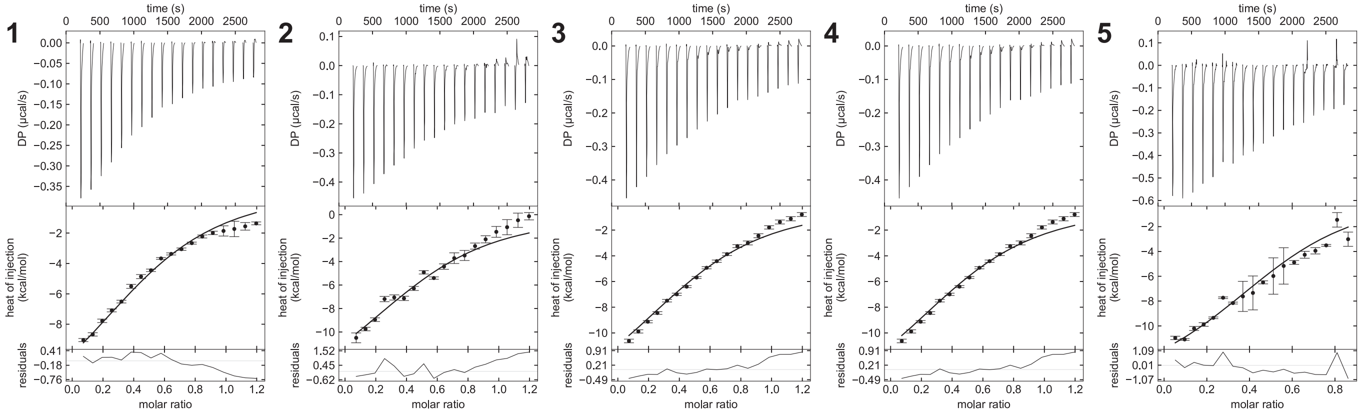

	Best-fit value	68.3% Confidence interval
K_D	11.24	5.91, 24.90
ΔH	-16.96	-25.18, -12.30

C

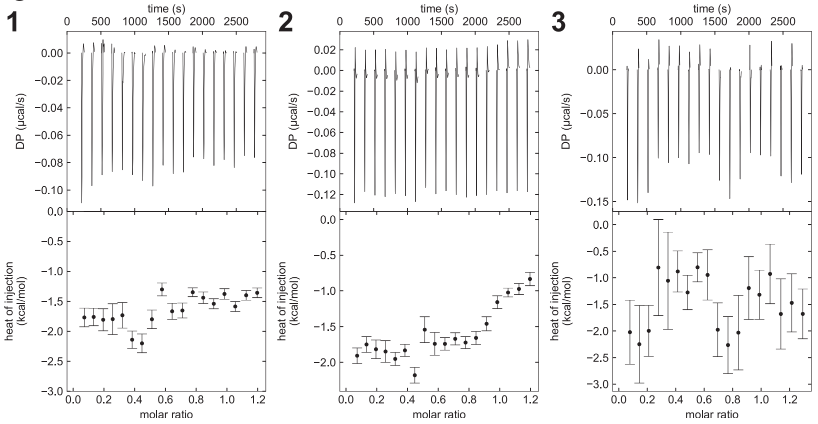

Figure EV5.

Appendix table of contents

Appendix Fig. S1

Appendix Fig. S2

Appendix Fig. S3

Appendix Fig. S4

Appendix Fig. S5

Appendix Fig. S6

Appendix Fig. S7

Appendix Fig. S8

Appendix Fig. S9

Appendix Fig. S10

Appendix Fig. S11

Supplementary Data S1

Supplementary Data S2

Fig. S1

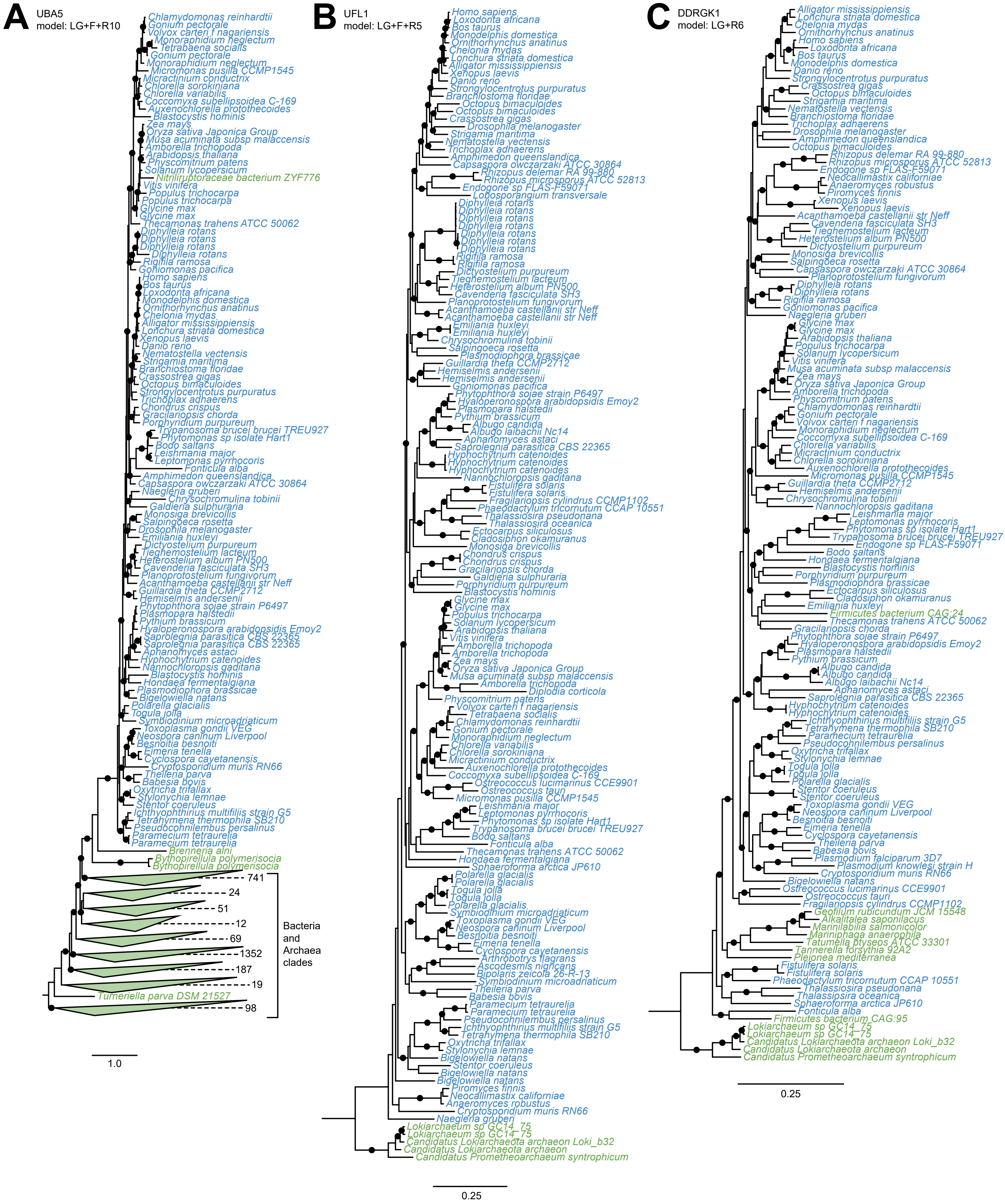
A
UBA5
model: LG+F+R10
Chlamydomonas reinhardtii
Gonium pectorale
Volvox carteri f nagariensis
Monoraphidium neglectum
Tetrabaena socialis
Gonium pectorale
Monoraphidium neglectum
Micromonas pusilla CCMP1545
Micractinium conductrix
Chlorella sorokiniana
Chlorella variabilis
Coccomyxa subellipsoidea C-169
Auxenochlorella protothecoides
Blastocystis hominis
Zea mays
Oryza sativa Japonica Group
Musa acuminata subsp malaccensis
Amborella trichopoda
Arabidopsis thaliana
Physcomitrium patens
Solanum lycopersicum
Nitriliruptoraceae bacterium ZYF776
Vitis vinifera
Populus trichocarpa
Populus trichocarpa
Glycine max
Glycine max
Thecamonas trahens ATCC 50062
Diphylleia rotans
Diphylleia rotans
Diphylleia rotans
Diphylleia rotans
Rigifila ramosa
Goniomonas pacifica
Homo sapiens
Bos taurus
Loxodonta africana
Monodelphis domestica
Ornithorhynchus anatinus
Chelonia mydas
Alligator mississippiensis
Lonchura striata domestica
Xenopus laevis
Danio rerio
Nematostella vectensis
Strigamia maritima
Branchiostoma floridae
Crassostrea gigas
Octopus bimaculoides
Strongylocentrotus purpuratus
Trichoplax adhaerens
Chondrus crispus
Gracilariopsis chorda
Porphyridium purpureum
Trypanosoma brucei brucei TREU927
Phytomonas sp isolate Hart1
Bodo saltans
Leishmania major
Leptomonas pyrrhocoris
Fonticula alba
Amphimedon queenslandica
Capsaspora owczarzaki ATCC 30864
Naegleria gruberi
Chrysochromulina tobinii
Galdieria sulphuraria
Monosiga brevicollis
Salpingoeca rosetta
Drosophila melanogaster
Emiliania huxleyi
Dictyostelium purpureum
Tieghemostelium lacteum
Heterostelium album PN500
Cavenderia fasciculata SH3
Planoprotostelium fungivorum
Acanthamoeba castellanii str Neff
Guillardia theta CCMP2712
Hemiselmis andersenii
Phytophthora sojae strain P6497
Plasmopara halstedii
Pythium brassicum
Hyaloperonospora arabidopsidis Emoy2
Saprolegnia parasitica CBS 22365
Saprolegnia parasitica CBS 22365
Aphanomyces astaci
Hyphochytrium catenoides
Nannochloropsis gaditana
Blastocystis hominis
Hondaea fermentalgiana
Plasmodiophora brassicae
Bigelowiella natans
Polarella glacialis
Togula jolla
Symbiodinium microadriaticum
Toxoplasma gondii VEG
Neospora caninum Liverpool
Besnoitia besnoiti
Eimeria tenella
Cyclospora cayetanensis
Cryptosporidium muris RN66
Theileria parva
Babesia bovis
Oxytricha trifallax
Stylonychia lemnae
Stentor coeruleus
Ichthyophthirius multifiliis strain G5
Tetrahymena thermophila SB210
Pseudocohnilembus persalinus
Paramecium tetraurelia
Paramecium tetraurelia
Brenneria alni
Bythopirellula polymerisocia
Bythopirellula polymerisocia
741
24
51
12
69
1352
187
19
Turneriella parva DSM 21527
98
Bacteria
and
Archaea
clades
1.0
B
UFL1
model: LG+F+R5
Homo sapiens
Loxodonta africana
Bos taurus
Monodelphis domestica
Ornithorhynchus anatinus
Chelonia mydas
Lonchura striata domestica
Alligator mississippiensis
Xenopus laevis
Danio rerio
Strongylocentrotus purpuratus
Branchiostoma floridae
Octopus bimaculoides
Octopus bimaculoides
Crassostrea gigas
Drosophila melanogaster
Strigamia maritima
Nematostella vectensis
Trichoplax adhaerens
Amphimedon queenslandica
Capsaspora owczarzaki ATCC 30864
Rhizopus delemar RA 99-880
Rhizopus microsporus ATCC 52813
Endogone sp FLAS-F59071
Lobosporangium transversale
Diphylleia rotans
Diphylleia rotans
Diphylleia rotans
Diphylleia rotans
Diphylleia rotans
Diphylleia rotans
Diphylleia rotans
Rigifila ramosa
Rigifila ramosa
Dictyostelium purpureum
Tieghemostelium lacteum
Heterostelium album PN500
Cavenderia fasciculata SH3
Planoprotostelium fungivorum
Acanthamoeba castellanii str Neff
Acanthamoeba castellanii str Neff
Emiliania huxleyi
Emiliania huxleyi
Chrysochromulina tobinii
Salpingoeca rosetta
Plasmodiophora brassicae
Guillardia theta CCMP2712
Hemiselmis andersenii
Hemiselmis andersenii
Goniomonas pacifica
Phytophthora sojae strain P6497
Hyaloperonospora arabidopsidis Emoy2
Plasmopara halstedii
Pythium brassicum
Albugo candida
Albugo laibachii Nc14
Aphanomyces astaci
Saprolegnia parasitica CBS 22365
Hyphochytrium catenoides
Hyphochytrium catenoides
Hyphochytrium catenoides
Nannochloropsis gaditana
Fistulifera solaris
Fistulifera solaris
Fragilariopsis cylindrus CCMP1102
Phaeodactylum tricornutum CCAP 10551
Thalassiosira pseudonana
Thalassiosira oceanica
Ectocarpus siliculosus
Cladosiphon okamuranus
Monosiga brevicollis
Chondrus crispus
Chondrus crispus
Gracilariopsis chorda
Galdieria sulphuraria
Porphyridium purpureum
Blastocystis hominis
Glycine max
Glycine max
Populus trichocarpa
Solanum lycopersicum
Arabidopsis thaliana
Vitis vinifera
Amborella trichopoda
Amborella trichopoda
Zea mays
Oryza sativa Japonica Group
Musa acuminata subsp malaccensis
Amborella trichopoda
Diplodia corticola
Physcomitrium patens
Volvox carteri f nagariensis
Tetrabaena socialis
Chlamydomonas reinhardtii
Gonium pectorale
Monoraphidium neglectum
Chlorella variabilis
Chlorella sorokiniana
Micractinium conductrix
Auxenochlorella protothecoides
Coccomyxa subellipsoidea C-169
Ostreococcus lucimarinus CCE9901
Ostreococcus tauri
Micromonas pusilla CCMP1545
Leishmania major
Leptomonas pyrrhocoris
Phytomonas sp isolate Hart1
Trypanosoma brucei brucei TREU927
Bodo saltans
Fonticula alba
Thecamonas trahens ATCC 50062
Hondaea fermentalgiana
Sphaeroforma arctica JP610
Polarella glacialis
Polarella glacialis
Togula jolla
Togula jolla
Polarella glacialis
Symbiodinium microadriaticum
Toxoplasma gondii VEG
Neospora caninum Liverpool
Besnoitia besnoiti
Eimeria tenella
Cyclospora cayetanensis
Arthrobotrys flagrans
Ascodesmis nigricans
Bipolaris zeicola 26-R-13
Symbiodinium microadriaticum
Theileria parva
Babesia bovis
Paramecium tetraurelia
Paramecium tetraurelia
Pseudocohnilembus persalinus
Ichthyophthirius multifiliis strain G5
Tetrahymena thermophila SB210
Oxytricha trifallax
Stylonychia lemnae
Bigelowiella natans
Stentor coeruleus
Bigelowiella natans
Bigelowiella natans
Piromyces finnis
Neocallimastix californiae
Anaeromyces robustus
Cryptosporidium muris RN66
Naegleria gruberi
Lokiarchaeum sp GC14_75
Lokiarchaeum sp GC14_75
Candidatus Lokiarchaeota archaeon Loki_b32
Candidatus Lokiarchaeota archaeon
Candidatus Prometheoarchaeum syntrophicum
0.25
C
DDRGK1
model: LG+R6
Alligator mississippiensis
Lonchura striata domestica
Chelonia mydas
Ornithorhynchus anatinus
Homo sapiens
Loxodonta africana
Bos taurus
Monodelphis domestica
Danio rerio
Strongylocentrotus purpuratus
Crassostrea gigas
Octopus bimaculoides
Strigamia maritima
Nematostella vectensis
Branchiostoma floridae
Trichoplax adhaerens
Drosophila melanogaster
Amphimedon queenslandica
Octopus bimaculoides
Rhizopus delemar RA 99-880
Rhizopus microsporus ATCC 52813
Endogone sp FLAS-F59071
Neocallimastix californiae
Anaeromyces robustus
Piromyces finnis
Xenopus laevis
Xenopus laevis
Acanthamoeba castellanii str Neff
Cavenderia fasciculata SH3
Tieghemostelium lacteum
Heterostelium album PN500
Dictyostelium purpureum
Monosiga brevicollis
Salpingoeca rosetta
Capsaspora owczarzaki ATCC 30864
Planoprotostelium fungivorum
Diphylleia rotans
Diphylleia rotans
Rigifila ramosa
Goniomonas pacifica
Naegleria gruberi
Glycine max
Glycine max
Arabidopsis thaliana
Populus trichocarpa
Solanum lycopersicum
Vitis vinifera
Musa acuminata subsp malaccensis
Zea mays
Oryza sativa Japonica Group
Amborella trichopoda
Physcomitrium patens
Chlamydomonas reinhardtii
Gonium pectorale
Volvox carteri f nagariensis
Monoraphidium neglectum
Coccomyxa subellipsoidea C-169
Chlorella variabilis
Micractinium conductrix
Chlorella sorokiniana
Auxenochlorella protothecoides
Micromonas pusilla CCMP1545
Guillardia theta CCMP2712
Hemiselmis andersenii
Chrysochromulina tobinii
Nannochloropsis gaditana
Leishmania major
Leptomonas pyrrhocoris
Phytomonas sp isolate Hart1
Trypanosoma brucei brucei TREU927
Endogone sp FLAS-F59071
Bodo saltans
Hondaea fermentalgiana
Blastocystis hominis
Porphyridium purpureum
Plasmodiophora brassicae
Ectocarpus siliculosus
Cladosiphon okamuranus
Emiliania huxleyi
Firmicutes bacterium CAG:24
Thecamonas trahens ATCC 50062
Gracilariopsis chorda
Phytophthora sojae strain P6497
Hyaloperonospora arabidopsidis Emoy2
Plasmopara halstedii
Pythium brassicum
Albugo candida
Albugo candida
Albugo laibachii Nc14
Aphanomyces astaci
Saprolegnia parasitica CBS 22365
Hyphochytrium catenoides
Hyphochytrium catenoides
Ichthyophthirius multifiliis strain G5
Tetrahymena thermophila SB210
Paramecium tetraurelia
Pseudocohnilembus persalinus
Oxytricha trifallax
Stylonychia lemnae
Togula jolla
Togula jolla
Polarella glacialis
Stentor coeruleus
Stentor coeruleus
Toxoplasma gondii VEG
Neospora caninum Liverpool
Besnoitia besnoiti
Eimeria tenella
Cyclospora cayetanensis
Theileria parva
Babesia bovis
Plasmodium falciparum 3D7
Plasmodium knowlesi strain H
Cryptosporidium muris RN66
Bigelowiella natans
Ostreococcus lucimarinus CCE9901
Ostreococcus tauri
Fragilariopsis cylindrus CCMP1102
Geofilum rubicundum JCM 15548
Alkalitalea saponilacus
Marinilabilia salmonicolor
Mariniphaga anaerophila
Tatumella ptyseos ATCC 33301
Tannerella forsythia 92A2
Plesiomonas mediterranea
Fistulifera solaris
Fistulifera solaris
Phaeodactylum tricornutum CCAP 10551
Thalassiosira pseudonana
Thalassiosira oceanica
Sphaeroforma arctica JP610
Fonticula alba
Firmicutes bacterium CAG:95
Lokiarchaeum sp GC14_75
Lokiarchaeum sp GC14_75
Candidatus Lokiarchaeota archaeon Loki_b32
Candidatus Lokiarchaeota archaeon
Candidatus Prometheoarchaeum syntrophicum
0.25

Appendix Figure S1. The prokaryotic origins of UBA5, UFL1, and DDRGK1.

Maximum likelihood phylogenies generated from multiple sequence alignments containing eukaryotic and prokaryotic homologs of UBA5 (**A**), UFL1 (**B**), and DDRGK1 (**C**). Eukaryotes and prokaryotes are denoted in blue and green, respectively. Black circles represent SH-aLRT (Shimodaira Hasegawa approximate likelihood ratio tests) ≥ 0.85. Phylogenetic models used for generating each phylogeny and the number of sequences within collapsed nodes are noted. Scale bars represent the number of substitutions per 100 amino acids.

Fig. S2

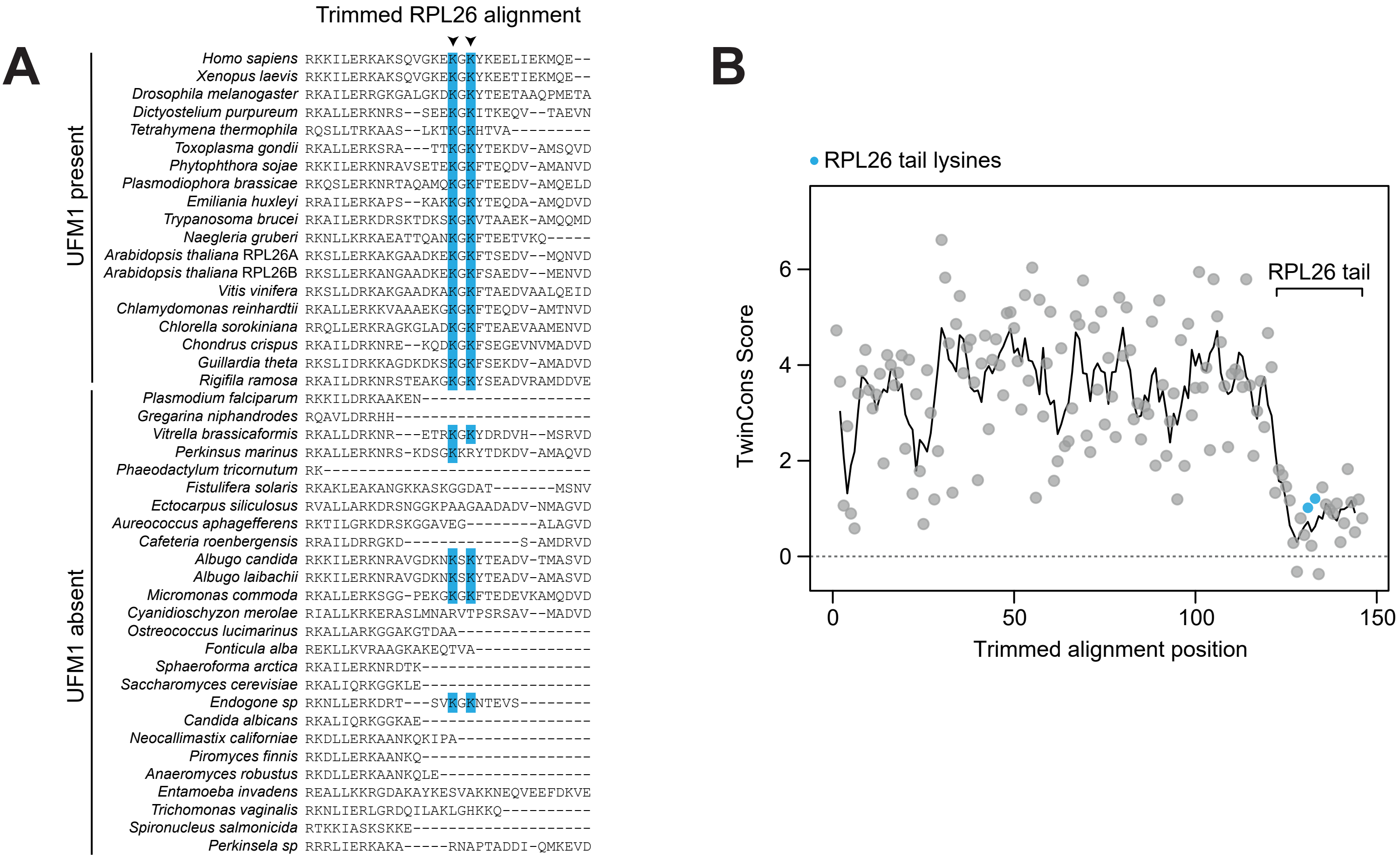

A
Trimmed RPL26 alignment
UFM1 present
Homo sapiens RKKILERKAKSQVGKEKGKYKEELIEKMQE--
Xenopus laevis RKKILERKAKSQVGKEKGKYKEETIEKMQE--
Drosophila melanogaster RKAILERRGKGALGKDKGKYTEETAAQPMETA
Dictyostelium purpureum RKALLERKNRS--SEEKGKITKEQV--TAEVN
Tetrahymena thermophila RQSLLTRKAAS--LKTKGKHTVA---------
Toxoplasma gondii RKALLERKSRA---TTKGKYTEKDV-AMSQVD
Phytophthora sojae RKKILERKNRAVSETEKGKFTEQDV-AMANVD
Plasmodiophora brassicae RKQSLERKNRTAQAMQKGKFTEEDV-AMQELD
Emiliania huxleyi RRAILERKAPS--KAKKGKYTEQDA-AMQDVD
Trypanosoma brucei RKAILERKDRSKTDKSKGKVTAAEK-AMQQMD
Naegleria gruberi RKNLLKRKAEATTQANKGKFTEETVKQ-----
Arabidopsis thaliana RPL26A RKSLLERKAKGAADKEKGKFTSEDV--MQNVD
Arabidopsis thaliana RPL26B RKSLLERKANGAADKEKGKFSAEDV--MENVD
Vitis vinifera RKSLLDRKAKGAADKAKGKFTAEDVAALQEID
Chlamydomonas reinhardtii RKALLERKKVAAAEKGKGKFTEQDV-AMTNVD
Chlorella sorokiniana RRQLLERKRAGKGLADKGKFTEAEVAAMENVD
Chondrus crispus RKAILDRKNRE--KQDKGKFSEGEVNVMADVD
Guillardia theta RKSLIDRKKAGDKDKSKGKFSEKDV-AMADVD
Rigifila ramosa RKAILDRKNRSTEAKGKGKYSEADVRAMDDVE
UFM1 absent
Plasmodium falciparum RKKILDRKAAKEN-------------------
Gregarina niphandrodes RQAVLDRRHH----------------------
Vitrella brassicaformis RKALLDRKNR---ETRKGKYDRDVH--MSRVD
Perkinsus marinus RKALLERKNRS-KDSGKKRYTDKDV-AMAQVD
Phaeodactylum tricornutum RK------------------------------
Fistulifera solaris RKAKLEAKANGKKASKGGDAT-------MSNV
Ectocarpus siliculosus RVALLARKDRSNGGKPAAGAADADV-NMAGVD
Aureococcus aphagefferens RKTILGRKDRSKGGAVEG--------ALAGVD
Cafeteria roenbergensis RRAILDRRGKD------------S-AMDRVD
Albugo candida RKKILERKNRAVGDKNKSKYTEADV-TMASVD
Albugo laibachii RKKILERKNRAVGDKNKSKYTEADV-AMASVD
Micromonas commoda RKALLERKSGG-PEKGKGKFTEDEVKAMQDVD
Cyanidioschyzon merolae RIALLKRKERASLMNARVTPSRSAV--MADVD
Ostreococcus lucimarinus RKALLARKGGAKGTDAA---------------
Fonticula alba REKLLKVRAAGKAKEQTVA-------------
Sphaeroforma arctica RKAILERKNRDTK-------------------
Saccharomyces cerevisiae RKALIQRKGGKLE-------------------
Endogone sp RKNLLERKDRT---SVKGKNTEVS--------
Candida albicans RKALIQRKGGKAE-------------------
Neocallimastix californiae RKDLLERKAANKQKIPA---------------
Piromyces finnis RKDLLERKAANKQ-------------------
Anaeromyces robustus RKDLLERKAANKQLE-----------------
Entamoeba invadens REALLKKRGDAKAYKESVAKKNEQVEEFDKVE
Trichomonas vaginalis RKNLIERLGRDQILAKLGHKKQ----------
Spironucleus salmonicida RTKKIASKSKKE--------------------
Perkinsela sp RRRLIERKAKA-----RNAPTADDI-QMKEVD
B
RPL26 tail lysines
RPL26 tail
TwinCons Score
0
2
4
6
Trimmed alignment position
0
50
100
150

Appendix Figure S2. Conservation analysis of RPL26 shows that the UFMylated tail region is divergent.

(A) **Multiple sequence alignment of RPL26 showing the conservation of the C-terminal tail in species with and without UFM1.** Lysine residues that are ufmylated have been highlighted. (**B) TwinCons analysis comparing the sequence conservation of RPL26.** The tail region is highly polymorphic.

Fig. S3

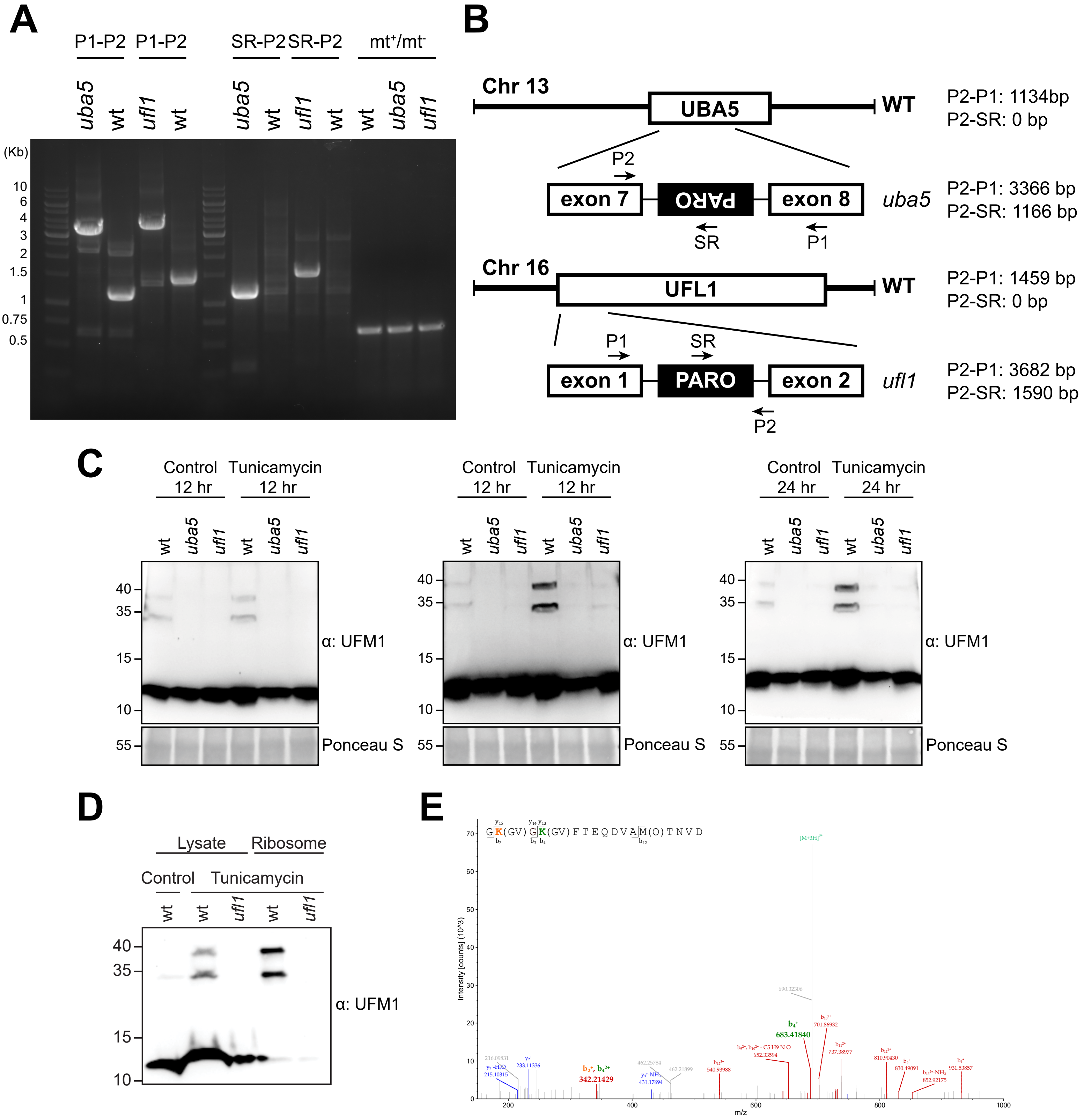
A
P1-P2
P1-P2
SR-P2
SR-P2
mt+/mt-
uba5
wt
ufl1
wt
uba5
wt
ufl1
wt
wt
uba5
ufl1
(Kb)
10
6
4
3
2
1.5
1
0.75
0.5
B
Chr 13
UBA5
WT
P2-P1: 1134bp
P2-SR: 0 bp
P2
exon 7
PARO
exon 8
uba5
SR
P1
P2-P1: 3366 bp
P2-SR: 1166 bp
Chr 16
UFL1
WT
P2-P1: 1459 bp
P2-SR: 0 bp
P1
SR
exon 1
PARO
exon 2
ufl1
P2
P2-P1: 3682 bp
P2-SR: 1590 bp
C
Control 12 hr
Tunicamycin 12 hr
Control 24 hr
Tunicamycin 24 hr
wt
uba5
ufl1
40
35
15
10
55
α: UFM1
Ponceau S
D
Lysate
Ribosome
Control
Tunicamycin
wt
wt
ufl1
wt
ufl1
40
35
15
10
α: UFM1
E
GK(GV)GK(GV)FTEQDVAM(O)TNVD
Intensity [counts] (10^3)
m/z
683.41840
342.21429
701.86932
737.38977
810.90430
830.49091
931.53857
852.92175
652.33594
540.93988
233.11336
215.10315
431.17694
462.21699
690.32306

Appendix Figure S3. Characterization of the *Chlamydomonas reinhardtii* UFMylation pathway mutants.

(A) Genotyping of *C. reinhardtii uba5* and *ufl1* mutants. *Left Panel*, mating type (mt +/-) and insertion site PCR products from purified genomic DNA samples prepared from wt, *uba5* and *ufl1* genotypes. PCR products were run on a 1% (w/v) agarose gel. DNA size markers are reported in Kb. **(B) Schematic diagram indicating the insertion site of the mutagenic cassette (PARO) in *ufl1* and *uba5* mutants.** Primers are indicated with arrows and expected PCR products from wild type and mutants are reported next to each respective diagram. **(C) RPL26 mono- and di-UFMylation is lost in *uba5* and *ufl1* mutants**. Cells were either left untreated or treated for 24 hours with 200 ng/mL tunicamycin. Protein extracts were analyzed by immunoblotting with anti-UFM1 antibodies. Total proteins were analyzed by Ponceau S staining. Quantification is shown in Figure 1C. **(D) *C. reinhardtii* (Cr) ribosomes are specifically mono- and di-UFMylated.** Liquid TAP cultures were either left untreated (control) or treated for 24 hours with 200 ng/mL tunicamycin. Protein extracts and purified ribosomes were analyzed by immunoblotting with anti-UFM1 antibodies. (E) ***C. reinhardtii* (Cr) RPL26 is di-UFMylated.** nanoLC-MS/MS spectra of K-GV-containing peptides associated with RPL26. Spectrum, derived from RPL26-(UFM1)$_2$, is of a RPL26 peptide containing K-GV remnants on K131 and K133, and oxidation of M141.

Fig. S4

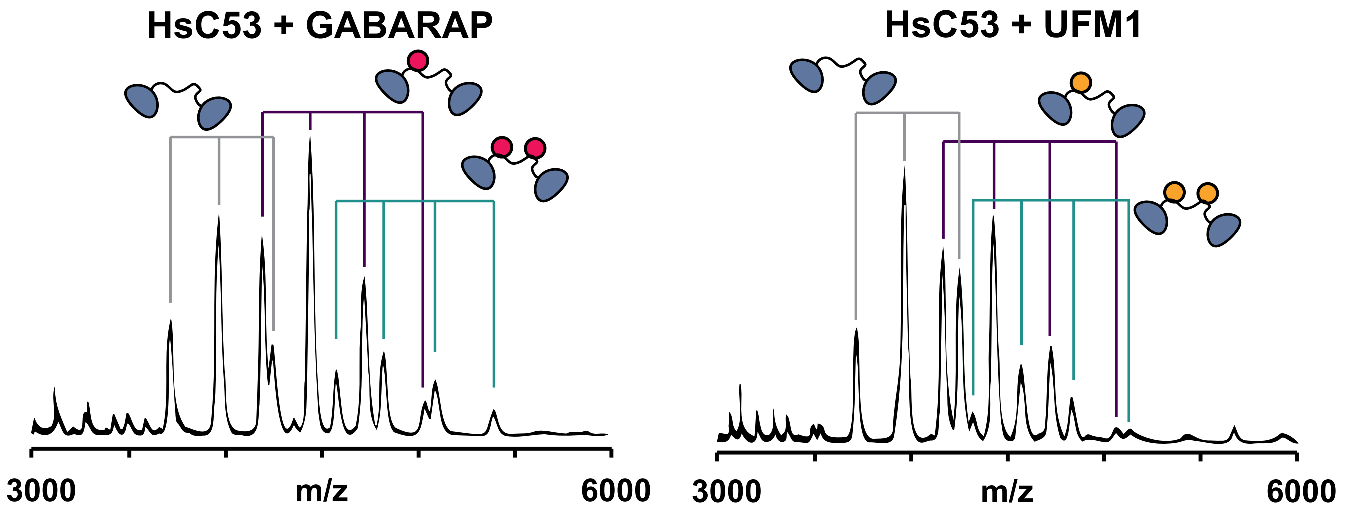

Appendix Figure S4. Native Mass-Spectrometry (nMS) spectra of HsC53 with GABARAP or HsUFM1 show very similar binding profiles.

Left Panel, GABARAP (4 µM) and HsC53 (2 µM). *Right Panel*, HsUFM1 (4 µM) and HsC53 (2 µM). Binding of HsC53 to GABARAP and HsUFM1 is observed in 1:1 (violet) and 1:2 ratios (teal)**.**

Fig. S5

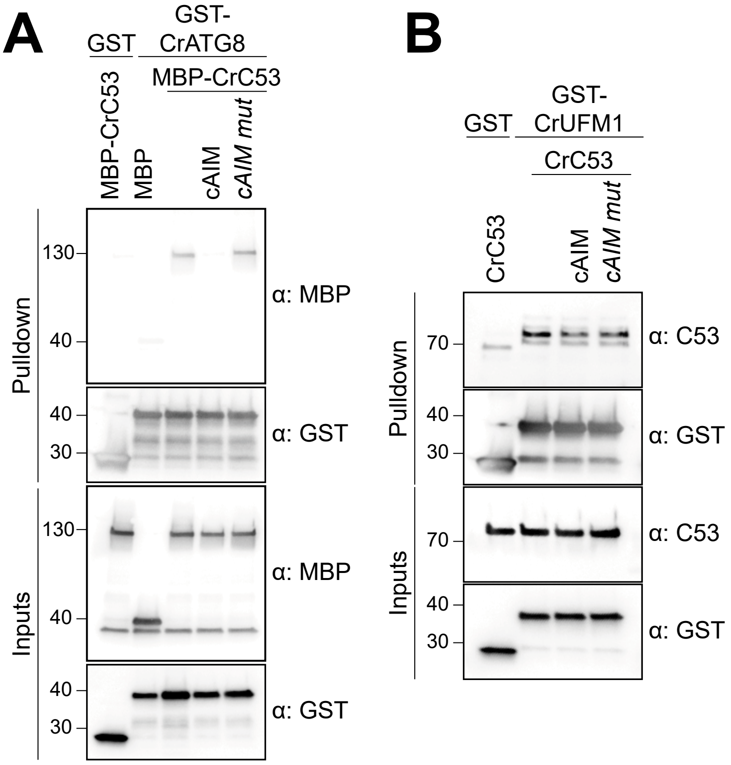

A
GST
GST-CrATG8
MBP-CrC53
MBP-CrC53
MBP
cAIM
cAIM mut
Pulldown
Inputs
130
40
30
α: MBP
α: GST
B
GST
GST-CrUFM1
CrC53
CrC53
cAIM
cAIM mut
70
α: C53

Appendix Figure S5. The canonical ATG8 Interacting Motif (cAIM) peptide cannot outcompete C53-UFM1 interaction for *C. reinhardtii* (Cr) protein orthologs.

(A) CrC53 binds CrATG8A in a cAIM-dependent manner. (B) CrC53 binds UFM1 in a cAIM-independent manner. Bacterial lysates containing recombinant protein or purified recombinant proteins were mixed and pulled down with glutathione magnetic agarose beads. Input and bound proteins were visualized by immunoblotting with anti-GST, anti-MBP or anti-AtC53 antibodies. cAIM wild type or mutant peptides were used to a final concentration of 200 µM.

Fig. S6

A

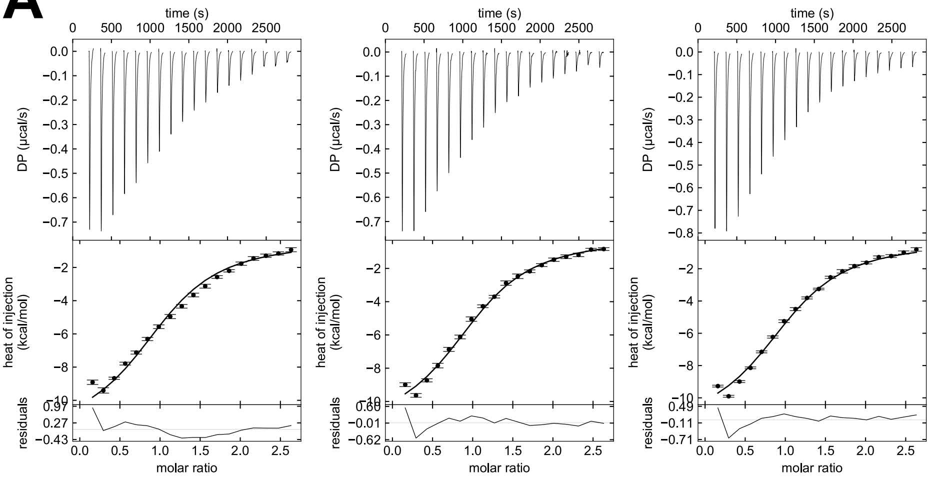

	Best-fit value	68.3% Confidence interval
K_D	7.51	5.30, 10.97
ΔH	-11.98	-14.31, -10.40

B

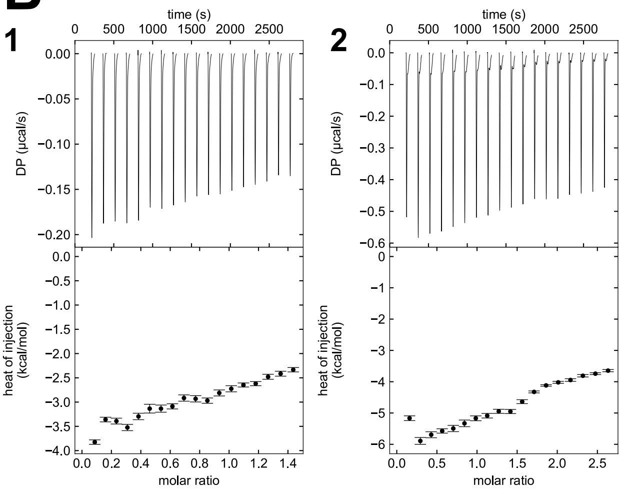

Appendix Figure S6. Titrations of HsUFM1 with UBA5 LIR peptide and HsC53^{wt}.

(A) Titrations of HsUFM1 with UBA5 LIR. The concentrations of reactants are 40 µM for UFM1 (in cell) and 550 µM UBA5 LIR (in syringe). **(B) Titrations of HsUFM1 with HsC53^{wt}** The concentrations of reactants are 40 µM for UFM1 (in cell) and 300 µM (**1**) or 550 µM HsC53^{wt} (in syringe) (**2**). Global analysis was performed using a hetero-association model A + B. The top panels show the SVD reconstructed thermograms, the middle panel shows the isotherms, and the bottom panel shows the residuals. Extracted global parameters and their 68.3% confidence interval are reported in the respective tables. Thermograms were reconstructed with NITPIC, global analysis was done in SEDPHAT, and data visualization was plotted in GUSSI. The dissociation constant (K_D) is reported µM units, while the enthalpy (ΔH) is reported in kcal/mol units.

Fig. S7

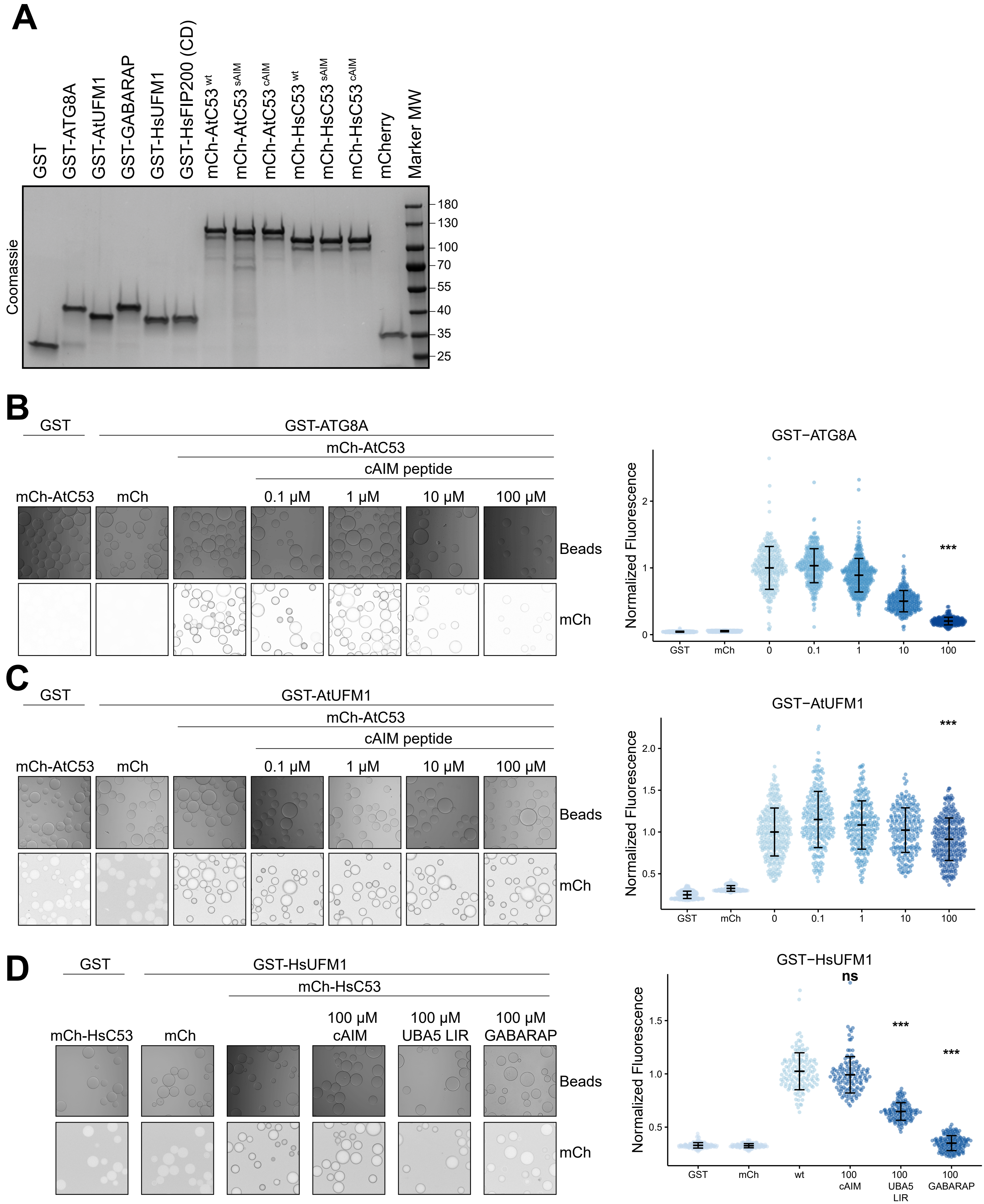
A
GST
GST-ATG8A
GST-AtUFM1
GST-GABARAP
GST-HsUFM1
GST-HsFIP200 (CD)
mCh-AtC53 wt
mCh-AtC53 sAIM
mCh-AtC53 cAIM
mCh-HsC53 wt
mCh-HsC53 sAIM
mCh-HsC53 cAIM
mCherry
Marker MW
Coomassie
180
130
100
70
55
40
35
25
B
GST
GST-ATG8A
mCh-AtC53
cAIM peptide
mCh-AtC53
mCh
0.1 µM
1 µM
10 µM
100 µM
Beads
mCh
GST−ATG8A
Normalized Fluorescence
***
GST
mCh
0
0.1
1
10
100
C
GST
GST-AtUFM1
mCh-AtC53
cAIM peptide
Beads
mCh
GST−AtUFM1
***
D
GST
GST-HsUFM1
mCh-HsC53
mCh-HsC53
mCh
100 µM cAIM
100 µM UBA5 LIR
100 µM GABARAP
Beads
mCh
GST−HsUFM1
ns
***
***
wt
100 cAIM
100 UBA5 LIR
100 GABARAP

Appendix Figure S7. The canonical ATG8 Interacting Motif (cAIM) cannot outcompete C53-UFM1 interaction.

(A) Purified proteins used for the protein-protein interaction microscopy binding assays. Recombinant proteins were analyzed for purity by SDS-PAGE followed by Coomassie staining. Marker molecular weights (MW) are indicated in kDa. mCh: mCherry. **(B, C) Microscopy-based protein–protein interaction assays showing unlike ATG8A-C53 interaction, UFM1-C53 interaction is insensitive to cAIM peptide competition**. Glutathione-sepharose beads were prepared by incubating them with GST-ATG8A (C) or GST-AtUFM1 (D). The pre-assembled beads were then washed and mixed with 1 µM of AtC53 containing increasing concentrations of cAIM peptide (0-100 µM). The beads were then imaged using a confocal microscope. *Left Panel,* representative confocal images (inverted grayscale) for each condition are shown. *Right panel*, normalized fluorescence is shown for each condition with the mean (± SD) of 2 independent replicates containing 2 technical replicates. Unpaired two-samples Wilcoxon test with continuity correction was performed to analyze the differences between wild type without cAIM peptide and wild type with 100 µM cAIM peptide. ***, p-value < 0.001. Total number of beads, mean, median, standard deviation and p-values are reported in Supplementary data S1. **(D) Microscopy-based protein–protein interaction assays showing UBA5 LIR peptide and GABARAP can compete for C53 interaction with UFM1**. Glutathione-sepharose beads were prepared by incubating them with GST-HsUFM1. The pre-assembled beads were then washed and mixed with 1 µM of HsC53 with either 100 µM cAIM peptide, 100 µM UBA5 LIR peptide or 100 µM GABARAP. The beads were then imaged using a confocal microscope. *Left Panel,* representative confocal images (inverted grayscale) for each condition are shown. *Right panel*, normalized fluorescence is shown for each condition with the mean (± SD). Unpaired two-samples Wilcoxon test with continuity correction was performed to analyze the differences between wild type and wild type mixed with either cAIM peptide, UBA5 LIR peptide or GABARAP. ns, p-value > 0.05, ***, p-value < 0.001. Total number of beads, mean, median, standard deviation and p-values are reported in Supplementary data S1.

Fig. S8

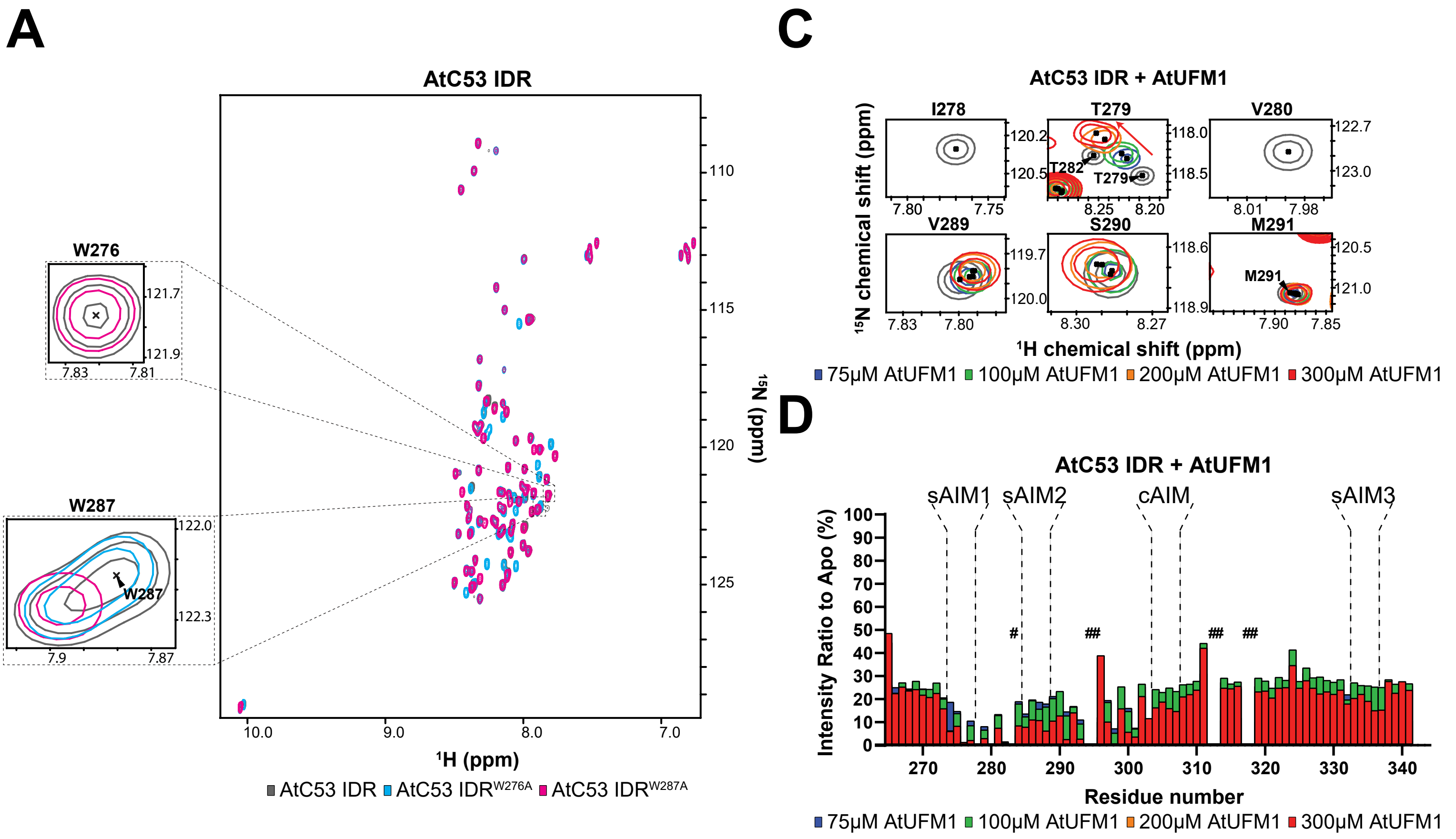

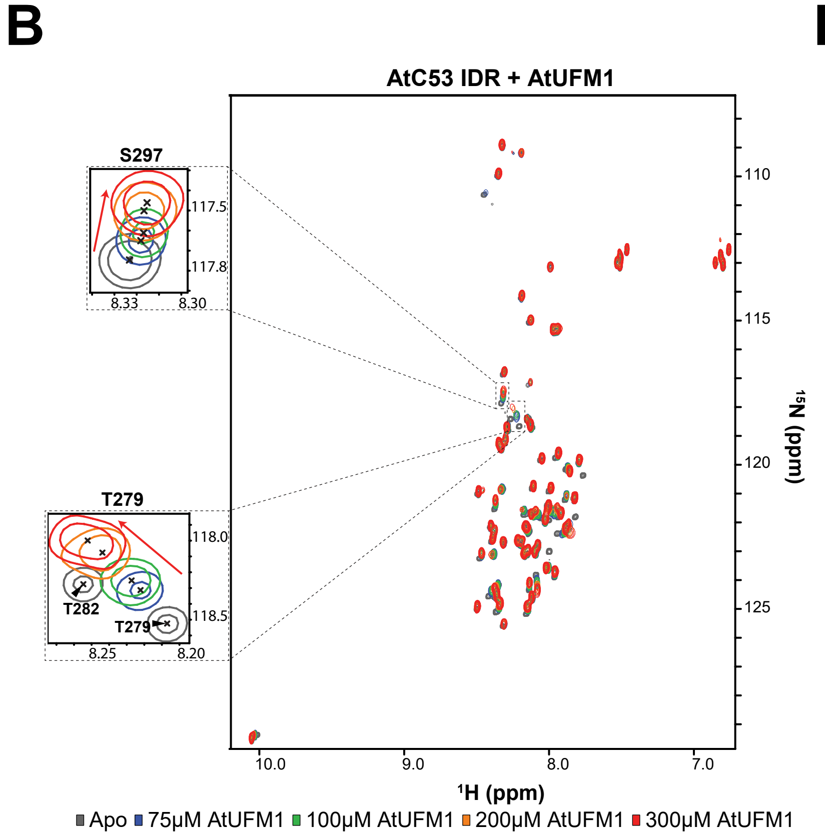

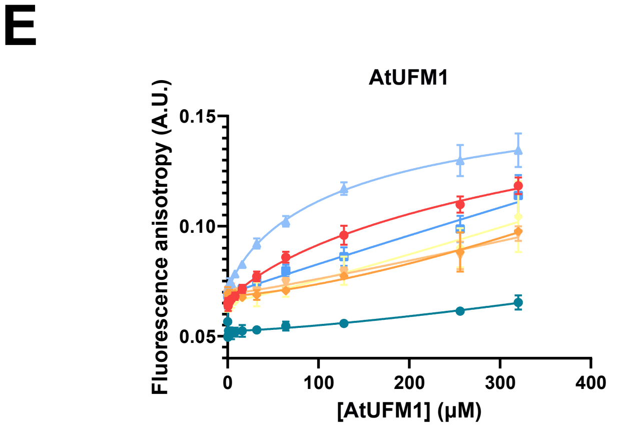

	Peptide sequence	KD (μM)	95% C.I.
sAIM1	AADSIDWDITVET	*N.D.*	*N.D.*
sAIM2	ETPEIDWDVSMVEEV	*N.D.*	*N.D.*
cAIM	DLGSYEIVNASDI	*N.D.*	*N.D.*
sAIM3	DVSEISWDVSVET	*N.D.*	*N.D.*
sAIM1,2	AADSIDWDITVETPEIDWDVSMVEEV	148.2	84.3 - 533.1
cAIM mut	AADSWDDIITVET	*N.D.*	*N.D.*
sAIM mut	AADSIDADITVET	*N.D.*	*N.D.*

Appendix Figure S8. Structural characterization of AtC53 IDR binding to AtUFM1 using NMR spectroscopy.

(A) Validation of AtC53 IDR backbone resonance assignments. Overlaid ^1H-^{15}N HSQC spectra of 100 µM isotope-labelled AtC53 IDR (grey), AtC53 IDR^{W276A} (cyan) and AtC53 IDR^{W287A} (magenta). Insets of resonances corresponding to residues W276 and W287 are shown. **(B) Addition of AtUFM1 changes the magnetic resonance of specific residues in AtC53.** Overlaid ^1H-^{15}N HSQC spectra of isotope-labelled AtC53 IDR (100 µM) in their free (gray) or bound state to 75 µM (blue), 100 µM (green), 200 µM (orange) and 300 µM (red) unlabelled AtUFM1. Examples of individual peaks that shift upon binding are shown as insets. Chemical shifts are indicated with arrows. **(C) Residues downstream AtC53 sAIM1 but not sAIM2 contribute to AtUFM1 binding.** Insets of overlaid ^1H-^{15}N HSQC spectra of isotope-labelled AtC53 IDR (100 µM) showing chemical shift perturbations of individual peaks from backbone amides of AIM residues in their free (gray) or bound state to 75 µM (blue), 100 µM (green), 200 µM (orange) and 300 µM (red) unlabelled AtUFM1. Chemical shifts are indicated with arrows. **(D) Signal intensity changes in AtC53 IDR upon binding of AtUFM1 are concentration dependent.** Intensity ratio broadening of AtC53 IDR (100 µM) in the presence of 75 µM (blue), 100 µM (green), 200 µM (orange) and 300 µM (red) AtUFM1. Bars corresponding to residues in AIMs are highlighted. Unassigned AtC53 IDR residues are indicated by hashtags. **(E) A peptide spanning C53 sAIM1 and sAIM2 binds UFM1.** Fluorescence anisotropy (FA) assay using AtC53 IDR-derived peptides as a probe for AtUFM1 binding. Error bars represent the FA values expressed in arbitrary units (A.U.) as mean ± s.d. of triplicate experiments using the same buffer and protein conditions. Peptide sequences are listed, sAIMs are highlighted. N.D. not determined.

Fig. S9

A

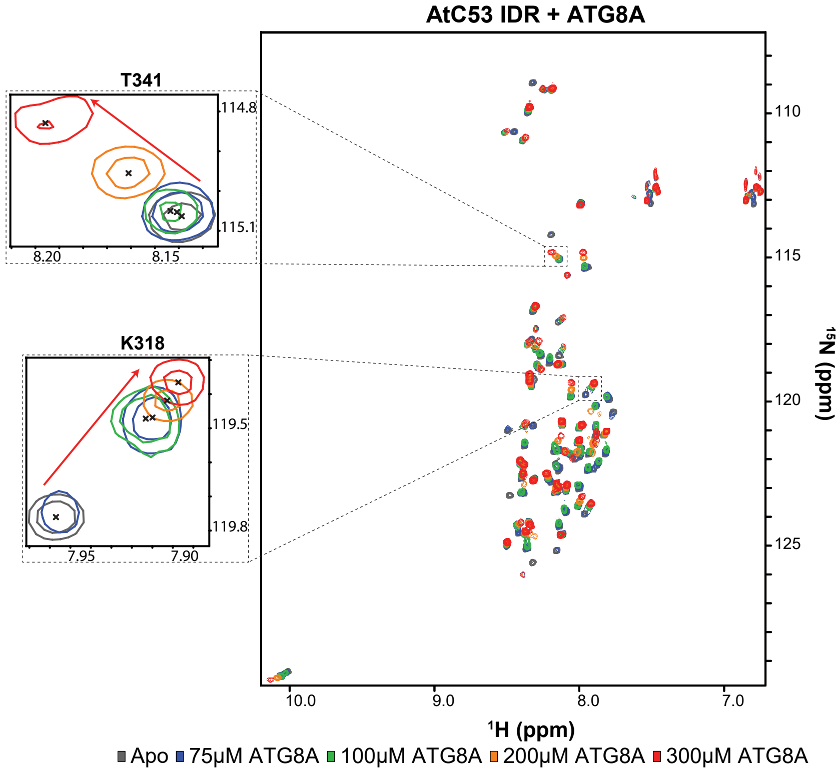

B

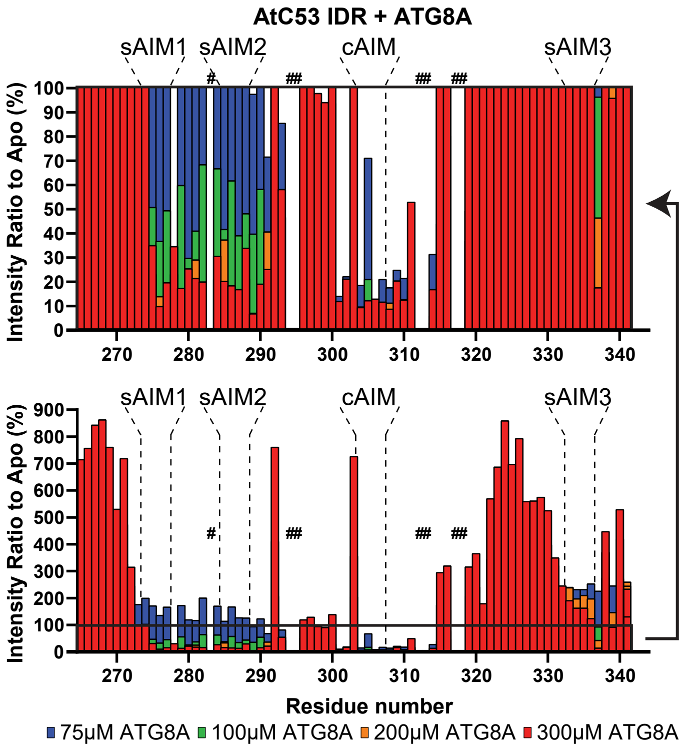

C

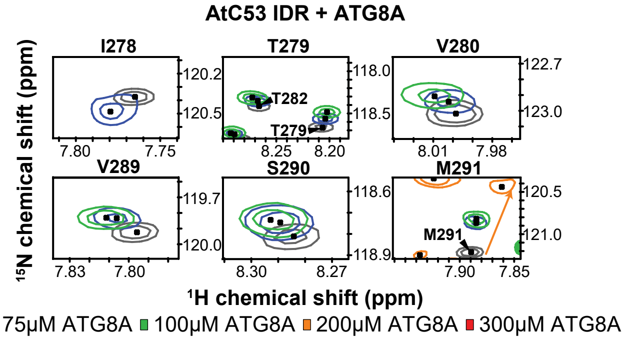

D

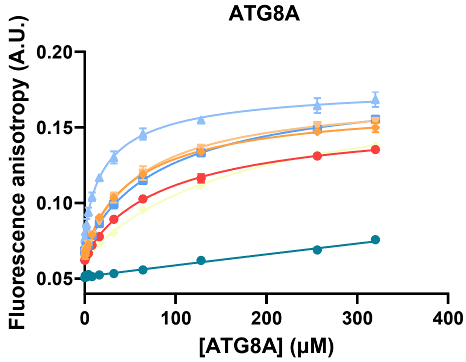

	Peptide sequence	KD (μM)	95% C.I.
sAIM1	AADSIDWDITVET	97.59	77.91 - 133.0
sAIM2	ETPEIDWDVSMVEEV	56.82	50.14 - 66.01
cAIM	DLGSYEIVNASDI	62.47	53.80 - 75.25
sAIM3	DVSEISWDVSVET	176.5	137.6 - 249.7
sAIM1,2	AADSIDWDITVETPEIDWDVSMVEEV	26.54	21.81 - 33.87
cAIM mut	AADSWDDITVET	94.27	77.47 - 122.2
sAIM mut	AADSIDADITVET	*N.D.*	*N.D.*

Appendix Figure S9. Structural characterization of AtC53 IDR binding to ATG8A using NMR spectroscopy.

(A) Addition of ATG8A affects a greater number of residues in the AtC53 IDR spectra. Overlaid ^{1}H-^{15}N HSQC spectra of isotope-labelled AtC53 IDR (100 µM) in their free (gray) or bound state to 75 µM (blue), 100 µM (green), 200 µM (orange) and 300 µM (red) unlabelled ATG8A. Insets of individual peaks that shifted upon binding are shown. Chemical shifts are indicated with arrows. **(B) Signal intensity changes in AtC53 IDR upon binding of ATG8A are concentration dependent.** Intensity ratio broadening of AtC53 IDR (100 µM) in the presence of 75 µM (blue), 100 µM (green), 200 µM (orange) and 300 µM (red) ATG8A. Top panel represents an inset of lower panel. Unassigned AtC53 IDR residues are indicated by hashtags. Bars corresponding to residues in AIMs are highlighted. Top panel represents an inset of lower panel. **(C) Residues downstream AtC53 sAIM1 and sAIM2 contribute to ATG8A binding.** Insets of overlaid ^{1}H-^{15}N HSQC spectra of isotope-labelled AtC53 IDR (100 µM) showing chemical shift perturbations of individual peaks from backbone amides of AIM residues in their free (gray) or bound state to 75 µM (blue), 100 µM (green), 200 µM (orange) and 300 µM (red) unlabelled ATG8A. Chemical shifts are indicated with arrows. **(D) A peptide spanning C53 sAIM1 and sAIM2 binds ATG8A.** Fluorescence anisotropy (FA) assay using AtC53 IDR-derived peptides as a probe for ATG8A binding. Error bars represent the FA values expressed in arbitrary units (A.U.) as mean ± s.d. of triplicate experiments using the same buffer and protein conditions. Peptide sequences are listed, sAIMs are highlighted. N.D. not determined.

Fig. S10

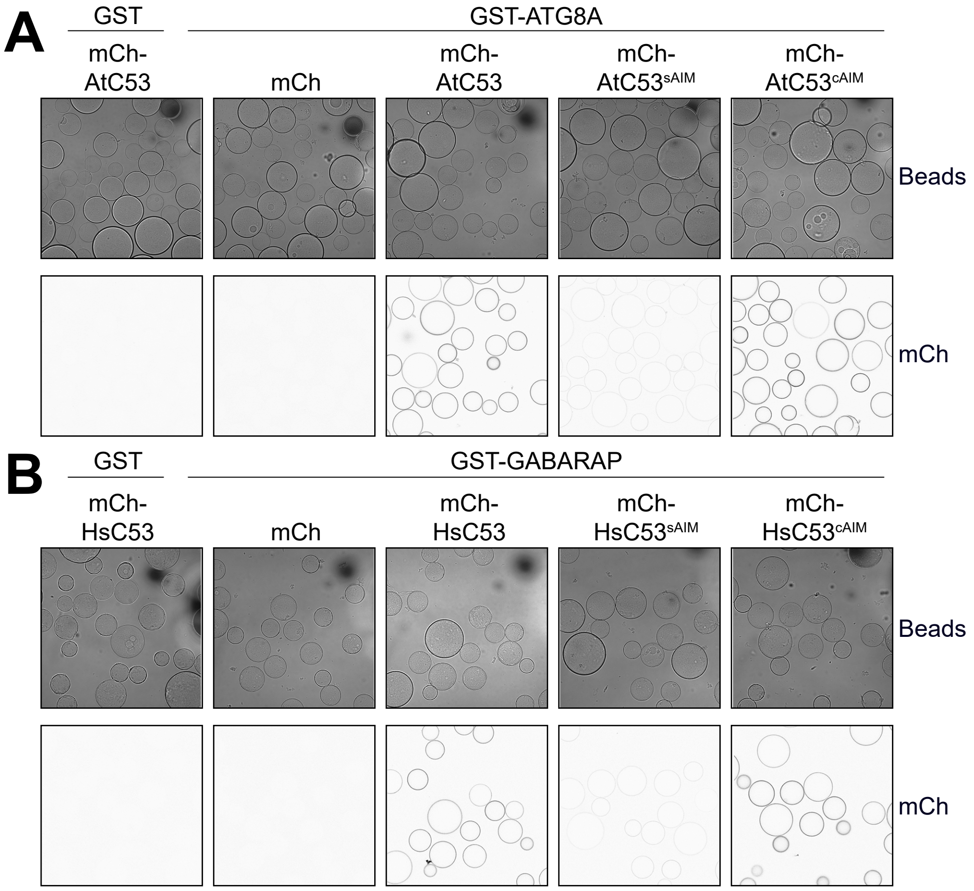

A
GST
GST-ATG8A
mCh-AtC53
mCh
mCh-AtC53
mCh-AtC53sAIM
mCh-AtC53cAIM
Beads
mCh
B
GST
GST-GABARAP
mCh-HsC53
mCh
mCh-HsC53
mCh-HsC53sAIM
mCh-HsC53cAIM
Beads
mCh

Appendix Figure S10. Microscopy-based protein–protein interaction assays showing $C53^{cAIM}$ has increased affinity towards ATG8 or GABARAP.

(A, B) Representative confocal images (inverted grayscale) for each condition from Figure 5 D, E are shown.

Fig. S11

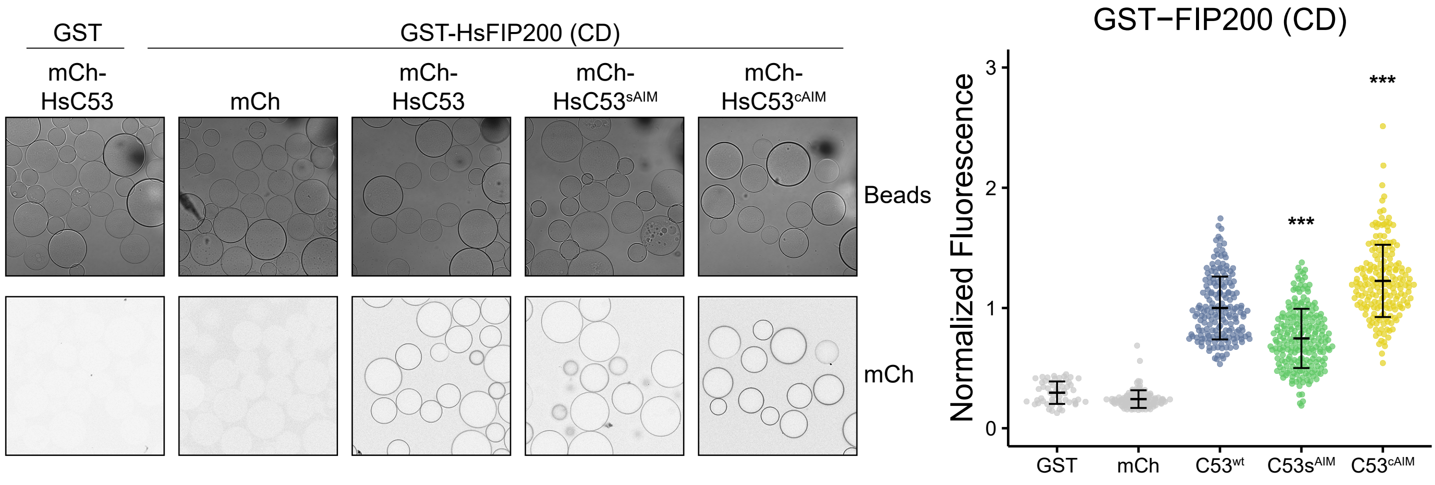

GST
GST-HsFIP200 (CD)
mCh-HsC53
mCh
mCh-HsC53
mCh-HsC53sAIM
mCh-HsC53cAIM
Beads
mCh
GST−FIP200 (CD)
Normalized Fluorescence
0
1
2
3
***
***
GST
mCh
C53wt
C53sAIM
C53cAIM

Appendix Fig. S11. C53-HsFIP200 Claw domain (CD) interaction is also mediated by the sAIM sequences and strengthened by sAIM to cAIM conversion.

Glutathione-sepharose beads were prepared by incubating them with GST-FIP200 CD. The pre-assembled beads were then washed and mixed with 1 μM of HsC53, 1 μM of $HsC53^{sAIM}$ or 1 μM of $HsC53^{cAIM}$ mutants. The beads were then imaged using a confocal microscope. *Left Panel,* representative confocal images (inverted grayscale) for each condition are shown. *Right panel*, normalized fluorescence is shown for each condition with the mean (± SD) of 2 independent replicates containing 2 technical replicates. Unpaired two-samples Wilcoxon test with continuity correction was performed to analyze the differences between wild type and mutants. ***, p-value < 0.001. Total number of beads, mean, median, standard deviation and p-values are reported in Supplementary data S1.

Fig. S12

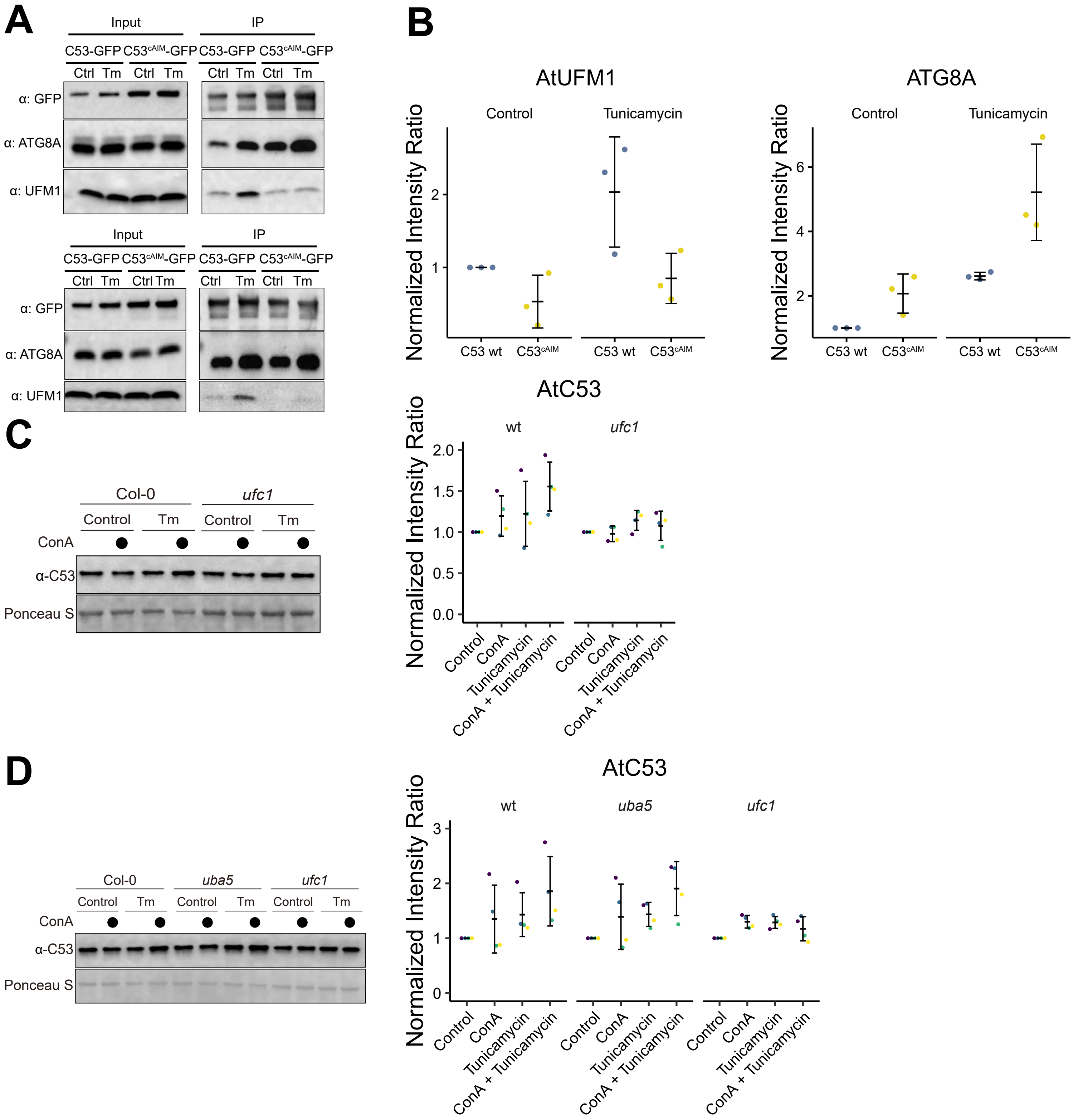
A
Input
IP
C53-GFP
C53cAIM-GFP
Ctrl
Tm
α: GFP
α: ATG8A
α: UFM1
B
AtUFM1
ATG8A
Control
Tunicamycin
Normalized Intensity Ratio
C53 wt
C53cAIM
AtC53
wt
ufc1
Control
ConA
Tunicamycin
ConA + Tunicamycin
C
Col-0
ConA
α-C53
Ponceau S
D
uba5

Appendix Figure S12. *In vivo* pull downs showing sAIM to cAIM conversion strengthens C53-ATG8 association while weakens C53-UFM1 association.

(A) Biological replicates of representative experiment shown in Figure 5F. 6-day old *Arabidopsis* seedlings expressing AtC53-GFP, AtC53^{cAIM}-GFP in *c53* mutant background were incubated in liquid 1/2 MS medium with 1% sucrose supplemented with DMSO as control (Ctrl) or 10 µg/ml tunicamycin (Tm) for 16 hours and used for co-immunoprecipitation. Lysates were incubated with GFP-Trap Magnetic Agarose, input and bound proteins were detected by immunoblotting using the respective antibodies as indicated. **(B) Quantification of blots in (Fig. 5F, Fig. S12A).** UFM1 and ATG8 protein levels that associate with AtC53-GFP or AtC53^{cAIM}-GFP are shown. Bars represent the mean (± SD) of 3 biological replicates (BR). **(C, D) Autophagic flux analysis of endogenous C53 protein using AtC53 antibody.** Western blot analysis of Col-0, *uba5* and *ufc1* Arabidopsis seedlings incubated in either control, or 10 µg/ml tunicamycin for 16 hrs. In addition, each treatment was supplemented with or without 1 µm concanamycin A (conA) as indicated to visualize vacuolar degradation. *Right panel*, quantification of the relative intensities of the protein bands were normalized for the total protein level of the lysate, relative protein intensity in the non-Tm and non-ConA treated control is set as 1 for each genotype. Average C53 levels and SD for n = 4 are shown.

Supplementary Data S1. Total number of beads, mean, median, standard deviation and p-values of the microscopy-based protein-protein interaction assays are reported.

Supplementary Data S2. Fiji macro and agarose bead model for automatic quantification.
