# Supplementary material for: Shuffled ATG8 interacting motifs form an ancestral bridge between UFMylation and autophagy
Source: EMBO J. 2023 Feb 10;42(10):e112053. doi: 10.15252/embj.2022112053 (PMC10183829; doi:10.15252/embj.2022112053)
Supplement: Supplementary file 5 [file EMBJ-42-e112053-s003.pdf]

THE EMBO JOURNAL

# Shuffled ATG8 interacting motifs form an ancestral bridge between UFMylation and autophagy

Lorenzo Picchianti, Victor Sanchez de Medina Hernandez, Ni Zhan, Nicholas Irwin, Roan Groh, Madlen Stephani, Harald Hornegger, Rebecca Beveridge, Justyna Sawa-Makarska, Thomas Lendl, Nenad Grujic, Christin Naumann, Sascha Martens, Thomas Richards, Tim Clausen, Silvia Ramundo, Elif Karagoz, and Yasin Dagdas
**DOI 10.15252/embj.2022112053**

*Corresponding author(s): Yasin Dagdas (yasin.dagdas@gmi.oeaw.ac.at) , Elif Karagoz (elif.karagoez@univie.ac.at)*



## Review Timeline:

| | |
|---|---|
| Transfer from Review Commons: | 6th Jul 22 |
| Editorial Decision: | 12th Jul 22 |
| Revision Received: | 14th Nov 22 |
| Editorial Decision: | 14th Dec 22 |
| Revision Received: | 20th Dec 22 |
| Editorial Decision: | 11th Jan 23 |
| Revision Received: | 12th Jan 23 |
| Accepted: | 18th Jan 23 |



Review COMMONS

*Editor: William Teale*

**Transaction Report: This manuscript was transferred to The EMBO JOURNAL following peer review at Review Commons.**

# Review #1

## 1. Evidence, reproducibility and clarity:

### Evidence, reproducibility and clarity (Required)

In this manuscript Picchianti et al. provide novel insights into the interaction of C53 with UFM1 and ATG8. Initially, the authors show that protein modification by UFM1 exists in the unicellular organism Chlamydomonas reinhardtii. To that end they demonstrated that pure Chlamydomonas UBA5, UFC1 and UFM1 proteins, can charge UFC1. Then, they showed that C53 interacts with ATG8 and UFM1. Specifically, they found that the sAIM are essential for the interaction with UFM1, while substituting this motif with canonical AIM prevents the binding of UFM1 but not of ATG8. Since binding of C53 to ATG8 recruits the autophagy machinery, the authors suggest that ufmylation of RPL26 releases UFM1 from C53 which allows the binding of ATG8. Overall, the authors demonstrate that C53 that forms a complex with UFL1 connects between protein ufmylation and autophagy by its ability to bind both UBLs.

Here the authors revisited the assumption that only multicellular organisms have the UFM1 system. Using bioinformatic tools they show that it exists also in unicellular organism. Also, they show that in some organisms the E3 complex UFL1, UFBP1 and C53 exist but not UBA5, UFC1 or UFM1. This is a very interesting observation that suggests an additional role for this complex. In Fig 1C the authors show that in Chlamydomonas RPL26 undergoes ufmylation. Please use IP against RPL26 and then a blot with anti UFM1. From the current experiment it is not clear how the authors know that this is indeed RPL26 that undergoes ufmylation

In the second part of the manuscript the authors characterize the interaction of C53 with ATG8 and UFM1. This is a continuation of their previous published work (Stephani et al, 2020) . Here the reviewer thinks that further data on the binding of these proteins to C53 is required. Specifically, defining the Kd of these interactions using ITC or other biophysical method can contribute to the study.

Under normal condition the authors suggest that C53 binds UFM1 and this keeps it inactive. The reviewer thinks that this claim needs further support. Using IP (maybe with crosslinker) the author can show that C53, in normal conditions, bind more UFM1 than ATG8. Also, since the interaction of UFM1 to C53 is noncovalent, it will be nice to show how alternations in UFM1 expression levels can affect the activation of C53.

Finally, the authors suggest that ufmylation of RPL26 allows binding of ATG8 to C53 and this, in turn, leads to C53 activation. Can the authors show that in cells lacking UBA5, under normal condition or with Tunicamycin treatment, ATG8 does not activate C53 due to the fact that UFM1 does not leave C53.

## 2. Significance:

### Significance (Required)

This manuscript advances our understanding of the connection of ufmylation to autophagy which is mediated by C53.

## 3. How much time do you estimate the authors will need to complete the suggested revisions:

### Estimated time to Complete Revisions (Required)

### (Decision Recommendation)

Between 1 and 3 months

## 4. Review Commons values the work of reviewers and encourages them to get credit for their work. Select 'Yes' below to register your reviewing activity at Publons; note that the content of your review will not be visible on Publons.

### Reviewer Publons

No

# Review #2

## 1. Evidence, reproducibility and clarity:

### Evidence, reproducibility and clarity (Required)

The manuscript from Picchianti et al. seeks to define the role of CDK5RAP3 (hereinafter referred as C53) during autophagy and its interplay with UFMylation. Together with UFL1 and DDRGK1, C53 is a component of a trimeric UFM1 E3 ligase complex that modifies the 60S ribosomal protein RPL26 at the endoplasmic reticulum (ER) surface upon ribosomal stalling (among other proposed functions that are not addressed). Several previous studies have implicated the UFMylation pathway in autophagy or ER-phagy although a non-autophagic fate for UFM1-tagged ribosomal subunits has also been reported.

A previous study from the same authors (PMID: 32851973) identified an intrinsically disorder region (IDR) in C53 that is necessary and sufficient for interaction between C53 and autophagy receptor, ATG8. They reported that this IDR comprises four non canonical ATG8 interacting motifs (AIM), named shuffled AIMs (sAIMs) and showed that combinatorial mutagenesis of sAIM1, sAIM2, and sAIM3 abrogates ATG8 binding. A similar effect was observed for plant C53, though an additional canonical AIM (cAIM) in the C53 IDR had to be mutated to completely abolish C53 and ATG8 interaction. The earlier study reported that C53 IDR also interacts with UFM1, and this interaction can be disrupted in vitro by adding increasing concentration of ATG8, suggesting that ATG8 and UFM1 may compete with one another for C53 binding.

The present paper attempts to build on this previous work by using phylogenomics to infer a co-evolutionary relationship between UFMylation machinery and sAIMs in C53, which the authors argue, constitutes further evidence of the primary importance of a role for UFMylation in ER homeostasis. The manuscript includes a lot of biochemical data using variations of in vitro and in vivo pull-down experiments to define the roles of individual AIMs in mediating the binding of C53 to ATG8 and to UFM1. They also use NMR spectroscopy in an attempt to define the structural basis of the UFM1 and ATG8 binding to C53, concluding that plant C53 interacts with UFM1 mainly through sAIM1, while interaction with ATG8 requires cAIM as well as sAIM1 and sAIM2. Finally, the authors attempt to contextualize these findings by conducting studies on Arabidopsis mutants, showing that replacing sAIMs with cAIMs causes increases sensitivity to ER stress and apparently increases formation of C53 intracellular puncta that may colocalize with ATG8.

From these data the authors concluded that the dual-ATG8 and UFM1 binding of C53 IDR regulates C53 recruitment to autophagosomes in response to ER stress.

**Major Issues:**

1. The phylogenomics analysis conclusion that UFM1 is common in unicellular

lineages and did not evolve in multicellular eukaryotes is not novel, as another comprehensive analysis of UFM1 phylogeny, published eight years ago - in 2014 - by Grau-Bové et al. (PMID: 25525215), also reported that UFM1, UBA5, UFC1, UFL1 and UFSP2 were likely present in LECA and lost in Fungi. Although the phylogenomic analysis by Picchianti et al. is also extended to DDRGK1 and C53 proteins, and some parasitic and algal lineages, their findings are incremental. Their proposed coevolution of sAIM and UFM1 is based on presence-absence correlation observed within five species (i.e., Albugo candida, Albuco laibachii, Piromyces finnis, Neocallimastix californiae, Anaeromyces robustus). However, this coevolutionary relationship must be further investigated by substantially increasing the taxonomic sampling within the UFM1-lacking group.

2. The manuscript presents an overwhelming amount of biochemical and structural data obtained from a variety of protein binding techniques (i.e., NMR spectroscopy, in vitro GST-pulldown, fluorescence microscopy-based on-bead binding assays, and native mass-spectrometry). The results are poorly explained and not organized in a logical manner. Moreover, no attempt was made to explain the rationale behind using one technique over the other or how one method complements another to build a stronger conclusion than any individual approach. Given that none of the methods employed report quantitative measurement of binding affinities between C53 IDR and UFM1 or ATG8, it is not clear how the data presented in this manuscript contribute to our understanding of the proposed competition model for UFM1 and ATG8 binding to C53 IDR. To conclude that an interaction is "stronger" or "weaker" it is necessary to measure equilibrium binding constants. Fortunately, there are suitable techniques, including surface plasmon resonance (SPR), microscale thermophoresis (MST), fluorescence anisotropy, or calorimetry that are available to dissect these complex competitive binding interactions and to build models.

3. The NMR studies have the potential to dissect the types of dynamic binding inherent in unstructured proteins. However, the abundant NMR data presented combined with the aforementioned binding studies, remarkably, do not seem to significantly advance our understanding of how the system is organized or even how UFM1 and ATG8 bind C53, beyond the rather vague and somewhat circular conclusion stated in the abstract: "...we confirmed the interaction of UFM1 with the C53 sAIMs and found that UFM1 and ATG8 bound the sAIMs in a different mode." Or on line 165 "Altogether these results suggested that ATG8 and UFM1 bbind the sAIMs withn C54 IDR, albeit in a different manner".

4. The functional assays performed in Arabidopsis do not support the competitive model between UFM1 and ATG8 for binding to C53 during C53-mediated autophagy. The fluorescence microscopy images do not provide convincing evidence of colocalization between C53 and ATG8. In fact, in contrast to the claims made in the text or the quantification, mCherry-C53 fluorescence does not seem to localize in discrete puncta and its signal does not seem to overlap with ATG8A.

**Minor Issues:**

1. The authors might choose to avoid teleological arguments such as (line 135): "As the phylogenomic analysis suggested that eh sAIMs have been retained to mediate C53-UFM1 interaction..."
2. The authors refer on multiple occasions to C53 "autoactivation" without defining what they mean by this. Do they propose that C53 UFMylates itself?.
3. The paper might want to avoid preachy philosophical statements like "Our evolutionary analysis also highlights why we should move beyond yeast and metazoans and instead consider the whole tree of life when using evolutionary arguments to guide biological research." (333-335). While this is indeed a laudable goal, given the rather limited insights from this study, it is unclear how this paper exemplifies the notion.

**Referees cross-commenting**

Referee #2

The challenge in providing a fair review of this manuscript is to clearly define what contributions are novel, significant advances. It is difficult to tell the way the manuscript is written, as it is unclear how the new data - which are voluminous- actually advance the model already put forth by the same authors in two previous publications. It is also unfortunate that the authors overlooked the 2004 phylogenomics paper. There clearly are some new pieces of information here, but the overall increment in knowledge is rather minimal.

Response from Referee #3

I agree that the authors somehow steamroll the reader with a wealth of data. But I think this can be addressed by the authors by requesting a lot more justification and by giving them the opportunity to put the significant advances into their own words. This is, in my opinion, quite doable in course of a revision. Overall I have to say that I am very sympathetic with the cross-eukaryote reactivity approach that the authors have taken. It is quite intriguing.

Response from Referee #2

I agree that the cross-eukaryote approach is intriguing. Shouldn't we be concerned that the 2004 publication already made two of their key points (ie present in LECA, loss in

Fungi). What is the incremental insight from this paper?

I'd appreciate an opinion from an evolutionary biologist as to how strongly one can conclude functional co-evolution from such correlative data, especially given the rather small number of supporting examples. Is it also necessary to consider counter-examples- ie species that have sAIMs but no UFM1 (I believe that they found a few such cases)?

Response from Referee #3

Well with these deep evolutionary questions this is always a challenge. Where does one stop to sample more homologs for one's analyses (one from each supergroup [which are no longer recognised by the community])? In that sense, the authors are right to make the parsimonious base assumption that if X and Y interact in species A and B (no matter how distant they are related) then X and Y interacted in the last common ancestor of A and B. That being said, if I would have designed this study, I would have sampled more broadly for my in vitro cross-eukaryote approach. But also this, I think, could be carried out by the authors in a reasonable timeframe. Specifically, they have now sampled from Amorphea and Archaeplastida, they should add one from TSAR, one Haptista, one Cryptista, and one CRuM. If they synthesised the proteins via a company, they could have the constructs in a few weeks for about 1K Euro - I do not think that this would be an unreasonable request.

## 2. Significance:

### Significance (Required)

Overall, while the manuscript contains an abundance of new data, the overall conclusion of the work, stated in the title: "Shuffled ATG8 interacting motifs form an ancestral bridge between UFMylation and C53-mediated autophagy" does not constitute a significant advance beyond other published phylogenomic analysis (below) and the two previous papers by the same authors, including the 2020 paper "A cross-kingdom conserved ER-phagy receptor maintains endoplasmic reticulum homeostasis during stress (PMID: 32851973)" and the 2021 paper "C53 is a cross-kingdom conserved reticulophagy receptor that bridges the gap between selective autophagy and ribosome stalling at the endoplasmic reticulum PMID: 33164651)". While a regulatory interaction between UFMylation and autophagy is of potential importance, the data in this manuscript do not constitute a major advance and fail to

provide new mechanistic insight to explain the role of C53 IDR in autophagy and its interplay with UFMylation

## 3. How much time do you estimate the authors will need to complete the suggested revisions:

### Estimated time to Complete Revisions (Required)

### (Decision Recommendation)

Cannot tell / Not applicable

## 4. Review Commons values the work of reviewers and encourages them to get credit for their work. Select 'Yes' below to register your reviewing activity at Publons; note that the content of your review will not be visible on Publons.

### Reviewer Publons

No

# Review #3

## 1. Evidence, reproducibility and clarity:

### Evidence, reproducibility and clarity (Required)

Picchianti and colleagues have investigated a conserved molecular framework that orchestrates ER homeostasis via autophagy. For this, they have carried out phylogenomics and large-scale gene family analyses across eukaryote diversity as well as a barrage of molecular lab work.
The amount of work carried out as well as the overall quality of the study is impressive.
I have only a few comments that should be very easy to tackle.

1. Maybe I missed it, but please upload all alignments used for phylogenetics and phylogenomics for reproducibility to e.g. Zenodo, Figshare or other suitable OA databases.
2. "Why these non-canonical motifs were selected during evolution, instead of canonical ATG8 interacting motifs remains unknown" --> Maybe there is no "why" and these were not selected at all. Could be random... drift, non-adaptive constructive neutral evolution. I am not saying that asking "why" in evolutionary biology is wrong. It, however, often does not yield satisfactory answers--or any answer at all.
3. The authors make a case for UFMylation in LECA and I am fully sympathetic with this. However, getting rid of misfoled/problematic proteins and subcellular entities is something that prokaryotes also to a certain degree must have (and still do) master. Are inclusion bodies or export their only answers (I don't know)? Of course, in eukaryotes with all their intracellular complexity this is likely more of an issue. Given the scope of this manuscript (i.e. shedding light on that ancient framework, deep evolutionary roots in eukaryote evolution etc. etc.) it would be very interesting to read the authors thoughts on this and also pinpoint the prokaryote/eukaryote divide in light of the machinery discussed here.

**Referees cross-commenting**

Referee #2

The challenge in providing a fair review of this manuscript is to clearly define what contributions are novel, significant advances. It is difficult to tell the way the manuscript is written, as it is unclear how the new data - which are voluminous- actually advance the model already put forth by the same authors in two previous publications. It is also unfortunate that the authors overlooked the 2004 phylogenomics paper. There clearly are some new pieces of information here, but the overall increment in knowledge is rather minimal.

Response from Referee #3

I agree that the authors somehow steamroll the reader with a wealth of data. But I think this can be addressed by the authors by requesting a lot more justification and by giving them the opportunity to put the significant advances into their own words. This is, in my opinion, quite doable in course of a revision. Overall I have to say that I am very sympathetic with the cross-eukaryote reactivity approach that the authors have taken. It is quite intriguing.

Response from Referee #2

I agree that the cross-eukaryote approach is intriguing. Shouldn't we be concerned that the 2004 publication already made two of their key points (ie present in LECA, loss in Fungi). What is the incremental insight from this paper?

I'd appreciate an opinion from an evolutionary biologist as to how strongly one can conclude functional co-evolution from such correlative data, especially given the rather small number of supporting examples. Is it also necessary to consider counter-examples- ie species that have sAIMs but no UFM1 (I believe that they found a few such cases)?

Response from Referee #3

Well with these deep evolutionary questions this is always a challenge. Where does one stop to sample more homologs for one's analyses (one from each supergroup [which are no longer recognised by the community])? In that sense, the authors are right to make the parsimonious base assumption that if X and Y interact in species A and B (no matter how distant they are related) then X and Y interacted in the last common ancestor of A and B. That being said, if I would have designed this study, I would have sampled more broadly for my in vitro cross-eukaryote approach. But also this, I think, could be carried out by the authors in a reasonable timeframe. Specifically, they have now sampled from Amorphea and Archaeplastida, they should add one from TSAR, one Haptista, one Cryptista, and one CRuM. If they synthesised the proteins via a company, they could have the constructs in a few weeks for about 1K Euro - I do not think that this would be an unreasonable request.

## 2. Significance:

### Significance (Required)

This study not only impresses with the volume of experiments and data, but also the courage to show conservation of a molecular framework by working with such a range of distantly-related eukaryotes.
The results and conclusions from this study should be interesting to anyone working in the broad fields of cellular stress and/or autophagy--both extremely timely topics.

## 3. How much time do you estimate the authors will need to complete the suggested revisions:

### Estimated time to Complete Revisions (Required)

### (Decision Recommendation)

Less than 1 month

## 4. Review Commons values the work of reviewers and encourages them to get credit for their work. Select 'Yes' below to register your reviewing activity at Publons; note that the content of your review will not be visible on Publons.

### Reviewer Publons

Yes

# Revision Plan

**Manuscript number:** RC-2022-01458

**Corresponding authors:** Yasin Dagdas, Elif Karagoz

## 1. General Statements

We are forwarding our manuscript "**Shuffled ATG8 interacting motifs form an ancestral bridge between UFMylation and C53-mediated autophagy**" from ReviewCommons, for consideration for publication in *The EMBO Journal*.

Autophagy of the endoplasmic reticulum (ER-phagy) is a fundamental process that is essential for maintaining cellular homeostasis and quality control. We recently identified a novel mechanism regulating ER-phagy in both plants and animals that is based on the ubiquitin-like protein modifiers ATG8 and UFM1, and the ER-associated protein, C53. Here, we use a combination of evolutionary, biochemical, and physiological experiments to investigate the evolution and regulation of this process. We reveal the dynamic evolution of UFM1 and the ubiquity of C53-mediated autophagy across eukaryotes. Leveraging these results, we then identify an ancestral molecular toggle switch, mediated by shuffled ATG8-interacting motifs (sAIMs), that controls C53-mediated autophagy through competitive binding between UFM1 and ATG8. These findings provide new insights into the evolution of UFM1, reveal a conserved mechanism for the regulation of ER-phagy, and raise new and exciting hypotheses about the diversity and function of the UFMylation pathway. We believe that this work will be of interest to those studying autophagy and cellular stress response but will also serve as an interesting example of the benefits of combining evolutionary analyses with biochemical and cellular experiments.

Our manuscript has been reviewed by three reviewers through *ReviewCommons*, whose comments, and our responses, can be found below. Two of the reviewers (Reviewer 1 and 3) were supportive of our work and its significance whereas Reviewer 2 questioned the novelty of our findings.

Each of the reviewers' comments can be addressed through a few supporting experiments as well as an improved manuscript which clarifies the novelty and significance of our results. While

being supportive of our work, Reviewer 1 requested minor additional experiments to support our mechanistic conclusions and Reviewer 3 suggested that we expand our characterizations of C53 function to additional eukaryotic supergroups. These experiments are straightforward to perform, the materials and protocols to accomplish them are already established, and our overall conclusions are robust to the resulting outcomes.

In contrast, Reviewer 2 did not suggest any additional experiments but rather challenged the novelty of our results as well as some of our interpretations. In particular, Reviewer 2 was uncertain of how our phylogenomic analyses built upon a previous study, published in 2014, which used comparative genomics to identify ubiquitin-related machinery across eukaryotes. Although it was an oversight to not reference this study (we cited a more recent article showing the same results), we were aware of their conclusions that UFMylation was present in the last eukaryotic common ancestor but absent in Fungi. We now clearly outline, both below and within the manuscript, our key phylogenomic results. These were acquired after implementing more advanced and comprehensive comparative genomic searches which allowed us to identify dynamic patterns in UFMylation evolution and permitted co-evolutionary analyses which were not only important for informing our experimental hypotheses but generated new functional questions. Our phylogenomic analyses are also linked to biochemical and physiological data, providing, for the first time, experimental support for our conclusions regarding UFMylation evolution. Similarly, Reviewer 2 suggested that our mechanistic results were an incremental extension of our previous work. Although our current work does of course build on our initial identification of C53-mediated autophagy, this manuscript provides novel insights into the importance and function of this process by revealing its ubiquity across eukaryotes and by characterizing the mechanistic details of its regulation. Ultimately, we disagree with Reviewer 2 but appreciate that this misunderstanding likely resulted from a lack of context and clarity in our manuscript which we have now resolved.

As outlined in detail below, we will address the reviewers concerns through additional experiments, analyses, and improvements to the text.

Thank you for considering our manuscript. We look forward to hearing from you.

**2. Description of the planned revisions**

We thank the reviewers for carefully evaluating our manuscript and for providing us with an opportunity to respond to their suggestions and criticisms. As you can see below in our point-by-point response, we address each of the points raised by the reviewers through the addition of supporting experiments, analyses, and an improved text. Altogether, we think these additional experiments and textual changes will significantly improve the manuscript. Therefore, we would like to thank all the reviewers and editors for their time and input.

The additional experiments that we plan to do to address the reviewers' concerns are in *italic*.

**Point-by-point Response:**

Reviewer #1 (Evidence, reproducibility, and clarity (Required)):

In this manuscript Picchianti et al. provide novel insights into the interaction of C53 with UFM1 and ATG8. Initially, the authors show that protein modification by UFM1 exists in the unicellular organism Chlamydomonas reinhardtii. To that end they demonstrated that pure Chlamydomonas UBA5, UFC1 and UFM1 proteins, can charge UFC1. Then, they showed that C53 interacts with ATG8 and UFM1. Specifically, they found that the sAIM are essential for the interaction with UFM1, while substituting this motif with canonical AIM prevents the binding of UFM1 but not of ATG8. Since binding of C53 to ATG8 recruits the autophagy machinery, the authors suggest that ufmylation of RPL26 releases UFM1 from C53 which allows the binding of ATG8. Overall, the authors demonstrate that C53 that forms a complex with UFL1 connects between protein ufmylation and autophagy by its ability to bind both UBLs.

Here the authors revisited the assumption that only multicellular organisms have the UFM1 system. Using bioinformatic tools they show that it exists also in unicellular organism. Also, they show that in some organisms the E3 complex UFL1, UFBP1 and C53 exist but not UBA5, UFC1 or UFM1. This is a very interesting observation that suggests an additional role for this complex. In Fig 1C the authors show that in Chlamydomonas RPL26 undergoes ufmylation. Please use IP against RPL26 and then a blot with anti UFM1. From the current experiment it is not clear how the authors know that this is indeed RPL26 that undergoes ufmylation

RPL26 is highly conserved across eukaryotes, so by comparing our western blots with previous studies (Walczak et. al., 2019, Wang et al. 2020), we concluded that these bands corresponded to UFMylated RPL26. However, we agree with the reviewer that we need to confirm the identify of RPL26 with additional assays. Since the submission of the manuscript, we tested RPL26 antibodies in Chlamydomonas and showed that they work well. So, *we will update our figure with the confirmation westerns.*

In the second part of the manuscript the authors characterize the interaction of C53 with ATG8 and UFM1. This is a continuation of their previous published work (Stephani et al, 2020). Here the reviewer thinks that further data on the binding of these proteins to C53 is required. Specifically, defining the Kd of these interactions using ITC or other biophysical method can contribute to the study.

We agree with the reviewer. To obtain the $K_D$ values, we will *perform ITC experiments with C53 wild type, a C53 sAIM mutant and a C53 cAIM variant titrated with ATG8 and UFM1.*

Under normal condition the authors suggest that C53 binds UFM1 and this keeps it inactive. The reviewer thinks that this claim needs further support. Using IP (maybe with crosslinker) the author can show that C53, in normal conditions, bind more UFM1 than ATG8. Also, since the interaction of UFM1 to C53 is noncovalent, it will be nice to show how alternations in UFM1 expression levels can affect the activation of C53.

We thank the reviewer for this suggestion. Since the submission of the manuscript, we have obtained UFM1 overexpression lines. *We will pull on C53 using our C53 antibody and check for ATG8 levels in wild type and UFM1 overexpressing lines under normal and stress conditions.* We think this will show how alterations in UFM1 levels can affect C53 activation.

Finally, the authors suggest that ufmylation of RPL26 allows binding of ATG8 to C53 and this, in turn, leads to C53 activation. Can the authors show that in cells lacking UBA5, under normal condition or with Tunicamycin treatment, ATG8 does not activate C53 due to the fact that UFM1 does not leave C53.

In Stephani et al., we showed that C53-mediated autophagy requires the UFMylation machinery. In ufl1 and ddrgk1 mutants, C53 becomes insensitive to ER stress. However, to supplement these results, we will perform *autophagic flux assays using the native C53 antibody to test autophagic degradation of C53 in a uba5 and ufc1 mutant under normal and tunicamycin stress conditions. The uba5 mutant that we have is a knockdown, so that's why we will include the ufc1 mutant in our experiments.*

Reviewer #1 (Significance (Required)):

This manuscript advances our understanding of the connection of ufmylation to autophagy which is mediated by C53.

Thank you!

Reviewer #2 (Evidence, reproducibility, and clarity (Required)):

# Revision Plan

The manuscript from Picchianti et al. seeks to define the role of CDK5RAP3 (hereinafter referred as C53) during autophagy and its interplay with UFMylation. Together with UFL1 and DDRGK1, C53 is a component of a trimeric UFM1 E3 ligase complex that modifies the 60S ribosomal protein RPL26 at the endoplasmic reticulum (ER) surface upon ribosomal stalling (among other proposed functions that are not addressed). Several previous studies have implicated the UFMylation pathway in autophagy or ER-phagy although a non-autophagic fate for UFM1-tagged ribosomal subunits has also been reported.
A previous study from the same authors (PMID: 32851973) identified an intrinsically disorder region (IDR) in C53 that is necessary and sufficient for interaction between C53 and autophagy receptor, ATG8. They reported that this IDR comprises four non canonical ATG8 interacting motifs (AIM), named shuffled AIMs (sAIMs) and showed that combinatorial mutagenesis of sAIM1, sAIM2, and sAIM3 abrogates ATG8 binding. A similar effect was observed for plant C53, though an additional canonical AIM (cAIM) in the C53 IDR had to be mutated to completely abolish C53 and ATG8 interaction. The earlier study reported that C53 IDR also interacts with UFM1, and this interaction can be disrupted in vitro by adding increasing concentration of ATG8, suggesting that ATG8 and UFM1 may compete with one another for C53 binding.

The present paper attempts to build on this previous work by using phylogenomics to infer a co-evolutionary relationship between UFMylation machinery and sAIMs in C53, which the authors argue, constitutes further evidence of the primary importance of a role for UFMylation in ER homeostasis. The manuscript includes a lot of biochemical data using variations of in vitro and in vivo pull-down experiments to define the roles of individual AIMs in mediating the binding of C53 to ATG8 and to UFM1. They also use NMR spectroscopy in an attempt to define the structural basis of the UFM1 and ATG8 binding to C53, concluding that plant C53 interacts with UFM1 mainly through sAIM1, while interaction with ATG8 requires cAIM as well as sAIM1 and sAIM2. Finally, the authors attempt to contextualize these findings by conducting studies on Arabidopsis mutants, showing that replacing sAIMs with cAIMs causes increases sensitivity to ER stress and apparently increases formation of C53 intracellular puncta that may colocalize with ATG8.

From these data the authors concluded that the dual-ATG8 and UFM1 binding of C53 IDR regulates C53 recruitment to autophagosomes in response to ER stress.

Major Issues:
1) The phylogenomics analysis conclusion that UFM1 is common in unicellular lineages and did not evolve in multicellular eukaryotes is not novel, as another comprehensive analysis of UFM1 phylogeny, published eight years ago - in 2014 - by Grau-Bové et al. (PMID: 25525215), also reported that UFM1, UBA5, UFC1, UFL1 and UFSP2 were likely present in LECA and lost in Fungi. Although the phylogenomic analysis by Picchianti et al. is also extended to DDRGK1 and C53 proteins, and some parasitic and algal lineages, their findings are incremental. Their proposed coevolution of sAIM and UFM1 is based on presence-absence correlation observed

within five species (i.e., Albugo candida, Albuco laibachii, Piromyces finnis, Neocallimastix californiae, Anaeromyces robustus). However, this coevolutionary relationship must be further investigated by substantially increasing the taxonomic sampling within the UFM1-lacking group.

We were aware that previous studies had investigated the distribution of UFMylation proteins across eukaryotes and that these analyses had predicted the presence of UFMylation in LECA and subsequent loss in Fungi. We included a more recent citation noting this (Tsaban et al. 2021) but apologise for not citing Grau-Bové et al. (2014), which we have now included. We must emphasize that our results are not incremental. Although we had made a point of emphasizing the presence of UFM1 in LECA, this was to counter a recent and highly cited paper in the field which claimed that UFMylation evolved in plants and animals (Walczak et al. 2019). Below we note the novel and important results from our phylogenomic analyses:

1. We used improved taxonomic sampling and more advanced comparative genomics methods to identify UFMylation components sensitively and specifically across eukaryotes. This involved the inclusion of additional eukaryotic genomes, phylogenetic annotation of orthologs, and genomic searches to complement proteome predictions. These methods are essential for accurately identifying UFMylation components and yield more robust results than using sequence similarity clustering (Tsaban et al. 2021) or un-curated Pfam HMMER search results (Grau-Bové et al. 2014).

2. By placing our UFMylation reconstructions in a modern phylogenetic context we were not only able to support previous observations which noted the presence of UFM1 in LECA and its loss in Fungi (Grau-Bové et al. 2014) and *Plasmodium* (Tsaban et al. 2021), but also to identify novel patterns in the evolution of UFMylation. This included the observation of recurrent losses in diverse but trophically-related lineages (such as algae and parasites) and revealed the retention of certain UFMylation components in the absence of UFM1. We identified the frequent co-retention of UFL1 and DDRGK1 following UFM1 loss in multiple eukaryotic groups, including Fungi, which were previously thought to be devoid of UFMylation machinery. These previously uncharacterized patterns, suggest that these proteins could have alternative functions and may be functionally associated with life history. These results therefore expand on and add complexity to our understanding of the evolution of UFMylation.

3. By conducting a comprehensive and accurate survey of UFMylation components we were able to use our data to examine co-evolutionary trends between C53 and UFM1, which would have been incomplete and inaccurate using previously curated datasets. As the reviewer noted, only five species were identified that encoded C53 but lacked UFM1. This is not a reflection of insufficient taxon sampling, but rather the strong co-evolution between C53 and UFM1 (i.e., when UFM1 is lost, C53 is almost always lost as well). We attempted to identify additional cases by searching hundreds of fungal and oomycete genomes as well as those from other eukaryotes, but no other species were found. We agree with the reviewer that additional taxa would have

made our analyses stronger, but importantly, we do not rely on genomic correlations to infer function. Rather, we use these correlations to generate functional hypotheses which we then tested experimentally. In this way, we do not rely on the strength of our correlations.

We have now revised the manuscript to include additional context (including citations) and have improved the clarity of the text to better convey the novelty of our findings.

2) The manuscript presents an overwhelming amount of biochemical and structural data obtained from a variety of protein binding techniques (i.e., NMR spectroscopy, in vitro GST-pulldown, fluorescence microscopy-based on-bead binding assays, and native mass-spectrometry). The results are poorly explained and not organized in a logical manner. Moreover, no attempt was made to explain the rationale behind using one technique over the other or how one method complements another to build a stronger conclusion than any individual approach. Given that none of the methods employed report quantitative measurement of binding affinities between C53 IDR and UFM1 or ATG8, it is not clear how the data presented in this manuscript contribute to our understanding of the proposed competition model for UFM1 and ATG8 binding to C53 IDR. To conclude that an interaction is "stronger" or "weaker" it is necessary to measure equilibrium binding constants. Fortunately, there are suitable techniques, including surface plasmon resonance (SPR), microscale thermophoresis (MST), fluorescence anisotropy, or calorimetry that are available to dissect these complex competitive binding interactions and to build models.

We thank the reviewer for their suggestion. Although we attempted to describe the rationale behind each experiment (please see the line 135-137; on-bead binding assays, line154-157; NMR, 177-181), we agree that the volume of data and variety of techniques warrants additional explanation. We will revise the manuscript to further explain our rationale for using each of the different approaches. As we noted above in our response to reviewer 1, we will also *perform relevant ITC binding assays to quantify the interaction between C53, ATG8, and UFM1*.

3) The NMR studies have the potential to dissect the types of dynamic binding inherent in unstructured proteins. However, the abundant NMR data presented combined with the aforementioned binding studies, remarkably, do not seem to significantly advance our understanding of how the system is organized or even how UFM1 and ATG8 bind C53, beyond the rather vague and somewhat circular conclusion stated in the abstract: "...we confirmed the interaction of UFM1 with the C53 sAIMs and found that UFM1 and ATG8 bound the sAIMs in a different mode." Or on line 165 "Altogether these results suggested that ATG8 and UFM1 bind the sAIMs withn C54 IDR, albeit in a different manner".

We agree that NMR has the potential to dissect the complex binding interactions between UFM1, ATG8, and C53, but disagree with the reviewer's interpretation that our NMR data fail to achieve this. To sum up, our NMR data:

1. Revealed the structural basis of the interaction of C53-IDR with ATG8 and UFM1 at atomic resolution by showing that UFM1 binds preferentially to sAIM1 in the fast-intermediate exchange [Fig.4 and Fig. S7B], instead ATG8 binds cAIM in the slow-intermediate exchange, and once cAIM is occupied, it binds sAIM1,2 with lower affinity in the fast-intermediate exchange (Fig.4 and Fig.S7D).

2. Determined conformational changes in C53 IDR upon binding of ATG8, but not UFM1 (Fig.S7E), which lead to increased dynamics in distinct regions in C53 IDR. These data could explain how binding of first ATG8 would trigger C53-dependent recruitment of the tripartite complex to autophagosomes.

3. Identified how UFM1 binds to atypical hydrophobic patch in C53 sAIM, similar to what was shown for the UBA5 LIR/UFIM.

To sum up, our results shed light on how both UBLs interact with C53, being sAIM1 the highest affinity binding site for UFM1 while ATG8 binds cAIM preferentially before occupying sAIM1,2.

*To provide more detailed information on the atomic details of the interaction between C53 and the UBLs, we will perform molecular docking studies by using the restraints obtained from the experimental NMR data.*

4) The functional assays performed in Arabidopsis do not support the competitive model between UFM1 and ATG8 for binding to C53 during C53-mediated autophagy. The fluorescence microscopy images do not provide convincing evidence of colocalization between C53 and ATG8. In fact, in contrast to the claims made in the text or the quantification, mCherry-C53 fluorescence does not seem to localize in discrete puncta and its signal does not seem to overlap with ATG8A.

We disagree with the reviewer's interpretation of these results although we acknowledge that there is some subtlety in interpreting the co-localization data. Importantly, Arabidopsis has 9 ATG8 isoforms and C53 can bind to most of them with varying affinities (see Stephani et al). Because of this, we do not expect C53 puncta to fully colocalize with ATG8A puncta. Additionally, the C53 puncta are smaller and more subtle than ATG8 puncta, which label the entire autophagosome. *To reconcile this, we will quantify the effect by performing colocalization analyses under normal and stress conditions. We will also upload all the raw images as supporting material, so that anyone can independently assess our images.*

Minor Issues:

1. The authors might choose to avoid teleological arguments such as (line 135): "As the phylogenomic analysis suggested that eh sAIMs have been retained to mediate C53-UFM1 interaction..."

We thank the reviewer for this suggestion and will modify the text accordingly.

2. The authors refer on multiple occasions to C53 "autoactivation" without defining what they mean by this. Do they propose that C53 UFMylates itself?.

We refer to C53 activity as the ability to recruit the autophagy machinery and initiate cargo sequestration and degradation in the vacuole. We attempted to explain this in lines 57-61 but we will reword it more clearly, as suggested by the reviewer.

3. The paper might want to avoid preachy philosophical statements like "Our evolutionary analysis also highlights why we should move beyond yeast and metazoans and instead consider the whole tree of life when using evolutionary arguments to guide biological research." (333-335). While this is indeed a laudable goal, given the rather limited insights from this study, it is unclear how this paper exemplifies the notion.

We added this statement as we were intrigued by our evolutionary analyses' ability to link C53 to UFM1 (an association which took years to identify experimentally) and generate useful functional hypotheses about the interaction between C53 sAIMs and UFM1. As we mentioned above, we also wanted to highlight this point in reference to a recent prominent study in the field which drew conclusions after only considering animals, plants, and fungi (Walczak et al., 2019). We believe this point is important and underappreciated by some cell biologists, but we will modify the text to make it more generic: "This work highlights the utility of using evolutionary analyses and eukaryotic diversity to generate mechanistic hypotheses for cellular processes".

Reviewer #2 (Significance (Required)):

Overall, while the manuscript contains an abundance of new data, the overall conclusion of the work, stated in the title: "Shuffled ATG8 interacting motifs form an ancestral bridge between UFMylation and C53-mediated autophagy" does not constitute a significant advance beyond other published phylogenomic analysis (below) and the two previous papers by the same authors, including the 2020 paper "A cross-kingdom conserved ER-phagy receptor maintains endoplasmic reticulum homeostasis during stress (PMID: 32851973)" and the 2021 paper "C53 is a cross-kingdom conserved reticulophagy receptor that bridges the gap between selective autophagy and ribosome stalling at the endoplasmic reticulum PMID: 33164651)". While a regulatory interaction between UFMylation and autophagy is of potential importance, the data in this manuscript do not constitute a major advance and fail to provide new mechanistic insight to explain the role of C53 IDR in autophagy and its interplay with UFMylation

We disagree with the reviewer's suggestion that our work does not constitute a significant advance. We outlined above in detail the novel insights that were obtained from our phylogenomic analysis which involved using improved methods to reveal a much more dynamic and informative picture of UFMylation evolution than has been described previously. Likewise, this manuscript builds substantially on our previous mechanistic work. In our 2020 paper (which is summarized in the mentioned 2021 review article), we identified C53 as an ER-associated protein that binds ATG8 through sAIMs and interacts with the phagophore after RPL26 UFMylation. This work linked C53 activity to ER-phagy and highlighted its importance in plant and animal stress response. However, key questions remained unanswered prior to our current work such as whether this mechanism is conserved across eukaryotes, especially in unicellular species, how C53 activity is regulated, and how UFM1 and ATG8 interact with C53. Our current manuscript builds on this work with the following key results:

1. We use a combination of phylogenomic and experimental analyses to demonstrate that C53 function is conserved across eukaryotes.

2. We reveal a mechanism whereby UFM1 and ATG8 compete for binding at the sAIMs in the C53 IDR and characterize how each of these ubiquitin-like proteins interacts in an alternative way (see the NMR results described above).

3. We show how the sAIMs are required for the regulation of C53-mediated autophagy and reveal the importance of UFM1-ATG8 competition in preventing C53 autoactivation, which causes unnecessary autophagic degradation and impairs cellular stress responses.

These insights are fundamental for understanding the mechanisms regulating C53-mediated autophagy which were unknown before this work. We will therefore adjust our manuscript to more clearly and explicitly explain how our data build on previous observations so that the novelty and significance of our results are clearer.

Reviewer #3 (Evidence, reproducibility and clarity (Required)):

Picchianti and colleagues have investigated a conserved molecular framework that orchestrates ER homeostasis via autophagy. For this, they have carried out phylogenomics and large-scale gene family analyses across eukaryote diversity as well as a barrage of molecular lab work. The amount of work carried out as well as the overall quality of the study is impressive.

Thank you!

I have only a few comments that should be very easy to tackle.

(1) Maybe I missed it, but please upload all alignments used for phylogenetics and phylogenomics for reproducibility to e.g. Zenodo, Figshare or other suitable OA databases.

We included the alignments in the supplementary data, but as suggested, we will *upload all the source data including the scripts and the alignments to Zenodo.*

(2) "Why these non-canonical motifs were selected during evolution, instead of canonical ATG8 interacting motifs remains unknown" --> Maybe there is no "why" and these were not selected at all. Could be random... drift, non-adaptive constructive neutral evolution. I am not saying that asking "why" in evolutionary biology is wrong. It, however, often does not yield satisfactory answers--or any answer at all.

The reviewer is completely right that "why" is not the right way to frame an evolutionary question. Thank you for pointing this out. We will revise the text and make sure that we remove these kinds of deterministic statements.

(3) The authors make a case for UFMylation in LECA and I am fully sympathetic with this. However, getting rid of misfoled/problematic proteins and subcellular entities is something that prokaryotes also to a certain degree must have (and still do) master. Are inclusion bodies or export their only answers (I don't know)? Of course, in eukaryotes with all their intracellular complexity this is likely more of an issue. Given the scope of this manuscript (i.e. shedding light on that ancient framework, deep evolutionary roots in eukaryote evolution etc. etc.) it would be very interesting to read the authors thoughts on this and also pinpoint the prokaryote/eukaryote divide in light of the machinery discussed here.

Thank you for this suggestion. We did indeed check whether any of the UFMylation machinery were present in prokaryotes and only found homologs of UFSP2. These results are consistent with Grau-Bové et al. (2014) who conducted an equivalent analysis and concluded that UFMylation machinery were derived during eukaryogenesis. We will make reference to this in the revised manuscript.

Reviewer #3 (Significance (Required)):

This study not only impresses with the volume of experiments and data, but also the courage to show conservation of a molecular framework by working with such a range of distantly-related eukaryotes.
The results and conclusions from this study should be interesting to anyone working in the broad fields of cellular stress and/or autophagy--both extremely timely topics.

We thank the reviewer for understanding our take-home message and the advances made. We especially thank the reviewer for understanding the challenge of connecting *in silico* genomic data with *in vivo* and *in vitro* experiments.

CROSS-CONSULTATION COMMENTS

Referee #2
The challenge in providing a fair review of this manuscript is to clearly define what contributions are novel, significant advances. It is difficult to tell the way the manuscript is written, as it is unclear how the new data - which are voluminous- actually advance the model already put forth by the same authors in two previous publications. It is also unfortunate that the authors overlooked the 2004 phylogenomics paper. There clearly are some new pieces of information here, but the overall increment in knowledge is rather minimal.

Response from Referee #3
I agree that the authors somehow steamroll the reader with a wealth of data. But I think this can be addressed by the authors by requesting a lot more justification and by giving them the opportunity to put the significant advances into their own words. This is, in my opinion, quite doable in course of a revision. Overall I have to say that I am very sympathetic with the cross-eukaryote reactivity approach that the authors have taken. It is quite intriguing.

We thank the reviewers for this useful exchange. We agree that our manuscript was not clear enough to emphasize the novelty of our results which likely resulted from the volume and diversity of the experiments and analyses that were presented. We have now revised the manuscript to improve the context and rationale for the study, the intent and hypotheses behind each experiment, and the novel results and insights obtained in each section.

Response from Referee #2
I agree that the cross-eukaryote approach is intriguing. Shouldn't we be concerned that the 2004 publication already made two of their key points (ie present in LECA, loss in Fungi). What is the incremental insight from this paper?

I'd appreciate an opinion from an evolutionary biologist as to how strongly one can conclude functional co-evolution from such correlative data, especially given the rather small number of supporting examples. Is it also necessary to consider counter-examples- ie species that have sAIMs but no UFM1 (I believe that they found a few such cases)?

Importantly, we do not conclude functional co-evolution from our correlative data. Instead, we used these correlations to generate hypotheses that we tested with various experiments in different model systems. For example, the apparent correlation between C53 sAIMs and UFM1 prompted us to test whether or not UFM1 and sAIMs interact. Regardless of sample size or

statistical significance, phylogenomic analyses can never demonstrate functional links, only correlations, which is why we combined these two approaches. Although only a few species encoded C53 without UFM1, each of these contained C53 cAIMs and lacked sAIMs (Figure 2c). There are species with UFM1 that lack C53 but this makes sense as UFM1 is used in other processes besides ER-phagy. We have revised the text to make our approach and reliance on certain data clearer.

Response from Referee #3
Well with these deep evolutionary questions this is always a challenge. Where does one stop to sample more homologs for one's analyses (one from each supergroup [which are no longer recognised by the community])? In that sense, the authors are right to make the parsimonious base assumption that if X and Y interact in species A and B (no matter how distant they are related) then X and Y interacted in the last common ancestor of A and B. That being said, if I would have designed this study, I would have sampled more broadly for my in vitro cross-eukaryote approach. But also this, I think, could be carried out by the authors in a reasonable timeframe. Specifically, they have now sampled from Amorphea and Archaeplastida, they should add one from TSAR, one Haptista, one Cryptista, and one CRuM. If they synthesised the proteins via a company, they could have the constructs in a few weeks for about 1K Euro - I do not think that this would be an unreasonable request.

We agree that testing C53 function in additional species would strengthen our understanding of the conservation of this pathway across eukaryotes, as it cannot be assumed that orthologous proteins will function in the same way across all species. To our knowledge there is no other work showing experimentally that the UFMylation pathway is working in a single-celled organism. We focussed our efforts on the unicellular green alga, *Chlamydomonas* due to its relative experimental tractability. However, testing this was not trivial as it required us to establish expression and purification protocols, isolate *Chlamydomonas* mutants, optimize physiological stress assays, and perform the experiments.

Nevertheless, we agree that we could expand our *in vitro* assays with C53 orthologs from additional species. As suggested by reviewer 3, *we will now synthesize 6 more C53 isoforms from two TSAR representatives (the alveolate, Tetrahymena thermophila, and the stramenopile, Phytophthora sojae), as well as a representative from Haptista (Emiliania), Cryptista (Guillardia), Diplomonada (Trypanosoma), and CRuMs (Rigifila). We will test their interaction with human and plant ATG8 and UFM1 proteins.* We have also added two species from CRuMs into our phylogenomic analysis.

# Revision Plan

**The list of experiments that we can do to address the reviewer's concerns:**

1. Repeat experiment in Figure 1C probing with $\alpha$-RPL26.

2. To calculate $K_D$ values, perform ITC experiments with C53 wild-type, C53 sAIM mutant and C53 cAIM variant titrated with ATG8 and UFM1.

3. Perform CoIP experiments using C53 antibody in wild type and UFM1 overexpressing lines and detect for ATG8 association, under normal and stress conditions.

4. We will test autophagic degradation of C53 in *uba5* and *ufc1* mutants under normal and tunicamycin stress conditions by performing autophagic flux assays using the native C53 antibody

5. Molecular docking studies to see C53's structural rearrangements leading to ATG8 and UFM1 binding.

6. Figures from co-localization experiments in Figure 5G will be revisited and we will perform additional co-localization analyses such as Pearson coefficient under normal and stress conditions. We will also upload all the raw images as supporting material, so that anyone can independently assess our images.

7. We will upload all the source data for phylogenomic analyses, including scripts and alignments to Zenodo.

8. Test the interaction of 6 newly synthesised C53 isoforms from: (1) an alveolate (*tsAr*, *Ciliate*), (2) a stramenopile (*tSar*, *Phaeodactylum*), (3) a haptophyte (*Emiliania*), (4) a cryptophyte (*Guillardia*), (5) a diplomonad (*Trypanosoma*) and (6) a *CrRuM* with human and plant ATG8 and UFM1 proteins.

**1st Editorial Decision** **12th Jul 2022**

Dear Yasin,

Thank you for transferring your manuscript with Review Commons referee reports and responses to The EMBO Journal.

Given the referees' positive recommendations, I would like to invite you to submit a revised version of the manuscript, addressing the comments of all three reviewers. I should add that it is EMBO Journal policy to allow only a single round of revision, and acceptance of your manuscript will therefore depend on the completeness of your responses in this revised version.

The editorial team here at EMBO Journal is very interested in your study and I am, in principle, fully happy with your revision plan. However, it would be really good to explore with you whether there are ways for you easily to extend the physiological analysis of the plants you generated for figure 5. I think this could be a very efficient way of nicely rounding off the study. Maybe we could Zoom to discuss this next week? As I say, though, this would really only be icing on the cake.

When preparing your letter of response to the referees' comments, please bear in mind that this will form part of the Review Process File, and will therefore be available online to the community. For more details on our Transparent Editorial Process, please visit our website.

We generally allow three months as standard revision time. As a matter of policy, competing manuscripts published during this period will not negatively impact on our assessment of the conceptual advance presented by your study. However, we request that you contact the editor as soon as possible upon publication of any related work, to discuss how to proceed. Should you foresee a problem in meeting this three-month deadline, please let us know in advance and we may be able to grant an extension.

Thank you for the opportunity to consider your work for publication. I look forward to your revision.

Yours sincerely,

William

William Teale, PhD
Editor
The EMBO Journal
w.teale@embojournal.org

Instructions for preparing your revised manuscript:

Please make sure you upload a letter of response to the referees' comments together with the revised manuscript.

Please also check that the title and abstract of the manuscript are brief, yet explicit, even to non-specialists.

When assembling figures, please refer to our figure preparation guideline in order to ensure proper formatting and readability in print as well as on screen:
https://bit.ly/EMBOPressFigurePreparationGuideline
See also figure legend guidelines: https://www.embopress.org/page/journal/14602075/authorguide#figureformat

IMPORTANT: When you send the revision we will require
- a point-by-point response to the referees' comments, with a detailed description of the changes made (as a word file).
- a word file of the manuscript text.
- individual production quality figure files (one file per figure)
- a complete author checklist, which you can download from our author guidelines (https://www.embopress.org/page/journal/14602075/authorguide).
- Expanded View files (replacing Supplementary Information)
Please see out instructions to authors
https://www.embopress.org/page/journal/14602075/authorguide#expandedview

Please remember: Digital image enhancement is acceptable practice, as long as it accurately represents the original data and

conforms to community standards. If a figure has been subjected to significant electronic manipulation, this must be noted in the figure legend or in the 'Materials and Methods' section. The editors reserve the right to request original versions of figures and the original images that were used to assemble the figure.

Further information is available in our Guide For Authors: https://www.embopress.org/page/journal/14602075/authorguide

We realize that it is difficult to revise to a specific deadline. In the interest of protecting the conceptual advance provided by the work, we recommend a revision within 3 months (10th Oct 2022). Please discuss the revision progress ahead of this time with the editor if you require more time to complete the revisions. Use the link below to submit your revision:

Link Not Available

------------------------------------------------

***

Rev_Com_number: RC-2022-01458
New_manu_number: EMBOJ-2022-112053
Corr_author: Dagdas
Title: Shuffled ATG8 interacting motifs form an ancestral bridge between UFMylation and C53-mediated autophagy

**Manuscript number:** EMBOJ-2022-112053
**Corresponding authors:** Yasin Dagdas, Elif Karagoz

**1. General Statements**

We are re-submitting our revised manuscript "**Shuffled ATG8 interacting motifs form an ancestral bridge between UFMylation and C53-mediated autophagy**" for consideration for publication in *The EMBO Journal*.

Autophagy of the endoplasmic reticulum (ER-phagy) is a fundamental process that is essential for maintaining cellular homeostasis and quality control. We recently identified a novel mechanism regulating ER-phagy in both plants and animals that is based on the ubiquitin-like protein modifiers ATG8 and UFM1, and the ER-associated protein, C53. Here, we use a combination of evolutionary, biochemical, structural and physiological experiments to investigate the evolution and regulation of this process. We reveal the dynamic evolution of UFM1 and the ubiquity of C53-mediated autophagy across eukaryotes. Leveraging these results, we then identify an ancestral molecular toggle switch, mediated by shuffled ATG8-interacting motifs (sAIMs), that controls C53-mediated autophagy through competitive binding between UFM1 and ATG8. These findings provide new insights into the evolution of UFM1, reveal a conserved mechanism for the regulation of ER-phagy, and raise new and exciting hypotheses about the diversity and function of the UFMylation pathway. We believe that this work will be of interest to those studying autophagy and cellular stress response but will also serve as an interesting example of the benefits of combining evolutionary analyses with biochemical and cellular experiments.

Our manuscript has been reviewed by three reviewers through *ReviewCommons*, whose comments, and our responses, can be found below. Two of the reviewers (Reviewer 1 and 3) were supportive of our work and its significance whereas Reviewer 2 questioned the novelty of our findings.

**We have addressed each of the reviewers' comments with new experiments and revised the text to clarify the novelty and significance of our results.** While being supportive of our work, Reviewer 1 requested minor additional experiments to support our mechanistic conclusions and Reviewer 3 suggested that we expand our characterizations of C53 function to additional eukaryotic supergroups.

We have performed all of these experiments. They certainly improved our manuscript and supported our previous conclusions.

In contrast, Reviewer 2 did not suggest any additional experiments but rather challenged the novelty of our results as well as some of our interpretations. In particular, Reviewer 2 was uncertain of how our phylogenomic analyses built upon a previous study, published in 2014, which used comparative genomics to identify ubiquitin-related machinery across eukaryotes. Although it was an oversight to not reference this study (we cited a more recent article showing the same results), we were aware of their conclusions that UFMylation was present in the last eukaryotic common ancestor but absent in Fungi. We now clearly outline our key phylogenomic results. These were acquired after implementing more advanced and comprehensive comparative genomic searches which allowed us to identify dynamic patterns in UFMylation evolution and permitted co-evolutionary analyses which were not only important for informing our experimental hypotheses but generated new functional questions. Our phylogenomic analyses are also linked to biochemical and physiological data, providing, for the first time, experimental support for our conclusions regarding UFMylation evolution. Similarly, Reviewer 2 suggested that our mechanistic results were an incremental extension of our previous work. Although our current work does of course build on our initial identification of C53-mediated autophagy, this manuscript provides novel insights into the importance and function of this process by revealing its ubiquity across eukaryotes and by characterizing the mechanistic details of its regulation. Ultimately, we disagree with Reviewer 2 but appreciate that this misunderstanding likely resulted from a lack of context and clarity in our manuscript which we have now resolved.

As outlined in detail below, we addressed the reviewers concerns through additional experiments, analyses, and improvements to the text.

Thank you for considering our manuscript. We look forward to hearing from you.

**2. Summary of the revisions**

We thank the reviewers for carefully evaluating our manuscript and for providing us with an opportunity to respond to their suggestions and criticisms. As you can see below in our point-by-point response, we address each of the points raised by the reviewers through the addition of supporting experiments, analyses, and an improved text. Altogether, we think these additional experiments and textual changes significantly improved the manuscript. Therefore, we would like to thank all the reviewers and editors for their time and input.

**Point-by-point Response:**

Reviewer #1 (Evidence, reproducibility, and clarity (Required)):

In this manuscript Picchianti et al. provide novel insights into the interaction of C53 with UFM1 and ATG8. Initially, the authors show that protein modification by UFM1 exists in the unicellular organism Chlamydomonas reinhardtii. To that end they demonstrated that pure Chlamydomonas UBA5, UFC1 and UFM1 proteins, can charge UFC1. Then, they showed that C53 interacts with ATG8 and UFM1. Specifically, they found that the sAIM are essential for the interaction with UFM1, while substituting this motif with canonical AIM prevents the binding of UFM1 but not of ATG8. Since binding of C53 to ATG8 recruits the autophagy machinery, the authors suggest that ufmylation of RPL26 releases UFM1 from C53 which allows the binding of ATG8. Overall, the authors demonstrate that C53 that forms a complex with UFL1 connects between protein ufmylation and autophagy by its ability to bind both UBLs.

Here the authors revisited the assumption that only multicellular organisms have the UFM1 system. Using bioinformatic tools they show that it exists also in unicellular organism. Also, they show that in some organisms the E3 complex UFL1, UFBP1 and C53 exist but not UBA5, UFC1 or UFM1. This is a very interesting observation that suggests an additional role for this complex. In Fig 1C the authors show that in Chlamydomonas RPL26 undergoes ufmylation. Please use IP against RPL26 and then a blot with anti UFM1. From the current experiment it is not clear how the authors know that this is indeed RPL26 that undergoes ufmylation

RPL26 is highly conserved across eukaryotes, so by comparing our western blots with previous studies (Walczak et. al., 2019, Wang et al. 2020), we concluded that these bands corresponded to UFMylated RPL26. However, we agree with the reviewer that we need to confirm the identify of RPL26 with additional assays. To confirm these are UFMylated RPL26 bands, we performed two additional experiments: (1) We isolated ribosomes biochemically, by sucrose cushions and performed western blots using UFM1 antibody. As presented in Appendix Fig. S3D, the total lysate and purified ribosomes had the same pattern: UFM1 bands were induced upon ER-stress and absent in *ufl1* mutants; (2) We performed mass spectrometry experiments to detect RPL26 UFMylation. As presented in Appendix Fig. S3D, mass spectrometry also confirmed the UFMylation of RPL26.

In the second part of the manuscript the authors characterize the interaction of C53 with ATG8 and UFM1. This is a continuation of their previous published work (Stephani et al, 2020). Here the reviewer

thinks that further data on the binding of these proteins to C53 is required. Specifically, defining the Kd of these interactions using ITC or other biophysical method can contribute to the study.

We agree with the reviewer. To obtain the $K_D$ values, we performed ITC experiments with C53 *wild type*, a $C53^{sAIM}$ mutant and a $C53^{cAIM}$ variant titrated with ATG8 and UFM1. As presented in Appendix Fig. S6, ITC experiments did not detect an interaction between UFM1 and C53, under the experimental conditions we tested. However, we were able to confirm the stronger affinity of ATG8 to $C53^{cAIM}$ in five independent ITC replicates. These experiments are presented in Fig. EV5. Complementarily, we characterized the interaction of the peptides derived from sAIMs and cAIM in AtC53 with AtUFM1 and AtATG8, using fluorescence anisotropy experiments presented in Fig. S8E and S9D.

Under normal condition the authors suggest that C53 binds UFM1 and this keeps it inactive. The reviewer thinks that this claim needs further support. Using IP (maybe with crosslinker) the author can show that C53, in normal conditions, bind more UFM1 than ATG8. Also, since the interaction of UFM1 to C53 is noncovalent, it will be nice to show how alternations in UFM1 expression levels can affect the activation of C53.

We thank the reviewer for this suggestion. We opted for *in vitro* competition of UFM1 by ATG8 for two reasons: As we mentioned above UFM1's affinity is lower compared to ATG8, so we would need very high concentrations of UFM1 to reach saturation in *in vitro* experiments. Unfortunately, purified UFM1 started to aggregate when we tried to concentrate it at molarities that we need for those experiments. For *in vivo* experiments, we would need to overexpress all the UFMylation machinery to increase the local concentration of UFM1 at stalled ribosomes. This experiment is not feasible within the time frame of a revision. However, we strongly believe sAIM to cAIM conversion addresses the reviewer's concern. By changing sAIMs to cAIMs, we are only changing the affinity of C53 towards UFM1 and ATG8. We think those experiments are better controlled and provide more precise information compared to a UFM1 overexpression experiment.

Finally, the authors suggest that ufmylation of RPL26 allows binding of ATG8 to C53 and this, in turn, leads to C53 activation. Can the authors show that in cells lacking UBA5, under normal condition or with Tunicamycin treatment, ATG8 does not activate C53 due to the fact that UFM1 does not leave C53.

We thank the reviewer for this suggestion. We now performed C53 flux experiments in ufc1 mutants and showed that C53 flux is impaired in these mutants. We also performed flux experiments in a knockdown of UBA5. Unlike ufc1 mutant, we did not observe a significant change in C53 flux in uba5 knockdowns, suggesting the remaining protein is enough for sustained C53 flux. These experiments are presented in Appendix Fig. S12 C, D.

Reviewer #1 (Significance (Required)):

This manuscript advances our understanding of the connection of ufmylation to autophagy which is mediated by C53.

Thank you!

Reviewer #2 (Evidence, reproducibility, and clarity (Required)):

The manuscript from Picchianti et al. seeks to define the role of CDK5RAP3 (hereinafter referred as C53) during autophagy and its interplay with UFMylation. Together with UFL1 and DDRGK1, C53 is a component of a trimeric UFM1 E3 ligase complex that modifies the 60S ribosomal protein RPL26 at the endoplasmic reticulum (ER) surface upon ribosomal stalling (among other proposed functions that are not addressed). Several previous studies have implicated the UFMylation pathway in autophagy or ER-phagy although a non-autophagic fate for UFM1-tagged ribosomal subunits has also been reported.
A previous study from the same authors (PMID: 32851973) identified an intrinsically disorder region (IDR) in C53 that is necessary and sufficient for interaction between C53 and autophagy receptor, ATG8. They reported that this IDR comprises four non canonical ATG8 interacting motifs (AIM), named shuffled AIMs (sAIMs) and showed that combinatorial mutagenesis of sAIM1, sAIM2, and sAIM3 abrogates ATG8 binding. A similar effect was observed for plant C53, though an additional canonical AIM (cAIM) in the C53 IDR had to be mutated to completely abolish C53 and ATG8 interaction. The earlier study reported that C53 IDR also interacts with UFM1, and this interaction can be disrupted in vitro by adding increasing concentration of ATG8, suggesting that ATG8 and UFM1 may compete with one another for C53 binding.

The present paper attempts to build on this previous work by using phylogenomics to infer a co-evolutionary relationship between UFMylation machinery and sAIMs in C53, which the authors argue, constitutes further evidence of the primary importance of a role for UFMylation in ER homeostasis. The manuscript includes a lot of biochemical data using variations of in vitro and in vivo pull-down experiments to define the roles of individual AIMs in mediating the binding of C53 to ATG8 and to UFM1. They also use NMR spectroscopy in an attempt to define the structural basis of the UFM1 and ATG8 binding to C53, concluding that plant C53 interacts with UFM1 mainly through sAIM1, while interaction with ATG8 requires cAIM as well as sAIM1 and sAIM2. Finally, the authors attempt to contextualize these findings by conducting studies on Arabidopsis mutants, showing that replacing sAIMs with cAIMs causes increases sensitivity to ER stress and apparently increases formation of C53 intracellular puncta that may colocalize with ATG8.

From these data the authors concluded that the dual-ATG8 and UFM1 binding of C53 IDR regulates C53 recruitment to autophagosomes in response to ER stress.

Major Issues:
1) The phylogenomics analysis conclusion that UFM1 is common in unicellular lineages and did not evolve in multicellular eukaryotes is not novel, as another comprehensive analysis of UFM1 phylogeny, published eight years ago - in 2014 - by Grau-Bové et al. (PMID: 25525215), also reported that UFM1, UBA5, UFC1, UFL1 and UFSP2 were likely present in LECA and lost in Fungi. Although the phylogenomic analysis by Picchianti et al. is also extended to DDRGK1 and C53 proteins, and some

parasitic and algal lineages, their findings are incremental. Their proposed coevolution of sAIM and UFM1 is based on presence-absence correlation observed within five species (i.e., Albugo candida, Albuco laibachii, Piromyces finnis, Neocallimastix californiae, Anaeromyces robustus). However, this coevolutionary relationship must be further investigated by substantially increasing the taxonomic sampling within the UFM1-lacking group.

We were aware that previous studies had investigated the distribution of UFMylation proteins across eukaryotes and that these analyses had predicted the presence of UFMylation in LECA and subsequent loss in Fungi. We included a more recent citation noting this (Tsaban et al. 2021) but apologise for not citing Grau-Bové et al. (2014), which we have now included. We must emphasize that our results are not incremental. Although we had made a point of emphasizing the presence of UFM1 in LECA, this was to counter a recent and highly cited paper in the field which claimed that UFMylation evolved in plants and animals (Walczak et al. 2019). Below we note the novel and important results from our phylogenomic analyses:

1. We used improved taxonomic sampling and more advanced comparative genomics methods to identify UFMylation components sensitively and specifically across eukaryotes. This involved the inclusion of additional eukaryotic genomes, phylogenetic annotation of orthologs, and genomic searches to complement proteome predictions. These methods are essential for accurately identifying UFMylation components and yield more robust results than using sequence similarity clustering (Tsaban et al. 2021) or un-curated Pfam HMMER search results (Grau-Bové et al. 2014).

2. By placing our UFMylation reconstructions in a modern phylogenetic context, we were not only able to support previous observations which noted the presence of UFM1 in LECA and its loss in Fungi (Grau-Bové et al. 2014) and *Plasmodium* (Tsaban et al. 2021), but also to identify novel patterns in the evolution of UFMylation. This included the observation of recurrent losses in diverse but trophically-related lineages (such as algae and parasites) and revealed the retention of certain UFMylation components in the absence of UFM1. We identified the frequent co-retention of UFL1 and DDRGK1 following UFM1 loss in multiple eukaryotic groups, including Fungi, which were previously thought to be devoid of UFMylation machinery. These previously uncharacterized patterns, suggest that these proteins could have alternative functions and may be functionally associated with life history. These results therefore expand on and add complexity to our understanding of the evolution of UFMylation.

3. By conducting a comprehensive and accurate survey of UFMylation components we were able to use our data to examine co-evolutionary trends between C53 and UFM1, which would have been incomplete and inaccurate using previously curated datasets. As the reviewer noted, only five species were identified that encoded C53 but lacked UFM1. This is not a reflection of insufficient taxon sampling, but rather the strong co-evolution between C53 and UFM1 (i.e., when UFM1 is lost, C53 is almost always lost as well). We attempted to identify additional cases by searching hundreds of fungal and oomycete genomes as well as those from other eukaryotes, but no other species were found. We agree with the reviewer that additional taxa would have made our analyses stronger, but importantly, we do not rely on genomic correlations to infer function. Rather, we use these correlations to generate

functional hypotheses which we then tested experimentally. In this way, we do not rely on the strength of our correlations.

We have now revised the manuscript to include additional context (including citations) and have improved the clarity of the text to better convey the novelty of our findings.

2) The manuscript presents an overwhelming amount of biochemical and structural data obtained from a variety of protein binding techniques (i.e., NMR spectroscopy, in vitro GST-pulldown, fluorescence microscopy-based on-bead binding assays, and native mass-spectrometry). The results are poorly explained and not organized in a logical manner. Moreover, no attempt was made to explain the rationale behind using one technique over the other or how one method complements another to build a stronger conclusion than any individual approach. Given that none of the methods employed report quantitative measurement of binding affinities between C53 IDR and UFM1 or ATG8, it is not clear how the data presented in this manuscript contribute to our understanding of the proposed competition model for UFM1 and ATG8 binding to C53 IDR. To conclude that an interaction is "stronger" or "weaker" it is necessary to measure equilibrium binding constants. Fortunately, there are suitable techniques, including surface plasmon resonance (SPR), microscale thermophoresis (MST), fluorescence anisotropy, or calorimetry that are available to dissect these complex competitive binding interactions and to build models.

We thank the reviewer for their suggestion. Although we attempted to describe the rationale behind each experiment, we agree that the volume of data and variety of techniques warrants additional explanation. We now revised the manuscript to further explain our rationale for using each of the different approaches. As we noted above in our response to reviewer 1, we have also performed relevant ITC binding assays, now presented in Fig. EV5.

3) The NMR studies have the potential to dissect the types of dynamic binding inherent in unstructured proteins. However, the abundant NMR data presented combined with the aforementioned binding studies, remarkably, do not seem to significantly advance our understanding of how the system is organized or even how UFM1 and ATG8 bind C53, beyond the rather vague and somewhat circular conclusion stated in the abstract: "...we confirmed the interaction of UFM1 with the C53 sAIMs and found that UFM1 and ATG8 bound the sAIMs in a different mode." Or on line 165 "Altogether these results suggested that ATG8 and UFM1 bind the sAIMs withn C54 IDR, albeit in a different manner".

We agree that NMR has the potential to dissect the complex binding interactions between UFM1, ATG8, and C53, but disagree with the reviewer's interpretation that our NMR data fail to achieve this. To sum up, our NMR data:

1. Revealed the structural basis of the interaction of C53-IDR with ATG8 and UFM1 at atomic resolution by showing that UFM1 binds preferentially to sAIM1 in the fast-intermediate exchange [Fig.4 and Appendix Fig. S8], instead ATG8 binds cAIM in the slow-intermediate exchange, and once cAIM is

occupied, it binds sAIM1,2 with lower affinity in the fast-intermediate exchange (Fig.4 and Appendix Fig.S9).

2. Determined conformational changes in C53 IDR upon binding of ATG8, but not UFM1 (Appendix Fig.S8D, S9B), which lead to increased dynamics in distinct regions in C53 IDR. These data could explain how binding of first ATG8 would trigger C53-dependent recruitment of the tripartite complex to autophagosomes.

3. Identified how UFM1 binds to atypical hydrophobic patch in C53 sAIM, similar to what was shown for the UBA5 LIR/UFIM.

To sum up, our results shed light on how both UBLs interact with C53, being sAIM1 the highest affinity binding site for UFM1 while ATG8 binds cAIM preferentially before occupying sAIM1,2.

4) The functional assays performed in Arabidopsis do not support the competitive model between UFM1 and ATG8 for binding to C53 during C53-mediated autophagy. The fluorescence microscopy images do not provide convincing evidence of colocalization between C53 and ATG8. In fact, in contrast to the claims made in the text or the quantification, mCherry-C53 fluorescence does not seem to localize in discrete puncta and its signal does not seem to overlap with ATG8A.

We disagree with the reviewer's interpretation of these results although we acknowledge that there is some subtlety in interpreting the co-localization data. Importantly, Arabidopsis has 9 ATG8 isoforms and C53 can bind to most of them with varying affinities (see Stephani et al). Because of this, we do not expect C53 puncta to fully colocalize with ATG8A puncta. Additionally, the C53 puncta are smaller and more subtle than ATG8 puncta, which label the entire autophagosome. We now revised our result with optimal background subtraction parameters, the colocalization are very clear in our current version. These results are presented in Fig. 5G. We have also uploaded all the raw data linked to each figure that are readily accessible via DOI: https://doi.org/10.5281/zenodo.7313984

Minor Issues:
1. The authors might choose to avoid teleological arguments such as (line 135): "As the phylogenomic analysis suggested that eh sAIMs have been retained to mediate C53-UFM1 interaction..."

We thank the reviewer for this suggestion and modified the text accordingly.

2. The authors refer on multiple occasions to C53 "autoactivation" without defining what they mean by this. Do they propose that C53 UFMylates itself?.

We refer to C53 activity as the ability to recruit the autophagy machinery and initiate cargo sequestration and degradation in the vacuole. We revised the text by changing autoactivation with initiation of the C53-mediated autophagy.

3. The paper might want to avoid preachy philosophical statements like "Our evolutionary analysis also highlights why we should move beyond yeast and metazoans and instead consider the whole tree of life when using evolutionary arguments to guide biological research." (333-335). While this is indeed a laudable goal, given the rather limited insights from this study, it is unclear how this paper exemplifies the notion.

We added this statement as we were intrigued by our evolutionary analyses' ability to link C53 to UFM1 (an association which took years to identify experimentally) and generate useful functional hypotheses about the interaction between C53 sAIMs and UFM1. As we mentioned above, we also wanted to highlight this point in reference to a recent prominent study in the field which drew conclusions after only considering animals, plants, and fungi (Walczak et al., 2019). We believe this point is important and underappreciated by some cell biologists, but we modified the text as suggested by the reviewer.

Reviewer #2 (Significance (Required)):

Overall, while the manuscript contains an abundance of new data, the overall conclusion of the work, stated in the title: "Shuffled ATG8 interacting motifs form an ancestral bridge between UFMylation and C53-mediated autophagy" does not constitute a significant advance beyond other published phylogenomic analysis (below) and the two previous papers by the same authors, including the 2020 paper "A cross-kingdom conserved ER-phagy receptor maintains endoplasmic reticulum homeostasis during stress (PMID: 32851973)" and the 2021 paper "C53 is a cross-kingdom conserved reticulophagy receptor that bridges the gap between selective autophagy and ribosome stalling at the endoplasmic reticulum PMID: 33164651)". While a regulatory interaction between UFMylation and autophagy is of potential importance, the data in this manuscript do not constitute a major advance and fail to provide new mechanistic insight to explain the role of C53 IDR in autophagy and its interplay with UFMylation

We disagree with the reviewer's suggestion that our work does not constitute a significant advance. We outlined above in detail the novel insights that were obtained from our phylogenomic analysis which involved using improved methods to reveal a much more dynamic and informative picture of UFMylation evolution than has been described previously. Likewise, this manuscript builds substantially on our previous mechanistic work. In our 2020 paper (which is summarized in the mentioned 2021 review article), we identified C53 as an ER-associated protein that binds ATG8 through sAIMs and interacts with the phagophore after RPL26 UFMylation. This work linked C53 activity to ER-phagy and highlighted its importance in plant and animal stress response. However, key questions remained unanswered prior to our current work such as whether this mechanism is conserved across eukaryotes, especially in unicellular species, how C53 activity is regulated, and how UFM1 and ATG8 interact with C53. Our current manuscript builds on this work with the following key results:

1. We use a combination of phylogenomic and experimental analyses to demonstrate that C53 function is conserved across eukaryotes.

2. We reveal a mechanism whereby UFM1 and ATG8 compete for binding at the sAIMs in the C53 IDR and characterize how each of these ubiquitin-like proteins interacts in an alternative way (see the NMR results described above).

3. We show how the sAIMs are required for the regulation of C53-mediated autophagy and reveal the importance of UFM1-ATG8 competition in preventing C53 autoactivation, which causes unnecessary autophagic degradation and impairs cellular stress responses.

These insights are fundamental for understanding the mechanisms regulating C53-mediated autophagy which were unknown before this work. We now adjusted our manuscript to more clearly and explicitly explain how our data build on previous observations, so that the novelty and significance of our results are clearer.

Reviewer #3 (Evidence, reproducibility and clarity (Required)):

Picchianti and colleagues have investigated a conserved molecular framework that orchestrates ER homeostasis via autophagy. For this, they have carried out phylogenomics and large-scale gene family analyses across eukaryote diversity as well as a barrage of molecular lab work.
The amount of work carried out as well as the overall quality of the study is impressive.

Thank you!

I have only a few comments that should be very easy to tackle.

(1) Maybe I missed it, but please upload all alignments used for phylogenetics and phylogenomics for reproducibility to e.g. Zenodo, Figshare or other suitable OA databases.

We included the alignments in the supplementary data, but as suggested, we have now uploaded all the source data including the scripts and the alignments to Zenodo, DOI: https://doi.org/10.5281/zenodo.7313984

(2) "Why these non-canonical motifs were selected during evolution, instead of canonical ATG8 interacting motifs remains unknown" --> Maybe there is no "why" and these were not selected at all. Could be random... drift, non-adaptive constructive neutral evolution. I am not saying that asking "why" in evolutionary biology is wrong. It, however, often does not yield satisfactory answers--or any answer at all.

The reviewer is completely right that "why" is not the right way to frame an evolutionary question. Thank you for pointing this out. We have revised the text and removed these kinds of deterministic statements.

(3) The authors make a case for UFMylation in LECA and I am fully sympathetic with this. However, getting rid of misfoled/problematic proteins and subcellular entities is something that prokaryotes also to a certain degree must have (and still do) master. Are inclusion bodies or export their only answers (I don't know)? Of course, in eukaryotes with all their intracellular complexity this is likely more of an issue. Given the scope of this manuscript (i.e., shedding light on that ancient framework, deep evolutionary roots in eukaryote evolution etc. etc.) it would be very interesting to read the authors thoughts on this and also pinpoint the prokaryote/eukaryote divide in light of the machinery discussed here.

Thank you for this interesting suggestion. To address this comment, we searched prokaryotic reference proteomes using our UFMylation HMMs and identified homologs for UBA5, UFL1, and DDRGK1 whereas UFC1, UFSP2, C53, and UFM1 are strictly eukaryotic. Interestingly, UFL1 and DDRGK1 were largely restricted to Asgard archaea suggesting that the UFL1-DDRGK1 complex may have had a pre-eukaryotic function prior to the origin of UFM1. This may explain the retention of UFL1 and DDRGK1 in various eukaryotic lineages after the loss of UFM1. We have described these results and have added the phylogenies as a supplemental figure (Appendix Fig. S1).

Reviewer #3 (Significance (Required)):

This study not only impresses with the volume of experiments and data, but also the courage to show conservation of a molecular framework by working with such a range of distantly-related eukaryotes.
The results and conclusions from this study should be interesting to anyone working in the broad fields of cellular stress and/or autophagy--both extremely timely topics.

We thank the reviewer for understanding our take-home message and the advances made. We especially thank the reviewer for understanding the challenge of connecting *in silico* genomic data with *in vivo* and *in vitro* experiments.

CROSS-CONSULTATION COMMENTS

Referee #2
The challenge in providing a fair review of this manuscript is to clearly define what contributions are novel, significant advances. It is difficult to tell the way the manuscript is written, as it is unclear how the new data - which are voluminous- actually advance the model already put forth by the same authors in two previous publications. It is also unfortunate that the authors overlooked the 2004 phylogenomics paper. There clearly are some new pieces of information here, but the overall increment in knowledge is rather minimal.

Response from Referee #3
I agree that the authors somehow steamroll the reader with a wealth of data. But I think this can be addressed by the authors by requesting a lot more justification and by giving them the opportunity to put the significant advances into their own words. This is, in my opinion, quite doable in course of a

revision. Overall I have to say that I am very sympathetic with the cross-eukaryote reactivity approach that the authors have taken. It is quite intriguing.

We thank the reviewers for this useful exchange. We agree that our manuscript was not clear enough to emphasize the novelty of our results which likely resulted from the volume and diversity of the experiments and analyses that were presented. We have now revised the manuscript to improve the context and rationale for the study, the intent and hypotheses behind each experiment, and the novel results and insights obtained in each section.

Response from Referee #2
I agree that the cross-eukaryote approach is intriguing. Shouldn't we be concerned that the 2004 publication already made two of their key points (ie present in LECA, loss in Fungi). What is the incremental insight from this paper?

I'd appreciate an opinion from an evolutionary biologist as to how strongly one can conclude functional co-evolution from such correlative data, especially given the rather small number of supporting examples. Is it also necessary to consider counter-examples- ie species that have sAIMs but no UFM1 (I believe that they found a few such cases)?

Importantly, we do not conclude functional co-evolution from our correlative data. Instead, we used these correlations to generate hypotheses that we tested with various experiments in different model systems. For example, the apparent correlation between C53 sAIMs and UFM1 prompted us to test whether or not UFM1 and sAIMs interact. Regardless of sample size or statistical significance, phylogenomic analyses can never demonstrate functional links, only correlations, which is why we combined these two approaches. Although only a few species encoded C53 without UFM1, each of these contained C53 cAIMs and lacked sAIMs (Fig. 2C). There are species with UFM1 that lack C53 but this makes sense as UFM1 is used in other processes besides ER-phagy. We have revised the text to make our approach and reliance on certain data clearer.

Response from Referee #3
Well with these deep evolutionary questions this is always a challenge. Where does one stop to sample more homologs for one's analyses (one from each supergroup [which are no longer recognised by the community])? In that sense, the authors are right to make the parsimonious base assumption that if X and Y interact in species A and B (no matter how distant they are related) then X and Y interacted in the last common ancestor of A and B. That being said, if I would have designed this study, I would have sampled more broadly for my in vitro cross-eukaryote approach. But also this, I think, could be carried out by the authors in a reasonable timeframe. Specifically, they have now sampled from Amorphea and Archaeplastida, they should add one from TSAR, one Haptista, one Cryptista, and one CRuM. If they synthesised the proteins via a company, they could have the constructs in a few weeks for about 1K Euro - I do not think that this would be an unreasonable request.

We agree that testing C53 function in additional species would strengthen our understanding of the conservation of this pathway across eukaryotes, as it cannot be assumed that orthologous proteins will function in the same way across all species. To our knowledge there is no other work showing experimentally that the UFMylation pathway is working in a single-celled organism. We focussed our efforts on the unicellular green alga, *Chlamydomonas* due to its relative experimental tractability. However, testing this was not trivial as it required us to establish expression and purification protocols, isolate *Chlamydomonas* mutants, optimize physiological stress assays, and perform the experiments.

Nevertheless, we agree that we could expand our *in vitro* assays with C53 orthologs from additional species. As suggested by reviewer 3, we now synthesized 7 more C53 isoforms from two TSAR representatives (the alveolate, Tetrahymena thermophila, and the stramenopile, Phytophthora sojae), as well as a representative from Haptista (Emiliania), Cryptista (Guillardia), Diplomonada (Trypanosoma), Amoebozoa (Dictyostelium), and CRuMs (Rigifila). We have tested their interaction with human and plant ATG8 and UFM1 proteins. We have also added two species from CRuMs into our phylogenomic analysis. All of these C53 isoforms interacted with ATG8 and UFM1. These results are presented in Fig. EV2.

**The list of experiments that we performed to address the reviewer's concerns:**

1. Confirmation of RPL26 UFMylation in Chlamydomonas.

Comparison of UFM1 western patterns in total lysates and purified ribosomes (presented in Appendix Fig. S3D) and detection of RPL26 UFMylation using mass spectrometry (presented in Appendix Fig. S3D).

2. To calculate $K_D$ values, perform ITC experiments with C53 wild-type, C53 sAIM mutant and C53 cAIM variant titrated with ATG8 and UFM1.

We performed ITC experiments with C53 and mutants titrated with either ATG8 (Fig. EV5) or UFM1 (Appendix Fig. S6). Regarding C53 titrations with ATG8, the ITC experiments validated the results obtained with previously done microscopy-based on-bead protein-protein interaction assays. However, we did not detect binding between UFM1 and C53 by ITC. For this reason, we used the microscopy-based assays to capture very low affinity, avidity-driven interactions and clearly explained our rationale in the text.

3. Perform CoIP experiments using C53 antibody in wild type and UFM1 overexpressing lines and detect for ATG8 association, under normal and stress conditions.

We performed CoIP experiments using endogenous C53. However, we noticed that the AtC53 antibody we produced is not suitable for IP. We were not able to detect the interaction between C53 and ATG8. In fact, in Fig. RP1, ATG8 could be detected in the input in all samples (red asterisk), but corresponding band could not be detected in the IP sample.

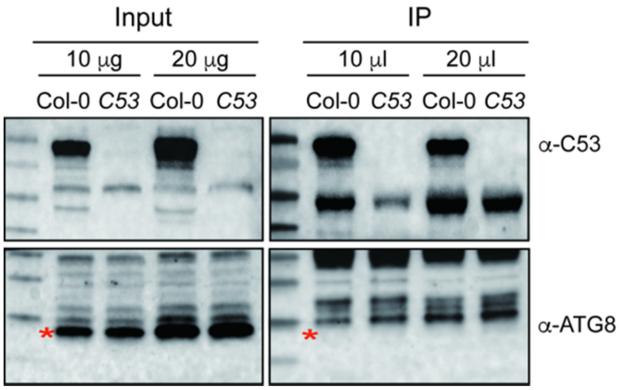


**Figure revision plan 1. *In vivo* co-immunoprecipitation analysis of extracts of Arabidopsis Col-0 and *c53* seedlings using endogenous AtC53 antibody.** Plant extracts were incubated with C53 antibody for 2.5 hrs, then protein A beads were added and incubated for another 1.5 hrs, the beads were washed and binding proteins were eluted.

4. We will test autophagic degradation of C53 in *uba5* and *ufc1* mutants under normal and tunicamycin stress conditions by performing autophagic flux assays using the native C53 antibody

We tested C53 autophagy flux in *uba5* knockdown and *ufc1* mutant lines (Fig. S12C, D). We detected decreased C53 degradation in *ufc1* mutant lines, suggesting that UFC1 is needed to enhance C53

recruitment to the ER membrane. Consistently, a recent study suggested that C53 might form a higher order complex with UFC1 (Peter J. et al., 2022).

5. Molecular docking studies to see C53's structural rearrangements leading to ATG8 and UFM1 binding.

To support our NMR observations and provide a better explanation on why UFM1/ATG8 may bind with different affinities to C53 sAIM1 and sAIM2, we have included two additional figures, Appendix Fig. S8C and Appendix Fig. S9C, showing how the backbone amide signals from residues downstream sAIM1 and sAIM2 shift upon addition of increasing concentrations of UFM1 and ATG8, respectively. As observed, additional contacts from neighboring residues apart from sAIM residues contribute to UFM1 and ATG8 binding, clearly showing that they play a major role in the differential recognition of both UBLs by each sAIM. To further support this conclusion with a quantitative assay, we have performed Fluorescence Anisotropy experiments with TAMRA-labelled C53-derived peptides harboring the individual sAIMs as well as a combination of sAIM1 and sAIM2 (sAIM1,2). In the newly included Appendix Fig. S8E and Appendix Fig. S9D, we show that the sAIM1,2 peptide binds both UBLs with higher affinity than the individual sAIM peptides, supporting our notion that additional neighboring residues to sAIMs contribute on the efficient recognition of both UBLs.

To better define the binding surface where HsC53 IDR interacts with HsUFM1 in Fig. EV3A, we have acquired additional 2D HSQC experiments of $^{15}N$-labelled HsUFM1 in the presence of unlabelled HsC53 IDR following a titration series. We have now confidently mapped the residues on HsUFM1 involved in C53 interaction and have modified the text accordingly. Using the newly defined HsUFM1 interaction site, we have performed molecular docking studies to provide more detailed information on the atomic details of this interaction discussed below.

To gain structural insights into how C53 IDR interacts with UBLs based on our NMR data, we have performed molecular docking studies on HADDOCK. As inputs, we used the AlphaFold prediction of HsC53 IDR (in teal), the solution NMR HsUFM1 structure (PDB:1WXS) and the X-ray crystal structure of GABARAP (PDB:6HB9). To filter down all models generated, we applied the following restrictions:

- At least one Trp (W) in HsC53 IDR must be in contact with HsUFM1/GABARAP surface.
- Residues on HsUFM1/GABARAP surface that are in contact with HsC53 IDR should match those for which we have observed the highest CSPs in our NMR titrations.

In line with the newly included Fluorescence Anisotropy data (Appendix Fig. S8E, Appendix Fig. S9D), the best models satisfying our restrictions docked HsC53 IDR sAIM1 and sAIM2 onto HsUFM1 (Fig. RP2A) or GABARAP (Fig. RP2B) surface. In the case of UFM1, C53 would dock in its hydrophobic pocket using Trp W269 (sAIM1) and Phe F272, while Trp W294 (sAIM2) would establish additional contacts. In the case of GABARAP, C53 would dock on both hydrophobic pockets HP1 and HP2 using Ile I292, Trp W294 (sAIM2) and Ile I296, while Trp W269 (sAIM1) would establish additional contacts with GABARAP's Lys K46. In both cases, the models agree with our observations and highlight the contribution of additional residues following sAIMs in stabilizing C53-UBLs interactions. However, the

models generated failed to fully explain our NMR data since additional residues on HsUFM1/GABARAP that didn't show CSPs in NMR experiments are predicted to contact C53 IDR. A plausible explanation would be that the C53 IDR is treated as a rather rigid entity when performing molecular docking studies, failing to capture its intrinsic dynamic behaviour in solution that would lead to different conformations upon its interaction with UBLs. Therefore, we decided to leave these models out of the revised manuscript.

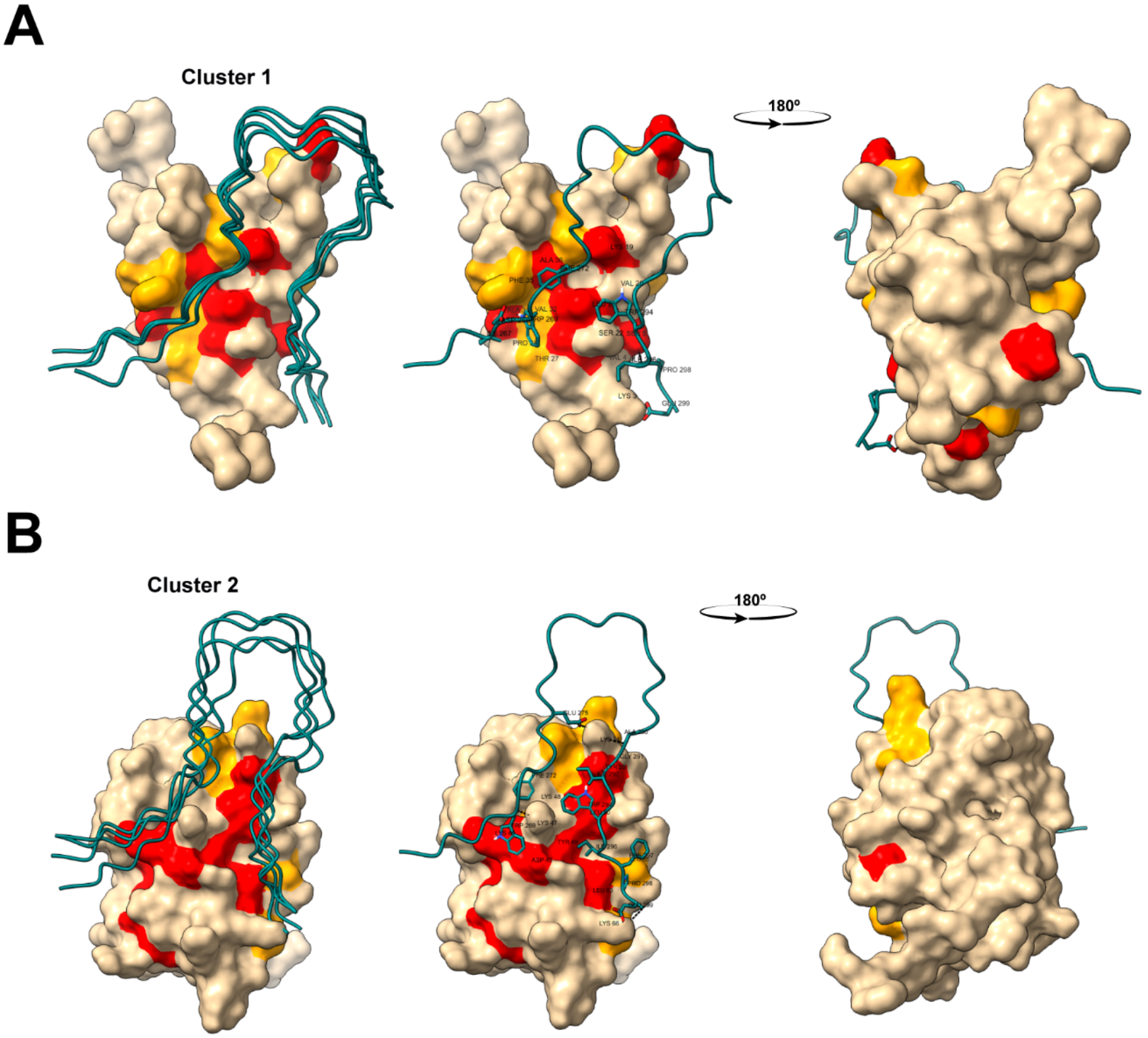


**Figure revision plan 2. Molecular docking of UFM1-IDR and ATG8-IDR provides three-dimensional structural models of C53-UBLs interaction**. Representative poses of cluster families for HsUFM1 (A) and GABARAP (B) binding to HsC53 IDR (teal). Left panel, superimposition of models within a cluster family. Right panel, representative interaction model. Modelled atoms involved in the interaction are displayed, modelled hydrogen bonds are shown as dashed lines. UFM1 (PDB: 1WXS) and GABARAP (PDB: 6HB9) structures are colored as in Figures EV3 and EV4.

6. Figure 5G has been revised with optimal background extraction parameters. The colocalization and the differences in puncta numbers are clearer now.

7. We have uploaded all the raw data to Zenodo. It is possible to download everything at the following link: https://doi.org/10.5281/zenodo.7313984

8. Test the interaction of 6 newly synthesised C53 isoforms from: (1) an alveolate (*tsAr*, *Ciliate*), (2) a stramenopile (*tSar*, *Phaeodactylum*), (3) a haptophyte (*Emiliania*), (4) a cryptophyte (*Guillardia*), (5) a diplomonad (*Trypanosoma*) and (6) a *CrRuM* with human and plant ATG8 and UFM1 proteins.

We added two species of CRuMs into all of the phylogenomic analyses (Fig. 1, Fig. 2) and added correlation statistics for the ufmylation proteins and C53 (Fig. EV1I). For the C53 alignment figure (Fig. 2C) the newly synthesized C53s were included.

We now synthesized 7 more C53 isoforms from two TSAR representatives (the alveolate, *Tetrahymena thermophila*, and the stramenopile, *Phytophthora sojae*), as well as a representative from Haptista (Emiliania), Cryptista (Guillardia), Diplomonada (Trypanosoma), Amoebozoa (Dictyostelium), and CRuMs (Rigifila). We have tested the interaction of these 7 new C53 orthologs with human and plant ATG8 and UFM1 proteins, using *in vitro* pulldowns. We detected interaction with all of them with different degrees of affinity (Fig. EV2). These results further support our phylogenomic correlations, since all these C53s have sAIMs in their sequence. They also complement the results from the divergent C53s analysed in Fig. 2C, D.

9. Further experiments:

We further explored the physiological differences between c53 complemented lines with either $C53^{wt}$, $C53^{sAIM}$ and $C53^{cAIM}$. We tried to identify if not only the roots but also the root hairs were affected in condition of ER stress caused by tunicamycin. As reported in Fig. RP3 and RP4, we were not able to see any difference among wild type and mutants.

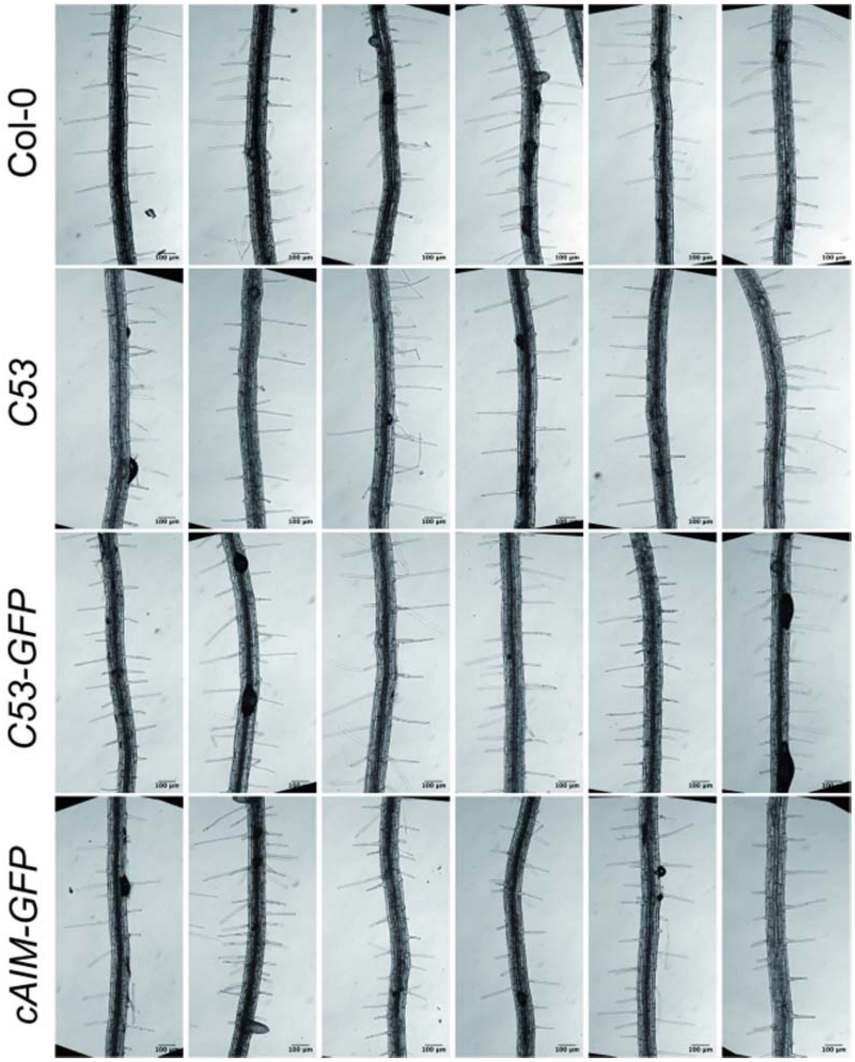


**Figure revision plan 3. Root hair phenotypes in control condition**. Representative root hair phenotypes of Col-0, *c53*, *C53^wt-GFP* and *C53^cAIM-GFP* plants. 6-day-old plants grown vertically on non-sucrose control plates were mounted and observed using Zeiss microscope Axio Observer Z1 (inverted) with sCMOS camera. Root hair from maturation zone is photographed. Scale bars = 100 µm.

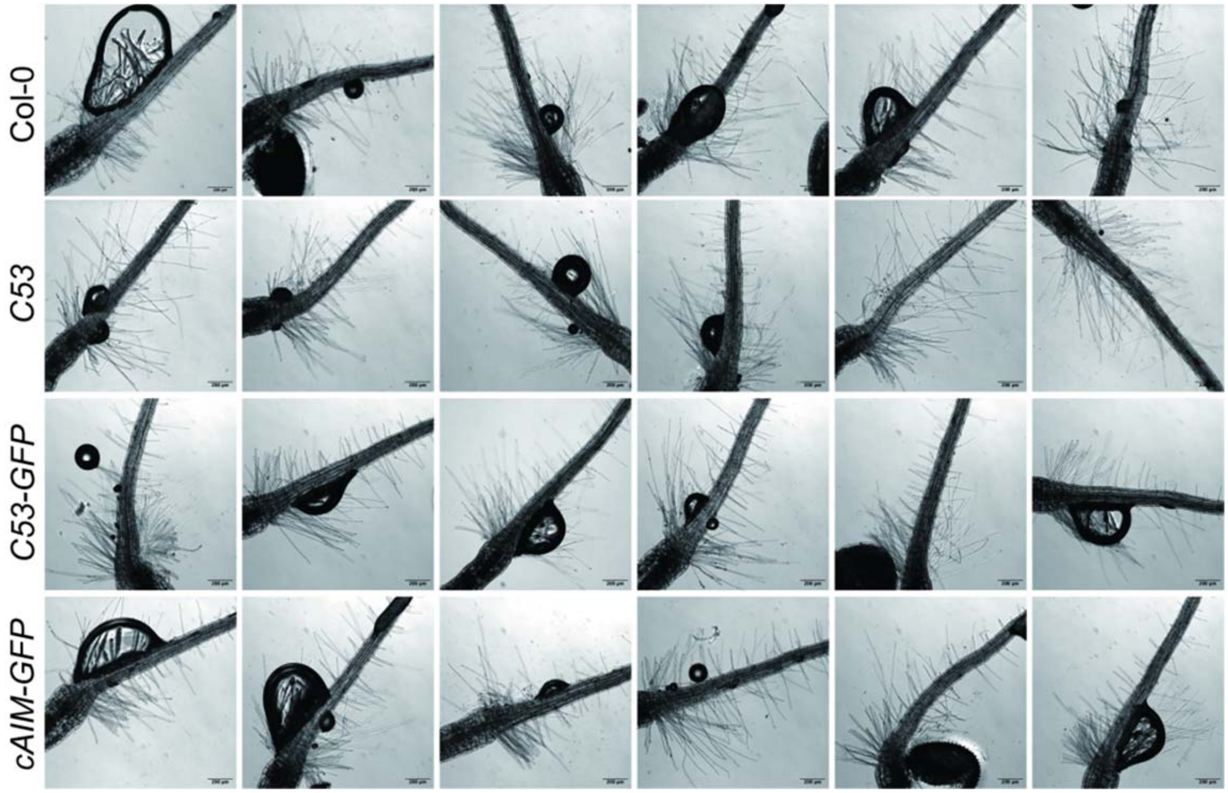


**Figure revision plan 4. Root hair phenotypes in ER-stress condition caused by tunicamycin**. Representative root hair phenotypes of Col-0, *c53*, *C53*$^{wt}$*-GFP* and *C53*$^{cAIM}$*-GFP* plants. 6-day-old plants grown vertically on non-sucrose plates supplemented with 100 ng/ml Tunicamycin were mounted and observed using Zeiss microscope Axio Observer Z1 (inverted) with sCMOS camera. Root hair under Tunicamycin stress condition is rather short, instead, root hair initiation zone is photographed. Scale bars = 200 µm.

Dear Yasin,

I have attached the re-review reports to the bottom of this email. As you will see there is still a substantial amount of work outlined, but I am sure some if not most of it can be addressed fairly painlessly and without any extra experiments. My suggestion is this: digest the reports over the next few days and let's try to schedule a Zoom meeting on Monday or Tuesday to finalise a plan of how to move forward.

Best wishes,

William

William Teale, PhD
Editor
The EMBO Journal
w.teale@embojournal.org

------------------------------------------------

Referee #1:

The authors addressed most of the concerns raised in the previous review and have improved their manuscript by making edits to the text and by including a substantial amount of new data. In particular, the inclusion of measurements of binding affinities for the interaction between C53 and ATG8 or UFM1 strengthen their conclusions. Even with the additional data, the findings of this manuscript do not constitute more than an incremental advance beyond their previously published work (Stephani et al., 2020). The impact of this manuscript would be considerably greater if the data presented were to provide mechanistic insight on the interplay between C53-mediated autophagy and UFMylation. The phylogenomic analysis is also incremental as two previously published studies reached similar main conclusions: ie that UFMylation was present in the last eukaryotic ancestor (LECA), and it was loss in Fungi and other lineages during evolution.

Additional issues that need to be addressed:

1) The new ITC and fluorescence anisotropy data should include statistics and appropriate labeling. Specifically, (a) there is no standard deviation for the KD shown in Figures EV5, S6, S8E, and S9D; (b) the label "IC50" for the fluorescence anisotropy data shown in Figures S8E and S9D should be replaced with KD, as these are not competition binding assays; (c) the Y axes for Figures S8E and S9D should note that the units are arbitrary.

2) The authors propose that C53 binds UFM1 under normal conditions, thus keeping it "inactive", and it switches preference towards ATG8 binding upon ER stress (e.g., triggered by tunicamycin) to "activate" C53-mediated autophagy. Although their model is intriguing, the in vivo pulldown results (Figs. 5F and S12A-B) contradict it, as both UFM1 and ATG8A immunoprecipitated by C53 increase upon tunicamycin treatment. Upon ER stress induction triggered by tunicamycin C53-UFM1 association should become weaker, as previously reported in Stephani et al, 2020 (see Figure 9C). Can the author clarify this discrepancy? Also, the data presented in Stephani et al. (Figure 9C) show that under normal conditions C53 binds to di-UFMylated RPL26, while Figures 5F and S12A-B of this manuscript show that C53 interacts with free UFM1 under both normal and ER stress conditions. Can the authors clarify this point, as it seems relevant for their proposed model? What species are immunoprecipitated by C53 (free UFM1 or di-UFMylated RPL26)?

3) The authors state (lines 43-45): "RPL26 UFMylation is triggered by the stalling of ER-bound ribosomes and is necessary for autophagic degradation of incomplete polypeptides trapped on the ribosomes (Wang et al, 2020; Liang et al, 2020)". This statement is inaccurate and should be modified. Wang et al. showed that RPL26 UFMylation facilitates degradation of incomplete arrested polypeptides through an autophagy-independent lysosomal pathway. On the contrary, Liang et al. reported that knockout of UFMylation genes impairs bulk ER-phagy but did not examine whether RPL26 UFMylation is required for clearance of incomplete polypeptides. To my knowledge there has been no published report that UFMylation is necessary for autophagic degradation of incomplete polypeptides.

4) The authors state (lines 45-46): "We have shown that C53 mediates the degradation of these incomplete polypeptides in an UFMylation-dependent manner (Stephani et al, 2020)." In Stephani et al., the authors showed that (a) overexpression of an ER-targeted poly-lysine (K20) stalling reporter (from Wang et al., 2020) in Hela cells generates foci that colocalize with C53 (Figure 7A - supplement 2), and (b) treatment of HeLa cells with high-dose elongation inhibitors results in the appearance of C53 foci (Figure 7C - supplement 2). However, they did not show whether degradation of incomplete polypeptides require C53-mediated autophagy. Their statement is a misleading overinterpretation of their data and should be revised to reflect the actual findings.

5) In the Abstract, the statement "Stalling of ER-bound ribosomes trigger their UFMylation and activates C53-mediated autophagy to clear toxic incomplete polypeptides" is also inaccurate as none of the previously published work have shown that UFMylation induces autophagic degradation of ER-targeted incomplete arrested polypeptides.

6) The citation (Stephani et al., 2020) to their previous work should be removed from the following statement (lines 395-398): "In ER homeostasis, UFMylation is activated by stalling of ER-bound ribosomes and brings about the degradation of incomplete polypeptides, which can be toxic for the cell (Wang et al, 2020; Stephani et al, 2020)".

Referee #2:

The authors improved their manuscript. Below are several points that still have to be addressed:

While purified ribosomes support ufmylated RPL26, it will be nice to strengthen these data. Does anti-Human RPL26 antibody identify CrRPL26? If yes, please show a blot with this antibody to confirm ufmylated RPL26. Regarding the mass spec data, please add a control of Cr lacking UFL1 to show that there is no ufmylated RPL26 peptide.

In the cell lysate, can the authors suggest why they didn't detect ufmylated UFC1 or UBA5. Can they show that in the absence of BME, charged UFC1 is detected?

Since the method presented in fig 3C&D allows the detection of interactions with low affinity, please use this approach to show the competition between ATG8 and UFM1 on C53. Specifically, perform the experiment as in fig. 3D, but instead of adding cAIM peptide add untagged ATG8/ GABARP. In that case, ATG8 binds C53 and therefore, no binding of mCH-C53 will be detected.

The authors claim that they cannot reach a high concentration of UFM1. Is this because they use GST-UFM1? In other words, the problem could be due to GST that enforces the dimerization of UFM1.

Referee #3:

The authors have tackled all of my suggestions to my full satisfaction. I do not have any additional comments and congratulate the authors on a very nice paper.

Dear Editors,

Please see our point-by-point response to the reviewer's comments below. Despite agreeing that we have performed extensive revisions based on their requests, Reviewer 1 and Reviewer 2 asked for additional experiments that we think are not necessary to support the main conclusions of this manuscript. We already have 5 main, 5 extended view, and 12 supplemental figures. We do not want to add more experiments that will dilute the focus of our story.

Looking forward to hearing from you.

Sincerely,

Yasin Dagdas

Referee #1:

The authors addressed most of the concerns raised in the previous review and have improved their manuscript by making edits to the text and by including a substantial amount of new data. In particular, the inclusion of measurements of binding affinities for the interaction between C53 and ATG8 or UFM1 strengthen their conclusions. Even with the additional data, the findings of this manuscript do not constitute more than an incremental advance beyond their previously published work (Stephani et al., 2020). The impact of this manuscript would be considerably greater if the data presented were to provide mechanistic insight on the interplay between C53-mediated autophagy and UFMylation. The phylogenomic analysis is also incremental as two previously published studies reached similar main conclusions: ie that UFMylation was present in the last eukaryotic ancestor (LECA), and it was loss in Fungi and other lineages during evolution.

We thank the reviewer for critically evaluating our manuscript. We agree with the reviewer that the interplay between C53-mediated autophagy and UFMylation is highly exciting and needs more investigation. However, that is not the focus of this manuscript. Here, we are focusing on the sAIMs that link C53 to UFMylation. Nevertheless, the reviewer will be pleased to know that we are working on another manuscript that will address their question.

Regarding their comment on whether our phylogenetic analyses are incremental or not, we are copying our response from the previous rebuttal letter below. In addition to these points, our recent analyses also revealed UFL1 and DDRGK1 homologs in Asgard archaea. This was not discovered in any of the previous studies cited by the reviewer and we think this alone is a highly significant finding. In addition, as we detailed below, previous studies only made suggestions based on evolutionary conservation. Here, we have tested those suggestions using biochemical assays. For example, we synthesized several C53 homologs from various eukaryotic taxa (as suggested by reviewers) and assessed their UFM1 and ATG8 binding. Or, we have shown biochemically and physiologically that UFMylation is functional in a unicellular organism. Reducing these findings to “incremental” is not fair.

From the previous letter:
We were aware that previous studies had investigated the distribution of UFMylation proteins across eukaryotes and that these analyses had predicted the presence of UFMylation in LECA and subsequent loss in Fungi. We included a more recent citation noting this (Tsaban et al. 2021) but apologise for not citing Grau-Bové et al. (2014), which we have now included.

We must emphasize that our results are not incremental. Although we had made a point of emphasizing the presence of UFM1 in LECA, this was to counter a recent and highly cited paper in the field which claimed that UFMylation evolved in plants and animals (Walczak et al. 2019). Below we note the novel and important results from our phylogenomic analyses:

1. We used improved taxonomic sampling and more advanced comparative genomics methods to identify UFMylation components sensitively and specifically across eukaryotes. This involved the inclusion of additional eukaryotic genomes, phylogenetic annotation of orthologs, and genomic searches to complement proteome predictions. These methods are essential for accurately identifying UFMylation components and yield more robust results than using sequence similarity clustering (Tsaban et al. 2021) or un-curated Pfam HMMER search results (Grau-Bové et al. 2014).

2. By placing our UFMylation reconstructions in a modern phylogenetic context, we were not only able to support previous observations which noted the presence of UFM1 in LECA and its loss in Fungi (Grau-Bové et al. 2014) and *Plasmodium* (Tsaban et al. 2021), but also to identify novel patterns in the evolution of UFMylation. This included the observation of recurrent losses in diverse but trophically-related lineages (such as algae and parasites) and revealed the retention of certain UFMylation components in the absence of UFM1. We identified the frequent co-retention of UFL1 and DDRGK1 following UFM1 loss in multiple eukaryotic groups, including Fungi, which were previously thought to be devoid of UFMylation machinery. These previously uncharacterized patterns, suggest that these proteins could have alternative functions and may be functionally associated with life history. These results therefore expand on and add complexity to our understanding of the evolution of UFMylation.

3. By conducting a comprehensive and accurate survey of UFMylation components we were able to use our data to examine co-evolutionary trends between C53 and UFM1, which would have been incomplete and inaccurate using previously curated datasets. As the reviewer noted, only five species were identified that encoded C53 but lacked UFM1. This is not a reflection of insufficient taxon sampling, but rather the strong co-evolution between C53 and UFM1 (i.e., when UFM1 is lost, C53 is almost always lost as well). We attempted to identify additional cases by searching hundreds of fungal and oomycete genomes as well as those from other eukaryotes, but no other species were found. We agree with the reviewer that additional taxa would have made our analyses stronger, but importantly, we do not rely on genomic correlations to infer function. Rather, we use these correlations to generate functional hypotheses which we then tested experimentally. In this way, we do not rely on the strength of our correlations.

Additional issues that need to be addressed:

1) The new ITC and fluorescence anisotropy data should include statistics and appropriate labeling. Specifically, (a) there is no standard deviation for the KD shown in Figures EV5, S6, S8E, and S9D; (b) the label "IC50" for the fluorescence anisotropy data shown in Figures S8E and S9D should be replaced with KD, as these are not competition binding assays; (c) the Y axes for Figures S8E and S9D should note that the units are arbitrary.

Regarding the ITC experiments in Figure EV5 and S6, we have already included the error analysis for the $K_D$ by using a confidence level of 0.683, which corresponds to one s.d. in the case of Gaussian error distribution (Brautigam C. A. et al., 2016; Zhao H. et al., 2014). In general, it is more accurate to express uncertainties with confidence intervals rather than +/- s.d. (Paketurytė V. et al., 2021).

Regarding the fluorescence anisotropy experiments, Figures S8E and S9D now include a 95% confidence interval (C.I.). The reviewer rightly points out that $K_D$ is the factor measured by Fluorescence Anisotropy, not the IC50. We have modified Figures S8E and S9D, accordingly. We have also modified the Y axis to 'Fluorescence Anisotropy (A.U.)' for clarification. Figure legends have also been changed.

2) The authors propose that C53 binds UFM1 under normal conditions, thus keeping it "inactive", and it switches preference towards ATG8 binding upon ER stress (e.g., triggered by tunicamycin) to "activate" C53-mediated autophagy. Although their model is intriguing, the in vivo pulldown results (Figs. 5F and S12A-B) contradict it, as both UFM1 and ATG8A immunoprecipitated by C53 increase upon tunicamycin treatment. Upon ER stress induction triggered by tunicamycin C53-UFM1 association should become weaker, as previously reported in Stephani et al, 2020 (see Figure 9C). Can the author clarify this discrepancy? Also, the data presented in Stephani et al. (Figure 9C) show that under normal conditions C53 binds to di-UFMylated RPL26, while Figures 5F and S12A-B of this manuscript show that C53 interacts with free UFM1 under both normal and ER stress conditions. Can the authors clarify this point, as it seems relevant for their proposed model? What species are immunoprecipitated by C53 (free UFM1 or di-UFMylated RPL26)?

As the reviewers also pointed out, we have performed extensive characterization of the UFM1-ATG8 competition, using *in vivo* and *in vitro* experiments. Particularly, we think the sAIM to cAIM conversion experiments are really crucial, as they only change the ATG8 and UFM1 affinity of C53. In those experiments, we clearly saw a shift towards ATG8 binding in both *in vitro* and *in vivo* experiments. Based on these results, we put together the ATG8-UFM1 competition model. Regarding the coIP experiments, indeed in this manuscript we focused on the free UFM1 levels and compared those to ATG8. As suggested by the reviewer, we will explore the C53-UFMylation crosstalk further and investigate whether there's any difference in terms of conjugated vs. free UFM1 binding. Our current ribosome profiling proteomics experiments suggest C53 could associate with UFMylated RPL26. But as we explained above, this is not the focus of this manuscript and we will share these findings in another manuscript.

3) The authors state (lines 43-45): "RPL26 UFMylation is triggered by the stalling of ER-bound ribosomes and is necessary for autophagic degradation of incomplete polypeptides trapped on the ribosomes (Wang et al, 2020; Liang et al, 2020)". This statement is inaccurate and should be modified. Wang et al. showed that RPL26 UFMylation facilitates degradation of incomplete arrested polypeptides through an autophagy-independent lysosomal pathway. On the contrary, Liang et al. reported that knockout of UFMylation genes impairs bulk ER-phagy but did not examine whether RPL26 UFMylation is required for clearance of incomplete polypeptides. To my knowledge there has been no published report that UFMylation is necessary for autophagic degradation of incomplete polypeptides.

We have modified this statement as "RPL26 UFMylation is triggered by the stalling of ER-bound ribosomes and contributes to the degradation of incomplete polypeptides trapped on the ribosomes".

4) The authors state (lines 45-46): "We have shown that C53 mediates the degradation of these incomplete polypeptides in an UFMylation-dependent manner (Stephani et al, 2020)." In Stephani et al., the authors showed that (a) overexpression of an ER-targeted poly-lysine (K20) stalling reporter (from Wang et al., 2020) in Hela cells generates foci that colocalize with C53 (Figure 7A - supplement 2), and (b) treatment of HeLa cells with high-dose elongation

inhibitors results in the appearance of C53 foci (Figure 7C - supplement 2). However, they did not show whether degradation of incomplete polypeptides require C53-mediated autophagy. Their statement is a misleading overinterpretation of their data and should be revised to reflect the actual findings.

We thank the reviewer for carefully analyzing our previous findings. As the reviewer can see in Stephani et al., we also had C53 knockdown experiments, presented in Fig.7-figure supplement 2D, where we have shown that C53 is required for lysosomal delivery of ER-K20. In the same manuscript, we have also shown extensive evidence that C53 degradation is mediated by autophagy (presented in Fig2). We understand that the reviewer would like to emphasize the recent findings from a very recent preprint from the Ye lab (Wang et al., BioRxiv, 2022), where the authors suggested that the lysosomal degradation of ER-K20 is mediated via a Golgi pathway. However, we would like to point out that the adaptor protein that was identified by Wang et al. is not conserved in plants, suggesting that is not the only mechanism for ER-K20 degradation. As we pointed out above, we agree with the reviewer that this mechanism needs further investigation and we are exploring it further, using our multi-species approach.

5) In the Abstract, the statement "Stalling of ER-bound ribosomes trigger their UFMylation and activates C53-mediated autophagy to clear toxic incomplete polypeptides" is also inaccurate as none of the previously published work have shown that UFMylation induces autophagic degradation of ER-targeted incomplete arrested polypeptides.

Please see above.

6) The citation (Stephani et al., 2020) to their previous work should be removed from the following statement (lines 395-398): "In ER homeostasis, UFMylation is activated by stalling of ER-bound ribosomes and brings about the degradation of incomplete polypeptides, which can be toxic for the cell (Wang et al, 2020; Stephani et al, 2020)".

We disagree with the reviewer. If they look at our findings presented in Figure 7 (Stephani et al. 2020), they can see that we have shown the link between ribosome stalling and autophagy.

Referee #2:

The authors improved their manuscript. Below are several points that still have to be addressed:

We thank the reviewer for appreciating our revisions.

While purified ribosomes support ufmylated RPL26, it will be nice to strengthen these data. Does anti-Human RPL26 antibody identify CrRPL26? If yes, please show a blot with this antibody to confirm ufmylated RPL26. Regarding the mass spec data, please add a control of Cr lacking UFL1 to show that there is no ufmylated RPL26 peptide.

We would like to point out that we have shown that UFM1 antibody detects the same bands from total lysates and isolated ribosomes, and this band is only present in wild-type cells but not in *ufl1* mutants (Fig. S3). We then went further and analyzed these bands using mass spectrometry and showed that these are indeed UFMylated RPL26 bands. We think this is quite extensive validation. We will now provide the mass spectrometry data obtained from *ufl1* cells, where we did not detect any RPL26 UFMylation. (SourceDataForFigure3E.xls).

In the cell lysate, can the authors suggest why they didn't detect ufmylated UFC1 or UBA5. Can they show that in the absence of BME, charged UFC1 is detected?

The *in vivo* experiments (Figure 1C, Figure S3C) specifically focus on detecting substrates of the UFMylation machinery that are modified with UFM1 via an isopeptide bond to lysine. Thus, a reducing agent (BME) was used to break thioester bonds such as UBA5-UFM1 and UFC1-UFM1. In fact, we have already shown *in vitro* that UBA5 and UFC1 form a thioester bond with UFM1 that can be reduced by the addition of BME (Figure 1B).

Since the method presented in fig 3C&D allows the detection of interactions with low affinity, please use this approach to show the competition between ATG8 and UFM1 on C53. Specifically, perform the experiment as in fig. 3D, but instead of adding cAIM peptide add untagged ATG8/ GABARP. In that case, ATG8 binds C53 and therefore, no binding of mCH-C53 will be detected.

We have previously reported that ATG8 can outcompete UFM1-C53 interaction in a concentration-dependent manner using *in-vitro* pulldown experiments (Stephani et al. 2020, Figure 9A). In the present manuscript, we have also shown that GABARAP outcompetes the UFM1-C53 interaction using microscopy-based protein-protein interaction assays (Figure S7). Performing a titration series as suggested by the reviewer would not provide additional information to this manuscript.

The authors claim that they cannot reach a high concentration of UFM1. Is this because they use GST-UFM1? In other words, the problem could be due to GST that enforces the dimerization of UFM1.

We agree with the reviewer that our statement needs further clarification. We used purified His-tagged UFM1 for biophysical and structural biology studies in this manuscript. Although we can reach a relatively high concentration of His-tagged UFM1 (~600 µM) that allows us to perform NMR, ITC, and Fluorescence Anisotropy studies, we would require higher UFM1 concentrations than what we can achieve to perform *in vitro* competition experiments, e.g. outcompeting the ATG8-C53 interaction, due to the difference in affinities for both interactions. While we have previously reported a $K_D$ in the low micromolar range for ATG8-C53 interaction by ITC (Stephani et al. 2020, Figure 4D), in this manuscript we failed to obtain Kd values for UFM1-C53 interaction by the same method, probably due to the transient nature of the interaction, and could only report a $K_D$ in the high micromolar range for UFM1-C53 sAIM1,2 peptide interaction by Fluorescence Anisotropy (Figure S8E). Thus, to perform competition experiments we would require purified UFM1 to molarities that cannot be reached, as protein aggregates at higher concentrations than stated above.

Referee #3:

The authors have tackled all of my suggestions to my full satisfaction. I do not have any additional comments and congratulate the authors on a very nice paper.

Thank you!

Dear Yasin,

Before I can finally accept manuscript EMBOJ-2022-112053, there are some remaining editorial points which need to be addressed. Would you therefore please:

- include up to five keywords
- update the Conflict of Interest statement, to a Disclosure and Competing Interests statement
- remove the Author Contributions section from the manuscript
- include callouts for Figures EV1, EV5 and panel callouts for Appendix Figures S2, S5 and S6
- include reference to the legends of the two Supplemental Data files in the Table of Contents of Appendix 1.

In addition,

- the Materials and Methods section should follow directly after the Discussion section,
- the Reagent Table should be uploaded separately using the file type 'Reagent Table',
- please acknowledge the duplication of control panels in the legend of figures 3c and 3d to avoid any suspicion in the future,
- please also remove the error bars in Fig1c, as n=2. A bar may be used to indicate the range of the data if this is explained in the legend.

EMBO Press is an editorially independent publishing platform for the development of EMBO scientific publications.

Best wishes,

William

William Teale
Editor
The EMBO Journal

------------------------------------------------

***
Rev_Com_number: RC-2022-01458
New_manu_number: EMBOJ-2022-112053R1
Corr_author: Dagdas
Title: Shuffled ATG8 interacting motifs form an ancestral bridge between UFMylation and autophagy

EMBOJ-2022-112053

Dear Dr. Teale,

We are pleased to hear that our manuscript is close to acceptance. We have modified the manuscript and figures as you suggested. Please see the details below. We have also uploaded the manuscript with track changes.

Yours sincerely,

Yasin Dagdas

Before I can finally accept manuscript EMBOJ-2022-112053, there are some remaining editorial points which need to be addressed. Would you therefore please:

- include up to five keywords

Included them right after the Abstract

- update the Conflict of Interest statement, to a Disclosure and Competing Interests statement

Updated.

- remove the Author Contributions section from the manuscript

Removed.

- include callouts for Figures EV1, EV5 and panel callouts for Appendix Figures S2, S5 and S6

Included.

- include reference to the legends of the two Supplemental Data files in the Table of Contents of Appendix 1.

Included.

In addition,

- the Materials and Methods section should follow directly after the Discussion section,

Reorganized the manuscript as you suggested.

- the Reagent Table should be uploaded separately using the file type 'Reagent Table',

We have removed it from the main text and uploaded it as The Reagents Table.

- please acknowledge the duplication of control panels in the legend of figures 3c and 3d to avoid any suspicion in the future,

We apologize for this. To avoid any confusion, we have replaced those images. There's no duplication now.

- please also remove the error bars in Fig1c, as n=2. A bar may be used to indicate the range of the data if this is explained in the legend.

Removed the error bars and modified the legend.

## 3rd Revision - Editorial Decision 18th Jan 2023

Dear Yasin,

I am pleased to inform you that your manuscript has been accepted for publication in the EMBO Journal.

Congratulations on a really interesting study!

------------------------------------------------

Please note that it is EMBO Journal policy for the transcript of the editorial process (containing referee reports and your response letter) to be published as an online supplement to each paper. If you do NOT want this, you will need to inform the Editorial Office via email immediately. More information is available here:
https://emboj.embopress.org/about#Transparent_Process

Your manuscript will be processed for publication in the journal by EMBO Press. Manuscripts in the PDF and electronic editions of The EMBO Journal will be copy edited, and you will be provided with page proofs prior to publication. Please note that supplementary information is not included in the proofs.

Please note that you will be contacted by Wiley Author Services to complete licensing and payment information. The 'Page Charges Authorization Form' is available here: https://www.embopress.org/pb-assets/embo-site/tej_apc.pdf

EMBO Press participates in many Publish and Read agreements that allow authors to publish Open Access with reduced/no publication charges. Check your eligibility: https://authorservices.wiley.com/author-resources/Journal-Authors/open-access/affiliation-policies-payments/index.html

Should you be planning a Press Release on your article, please get in contact with embojournal@wiley.com as early as possible, in order to coordinate publication and release dates.

If you have any questions, please do not hesitate to call or email the Editorial Office. Thank you for your contribution to The EMBO Journal.

Yours sincerely,

William

William Teale, PhD
Editor
The EMBO Journal
w.teale@embojournal.org

***
Rev_Com_number: RC-2022-01458
New_manu_number: EMBOJ-2022-112053R2
Corr_author: Dagdas
Title: Shuffled ATG8 interacting motifs form an ancestral bridge between UFMylation and autophagy

## EMBO Press Author Checklist

| | |
|---|---|
| Corresponding Author Name: Elif G. Karagöz, Yasin Dagdas | |
| Journal Submitted to: The EMBO Journal | |
| Manuscript Number: EMBOJ-2022-112053 | |

**USEFUL LINKS FOR COMPLETING THIS FORM**
The EMBO Journal - Author Guidelines
EMBO Reports - Author Guidelines
Molecular Systems Biology - Author Guidelines
EMBO Molecular Medicine - Author Guidelines

**Reporting Checklist for Life Science Articles (updated January**
This checklist is adapted from Materials Design Analysis Reporting (MDAR) Checklist for Authors. MDAR establishes a minimum set of requirements in transparent reporting in the life sciences (see Statement of Task: 10.31222/osf.io/9sm4x). Please follow the journal's guidelines in preparing your
**Please note that a copy of this checklist will be published alongside your article.**

**Abridged guidelines for figures**
**1. Data**
The data shown in figures should satisfy the following conditions:
- the data were obtained and processed according to the field's best practice and are presented to reflect the results of the experiments in an accurate and unbiased manner.
- ideally, figure panels should include only measurements that are directly comparable to each other and obtained with the same assay.
- plots include clearly labeled error bars for independent experiments and sample sizes. Unless justified, error bars should not be shown for technical
- if n<5, the individual data points from each experiment should be plotted. Any statistical test employed should be justified.
- Source Data should be included to report the data underlying figures according to the guidelines set out in the authorship guidelines on Data

**2. Captions**
Each figure caption should contain the following information, for each panel where they are relevant:
- a specification of the experimental system investigated (eg cell line, species name).
- the assay(s) and method(s) used to carry out the reported observations and measurements.
- an explicit mention of the biological and chemical entity(ies) that are being measured.
- an explicit mention of the biological and chemical entity(ies) that are altered/varied/perturbed in a controlled manner.
- the exact sample size (n) for each experimental group/condition, given as a number, not a range;
- a description of the sample collection allowing the reader to understand whether the samples represent technical or biological replicates (including how many animals, litters, cultures, etc.).
- a statement of how many times the experiment shown was independently replicated in the laboratory.
- definitions of statistical methods and measures:
  - common tests, such as t-test (please specify whether paired vs. unpaired), simple χ2 tests, Wilcoxon and Mann-Whitney tests, can be unambiguously identified by name only, but more complex techniques should be described in the methods section;
  - are tests one-sided or two-sided?
  - are there adjustments for multiple comparisons?
  - exact statistical test results, e.g., P values = x but not P values < x;
  - definition of 'center values' as median or average;
  - definition of error bars as s.d. or s.e.m.

**Please complete ALL of the questions below.**
**Select "Not Applicable" only when the requested information is not relevant for your study.**

**Materials**

| Newly Created Materials | Information included in the manuscript? | In which section is the information available? (Reagents and Tools Table, Materials and Methods, Figures, Data Availability Section) |
|---|---|---|
| New materials and reagents need to be available; do any restrictions apply? | Yes | Materials and Methods |

| Antibodies | Information included in the manuscript? | In which section is the information available? (Reagents and Tools Table, Materials and Methods, Figures, Data Availability Section) |
|---|---|---|
| For **antibodies** provide the following information:<br>- Commercial antibodies: RRID (if possible) or supplier name, catalogue number and or/clone number<br>- Non-commercial: RRID or citation | Yes | Materials and Methods |

| DNA and RNA sequences | Information included in the manuscript? | In which section is the information available? (Reagents and Tools Table, Materials and Methods, Figures, Data Availability Section) |
|---|---|---|
| **Short novel DNA or RNA including primers, probes**: provide the sequences. | Yes | Materials and Methods |

| Cell materials | Information included in the manuscript? | In which section is the information available? (Reagents and Tools Table, Materials and Methods, Figures, Data Availability Section) |
|---|---|---|
| **Cell lines:** Provide species information, strain. Provide accession number in repository **OR** supplier name, catalog number, clone number, and/**OR** RRID. | Not Applicable | |
| **Primary cultures:** Provide species, strain, sex of origin, genetic modification status. | Not Applicable | |
| Report if the cell lines were recently **authenticated** (e.g., by STR profiling) and tested for mycoplasma contamination. | Not Applicable | |

| Experimental animals | Information included in the manuscript? | In which section is the information available? (Reagents and Tools Table, Materials and Methods, Figures, Data Availability Section) |
|---|---|---|
| **Laboratory animals or Model organisms:** Provide species, strain, sex, age, genetic modification status. Provide accession number in repository **OR** supplier name, catalog number, clone number, **OR** RRID. | Not Applicable | |
| **Animal observed in or captured from the field:** Provide species, sex, and age where possible**.** | Not Applicable | |
| Please detail **housing and husbandry conditions**. | Not Applicable | |

| Plants and microbes | Information included in the manuscript? | In which section is the information available? (Reagents and Tools Table, Materials and Methods, Figures, Data Availability Section) |
|---|---|---|
| **Plants:** provide species and strain, ecotype and cultivar where relevant, unique accession number if available, and source (including location for collected wild specimens). | Yes | Materials and Methods |
| **Microbes:** provide species and strain, unique accession number if available, and source. | Yes | Materials and Methods |

| Human research participants | Information included in the manuscript? | In which section is the information available? (Reagents and Tools Table, Materials and Methods, Figures, Data Availability Section) |
|---|---|---|
| If collected and within the bounds of privacy constraints report on age, sex and gender or ethnicity for all study participants. | Not Applicable | |

| Core facilities | Information included in the manuscript? | In which section is the information available? (Reagents and Tools Table, Materials and Methods, Figures, Data Availability Section) |
|---|---|---|
| If your work benefited from core facilities, was their service mentioned in the acknowledgments section? | Yes | Acknowledgments |

**Design**

| Study protocol | Information included in the manuscript? | In which section is the information available? (Reagents and Tools Table, Materials and Methods, Figures, Data Availability Section) |
|---|---|---|
| If study protocol has been **pre-registered, provide DOI in the manuscript**. For clinical trials, provide the trial registration number **OR** cite DOI. | Not Applicable | |
| Report the **clinical trial registration number** (at ClinicalTrials.gov or equivalent), where applicable. | Not Applicable | |

| Laboratory protocol | Information included in the manuscript? | In which section is the information available? (Reagents and Tools Table, Materials and Methods, Figures, Data Availability Section) |
|---|---|---|
| Provide DOI OR other citation details if **external detailed step-by-step protocols** are available. | Not Applicable | |

| Experimental study design and statistics | Information included in the manuscript? | In which section is the information available? (Reagents and Tools Table, Materials and Methods, Figures, Data Availability Section) |
|---|---|---|
| Include a statement about **sample size** estimate even if no statistical methods were used. | Yes | Figures, Materials and Methods |
| Were any steps taken to minimize the effects of subjective bias when allocating animals/samples to treatment (e.g. **randomization procedure**)? If yes, have they been described? | Yes | |
| Include a statement about **blinding** even if no blinding was done. | Not Applicable | |
| Describe **inclusion/exclusion criteria** if samples or animals were excluded from the analysis. Were the criteria pre-established?<br><br>If sample or data points were omitted from analysis, report if this was due to attrition or intentional exclusion and provide justification. | Not Applicable | |
| For every figure, are **statistical tests** justified as appropriate? Do the data meet the assumptions of the tests (e.g., normal distribution)? Describe any methods used to assess it. Is there an estimate of variation within each group of data? Is the variance similar between the groups that are being statistically compared? | Yes | Figures, Materials and Methods |

| Sample definition and in-laboratory replication | Information included in the manuscript? | In which section is the information available? (Reagents and Tools Table, Materials and Methods, Figures, Data Availability Section) |
|---|---|---|
| In the figure legends: state number of times the experiment was **replicated** in laboratory. | Yes | Figures |
| In the figure legends: define whether data describe **technical or biological replicates**. | Yes | Figures |

## Ethics

| Ethics | Information included in the manuscript? | In which section is the information available? (Reagents and Tools Table, Materials and Methods, Figures, Data Availability Section) |
|---|---|---|
| Studies involving **human participants**: State details of **authority granting ethics approval** (IRB or equivalent committee(s), provide reference number for approval. | Not Applicable | |
| Studies involving **human participants**: Include a statement confirming that **informed consent** was obtained from all subjects and that the experiments conformed to the principles set out in the WMA Declaration of Helsinki and the Department of Health and Human Services Belmont Report. | Not Applicable | |
| Studies involving **human participants:** For publication of **patient photos**, include a statement confirming that consent to publish was obtained. | Not Applicable | |
| Studies involving experimental **animals**: State details of **authority granting ethics approva**l (IRB or equivalent committee(s), provide reference number for approval. Include a statement of compliance with ethical regulations. | Not Applicable | |
| Studies involving **specimen and field samples:** State if relevant **permits** obtained, provide details of authority approving study; if none were required, explain why. | Not Applicable | |

| Dual Use Research of Concern (DURC) | Information included in the manuscript? | In which section is the information available? (Reagents and Tools Table, Materials and Methods, Figures, Data Availability Section) |
|---|---|---|
| Could your study fall under dual use research restrictions? Please check biosecurity documents and list of **select agents and toxins** (CDC): https://www.selectagents.gov/sat/list.htm | Not Applicable | |
| If you used a select agent, is the security level of the lab appropriate and reported in the manuscript? | Not Applicable | |
| If a study is subject to dual use research of concern regulations, is the name of the **authority granting approval and reference number** for the regulatory approval provided in the manuscript? | Not Applicable | |

## Reporting

The MDAR framework recommends adoption of discipline-specific guidelines, established and endorsed through community initiatives. Journals have their own policy about requiring specific guidelines and recommendations to complement MDAR.

| Adherence to community standards | Information included in the manuscript? | In which section is the information available? (Reagents and Tools Table, Materials and Methods, Figures, Data Availability Section) |
|---|---|---|
| State if relevant guidelines or checklists (e.g., **ICMJE, MIBBI, ARRIVE, PRISMA**) have been followed or provided. | Not Applicable | |
| For **tumor marker prognostic studies,** we recommend that you follow the **REMARK** reporting guidelines (see link list at top right). See author guidelines, under 'Reporting Guidelines'. Please confirm you have followed these guidelines. | Not Applicable | |
| For **phase II and III randomized controlled trials**, please refer to the **CONSORT** flow diagram (see link list at top right) and submit the CONSORT checklist (see link list at top right) with your submission. See author guidelines, under 'Reporting Guidelines'. Please confirm you have submitted this list. | Not Applicable | |

## Data Availability

| Data availability | Information included in the manuscript? | In which section is the information available? (Reagents and Tools Table, Materials and Methods, Figures, Data Availability Section) |
|---|---|---|
| Have **primary datasets** been deposited according to the journal's guidelines (see 'Data Deposition' section) and the respective accession numbers provided in the Data Availability Section? | Yes | Data availability, Matherials and Methods, Figures |
| Were **human clinical and genomic datasets** deposited in a public access-controlled repository in accordance to ethical obligations to the patients and to the applicable consent agreement? | Not Applicable | |
| Are **computational models** that are central and integral to a study available without restrictions in a machine-readable form? Were the relevant accession numbers or links provided? | Yes | Data availability, Matherials and Methods |
| If publicly available data were reused, provide the respective **data citations in the reference list**. | Not Applicable | |
